# Leopard (*Panthera pardus*) status, distribution, and the research efforts across its range

**DOI:** 10.7717/peerj.1974

**Published:** 2016-05-04

**Authors:** Andrew P. Jacobson, Peter Gerngross, Joseph R. Lemeris Jr., Rebecca F. Schoonover, Corey Anco, Christine Breitenmoser-Würsten, Sarah M. Durant, Mohammad S. Farhadinia, Philipp Henschel, Jan F. Kamler, Alice Laguardia, Susana Rostro-García, Andrew B. Stein, Luke Dollar

**Affiliations:** 1 Institute of Zoology, Zoological Society of London, London, United Kingdom; 2 Department of Geography, University College London, London, United Kingdom; 3 Big Cats Initiative, National Geographic Society, Washington, D.C., United States; 4 BIOGEOMAPS, Vienna, Austria; 5 Department of Biological Sciences, Fordham University, Bronx, NY, United States; 6 IUCN/SSC Cat Specialist Group, c/o KORA, Bern, Switzerland; 7 Wildlife Conservation Society, Bronx Zoo, Bronx, NY, United States; 8 Iranian Cheetah Society (ICS), Tehran, Iran; 9 Wildlife Conservation Research Unit, The Recanati-Kaplan Centre, Department of Zoology, University of Oxford, Tubney, Oxfordshire, United Kingdom; 10 Panthera, New York, NY, United States; 11 The Wildlife Institute, Beijing Forestry University, Beijing, China; 12 Landmark College, Putney, VT, United States; 13 Department of Biology, Pfeiffer University, Misenheimer, NC, United States; 14 Nicholas School of the Environment, Duke University, Durham, NC, United States

**Keywords:** Leopard, *Panthera pardus*, Decline, Distribution, Carnivore conservation

## Abstract

The leopard’s (*Panthera pardus*) broad geographic range, remarkable adaptability, and secretive nature have contributed to a misconception that this species might not be severely threatened across its range. We find that not only are several subspecies and regional populations critically endangered but also the overall range loss is greater than the average for terrestrial large carnivores. To assess the leopard’s status, we compile 6,000 records at 2,500 locations from over 1,300 sources on its historic (post 1750) and current distribution. We map the species across Africa and Asia, delineating areas where the species is confirmed present, is possibly present, is possibly extinct or is almost certainly extinct. The leopard now occupies 25–37% of its historic range, but this obscures important differences between subspecies. Of the nine recognized subspecies, three (*P. p. pardus, fusca,* and *saxicolor*) account for 97% of the leopard’s extant range while another three (*P. p. orientalis, nimr,* and *japonensis*) have each lost as much as 98% of their historic range. Isolation, small patch sizes, and few remaining patches further threaten the six subspecies that each have less than 100,000 km^2^ of extant range. Approximately 17% of extant leopard range is protected, although some endangered subspecies have far less. We found that while leopard research was increasing, research effort was primarily on the subspecies with the most remaining range whereas subspecies that are most in need of urgent attention were neglected.

## Introduction

The leopard (*Panthera pardus*) is a solitary, reclusive species of big cat. It is also the most widespread felid, extending across much of Africa, and Asia from the Middle East to the Pacific Ocean (Nowell & Jackson, 1996; Sunquist & Sunquist, 2002; Hunter, Henschel & Ray, 2013). Leopard habitat varies greatly. Found in tropical forests, grassland plains, deserts, and alpine areas (Nowell & Jackson, 1996), leopards can also persist near major towns, including Mumbai (Odden et al., 2014) and Johannesburg (Kuhn, 2014). The leopard has the broadest diet of larger obligate carnivores (Hayward et al., 2006). Their behavioral plasticity allows them to persist in areas where other big cats have been extirpated or severely isolated (Athreya et al., 2013; Athreya et al., 2014). This adaptability does not necessarily inure the species against all levels of threat, however.

The leopard is declining across its range similar to other large carnivores (Ripple et al., 2014). The key threats are all ongoing. They include habitat loss and fragmentation, prey depletion, conflict with people, unsustainable trophy hunting, poaching for body parts, and indiscriminate killing (Spalton et al., 2006; Breitenmoser et al., 2007; Datta, Anand & Naniwadekar, 2008; Kissui, 2008; Packer et al., 2010; Athreya et al., 2011; Raza et al., 2012; Swanepoel et al., 2015).

The International Union for the Conservation of Nature (IUCN) classifies the leopard as Vulnerable (Stein et al., in press) and recognizes nine subspecies (Miththapala, Seidensticker & O’Brien, 1996; Uphyrkina et al., 2001). Three subspecies (Amur, *P. p. orientalis,* Arabian, *P. p. nimr,* and Javan, *P. p. melas*) are classified as Critically Endangered (Ario, Sunarto & Sanderson, 2008; Jackson & Nowell, 2008; Mallon, Breitenmoser & Ahmad Khan, 2008) while two are Endangered (Persian, *P. p. saxicolor* and Sri Lankan*, P. p. kotyia*) (Khorozyan, 2008; Kittle & Watson, 2008). Recent papers recommend uplisting two other subspecies, the north Chinese (*P. p. japonensis*; Laguardia et al., 2015), and Indochinese leopard (*P. p. delacouri*; Rostro-García et al., 2016) from Near Threatened to Critically Endangered and Endangered respectively. The remaining two leopard subspecies, African (*P. p. pardus*) and Indian (*P. p. fusca*), are both Near Threatened (Henschel et al., 2008). Knowledge on leopard distribution is improving although detailed population estimates are still lacking (Stein & Hayssen, 2013). Earlier Africa-wide assessments of population size (Myers, 1976; Eaton, 1977; Martin & De Meulenaer, 1988; Shoemaker, 1993) employed questionable population models based on scant field data and were widely criticized as being unrealistic (Hamilton, 1981; Jackson, 1989; Norton, 1990; Bailey, 1993). Lack of empirical field data on distribution status and population size has prevented a range-wide population estimate, although researchers have guessed or estimated population size for all subspecies in IUCN assessments and recent papers (Laguardia et al., 2015; Rostro-García et al., 2016; Stein et al., in press). At the local scale, estimates of leopard population densities vary 300-fold from 0.1 individuals/100 km^2^ in the Ghanzi region of Botswana (Boast & Houser, 2012), to 30.9/100 km^2^ in Sariska Tiger Reserve, India (Edgaonkar, 2008). The variation in leopard densities is at least partially attributable to habitat productivity (Macdonald & Loveridge, 2010). Such a broad spread makes reliably estimating population numbers from known geographic ranges particularly difficult.

Robust estimates of distribution, population size, and threat require greater levels of research. Although they are essential in providing ecological insight (Durant et al., 2007), there is almost a complete absence of long term data on leopard populations (but see Phinda Private Game Reserve, South Africa; Balme, Slotow & Hunter, 2009). While the leopard generates considerable interest among researchers and conservationists, some regions and subspecies are studied far more intensively than others. This spatial bias is not unusual (e.g. birds (de Lima, Bird & Barlow, 2011), amphibians (Brito, 2008) or conservation biology in general (Fazey, Fischer & Lindenmayer, 2005)), but more comprehensive research efforts should be prioritized.

To create a more precise distribution of the leopard, we reviewed over 1,300 sources that report its presence and absence. From these data, we map the species across Africa and Asia, delineating its historic distribution as well as areas where the species is confirmed present, is possibly present, is possibly extinct, or is almost certainly extinct. We then measure how much range remains and how much has been lost. Furthermore, we investigate habitat patch metrics that may affect the population viability of fragments of leopard habitat and, in turn, the subspecies itself. Finally, we assess the different levels of research effort across subspecies and consider their implications for conservation.

## Materials and Methods

To create historic and current distribution maps for the leopard, we collected both peer-reviewed and grey literature primarily from the IUCN/Species Survival Commission (SSC) Cat Specialist Group library, but also from Internet search engines. We contacted over 75 species or area experts for information. We translated these data into geographic areas, mapped them in terms of historic and current distribution, and evaluated them based on the date and quality of the observation.

For this purpose, we define historic as approximately the year 1750. This is before the start of the Industrial Revolution, the colonial era in Africa, and the spread of firearms and human-induced land use changes became increasingly prevalent and caused significant changes in faunal communities (Morrison et al., 2007). It is possible leopard distribution changed prior to 1750, but it is more likely that major change came after this date. We base historic range on geo-referenced locations, generic geographic descriptions of leopard occurrence, and occasionally on indirect parameters like suitable habitat, natural barriers, terrain, climate, and natural vegetation cover. We also used terrestrial ecoregions (Olson et al., 2001) to delineate range boundaries between known geographic features.

To establish current distribution, newest and highest quality evidence, such as unambiguous photographs, genetic records or dead animals, were given more weight than data we could not confirm or which were more prone to error, such as tourist records. Hard facts with verified and unchallenged species observations were categorized differently from more subjective field evidence. Field evidence was given extra credibility if confirmed by an expert. Absence data were just as valuable as presence data and helped to delineate patch boundaries. In some cases, presence data may have been old, unverifiable, or from a transient individual, and hence could be in a region not marked as extant. We defined confirmed extant range as records not older than 3 leopard generations (21 years; Balme et al., 2013), based on the IUCN mapping standards. Some existing local range maps were incorporated into the assessment. We also used expert opinion, land cover, biogeographic data from other species, and other generic information from scientific and grey literature to refine the distribution boundaries, particularly in areas with older or fewer records. We also developed country profiles detailing historic and recent descriptions of leopard presence for all leopard range countries (Supplemental Document 1). Collection of evidence ended in January 2016.

These data were assembled to create historic and current range maps for the forthcoming IUCN Red List update to the leopard (Stein et al., in press). We used these layers to derive distribution and patch metrics. Using IUCN Red List guidelines, we categorized leopard range into extant, possibly extant (we use the term “possibly present”), possibly extinct, and extinct. Extant areas are regions where the leopard is confirmed or thought very likely to occur based on high-quality records not older than 21 years. Possibly present areas are regions where the leopard may possibly occur but recent records are lacking. The possible occurrence can be from expert opinion, unconfirmed records, or from records older than 21 years. Possibly extinct areas are regions where the leopard used to occur but there are no confirmed records in the last 21 years and they are unlikely still present due to habitat loss or other threats. Extinct areas are regions previously known or very likely to support the leopard but where searches have failed to produce records from the last 21 years and the intensity of threats could plausibly have extirpated the species.

Following Uphyrkina et al. (2001), we recognize nine leopard subspecies. Subspecies boundaries were slightly modified from Stein & Hayssen (2013). We shifted the boundaries to match nearby major geographical features (such as the Suez Canal in Egypt separating *P. p. pardus* from *nimr*, the Indus River in Pakistan separating *fusca* and *saxicolor*, the Irrawady River in Myanmar separating *fusca* and *delacouri*, and the Pearl River system in China separating *japonensis* and *delacouri*), or to follow political boundaries (the northern border of Israel and Jordan used to separate *P. p. nimr* from *saxicolor*).

We compiled the species’ distribution using QGIS 2.10.1-Pisa (Quantum GIS Development Team, 2015) and projected it using World Cylindrical equal area. Subsequent analyses were performed in ArcGIS 10.2 (Release 10.2.1; ESRI, Redlands, CA). We measure range loss as a percentage and current extent in km^2^. We provide two estimates of range loss. We calculate the upper and lower values for range loss by dividing confirmed extant range by total historic range, and the amount of extant, possibly present, and possibly extinct range by total historic range. Uncertain range expressed as a percentage of total historic range is the sum of possibly present and possibly extinct range divided by total historic range.

We also investigated the spatial configuration of habitat patches with FRAGSTATS v. 4.2 (McGarigal & Ene, 2013). We calculated three patch metrics for each subspecies, Patch Size Index, Core Area Index, and the Proximity Index, as further measures of the potential viability of leopard habitat patches. Patch Size Index is a measure of the relative size, or dominance, of a single patch. It is the percentage of the total historic extent comprised by the largest patch. A larger value indicates greater dominance of a single patch. Core Area Index is the core area of a range category (e.g., extant) as a percentage of the total extent of that category. A larger Core Area Index indicates a greater amount of core habitat. We define core area as the habitat area more than 10 km inside the patch edge. We used a negative buffer of 10 km as this is approximately the radius of a leopard home range in areas with low productivity such as arid or mountainous parts of Africa (Sunquist & Sunquist, 2002). While home ranges can be substantially smaller in more productive and/or well-protected habitats (see Bailey, 1993), we followed this cautionary approach considering that much of remaining leopard habitat has reduced carrying capacity for the leopard due to habitat disturbance and the depletion of prey populations. The Proximity Index is a measure of both the degree of isolation and of fragmentation within a specified search radius. A larger value indicates greater proximity to nearby patches and/or more of the corresponding patch type within the search radius. The index was developed by Gustafson and Parker (1992 cited in McGarigal & Ene, 2013) and equals the sum of patch area divided by the squared distance between the focal patch and other patches whose edges are within the search radius. In this context, patch edge-to-edge distance is computed from cell center to cell center. We used a search radius of 200 km as this is approximately the farthest known straight-line dispersal distance of a leopard (Fattebert et al., 2013). This is simplified from the original index since we are interested in only one patch type, habitat vs. non-habitat.

We downloaded protected area coverage (World Database on Protected Areas (WDPA)) from Protected Planet in October 2015 (UNEP & IUCN, 2015). Protected areas are categorized into six different levels of protection, with the lowest numbers representing strictest protection. To calculate amount of protected range, we primarily calculated range within categories one to four but with some additional modifications. We used categories one to four as a way to eliminate areas with less effective protection (Morrison et al., 2007) and double-counting as, for example, some areas are both a national park and World Heritage Site. We included all protected areas that were identified as “national parks’ or “national reserves” as some did not have protection categories or were listed as five or six. We then eliminated all protected areas that were not designated (i.e., they were only proposed) and any that were identified as a marine protected area. Finally, we made changes to two countries, Iran and China, as they otherwise had unrealistically low levels of protection. We replaced the WDPA data in Iran with Iran’s Department of Environment (2012) protected area data. The national parks, protected areas, wildlife reserves, and non-hunting areas were given a protected category from two to four. For China, we changed all National Nature Reserves from category five to four as these are China’s strictest protected areas and their core zones do not allow human settlements or resource extraction (Xu & Melick, 2007; Xie, Gan & Yang, 2014).

Human population densities are from LandScan 2015 High Resolution Global Population Data Set copyrighted by UT-Battelle LLC and operator of Oak Ridge National Laboratory under Contract no. DE-AC05-000R22725 with the US Department of Energy (Bright et al., 2014). LandScan is the finest resolution global population data set available and is updated annually. Data represent the ambient (or daily average) population at a roughly 1 km resolution (30″ × 30″). We calculated mean human population densities per patch and per range category for each subspecies.

### Literature review

We compiled published scientific articles on leopards and compared them across subspecies. We identified and categorized peer-reviewed articles in the English language on leopard ecology and conservation published between January 2000 and September 2015. We used the search terms ‘leopard’ and ‘*Panthera pardus*,’ and reviewed the IUCN/SSC Cat Specialist Group library, Web of Science, and Google Scholar. Only if the title, abstract, or keywords contained the search terms and if the paper appeared in a peer-reviewed journal did we include the article. We further refined our search by eliminating a handful of publications that were not directly relevant to wild leopard ecology or conservation (e.g., the research was based on captive animals). The literature search was conducted independently from the process used to create the distribution maps.

Articles were categorized by primary content, geography (i.e., subspecies), and year, similar to Balme et al. (2014). We divided articles into three content categories: applied, fundamental, and documental. The categories were hierarchical with applied at the top and documental at the bottom, such that if an article contained any material relevant to the higher category, it was included in that category. Thus, an article labeled “applied” may contain fundamental or documental content, but utilized the content to a higher management or conservation end. We further subdivided each category. Applied content was broken down into studies informing management or conservation policy, conducting population surveys, or analyzing habitat suitability/connectivity. Articles classified as “fundamental” (or “basic” using the terminology of Balme et al. (2014)) focused on leopard ecology and behavior. Fundamental articles were subdivided into studies of demography, feeding ecology, intraspecific interactions, interspecific interactions, habitat use/selection, and other (including anatomy, physiology, movement). Documental articles only documented leopard or human-leopard conflicts at particular locations. The article could be primarily about another species or topic (while still meeting the search criteria) but noting that the leopard or human-leopard conflicts were present at the site. Finally, we identified those articles with population density estimates because these can represent a step towards effective management.

## Results

**Figure 1:**
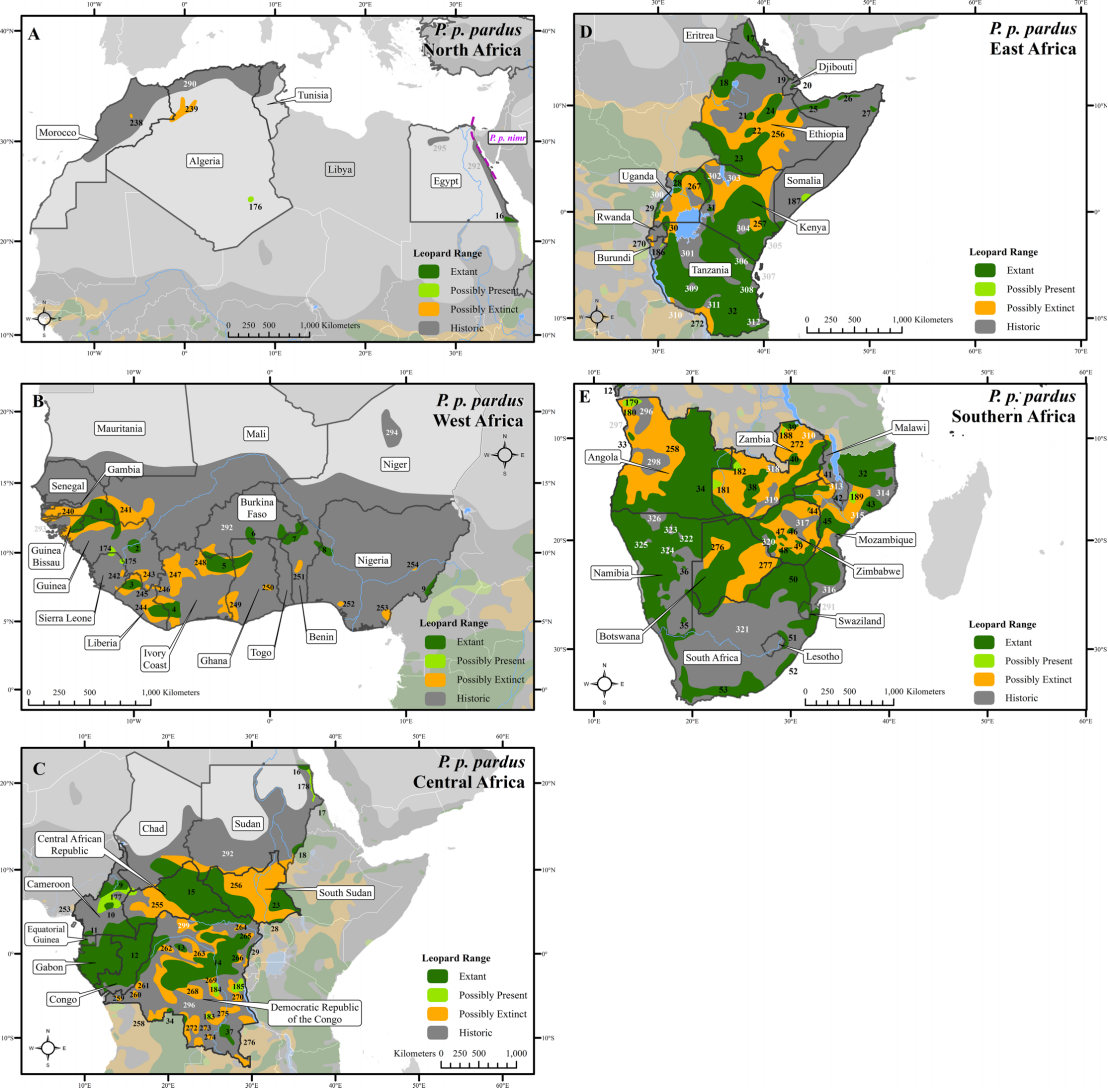
Leopard range across Africa. (A) North Africa, (B) West Africa, (C) Central Africa, (D) East Africa, (E) Southern Africa. Numbers in black refer to extant, possibly present, and possibly extinct habitat patch IDs while those in white refer to extinct patches.

**Figure 2:**
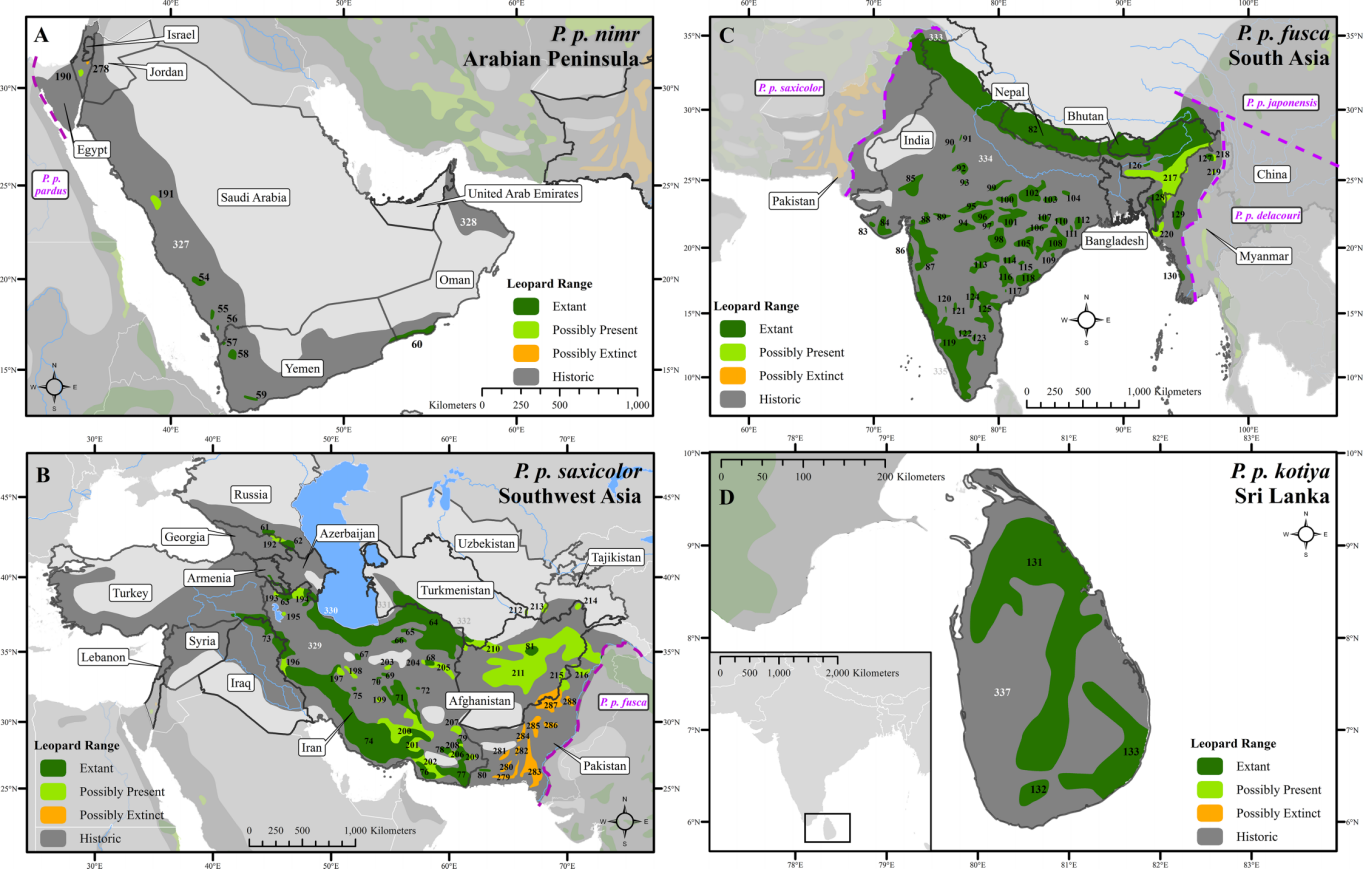
Leopard range and subspecies delineation across the Middle East and Asia. (A) Middle East, (B) Southwest Asia, (C) South Asia, (D) Sri Lanka. Numbers in black refer to extant, possibly present, and possibly extinct habitat patch IDs while those in white refer to extinct patches.

**Figure 3:**
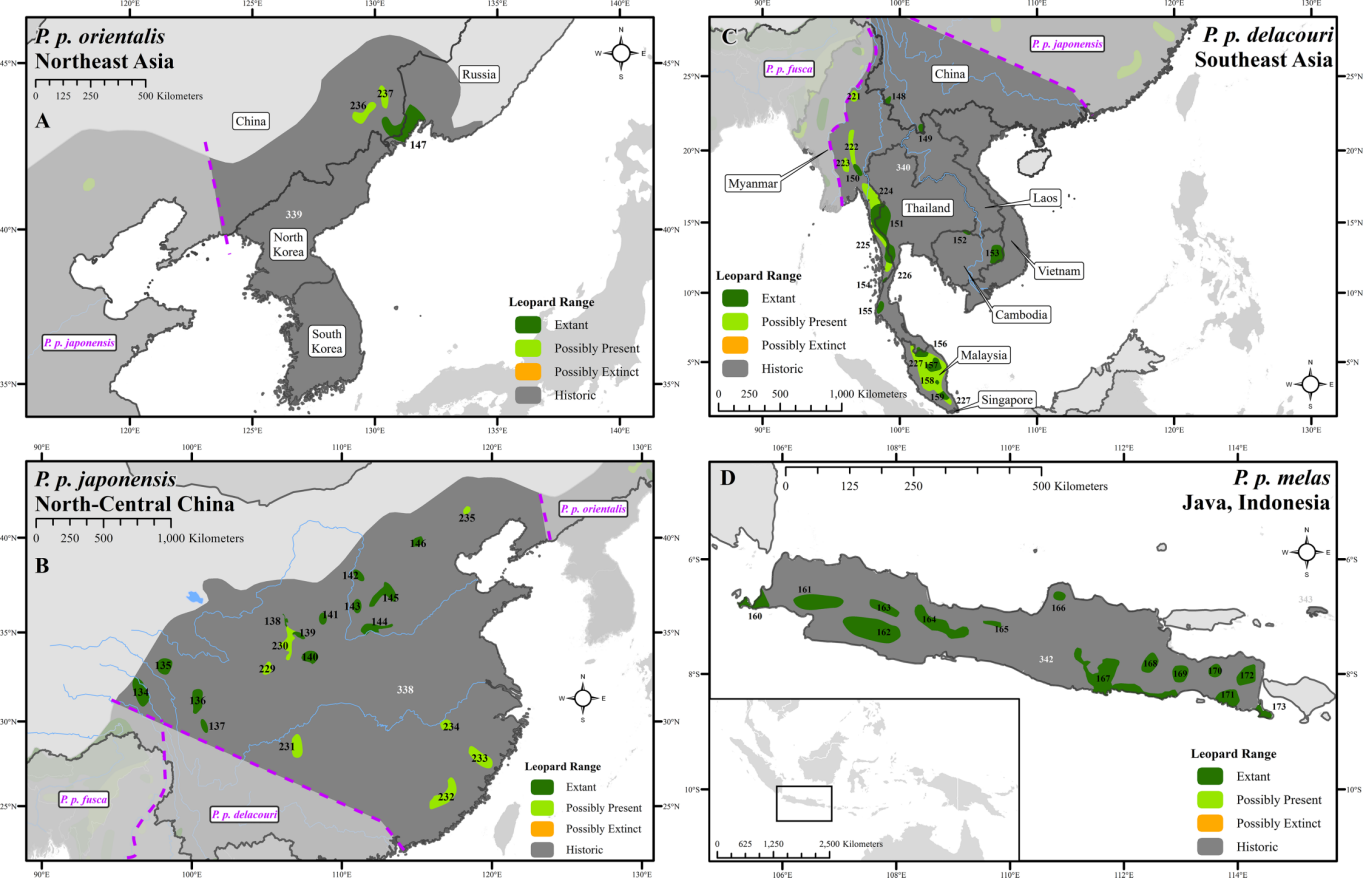
Leopard range and subspecies delineation across eastern Asia. (A) Far East, (B) China, (C) Southeast Asia, (D) Indonesia. Numbers in black refer to extant, possibly present, and possibly extinct habitat patch IDs while those in white refer to extinct patches.

We compiled 6,000 records at 2,500 locations from over 1,300 sources (Table S1). These showed that the leopard historically lived across nearly 35,000,000 km^2^ but is now confirmed present in only 25% of this area (Figs. 1–3 and S1–S4), in 173 extant patches covering ∼8,500,000 km^2^ (Tables 1 and S2). The leopard has suffered range loss of 63–75% (Table 2).

**Table 1:** Leopard distribution by range category.

Range categories	Area (km^2^)	% Of historical extent	# Of patches
Extant	8,510,500	25	173
Possibly present	738,000	2	64
Possibly extinct	3,528,700	10	52
Extinct	21,891,900	63	–
Grand total	34,669,100	100	–

**Table 2:** Range estimates for leopard subspecies. Values indicating greatest threat or loss are in bold. Subspecies are ranked from subspecies with least extant range to most.

Subspecies	Extant range km^2^ (% of total)	% Extant/historical	% Extant core/historical	% Range loss	% Uncertain remaining range	# Of extant countries (historical)*	% Protected extant range (cat. 1–4)
*Panthera pardus*	8,510,500	25	NA	63–75	10–15	62 (85)	17
*P. p. orientalis (CR)*	8,100 (0.1)	**2**	**1**	**97–98**	< 5	2 (4)	25
*P. p. nimr (CR)*	17,400 (0.2)	**2**	**1**	**98**	< 5	**3 (7)**	**9**
*P. p. melas (CR)*	20,600 (0.2)	16	3	84	< 5	1 (1)	17
*P. p. kotiya (EN)*	24,400 (0.3)	37	15	63	< 5	1 (1)	50
*P. p. japonensis*	68,000 (0.8)	**2**	**1**	**96–98**	< 5	1 (1)	18
*P. p. delacouri*	90,400 (1.1)	4	2	93–96	< 5	5 (8)	45
*P. p. saxicolor (EN)*	602,000 (7.1)	16	12	72–84	10–15	9 (14)	18
*P. p. fusca*	1,066,600 (12.5)	28	20	70–72	< 5	7 (7)	11
*P. p. pardus*	6,613,000 (77.7)	33	30	48–67	**15–20**	38 (47)	17

**Note:**

* Note that several countries are counted only once in the species total but are included in more than one subspecies (e.g., *P. p. orientalis* and *saxicolor* both include the Russian Federation). For the purpose of counting countries, we list some inclusions and exclusions. This is not meant to endorse any political statements about statehood. *P. p. delacouri* includes Singapore and does not count Hong Kong as a separate country; *P. p. saxicolor* does not count Nakhchivan, or the Nagorno-Karabakh Republic; Lebanon and Syria are included with *saxicolor* while Israel and Jordan are included with *nimr*; *P. p. pardus* does not count Spain, Western Sahara, or Zanzibar. For more information see Table S4.

Range loss varied between subspecies and regions. For Africa, range loss was 48–67%, while for Asia (all non-*P. p. pardus* subspecies) range loss was 83–87% (Table S3). Extant range is unequally split between subspecies with one (*P. p. pardus*) comprising 78% of range, while five others each account for less than 1% (Table 2). Within Africa, range loss varied greatly by region (Table S3). Estimated leopard range loss was up to 99% in North Africa, and 86–95% in West Africa, but only 28–51% in Southern Africa. Four subspecies have lost more than 90% of their historic range and six are spread across less than 100,000 km^2^. Three subspecies (*P. p. orientalis, nimr, japonensis*) were confirmed to reside in 2% or less of their historic extent while *delacouri* resided in only 4% of its historic extent. *P. p. kotiya* was confirmed extant across the greatest percentage of its historic extent at 37%. Five subspecies (*P. p. orientalis, nimr, japonensis, melas, delacouri*) have 5% or less of core area remaining.

The leopard was confirmed extant in 73% (62 of 85) of its historic range countries (Tables 2 and S4). *P. p. nimr* is extirpated from the greatest percentage of its historic range countries (57%, n = 4), but *P. p. pardus* has been extirpated from the greatest number of countries (n = 9).

Level of protection varied widely across subspecies despite 17% of the overall extant range protected (Table 2; see Table S5 for percentages in each country). *P. p. kotiya* and *delacouri* had the greatest remaining percentages of extant range within protected areas, 50 and 45% respectively. *P. p. nimr* and *fusca* had the least amount of extant range, with only 9% and 11% respectively, in protected areas.

The number of extant patches varied from one for *P. p. orientalis* to 53 for *pardus* (Table 3). Three subspecies had fewer than 10 extant habitat patches. Median patch size varied by more than a factor of 10, from only 961 km^2^ (*P. p. melas*) to 10,972 km^2^ (*pardus*). The Patch Size Index was highest for *P. p. kotiya* indicating the greatest proportion of remaining habitat in a single patch (Table 3). The Core Area Index was smallest for *P. p. melas* and *nimr* indicating the greatest proportion of remaining habitat within 10 km of the edge. *P. p. nimr, delacouri,* and *japonensis* all had very small Proximity Indices indicating the greatest isolation and fragmentation of remaining extant habitat patches. *P. p. pardus* had the highest Core Area Index and Proximity Index.

**Table 3:** Extant range patch metrics per subspecies. Values indicating greatest threat are in bold. Subspecies are ranked from subspecies with least extant range to most.

Subspecies	Extant range (km^2^)	# Of extant patches	Median patch size (km^2^)	Largest patch index	Core area index	Proximity index
*P. p. orientalis (CR)*	8,100	**1**	8,199	1.9	44.1	0
*P. p. nimr (CR)*	17,400	7	1,506	0.9	23.1	**0.4**
*P. p. melas (CR)*	20,600	14	**961**	3.8	**16.5**	7.5
*P. p. kotiya (EN)*	24,400	3	5,259	**28.0**	38.7	137.6
*P. p. japonensis*	68,000	13	3,376	0.4	51.3	1.6
*P. p. delacouri*	90,400	12	3,698	1.7	62.8	1.0
*P. p. saxicolor (EN)*	602,000	21	3,448	6.2	72.6	89.6
*P. p. fusca*	1,066,600	49	5,514	12.6	74.0	197.4
*P. p. pardus*	6,613,000	53	10,972	9.7	88.4	6727.5

**Figure 4:**
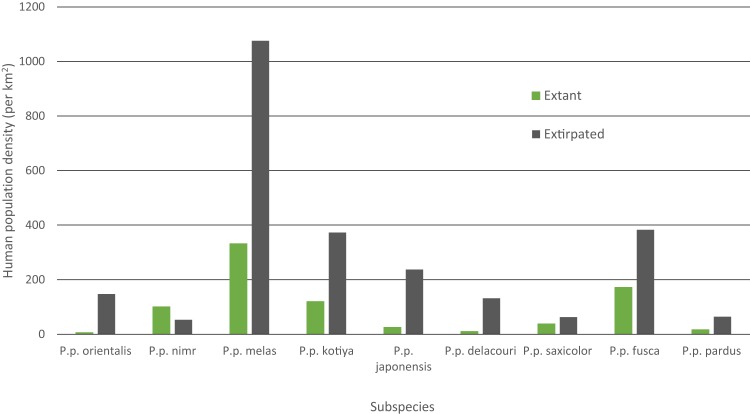
Mean HPD of extant and extirpated range per subspecies.

Mean human population density varied widely across subspecies and was generally lower for extant than extinct range (Fig. 4). Human density in extant range was highest for *P. p. melas* at 332 people/km^2^ and nearly double that of the next highest, *fusca* at 172 people/km^2^. *P. p. nimr* was the only subspecies where human density was higher in extant range than extinct range (101 and 53 people/km^2^ respectively). *P. p. orientalis* had the lowest population density in extant range (6 people/km^2^).

### Literature review

**Figure 5:**
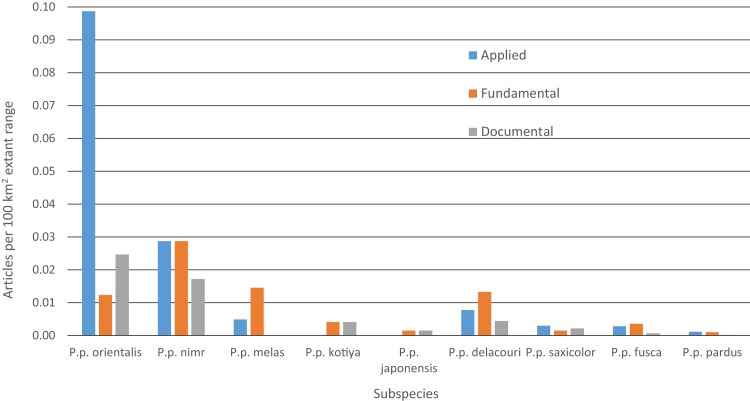
Number of leopard articles per subspecies per article type divided by extant range.

Between 2000 and 2015, we found 330 peer-reviewed published articles regarding leopard ecology and conservation (Tables 4 and S6). The number of articles published annually has increased steadily since 2000, rising from < 10 to ∼35 (Fig. S5). Some 46% of articles dealt with *P. p. pardus* and another 23% dealt with *P. p. fusca*. Yet, after dividing by the amount of extant range for each subspecies, most articles focused on *P. p. orientalis* (Fig. 5). Fewer than five articles were specifically on *P. p. japonensis, kotiya,* or *melas*. Overall, 45% of articles were applied, 42% fundamental and 13% documental. Of the subspecies with more than five articles, the highest percentage of applied articles for a subspecies was 73% for *P. p. orientalis. P. p. delacouri* had the fewest applied articles, at 30%. Of the three subspecies listed as Critically Endangered, *P. p. melas* only had one applied article, and *nimr* had five compared to eight for *orientalis*. Of the two subspecies listed as Endangered, *P. p. kotiya* had zero applied articles while *saxicolor* had 18. *P. p. saxicolor* and *nimr* have the highest percentages of documental articles at 23%. We could not easily assign a remaining seven articles to a single subspecies but they were still categorized by content.

**Table 4:** Number of published leopard articles by subspecies and category since 2000.

Subspecies	Total # articles per subspecies (% of total)	# Applied (% of subspp. total)	# Fundamental (% of subspp. total)	# Documental (% of subspp. total)	# With population density estimates
*P. p. orientalis (CR)*	11 (3%)	8 (73%)	1 (9%)	2 (18%)	3
*P. p. nimr (CR)*	13 (4%)	5 (38.5%)	5 (38.5%)	3 (23.1%)	2
*P. p. melas (CR)*	4 (1%)	1 (25%)	3 (75%)	0 (0%)	0
*P. p. kotiya (EN)*	2 (1%)	0 (0%)	1 (50%)	1 (50%)	0
*P. p. japonensis*	2 (1%)	0 (0%)	1 (50%)	1 (50%)	0
*P. p. delacouri*	23 (7%)	7 (30%)	12 (52%)	4 (17%)	4
*P. p. saxicolor (EN)*	40 (12%)	18 (45%)	9 (22%)	13 (23%)	3
*P. p. fusca*	75 (23%)	30 (40%)	38 (50%)	7 (9%)	10
*P. p. pardus*	153 (46%)	75 (49%)	67 (44%)	11 (7%)	22
**Grand Total**	330	148 (45%)	140 (42%)	42 (13%)	44

Lastly, we found 44 papers with detailed population density estimates (Table S7). The number of peer-reviewed population density estimates varied widely by subspecies, with none for *P. p. melas*, *kotiya* or *japonensis*, whereas there were 22 for *pardus*.

## Discussion

With our first-ever comprehensive delineation of the historic and current distribution of the leopard, we found the leopard was confirmed extant in only 25% of historic range, although, presence was uncertain in an additional 12% of historic range. Overall range loss was 63–75%, yet, some subspecies and regional populations were even more threatened and have suffered range loss greater than 94% (*P. p. orientalis, nimr, japonensis*, and North and West African regional populations of *P. p. pardus*). We also found that research effort concentrates on those subspecies with the greatest amount of remaining range (*P. p. pardus* and *fusca*), rather than on the most threatened subspecies. When controlling for total range extent however, the more threatened subspecies did proportionally receive higher research effort, which is encouraging.

While the leopard can persist in highly modified and densely-populated landscapes (Athreya et al., 2013; Athreya et al., 2014; Kuhn, 2014), this has not prevented widespread and significant range decline. Prior efforts to establish leopard range loss (e.g., Ray, Hunter & Zigouris, 2005; Morrison et al., 2007) investigated tens of species concurrently and suffered from a paucity of presence and absence records compared to our study, and likely underestimated range loss (36.6% for Africa, 35% range-wide, respectively). Contrary to the pervasive impression of the leopard as being one of the most widespread, adaptable and resilient carnivores, our calculated range loss of 63–75% exceeds the average range loss documented for the world’s largest carnivores (53% for 17 species; Ripple et al., 2014).

There are important differences between Africa and Asia. Historic leopard range in Africa was ∼20,000,000 km^2^, whereas in Asia it was ∼15,000,000 km^2^. Up to ∼13,100,000 km^2^ of leopard range (when including uncertain range) has been lost from both continents leading to a higher level of range loss in Asia (83–87%) than for Africa (48–67%).

At a local scale, some subspecies and regional populations have declined to critically low levels. Three subspecies have each declined to occupy only 2% of their historic range, *P. p. orientalis, nimr,* and *japonensis. P. p. delacouri* has declined to only 4% of its range and yet neither it nor *japonensis* are classified as Endangered by the IUCN. Our analysis provides further support to the recommendations of Laguardia et al. (2015) and Rostro-García et al. (2016) to uplist the threat status of *P. p. japonensis* and *delacouri* to Critically Endangered and Endangered respectively, based on range declines and small population sizes. Thus, overall, four of nine subspecies have declined to less than 5% of their historic range while one subspecies, *P. p. pardus,* comprises 78% of remaining extant global leopard range. Yet, this number is misleadingly reassuring. Much of *P. p. pardus’* range is concentrated in eastern, central and southern Africa while western and northern populations are highly threatened (Table S3). Durant et al. (2014) estimated leopard range across the Sahara (defined as those regions with < 250 mm of rain) to have declined from its historical extent by 97%, and our analysis confirms that decline. However, range contraction across the entire North African region is even greater at 99%. Leopard range has also declined substantially in West Africa with an estimated loss of 95%. Thus, even for this relatively widespread subspecies, there is still substantial cause for concern across large portions of its range.

More detailed genetic analyses are required to confirm subspecies’ status and distribution (but see Farhadinia et al., 2015a). While shifting the location of the boundary does alter range loss per subspecies, the exact interchange may be impossible to know. It is likely that the mainland Asian subspecies mixed significantly and were without hard boundaries, as Uphyrkina et al. (2002) suggested for *P. p. orientalis* and *japonensis*. Indeed, the rivers we selected as boundaries are not known as effective biological limits for leopards, but rivers can pose partial barriers to large carnivores (Cozzi et al., 2013), and for our purposes, they represented possible and geographically relevant boundaries close to the subspecies boundaries previously identified (Stein & Hayssen, 2013). More geographically comprehensive and detailed genetic analyses, including within Africa, are required as prior delineations of subspecies suffered from small sample sizes, captive born individuals, and ignored entire sub-regions e.g., North Africa, Southeast Asia (Uphyrkina et al., 2001). Alternative subspecies delineations have the potential to shift conservation priorities such as recent proposed changes for the lion (*Panthera leo*; Barnett et al., 2014) and tiger (*Panthera tigris*; Wilting et al., 2015).

The amount of extant range reported above may be conservative as several subspecies also have large areas of uncertain range (identified as possibly present or possibly extinct). *P. p. saxicolor* and *pardus* have the greatest amount of uncertain range. Importantly, the extant range of *P. p. delacouri* and *japonensis* could approximately double given new sightings in uncertain regions. Uncertain areas should be priorities for systematic field surveys. Yet, some range will likely remain unknown as it is in politically unstable regions (e.g., Somalia, South Sudan etc.). It is possible that we overlooked some data and areas were missed, such as, for example, the possibility of leopard presence in eastern Iran (M. Farhadinia, 2015, unpublished data), or in unprotected areas of India (V. Athreya, 2015, personal communication). Moreover, for areas of potential range where there was little historic information (e.g., areas of the Sahel), we assumed that if the original land cover likely provided suitable habitat and prey base, then it should be included as historic range. Thus, despite collecting over 1,300 sources and communicating with 75 experts, it is possible that both current and historic range may contain some inaccuracies, although it is unlikely that these are substantial. As new data become available, historic and current range should be further refined.

It is not just the amount of extant range that is important the spatial arrangement, shape and distribution of habitat patches strongly influence ecological processes (McGarigal & Marks, 1995). In general, more and larger patches are better for species persistence, as is more core area and greater connectivity between patches (MacArthur & Wilson, 1967; Pimm, Jones & Diamond, 1988). With this in mind, we demonstrate that *P. p. orientalis* is threatened not only because it occupies the least amount of extant range, but also because this range consists of only a single patch. A population restricted to a single patch is more susceptible to extinction through stochastic events than a metapopulation of multiple patches (Hanski & Ovaskainen, 2000); although, it is better than if the same amount of habitat was widely dispersed among multiple separate patches (MacArthur & Wilson, 1967). Similarly, *P. p. kotiya* has the highest Patch Size Index indicating that despite occurring in more than one patch, the Sri Lankan leopard is greatly dependent on a single patch. *P. p. melas* on the other hand has 14 patches with a median patch size of only 961 km^2^. With no peer-reviewed density estimates from Indonesia (Table S7), the number of leopards capable of residing within these relatively small patches is unknown. Increasing the threat to these patches, *P. p. melas* also has the smallest proportion of core area. While an increasing number of habitat patches is good, their value may be limited if they are not close enough for effective dispersal. *P. p. nimr*, *delacouri*, and *japonensis* all have very low Proximity Index values suggesting low connectivity between patches. Therefore, despite having between seven and 13 extant patches, the patches for these subspecies are largely disconnected from each other. Establishing and maintaining more ‘stepping-stone’ subpopulations and habitat corridors would improve connectivity (MacArthur & Wilson, 1967) and increase the genetic viability of each patch, and should be considered in conservation efforts for these subspecies.

The patch metrics presented here should be interpreted with caution because greater search effort is likely to bias results. In regions with better survey effort, more detailed patch boundaries can be drawn and hence could lead to splitting rather than lumping of patches, e.g., less detailed patch boundaries follow from more coarse scale data. This problem may affect all aspects of the analysis as it could result in reduced extant range, increased number of patches, reduced core area, reduced connectivity, etc. This bias will have affected some subspecies more than others as some were subjected to greater search effort. For instance, the tremendous size of extant range in Africa makes it difficult to survey effectively and despite occupying 57% of historic range, only 36% of records were from *P. p. pardus* (Tables S1 and S8).

Some subspecies populations are confined to a single country, which may increase their vulnerability to extinction. Individual countries vary in the resources they provide to conservation, and may be politically unstable, or more or less prone to natural and human disasters. Three subspecies are endemic to a country (*P. p. kotiya* to Sri Lanka, *melas* to Indonesia, and *japonensis* to China); while a further two subspecies (*P. p. fusca* and *saxicolor*) have > 75% of their extant range within one country (India and Iran respectively; Table S5). Thus, conservation action for leopards in these countries could help safeguard five of the nine subspecies.

Human population density can be a predictor for local carnivore extinctions (Woodroffe, 2000). Although Woodroffe (2000) found a significant relationship between high human densities and leopard extinction, the critical threshold for the leopard in Kenya was 958 people/km^2^, more than 10 times greater than any other carnivore investigated. This potentially highlights the adaptability of the leopard even among carnivores. Although not directly comparable, in our analyses, the overall mean population density of all extirpated patches was 142 people/km^2^, and the mean population density for extirpated patches per subspecies varied between 53 and 1,076 people/km^2^ (*P. p. nimr* and *melas* respectively). *P. p. melas* has the highest human density for its extant range, nearly double that of the next closest subspecies (332 to 172 people/km^2^ for *fusca*), and more than 50 times the density for the lowest subspecies (6 people/km^2^ for *pardus*). Nevertheless, *P. p. nimr* stands out as the only subspecies for which human population density in the extant range is higher than in the extinct range, with several individual patches containing relatively high human densities (Table S2). This is likely an artifact of the coarse scale of the distribution combined with very low human densities in the surrounding desert areas where the leopard has gone extinct. Populations of leopard and their prey in the deserts would have been limited by low and variable rainfall, making them particularly vulnerable to modest levels of human pressure. Alternatively, this could be a case in which both leopards and humans are reliant on limited, shared resources such as water (Khorozyan et al., 2014) and/or in which the leopard is heavily reliant on domestic or feral animals, possibly due to low levels of wild prey biomass, similar to a case in Pakistan (Shehzad et al., 2015). Overall, however, the observed wide variability suggests that human population density per se is not a threat and leopards can persist alongside high human density given proper management policy, local tolerance, and suitable cover and prey (Linnell, Swenson & Andersen, 2001; Chapron et al., 2014; Athreya et al., 2015).

### Literature review

Research effort is skewed towards the subspecies with the most remaining range with 46% of articles focused on *P. p. pardus* and 23% on *fusca*. Similarly, Balme et al. (2014) found ∼50% of articles focused on *P. p. pardus. P. p. japonensis, kotiya,* and *melas* each have fewer than five peer-reviewed articles (combined equaling 2.5% of all articles). However, we acknowledge that our literature review missed articles in languages other than English, particularly for *P. p. japonensis* (Laguardia et al., 2015). Yet, for some of the most endangered subspecies, this paucity of research may also represent a lack of conservation attention and focus. Given that threats to biodiversity are often at a local scale (Gardner et al., 2009), successful conservation practices often depend on evidence-based research at specific sites (Sutherland et al., 2004). While increased research does not necessarily equate with more effective conservation, research is often necessary to identify the most effective interventions at addressing threats and reversing decline. Consequently, those subspecies with the least amount of published research may lack the necessary analyses to implement and evaluate effective conservation interventions. In summary, six of the nine leopard subspecies persist across either less than 5% of their historic range, or are extant across less than 100,000 km^2^ and these subspecies receive much less total research effort than the remaining three subspecies that are more widely distributed.

Yet, encouragingly, there are more research articles for the three Critically Endangered subspecies on a per area basis. Two of them, *P. p. orientalis* and *nimr*, also have higher percentages of applied research articles than either fundamental or documental. A higher percentage of applied research articles should be expected for subspecies that are at greater risk of extinction as this represents more articles focused on informing policy, guiding management, or tracking population trends (see Table S9 for this further breakdown). Indeed, *P. p. orientalis* has the highest percentage of applied articles of any subspecies, possibly representing the long history of conservation and research focus on the subspecies (see Table S10 for a list of some conservation organizations with leopard-focused projects).

### Major threats

Leopards face multiple threats across their range including habitat loss and fragmentation (Nowell & Jackson, 1996), conflict with livestock or game keepers (Ogada et al., 2003; Kissui, 2008; Swanepoel et al., 2015), loss of prey (Datta, Anand & Naniwadekar, 2008; Qi et al., 2015), killing for the illegal trade in skins and parts (Oswell, 2010; Raza et al., 2012), and in some areas, unsustainable legal trophy hunting (Packer et al., 2010). The first four of these threats exert pressure on leopard populations across their range; however, each threat differs in the pressure it exerts on the different subspecies. We address each of these five main threats in turn.

Habitat loss and fragmentation is a primary driver of biodiversity loss (Fahrig, 2003) and contributor to leopard decline (Nowell & Jackson, 1996). Across much of leopard range, land has been converted to agriculture to produce crops for a growing human population. This process reduces the quality of habitat, fragments the remaining habitat, and threatens local capacity to support viable leopard populations. This is particularly the case in Southeast Asia where habitat loss has been a dominant driver of biodiversity loss (Sodhi et al., 2004) and leopard range contraction (Nowell & Jackson, 1996; Laguardia et al., 2015; Rostro-García et al., 2016). This threat will also likely be increasingly significant for leopards in Africa over the coming decades due to growing economies, changing land tenures, and increasing human populations (Ahlers et al., 2014; United Nations, 2015).

Protected area networks are cornerstones of conservation effort and could safeguard leopard populations from pressures due to land use change. However, the overlap between leopard range and protected areas is highly variable between subspecies. *P. p. nimr* has the smallest percentage of extant range protected at only 9% while *kotiya* has 50% of extant range protected. Researchers have previously called for increasing protected area coverage in the Middle East for leopards (Al-Johany, 2007). Increasing well-managed protected area coverage in other regions has demonstrated that this can contribute to leopard population recovery (Askerov et al., 2015). However, we acknowledge that the percentage of protected range may be less appropriate for wide ranging and widespread species such as the leopard whose home ranges can be larger than some protected areas, and where a greater percentage of protected range may actually reflect a diminished extant range. Also, these analyses make use of the WDPA, which we acknowledge contains inaccuracies in protected area boundaries and classification; however, it provides the most extensive data on protected areas available at present. Ultimately, protected areas are important components of conservation strategies, particularly as range contracts, but local laws governing poaching, problem animal control, or retaliatory killing may be more important than a protected area boundary in some cases (Athreya et al., 2013).

Intense persecution of leopards, particularly retribution for real and perceived livestock loss (Shoemaker, 1993; Ray, Hunter & Zigouris, 2005), is another widespread threat to the species. In some areas, such as India, leopards are also feared for their attacks on people (Singh, 2005). Among a range of large carnivores, leopards were reported to account for the greatest percentage of livestock depredation at some sites (Sangay & Vernes, 2008; Dar et al., 2009; Karanth et al., 2013; Thorn et al., 2013). But, even when losses due to leopard predation are few, predation can represent a significant challenge particularly for vulnerable and marginalized communities who may not have access to alternative livelihoods (Dickman, 2010). In addition, regardless whether livestock off-take due to leopard is low, when there is depredation by other large carnivores, leopards may become casualties, particularly if poisoning is used as leopards will scavenge or return to their kills (Myers, 1976; Zafar-Ul Islam et al., 2014). When native prey is scarce, leopard may even come to depend on livestock depredation, such as has been observed in Pakistan (Shehzad et al., 2015). Leopards may also come into conflict with game farmers, who do not tolerate leopard on their land because of predation of valuable game animals (Lindsey, Romañach & Davies-Mostert, 2009).

Loss of prey is the third key driver of leopard range contraction. In intact rainforest in Africa, wild meat off-take removes prey for leopard and may drive localized extinctions (Henschel et al., 2011; Stein et al., in press). Wild meat harvests continue to increase with the expansion of road networks, and mining and timber extraction. Loss of prey is also a key pressure on the species across its range in drylands (Lindsey et al., 2013; Durant et al., 2015) and is likely an important driver in range contraction across the savannahs of Africa, the Sahara and Middle East. Hunting of ungulates for food or trophies has significantly depressed wild prey numbers throughout the Caucasus as well (Mallon, Weinberg & Kopaliani, 2007). In many areas, leopard may also be incidental casualties of wire snares or gin traps used to capture game.

The fourth threat, illegal trade in leopard skins and parts, was and continues to be a major threat in many parts of their range in Africa (Myers, 1976; Hamilton, 1981; Ray, Hunter & Zigouris, 2005) and Asia (Oswell, 2010; Raza et al., 2012; Nowell, 2014a). Skins and canines are still traded widely and openly in villages and cities in some African countries where parts are used in traditional rituals (Stein et al., in press). A recent study of illegal trade in cheetah (*Acinonyx jubatus*) uncovered a widespread demand for spotted cat skins, particularly in Nigeria, Central Africa, and Sudan, but also to feed the market for skins in Southeast Asia (Nowell, 2014b). In some markets, leopards are the most commonly traded big cat species (Oswell, 2010), with shockingly high volumes (18,000 leopard claws seized in one operation in Khaga, Uttar Pradesh, India; Raza et al., 2012).

The final primary threat, unsustainable legal trophy hunting, is localized to those countries that allow leopard hunting, and where hunting regulations are not sufficient to ensure off-take is sustainable. However, it is possible, current levels of off-take are not set sustainably in any country that allows leopard hunting (Balme et al., 2010). Balme et al. (2010) argued that no country has comprehensive and detailed leopard population information combined with an understanding of the impact of hunting on leopards within a proper regulatory framework. Despite the popularity and importance of the leopard to the trophy hunting industry, there is scant research on the impacts of hunting (Balme et al., 2010; Lindsey et al., 2011). However, there is evidence that trophy hunting can negatively impact leopard populations, particularly as hunting can disrupt the social structure and spatial dynamics of leopards and contribute to infanticide (Balme, Slotow & Hunter, 2009; Balme, Hunter & Braczkowski, 2012; Packer et al., 2009; Packer et al., 2010). Yet, it is difficult to disentangle the impacts of trophy hunting from those of illegal killing and problem animal control which likely result in a greater absolute number of individuals killed (Balme et al., 2010; Jorge, 2012). In light of this argument, South Africa has recently imposed a yearlong leopard hunting ban for 2016 due to a lack of population data (Associated Foreign Press, 2016).

More research is needed to better understand the major threats facing the leopard in different parts of its range, and more funding and effort are needed to implement regionally-specific conservation programs addressing major threats. Importantly, threats and populations often cross national boundaries, reinforcing the importance of international cooperation and transboundary conservation programs (Breitenmoser et al., 2007; Breitenmoser et al., 2010; Fattebert et al., 2013; Farhadinia et al., 2015b; Jiang et al., 2015).

## Conclusions

We delineate historic and current range of the leopard and find them extant in only 25% of historic range, far less than the average terrestrial carnivore (Ripple et al., 2014). Additionally, a range-wide estimate obscures important differences in range contraction between subspecies and regions. Small patch size, few remaining patches, and isolation further threaten those subspecies with the least amount of remaining range (*P. p. orientalis, nimr, melas, kotiya,* and *japonensis*). Thus, while the leopard can persist in certain human-dominated landscapes provided there is cover and prey, favorable governmental policy, and a certain degree of tolerance (Athreya et al., 2013; Athreya et al., 2015), the leopard is in decline, and many subspecies and regional populations are highly threatened. Current research levels largely match those species with the greatest remaining range but neglect those subspecies that are the most in need of urgent attention.

## Supplemental Information

10.7717/peerj.1974/supp-1 Supplemental Information 1 Global leopard range. Global leopard range and subspecies delineations.

10.7717/peerj.1974/supp-2 Supplemental Information 2 Leopard range with presence records across Africa. Leopard range with presence records across Africa Leopard range with presence records across Africa: A–North Africa, B–West Africa, C–Central Africa, D–East Africa, E–Southern Africa. Numbers in black refer to extant, possibly present, and possibly extinct habitat patch IDs while those in white (or light grey) refer to extinct patches.

10.7717/peerj.1974/supp-3 Supplemental Information 3 Leopard range with presence records across the Middle East and Asia. Leopard range with presence records across the Middle East and Asia Leopard range with presence records and subspecies delineations across the Middle East and Asia: A–Middle East, B–Southwest Asia, C–South Asia, D–Sri Lanka. Numbers in black refer to extant, possibly present, and possibly extinct habitat patch IDs while those in white (or light grey) refer to extinct patches.

10.7717/peerj.1974/supp-4 Supplemental Information 4 Leopard range with presence records across eastern Asia. Leopard range with presence records and subspecies delineations across eastern Asia: A–Northeast Asia, B–North–Central China, C–Southeast Asia, D–Java, Indonesia. Numbers in black refer to extant, possibly present, and possibly extinct habitat patch IDs while those in white (or light grey) refer to extinct patches.

10.7717/peerj.1974/supp-5 Supplemental Information 5 Number of leopard publications by year. The number of peer-reviewed publications by year regarding wild leopard ecology or conservation between 2000 and 2014.

10.7717/peerj.1974/supp-6 Supplemental Information 6 Presence records. Presence records and sources. Note that we do not recommend simply taking these points and running a distribution model from them.

10.7717/peerj.1974/supp-7 Supplemental Information 7 Leopard patch IDs. Leopard patch names and IDs.

10.7717/peerj.1974/supp-8 Supplemental Information 8 Regional leopard range statistics. Range statistics among different regions of leopard range.

10.7717/peerj.1974/supp-9 Supplemental Information 9 Country persistence. Table of current leopard presence by country and region.

10.7717/peerj.1974/supp-10 Supplemental Information 10 Country statistics. Leopard range statistics by country and subspecies.

10.7717/peerj.1974/supp-11 Supplemental Information 11 Literature database. Literature organized by author accumulated via literature review process.

10.7717/peerj.1974/supp-12 Supplemental Information 12 Leopard population density estimates. Published leopard population density estimates by country and subspecies since 2000.

10.7717/peerj.1974/supp-13 Supplemental Information 13 Additional leopard range statistics by subspecies. Table of leopard range statistics by subspecies.

10.7717/peerj.1974/supp-14 Supplemental Information 14 Literature review categories. Literature content categories by subspecies.

10.7717/peerj.1974/supp-15 Supplemental Information 15 Leopard conservation organizations. Table of international leopard conservation and research organizations found on the Internet (in the English-language).

10.7717/peerj.1974/supp-16 Supplemental Information 16 Supplemental Document 1–Leopard Country Profiles. Profiles of leopard range countries.

## Additional Information and Declarations

### Data Deposition

The following information was supplied regarding data availability: The data are incorporated in the Supplemental Information.
