# Supplementary figures and images for: Leopard (Panthera pardus) status, distribution, and the research efforts across its range
Source: PeerJ. 2016 May 4;4:e1974. doi: 10.7717/peerj.1974 (PMC4861552; doi:10.7717/peerj.1974)

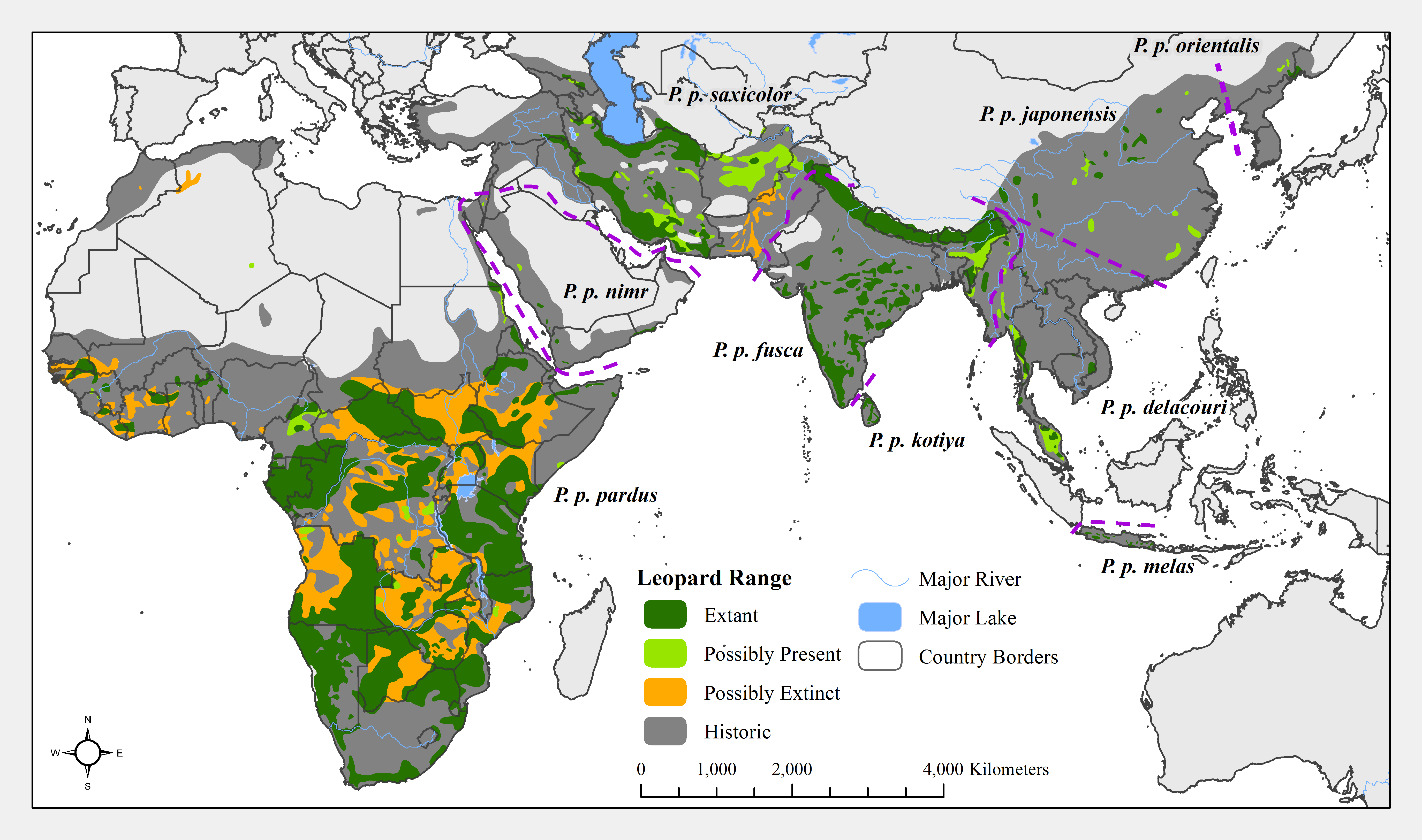

Supplement: Supplemental Information 1 — Global leopard range and subspecies delineations. [file peerj-04-1974-s001.png]

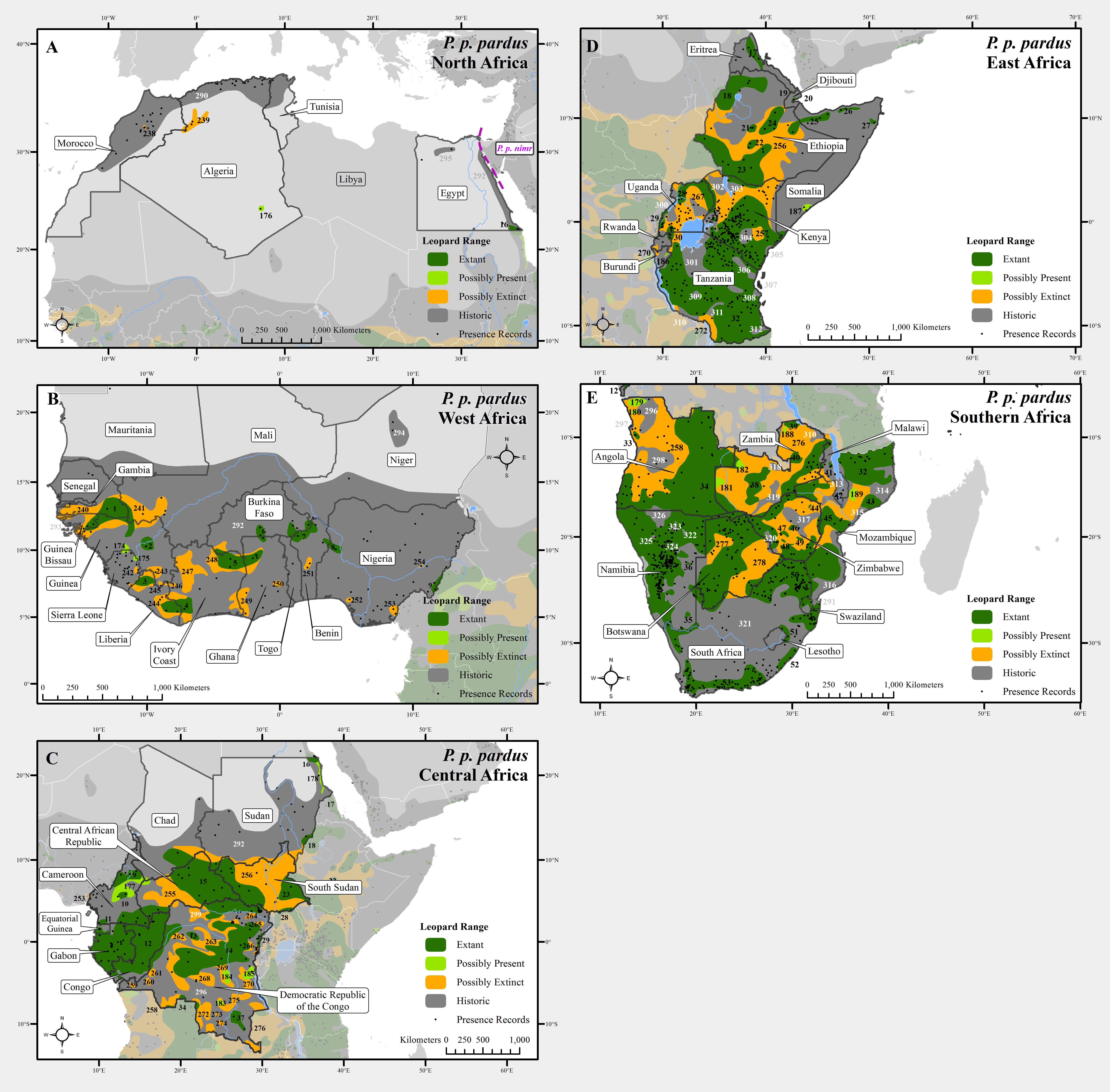

Supplement: Supplemental Information 2 — Leopard range with presence records across Africa Leopard range with presence records across Africa: A–North Africa, B–West Africa, C–Central Africa, D–East Africa, E–Southern Africa. Numbers in black refer to extant, possibly present, and possibly extinct habitat patch IDs while those in white (or light grey) refer to extinct patches. [file peerj-04-1974-s002.png]

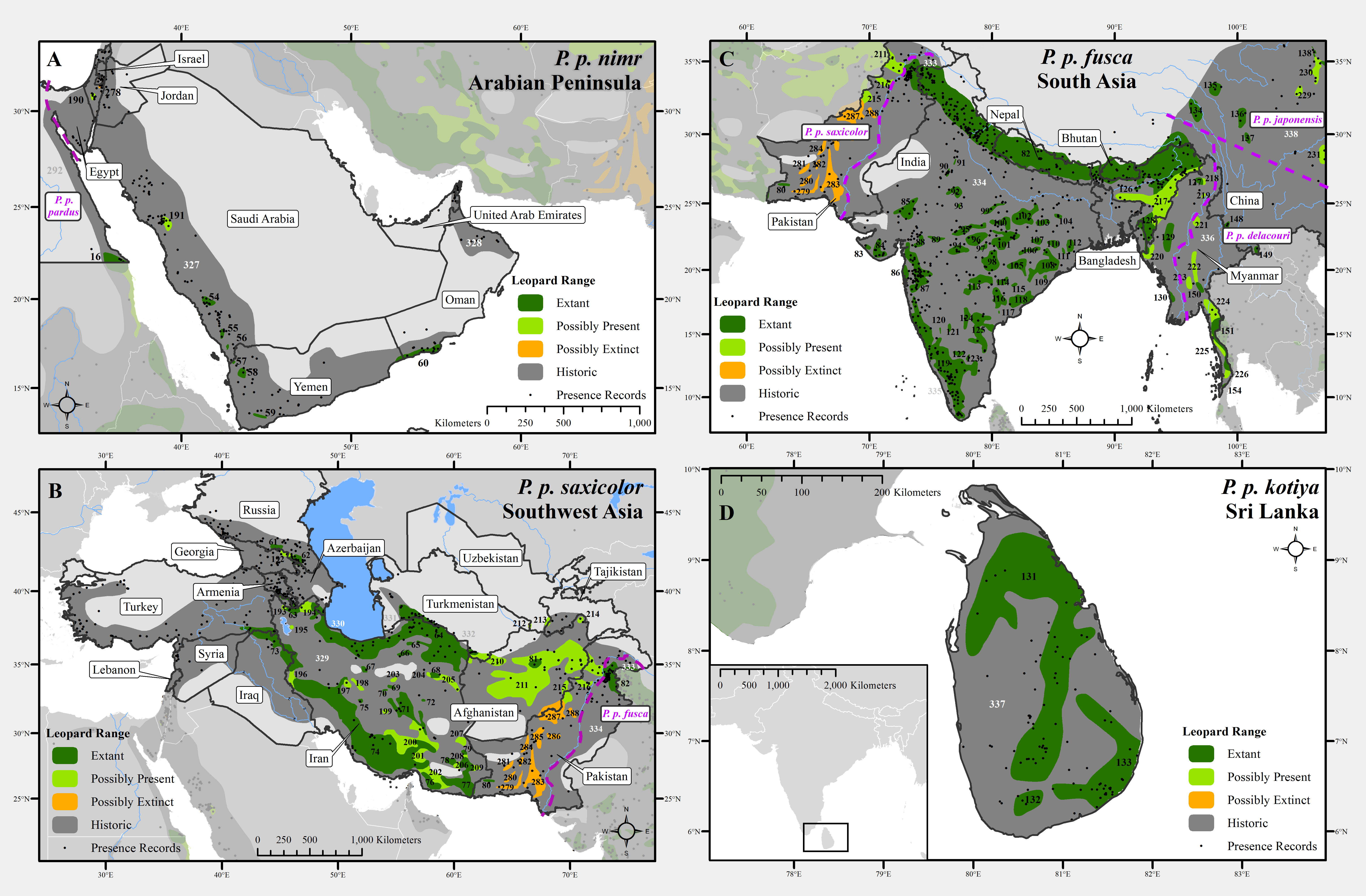

Supplement: Supplemental Information 3 — Leopard range with presence records across the Middle East and Asia Leopard range with presence records and subspecies delineations across the Middle East and Asia: A–Middle East, B–Southwest Asia, C–South Asia, D–Sri Lanka. Numbers in black refer to extant, possibly present, and possibly extinct habitat patch IDs while those in white (or light grey) refer to extinct patches. [file peerj-04-1974-s003.png]

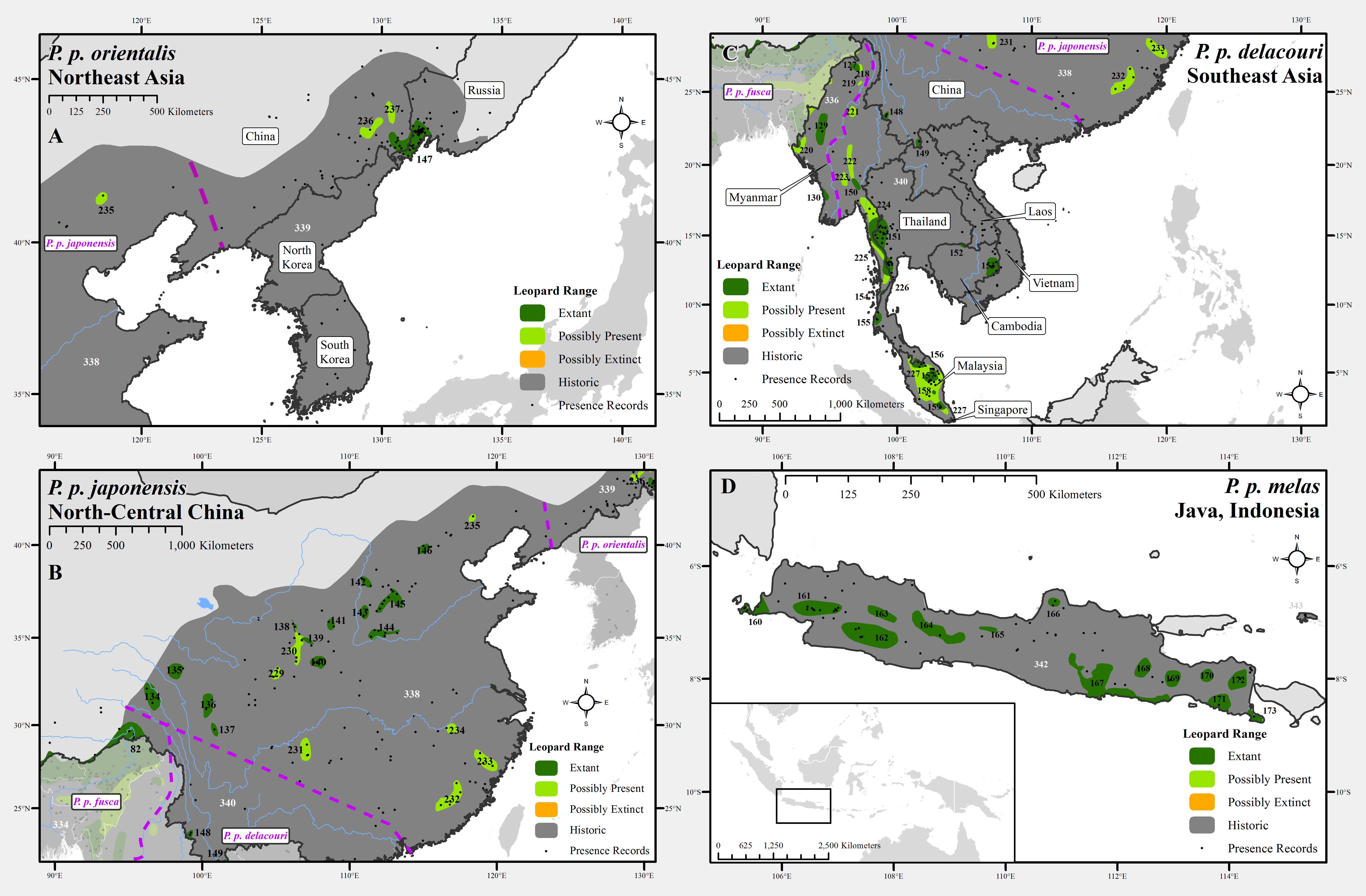

Supplement: Supplemental Information 4 — Leopard range with presence records and subspecies delineations across eastern Asia: A–Northeast Asia, B–North–Central China, C–Southeast Asia, D–Java, Indonesia. Numbers in black refer to extant, possibly present, and possibly extinct habitat patch IDs while those in white (or light grey) refer to extinct patches. [file peerj-04-1974-s004.png]
