# Supplementary material for: Leopard (Panthera pardus) status, distribution, and the research efforts across its range
Source: PeerJ. 2016 May 4;4:e1974. doi: 10.7717/peerj.1974 (PMC4861552; doi:10.7717/peerj.1974)
Supplement: Supplemental Information 16 — Profiles of leopard range countries. [file peerj-04-1974-s016.pdf]

Profiles for Leopard (*Panthera pardus*) Range Countries

Supplemental Document 1 to Jacobson et al. 2016

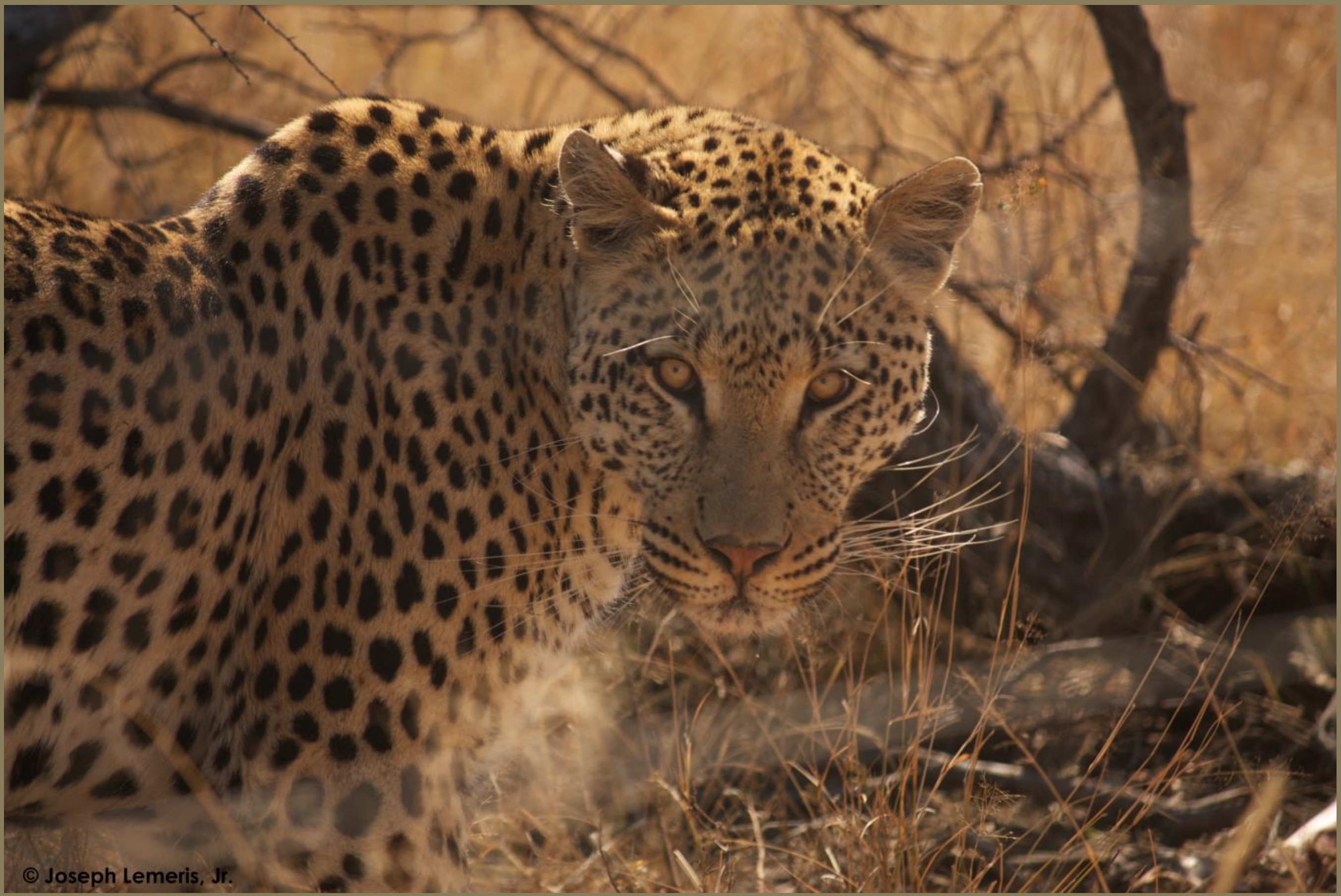

African Leopard (Panthera pardus pardus) 4
North Africa 5
West Africa 6
Central Africa 15
East Africa 20
Southern Africa 26
Arabian Leopard (P. p. nimr) 36
Persian Leopard (P. p. saxicolor) 42
Indian Leopard (P. p. fusca) 53
Sri Lankan Leopard (P. p. kotiya) 58
Indochinese Leopard (P. p. delacouri) 60
North Chinese Leopard (P. p. japonensis) 65
Amur Leopard (P. p. orientalis) 67
Javan Leopard (P. p.melas) 71
Table 1. Patch IDs 73
Bibliography 86

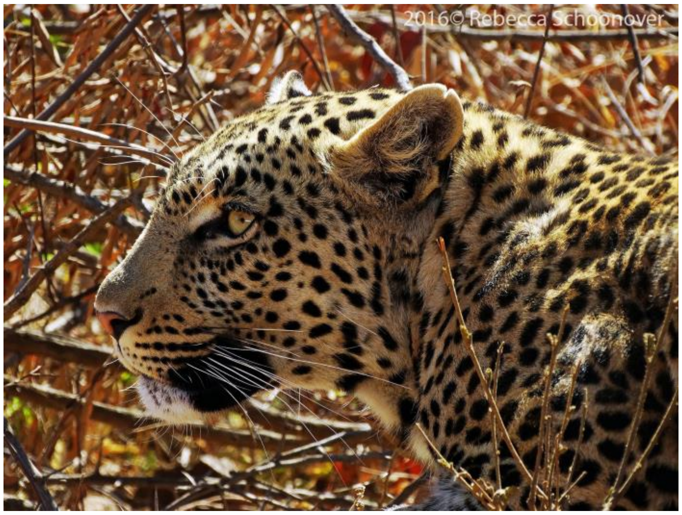

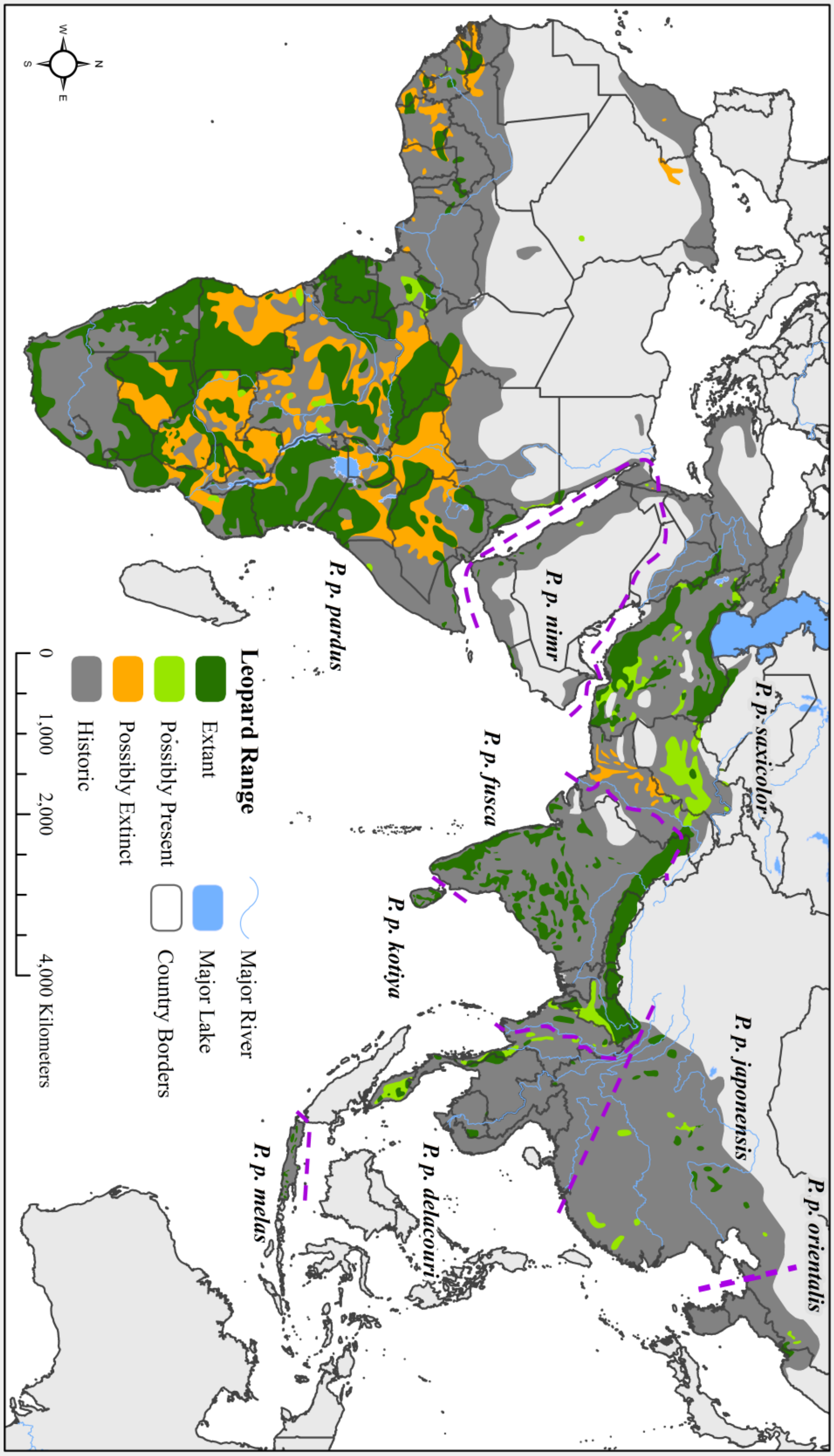
P. p. pardus
P. p. nimr
P. p. saxicolor
P. p. fusca
P. p. kotiya
P. p. japonensis
P. p. orientalis
P. p. delacouri
P. p. melas
Leopard Range
Extant
Possibly Present
Possibly Extinct
Historic
Major River
Major Lake
Country Borders
0
1,000
2,000
4,000 Kilometers
N
S
E
W

African Leopard

Panthera pardus pardus

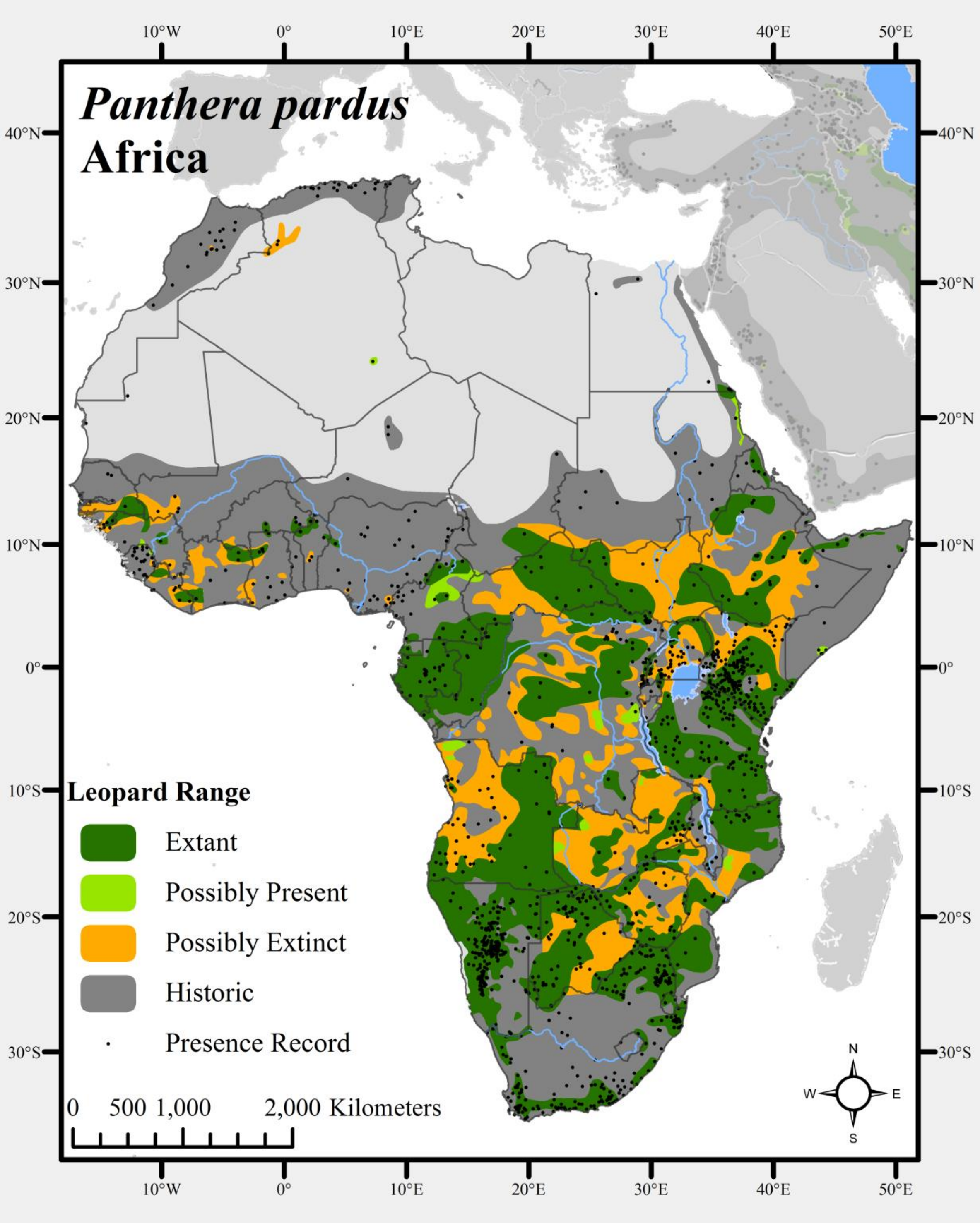

North Africa

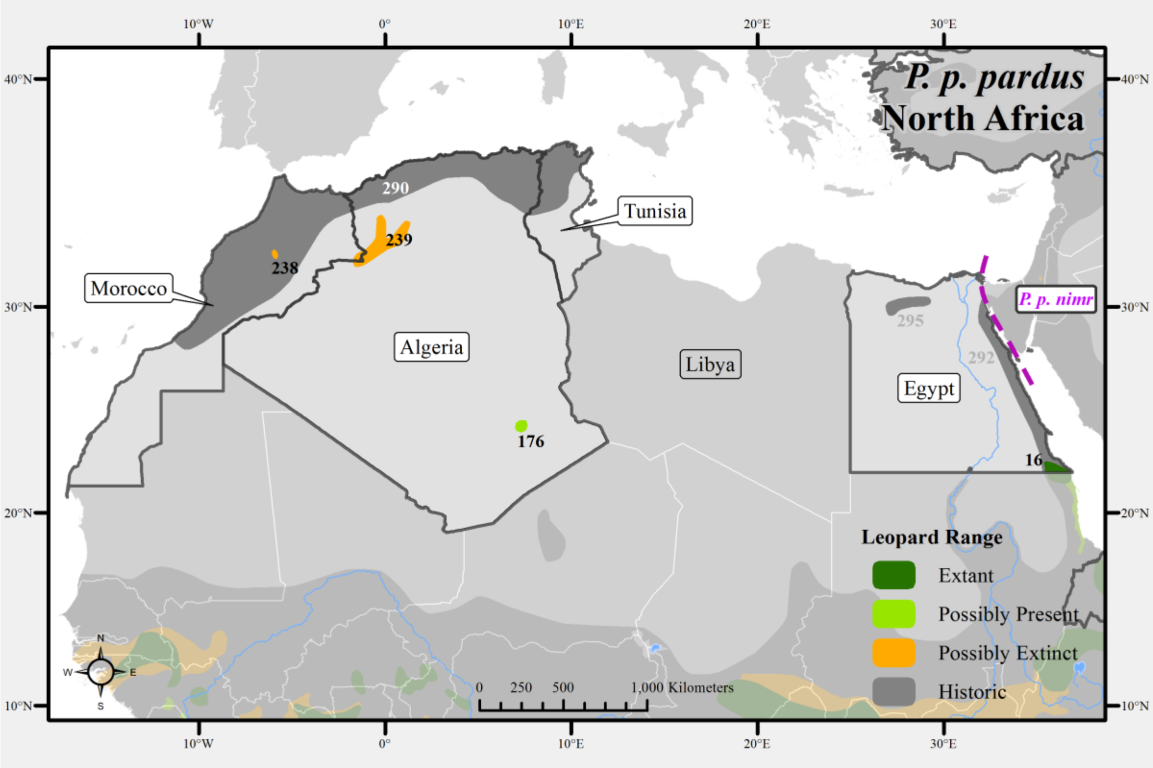

Algeria

Status: Possibly Present
Last Record: 2005 (Busby et al., 2009)

The leopard historically occurred along forested regions of the Mediterranean coastline and extended inland to the Tell and Saharan Atlas mountain ranges (Kowalski & Rzebik-Kowalska, 1991; P. Gerngross unpublished data). Over a period of just ten years, between 1873 and 1884, more than 1,200 leopards were killed throughout Algeria (Kowalski & Rzebik-Kowalska, 1991). The leopard was hunted for skins and for the bounty provided by the colonial administration. The leopard declined rapidly under the intense hunting pressure, nearly went extinct (Heim de Balsac, 1928), and has not recovered in the following decades.

The leopard may remain at very low numbers in the western Saharan Atlas Mountains and extend into Morocco (de Smet, 1989; Koen de Smet pers. comm. 2015). In 2007, Fabrice Cuzin reported a probable leopard presence near Figuig, Morocco (Henschel et al., 2008) and although unconfirmed reports continue there have been no confirmed sightings. The area is a military zone with restricted access, which has prevented access for detailed ecological surveys, but has also probably reduced poaching as large prey

items such as Barbary sheep (*Ammotragus lervia*), and Dorcas and Cuvier's gazelle (*Gazella dorcas* and *Gazella* cuvieri) remain (Koen de Smet pers. comm. 2015). There is insufficient data to claim this population is extirpated although the population has likely been critically small for decades.

In 2005, Busby et al. (2009) identified a single leopard scat via genetic analysis from the Ahaggar massif of Algeria, the first confirmed report of leopard in Algeria in over 50 years. However, leopards were never known to be present here before (although it is possible they were at low densities and simply overlooked or confused with cheetah [*Acinonyx jubatus*]) and it is far from existing populations. More research to confirm the leopard in this region is needed. Prior to this, leopards were thought to be extinct in Algeria since the mid-20th century (Kowalski & Rzebik-Kowalska, 1991). The last confirmed evidence of leopards in Algeria prior to Busby et al. (2009) was a report of a leopard killed in the extreme northeast province of El Tarf near El Kala (De Smet 1982; Kowalski & Rzebik-Kowalska, 1991) in 1960.

Egypt[1]

Status: Extant

Historically, the leopard likely occurred in the mountains of the Eastern Desert along the Red Sea coast (Osborn and Helmy, 1980), in the Nile Delta, and in the Qattara Depression (P. Gerngross unpublished data). Hoath (2003) cites a record of a leopard shot in the Qattara Depression in 1913. Leopard in Egypt was thought to be virtually extinct by the 1990's (Henschel et al., 2008; Hunter, Henschel and Ray, 2013). Yet, there were two leopard reports in 2010 from a shepherd and a Bedouin, in southeastern Egypt (Soultan et al., 2016). In 2014, a herder killed a leopard in Elba National Park in the Hala'ib triangle region, near the Red Sea and Sudan border (Soultan et al. 2016). The leopard may have been a transient individual from Sudan or it's possible a low density and small population of leopard has persisted in the Gebel Elba region for decades (Osborn & Helmy, 1980; Shoemaker, 1993; Soultan et al., 2016).

Libya

Status: Out of Historical Range

The leopard has not been recorded in Libya (Hufnagl, 1972).

Morocco

Status: Possibly Extinct
Last Record: 1983 (Drucker, 1991)

Historically, the leopard occurred in Morocco's Mediterranean coastal regions and the Atlas Mountains (P. Gerngross unpublished data), but by the 1950's the only remaining population was located in the Mid- (between Fes and Beni Mellal) and High Atlas (region of Marrakech) Mountains, where a population of 50-100 individuals were thought to still reside (IUCN, 1972 cited in Shoemaker, 1993). The population declined over

[1] See Egypt in the Middle East section for information on leopards in the Sinai Peninsula.

the 1980's and 1990's due to trophy hunting, retaliatory killings, and perceived threats to livestock (Shoemaker, 1993). Approximately 5-10 individuals were reported as "Morocco's last remaining population" in 1984 in the Oued-el-Abid/Bou Tferda region of the High Atlas Mountains (IUCN, 1972; Hafdaoui, 1984 cited in Shoemaker, 1993). The last confirmed killing of a leopard was a female at Idis, near Bou Tferda (Beni Mellal Province) in 1983 (Drucker, 1991). By 1996, the leopard population in the Atlas Mountains was estimated at no more than five individuals (Cuzin, 2003). Despite the small population, there continue to be reports of leopard around Bou Tferda into the 2000's (Purroy, 2010). In the Saharan Atlas Mountains, Fabrice Cuzin reported a probable record in 2007 on the Morocco-Algeria border near Figuig (Henschel et al., 2008). However, the region near Figuig is a military area with limited access and no reliable living records have been seen since (see Algeria for more information).

Tunisia

Status: Extinct
Last Record: NA

The few historical records available indicate that the leopard in Tunisia was restricted to the Tébessa Mountains, and previously forested regions in the northern part of the country (P. Gerngross unpublished data). In 1961, they were thought to exist in the forests between Bizerta and Tabarka, and in the mountain scrub at Tamerza (Chapuis) but are suspected to have gone extinct before 1970 (Shoemaker, 1993).

Note: Western Sahara

No information on current leopard population or distribution in Western Sahara was collected during this literature review. Eaton (1977) notes that no leopards were found in a survey of the Spanish Sahara by the U.S. Department of State. Due to its disputed political status, it may be that little research was performed in the region – however, extremely rare leopard occurrence in surrounding countries (Mauritania, Algeria, Morocco) suggests it is unlikely that leopards persist in Western Sahara.

West Africa

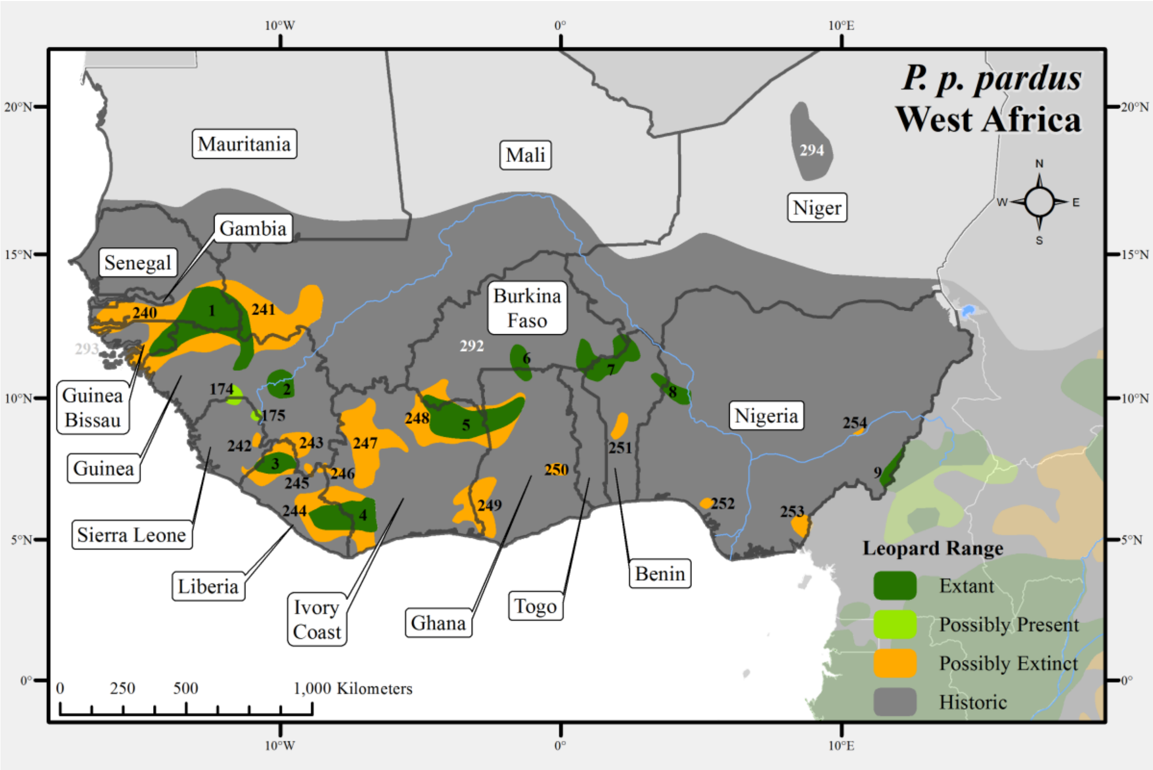

Benin

Status: Extant

Historically, the leopard occurred throughout the entire country (P. Gerngross unpublished data) but was already rare by the 1970's (Eaton 1977, Arnett 1982). Benin, along with Burkina Faso and Niger, is one of three countries that contain the W-Arly-Pendjari (WAP) Complex, a network of contiguous protected areas retaining some of the largest numbers of megafauna left in West Africa (Henschel et al. 2012). Leopards inhabit the WAP complex at low densities although the most recent data suggests the species may be recovering (UEMOA/PNUD, 2014). While Henschel et al. (2012) only recorded leopard tracks once during a spoor survey across the entire WAP complex in 2012, a repeat count following the same design conducted in 2014 revealed 31 spoor records (UEMOA/PNUD, 2014). Survey effort increased somewhat between 2012 and 2014, from 1110 km of transects to 1493 km. However, this is unlikely to have caused the marked increase in leopard records. Etotépé Sogbohossou (unpublished data) has also captured the species repeatedly by camera traps in the Pendjari National Park while surveying for cheetah.

Outside of the WAP Complex or contiguous areas thereof, there is little information on leopard and they may be absent from much of the country. Martin and de Meulenaer (1988) speculated on the presence of leopard

in a few other regions of the country including the Trois Rivières forest and the forests of L'Ouémé Supérieur, Wari Maro, and Monts Kouffé. However, the leopard is extinct from all these areas with the possible exception of the forests surrounding Monts Kouffé (Philipp Henschel pers. comm. 2015).

Trade in spotted felid skins is a direct and widespread threat for leopards throughout much of West Africa in general (Bailey, 1993) and in northern Benin in particular (Sogbohossou, 2006). However, Clark Lungren (pers. comm. 2016) noted that attempting to sell leopard skin is now quite rare in Benin and Burkina Faso, with most of the so-called leopard skins actually civet (*Civettictis civetta*) or serval (*Leptailurus serval*).

Burkina Faso

Status: Extant

The leopard historically occurred throughout all of Burkina Faso (P. Gerngross unpublished data), and remained widespread into the 1970's despite significant habitat degradation, persecution, and use in local medicines (Myers, 1976). Poisoning by herdsmen and hunting for skins contributed to population reductions starting in the 1960's (Clark Lungren pers. comm. 2016). Contemporary knowledge of leopards is restricted to the southeastern part of the country around the WAP complex, the PONASI ecosystem (Po, Nazinga, and Sissili), and the Comoé-Léraba community managed area adjacent to the Ivory Coast and Comoé National Park. Intriguingly, a leopard was recently killed in the extreme north of Burkina Faso but little is known about the incident (Clark Lungren pers. comm. 2016).

Burkina Faso, along with Benin and Niger, is one of three countries containing the WAP Complex, a network of contiguous protected areas retaining some of the largest numbers of megafauna in West Africa (Henschel et al. 2012). Médard, Traoré, & Berzins (2010) used interviews to ascertain the presence of leopards around the WAP complex in Burkina Faso. In several places, such as around the hunting camp Ougarou, or in Arly National Park, many guides or locals recognized the leopard, but in no place were they commonly seen (Médard, Traoré, & Berzins, 2010). Boulet et al. (2008) also reported observations of leopards from 2004-2008 on the Burkina Faso side of the WAP complex. Leopard spoor increased in the 2014 carnivore track survey compared to 2012 (Henschel et al., 2012), possibly representing a recovering population (UEMOA/PNUD, 2014). The rebounding population may be due in part to improving conditions in the privately managed hunting concessions of the Arly block, especially Konkombouri, Pama Centre Sud (Yéréyanga), and Pama Nord (Clark Lungren pers. comm. 2016).

Outside the WAP complex, local trackers suggest that leopards are present at low densities in the PONASI complex, at least in the Nazinga and Sissili components, and in the Comoé-Léraba classified forest (Clark Lungren and M. Karama pers. comm. 2016).

Ivory Coast

Status: Extant

Leopards were historically distributed throughout the country (P. Gerngross unpublished data). Shoemaker (1993) referenced older reports to indicate leopards may be rare outside of protected areas but were thought

to remain in all national parks. There is little recent evidence or research of leopard outside of protected areas. Martin and de Meulenaer (1988) note relatively little dedicated leopard poaching although they are used in traditional culture and may be killed in the bushmeat trade.

Bodendorfer et al. (2006) documented leopard occurrence in the 1990's in Marahoué National Park near the center of the country. However, the leopard has vanished from the area and surroundings due to heavy poaching and excessive bushmeat trade which caused a crash in ungulate and primate populations (Philipp Henschel, pers. comm, 2015).

Henschel et al. (2010) detected scat, tracks, and vocalizations of leopards in localized areas of dense gallery forest in Comoé National Park. Hoppe-Dominik et al. (2011) conducted transect-surveys in Taï National Park and confirmed the presence of leopards through visual observations and scat counts. Previously, Jenny (1996) estimated a leopard density of 7-11 individuals/100 km^2 in Taï National Park, the first scientific estimate of leopard densities from African rainforest.

Gambia

Status: Possibly Extinct
Last Record: 2000 (Craig Emms pers. comm. 2014)

Historically distributed throughout Gambia (P. Gerngross unpublished data), the leopard remained widespread into the 1970's, before becoming increasingly rare in the 1980's (Grubb, 1998). With only anecdotal reports of leopards for decades, at best there are only small, scattered populations of leopards in Gambia (Roy Armstrong pers. comm. 2014). Shoemaker (1993) commented on the lack of available information, and mentions substantial habitat loss/conversion restricting any remnant leopard population.

There was a sighting of a leopard in Kiang West National Park in 2000, which may still have good prey populations (Craig Emms pers. comm. 2014). Emms also found leopard tracks in 2001 on Jinack Island, part of Niumi National Park, on the north bank of the River Gambia. The area is small and the leopard was potentially a migrant from Saloum Delta National Park in Senegal. The best remaining leopard habitat in Gambia is Kiang West and Bao Bolon National Parks with possible leopard presence in the Kakima Delta, and the Bama Kuno and Kahlenge forest parks (Craig Emms pers. comm. 2014).

Ghana

Status: Extant

Historically widespread throughout the entire country (Grubb et al., 1998; P. Gerngross unpublished data), leopards already started to disappear from Ghana by the 1950's according to Dr. E.O. Asibey, Chief Administrator of the Ghana Forestry Commission (Martin & de Meulenaer, 1988). Rapid habitat loss/conversion, exploitation, persecution, and prey reduction due to the bushmeat trade significantly affected leopards in Ghana (Myers, 1976; Shoemaker, 1993). By 1971, the leopard was considered very rare in most areas (Shoemaker, 1993). At the time of the Shoemaker report, authorities in Ghana considered the

leopard nationally endangered. While the leopard received full protection of the law, the legal presence required to enforce wildlife protection was considered inadequate (Shoemaker, 1993).

Since Shoemaker, there have been limited mentions of the leopard in Ghana. The leopard is thought to have recently gone extinct around the Muni-Pomadze Ramsar site (Ryan & Attuquayefio 2000). Jachmann (2008) indicated the leopard is one of several common large mammals found in the Bia Conservation Area (comprising the Bia National Park and Bia Resource Reserve) and Appiah-Opoku (2011) mentioned leopard presence in Kakum National Park although neither stated the evidence to support these claims. Burton et al. (2011) gathered evidence of leopards in Mole National Park during camera trap and spoor transect surveys for lions (*Panthera leo*) between 2006-2009 and reported camera trap detection rates of 2.9 detections/100 trap days.

Guinea

Status: Extant

Once found throughout the entire country (P. Gerngross unpublished data), widespread encroachment and habitat conversion, have likely restricted the abundance and distribution leopards in Guinea (Shoemaker, 1993). Myers (1976) thought leopards may persist in fair numbers and be widespread. However, Teleki (1980) believed extensive habitat destruction probably limited the leopard population to no more than 400-500 individuals.

More recently, Brugière et al. (2005) interviewed hunters in villages along the border of Guinea and Guinea-Bissau in 2003 and January 2004. Over 80% of hunters in both countries recognized the leopard with the fewest interviewees recognizing leopard in coastal areas. Interview results suggested that leopard populations were declining, livestock depredation was widespread, and opportunistic hunting for leopard skin was also widespread (although possibly less so in Guinea than Guinea-Bissau) (Brugière et al., 2005). The leopard was relatively less common on the Guinean side of the border and seen as relatively more common than the lion. Directly south in the unprotected landscape around Sangaredi, leopards have not been seen for some time (Vincent Lapeyre pers. comm. 2016). Farther east, in the Foutah Djallon area of the Guinean highlands, leopards were spotted at least yearly in camera traps (Vincent Lapeyre pers. comm. 2016).

Ziegler, Nikolaus & Hutterer (2002) noted there was a "reliable report" of leopards in the Upper Niger National Park (Parc National du Haut Niger) at the time of its establishment in 1997. Houpline (2010), however, conducted a study in the park and did not document any leopards. They identified local threats such as habitat loss, decline of natural prey populations, and conflict with people. The study highlighted the illegal trade in wildlife parts coming from large carnivores, including 227 skins of leopards and 67 skins of lions over a period of only six months (Houpline, 2010). Leopards remain there (documented in a camera trap in 2015 and regular indirect evidence; Vincent Lapeyre pers. comm. 2016) but due to the small size of the core area of this park and the depleted prey populations, the leopard population is likely very small (Philipp Henschel pers. comm. 2015).

Guinea-Bissau

Status: Extant

Historically, leopards occurred throughout all of Guinea-Bissau (P. Gerngross unpublished data). An IUCN wildlife survey report from 1989 (cited in Shoemaker, 1993) documented leopards as present but rare throughout the country. More recently, leopards have been recorded in the southern and eastern part of the country, between the Geba river and the borders with Guinea and Senegal, and also in a few areas to the west of the Geba river, along the coast and near the border with the Casamance region, Senegal (Brugiere et al., 2005). Thibault (1993 cited in Brugière et al., 2005) reported the presence of leopards in the proposed Dulombi National Park located between the Corubal and Geba Rivers. Presence was confirmed in this area by recent camera trap records in the adjacent Boé protected area (Chimbo Foundation, 2014).

According to Brugière et al. (2005), over 80% of hunters in Guinea and Guinea-Bissau recognized the leopard with the fewest interviewees recognizing leopard in coastal areas. The leopard is possibly more common in Guinea-Bissau than Guinea according to the interviewees. Hunting is likely the gravest threat to large carnivores in the region (Brugière et al., 2005).

Liberia

Status: Extant

Historically, leopards were once "rather common" throughout Liberia (Martin & de Meulenaer, 1988) and were still fairly evenly distributed outside of mining and farming areas in the 1970s (Myers, 1976). However, anthropogenic expansion has had significant negative impacts on leopard populations and resulted in major habitat loss/conversion in Liberia (Shoemaker, 1993). The population was suspected to be no larger than 100 individuals in 1980 (Teleki, 1980). However, in contrast to other countries in West Africa, larger forested areas remain in Liberia. Though area-wide data are lacking, the northwestern (Gola Forest) and southeastern (Sapo National Park) parts of the country might still harbor leopard populations despite widespread persecution and habitat loss (Philipp Henschel pers. comm. 2015).

Hoke, Demey & Peal (2007) surveyed North Lorma and Grebo National Forests and identified leopard via tracks, dung, and camera traps. Vogt (2011) documented leopards in Sapo National Park during a 2007-2009 study. While no leopards are documented on the Liberian side of Gola National Park, Lindsell, Klop & Siaka (2011) documented leopard presence on the Sierra Leone side, allowing for the possibility of leopards to cross into Liberia from this trans-boundary protected area (Lindsell, Klop & Siaka, 2011).

Mali

Status: Possibly Present
Last Record: NA

Historically, the leopard occurred in the West Sudanian Savanna region of southern Mali and in parts of the adjacent Sahelian Acacia Savanna to the north (P. Gerngross unpublished data). There are rumors the

leopard once occurred in the Adrar des Ifoghas, a massif in northeast Mali, but supporting evidence is lacking (P. Gerngross unpublished data). Myers (1976) acknowledged continued leopard presence in the 1970's primarily in the western region but suspected that growing human populations and habitat loss would lead to the extirpation of leopards from most of the country. According to Sayer (1977), leopards were thought to be relatively common in the rocky hills of the Mandingue plateau, and tracks were occasionally seen in the Baoulé area.

The last possible leopard habitat is in the Guinean ecoregion of southwest Mali. This region covers only 6% of Mali and a USAID report suggested that "Mali's large mammal population has disappeared," and specifically mentioned the leopard to be "near extinction" (USAID, 2008). The evidence behind these statements is unknown but is plausible based on lack of leopard presence for decades.

Mauritania

Status: Extinct
Last Record: 1950's (José Carlos and Cândida Gomes Vale pers. comm. 2015).

No publications confirming the status and/or presence of leopards in Mauritania were collected during this literature review. Historic leopard range is restricted to the southern part of the country in the Sahel and the Sudan-Guinean zone (P. Gerngross unpublished data). Myers (1976) kept leopard presence to areas south of Mauritania and does not discuss Mauritania as potential leopard habitat. Martin and de Meulenaer (1988) suggested leopard presence in the country but Shoemaker (1993) was unable to confirm any other information about leopards in the country. The last leopard seen in Mauritania was in the 1950's, although Jose Carlos Brito and his colleagues sighted a potential leopard in 2009 in the Dakhlet-Nouadhibou province, western Mauritania (José Carlos and Cândida Gomes Vale pers. comm. 2015).

Niger

Status: Extant

Historically leopards were present throughout the Sahel zone of Niger and in the Aïr Mountains of northern Niger (P. Gerngross unpublished data). However, they are currently nearly extinct except for a small corner of range as part of the WAP complex in the southwest part of the country. Poaching by the military, poisoning, trade in skins, and prey loss contributed to the decline of leopards (Millington & Tiega, 1991). Large mammals were estimated to have declined by 90% between 1970 and 1990 (Millington & Tiega, 1991). Indeed, leopards were very rare (Eaton, 1977) or possibly extinct by 1991 (Millington & Tiega, 1991).

Leopards are extinct in the Aïr and Ténéré National Nature Reserves. The explorers Barth and de Bary found leopards common in Aïr during the 19th Century, but Guggisberg (1975) described leopard occurrence in that part of the Sahara as rare. More recently, between 1979-1991, Newby did not find any leopard sign in the Aïr and Ténéré National Nature Reserve (John Newby pers. comm. 2011).

The only remaining range for leopards in Niger is W National Park, part of the WAP complex. Niger, along with Benin and Burkina Faso, is one of three countries containing the WAP Complex, a network of contiguous protected areas retaining some of the largest numbers of megafauna in West Africa (Henschel et al., 2012). Henschel et al. (2012) and UEMOA/PNUD (2014) did not find any sign of leopard presence in Niger but villagers killed one leopard just outside W National Park in Niger in 2012 (Philipp Henschel pers. comm. 2015). The species is certainly present in W but appears to occur in very low numbers. Refer to Benin (above) for more information about leopards in the WAP Complex.

Nigeria

Status: Extant

Historically, the leopard occurred throughout all of Nigeria (P. Gerngross unpublished data) but has likely suffered among the most severe population and range declines of any country in Africa (Henschel et al., 2008). Threats to Nigerian wildlife include unsustainable harvesting by subsistence and commercial hunters, conversion of natural habitat, and insufficient funding and support across agencies (Myers, 1976; Drolet, 1990). Leopards have disappeared from much of the West African coastal forest belt by 1945 and were largely relegated to protected areas by the 1970's (Myers 1976; Happold 1987). Happold (1987) compiled various dates and locations of leopard presence including the Bende region, Benue-Pai river region, Ogoja Province, Gorgoram, Gwoza, Ikot Mbo, Kona, Mapeo, Okigwi, and the Gashaka, Gumti, Kambari, Kwiambana Game Reserves, Ohosu Forest Reserve, and Kainji Lake National Park, and uncertain presence in Yankari Game Reserve. In southeast Nigeria, Angelici, Akani & Luiselli (1998) documented leopard in only 2 of 47 surveyed forest patches. Relatively recent surveys confirmed the leopard still occurs in several isolated protected areas in eastern and southern Nigeria. Based off scat and spoor, Ikemeh (2007) document leopard presence in Okomu National Park, Okomu Forest Reserve and Weppa Farms. Ikemeh argues that there is a small but recovering leopard population in Okomu National Park. Henschel et al. (2010) found field sign of leopard in a remote part of northern Gashaka-Gumti National Park on the eastern border with Cameroon. Leopards were also detected during lion survey work in the western sector (Borgu) of Kainji Lake National Park (Henschel et al., 2014a; Table S3). Leopards may also still be present in Cross River National Park in the southeast (Okon, 2005); however, leopards have yet to be spotted on camera despite years of camera trap survey efforts on Cross River gorillas (*Gorilla gorilla diehli*) and their continued presence is doubtful. Finally, based off interview surveys in 2009, leopards were considered absent in Kamuku National Park – Kwiambana Game Reserve (Henschel et al., 2010).

Senegal

Status: Extant

Historically the leopard occurred throughout Senegal in the Sudanian savannah and Sahel zones (P. Gerngross unpublished data), although they were even occasionally found in the Ferlo desert to the north (Myers, 1976, Eaton, 1977). They were especially common along the riverine and gallery forest patches (Myers, 1976). In 1973, trade in leopard pelts was still between 100-200 a year; indicating a sizable local

population (Myers, 1976). However, leopard presence was gradually reduced and detailed information on leopard presence in the country is sparse.

Craig Emms suggested leopards may still be present at the coast, at Delta du Saloum National Park (Craig Emms pers. comm., 2014). However, the best leopard habitat remaining in Senegal is in the southeast around Niokola-Koba National Park. Leopards were documented throughout the 1990's in the park (di Silvestre, Novelli & Bogliani, 2000), and a park-wide large carnivore spoor survey conducted in 2011 estimated a population size of 400 individuals (Ndao & Henschel, 2011). Niokolo-Koba may well harbor the largest remaining leopard population in West Africa, and should be a top priority for the species' conservation effort in the region (Philipp Henschel pers. comm. 2015). A recent camera trap study estimated 2-4 leopards per 100 km^2 within a small study area inside the park (Kane, Morin & Kelly, 2015).

Sierra Leone

Status: Extant

Historically, leopards occurred throughout all of Sierra Leone (P. Gerngross unpublished data), yet only one publication documenting leopards since 2002 (the end of Sierra Leone's civil war) has been identified. Leopards remained highly abundant and widespread throughout the country well into the 1920's (Grubb et al., 1998). However, by the 1970's, they were confined to only ~20% of the country (Myers, 1976), mostly in areas heavily used by ranchers. By 1980, only 50-100 leopards were left in the country, and they were likely isolated in protected areas (Teleki, 1980). Martin and de Meulenaer (1988) believed that leopards existed into the 1980's in Outamba-Kilimi, the Loma Mountains Game Reserves, in Gola North, East, and West Forests with a few hanging on in the forests south of Freetown and in the Kangari Hills Game Sanctuary. Leopards were later found extinct in the forests south of Freetown (Anonymous, 2007a). Finally, although leopards were thought extinct from Gola Forest Reserve (Shoemaker, 1993), Lindsell, Klop & Siaka (2011) identified leopard presence while conducting line transects in the forest from 2005-2007.

Togo

Status: Extinct
Last Record: NA

Historically, leopards occurred throughout all of Togo (P. Gerngross unpublished data), yet no publication or records of leopards was identified after the year 2000. Leopards were very rare by the 1980's and authorities believed no more than 50 remained spread across Fazao Malfakassa, Keran National Park, Keran Hunting Reserve, and Togodo Forest (Martin & de Meulenaer, 1988). Leopards were possibly still present into the 2000's (Henschel et al., 2008) but are unlikely to remain extant.

Central Africa

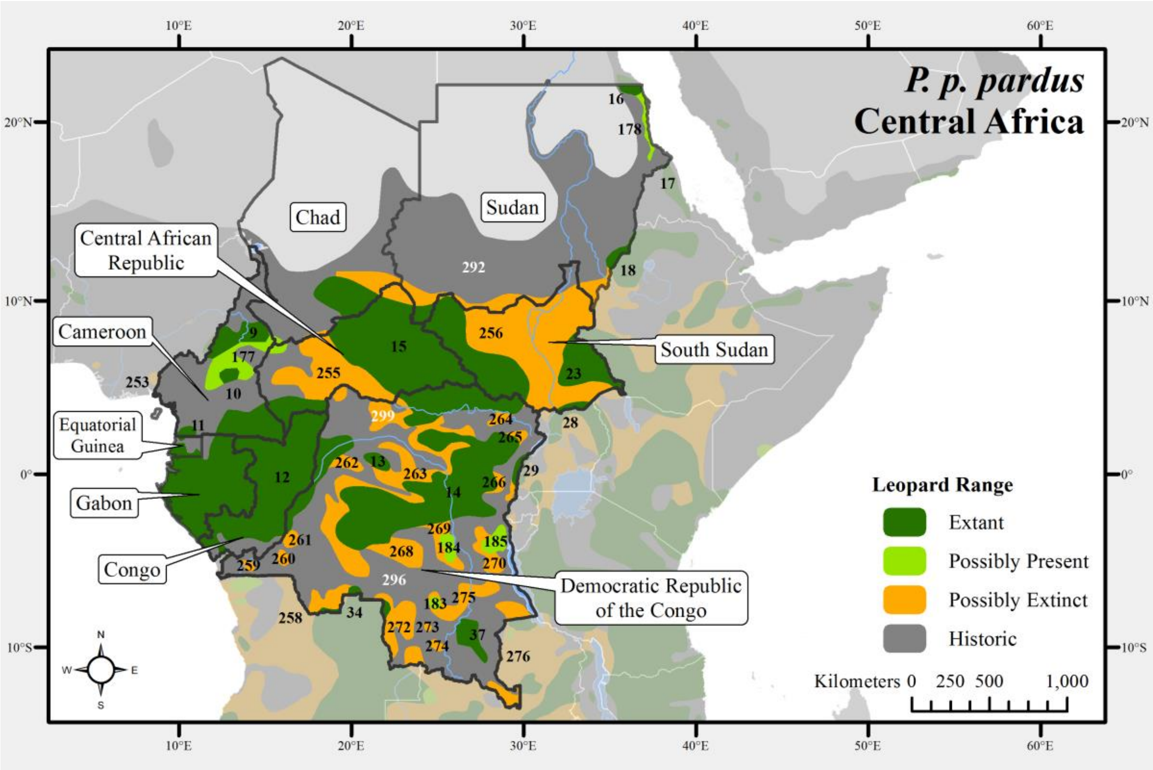

Cameroon

Status: Extant

Historically the leopard occurred throughout the entire country (P. Gerngross unpublished data) but its present distribution in Cameroon is fragmented. Myers (1976) noted that fair numbers of leopards survived in the southeast of the country with only scattered populations elsewhere. They also disappeared from most forest reserves in southwest Cameroon by the early 1980's (Maisels et al., 2001). Almost 30 years later the distribution is still largely true, although leopards are definitively present across the middle part of the country in the Bénoué Complex. Croes et al. (2011) conducted spoor transects across the national parks and hunting zones of the Bénoué Complex in 2007-2010 and found the densities of leopards within the national parks to be roughly 1.28 individuals/100 km^2 while the density was slightly higher in the surrounding hunting zones (~1.46 individuals/100 km^2).

Elsewhere in the country, Henschel conducted a literature review of leopard presence across the southern rainforest zone of Cameroon, confirming presence in Campo Ma'an, Dja, Mbam et Djerem, Lobeke, and Boumba Bek Nki (Henschel, 2008; Henschel, 2009). Henschel (2008) did not find evidence of leopards in several protected areas throughout the southwestern region of Cameroon, including Douala Edea Forest Reserve, Ebo Forest, Korup National Park, Mount Cameroon Forest Reserve, and Takamanda Forest Reserve. Leopards were absent from two different surveys in 2002-2005 in Banyang-Mbo Wildlife Sanctuary (Ray,

Hunter & Zigouris, 2005; Henschel, 2008). Leopards are also absent from Waza National Park with no tracks or sightings since at least the 1980's (Bauer & Kari, 2001) and have been extinct in the montane forests of Kilum-Ijim for at least 30 years (Hunter & Balme, 2005). Finally, camera traps failed to find leopards in Korup National Park, southwest Cameroon, between 2011-2013 (http://www.teamnetwork.org/site/korup-national-park, accessed December 2015).

Poaching and trophy hunting have been primary drivers of the leopard's decline in Cameroon (Croes et al., 2011). A 1996 ban on leopard trophy hunting may have led to a resurgence of leopard populations in some areas (Croes et al., 2011). Protected areas are also strongly linked to leopard persistence according to modeling results from Toni and Lodé (2013). They exhaustively gathered information on leopard presence and absence throughout Cameroon to investigate potential distribution and factors associated with persistence. They gathered 416 leopard occurrences and 245 absences. Leopards were primarily distributed in the Bénoué Complex, the southeast and scattered in a few protected areas elsewhere. Leopards seemed to be reliant on areas of low human population density and absent from Cameroonian rainforests where bushmeat extraction is high (Toni & Lodé, 2013). Long-term persistence of the leopard in the Congo Basin likely depends on strict regulation of excessive and unsustainable bushmeat harvesting practices (Henschel et al., 2011).

Central African Republic (CAR)

Status: Extant

Leopards historically occurred throughout the entirety of the Central African Republic (P. Gerngross unpublished data) but there is relatively little recent documentation of their status and distribution. Myers (1976) noted that leopards were common in many areas of CAR although absent near agricultural areas due to poisoning campaigns in the 1950's and 1960's. Leopards remained common into the 1980's in the north, northeast and in the rainforest region of the south (Martin & de Meulenaer, 1988).

Currently, it is possible that large wildlife populations including carnivores persist due to large intact swaths of habitat and low human population densities. However, heavily armed poaching gangs from Sudan and Chad sweep across CAR slaughtering wildlife (East, 2006). A comparison of aerial survey wildlife counts across 95,000 km^2 of northeastern CAR found large mammal populations had been decimated, declining ~94% between 1978 and 2010 (Bouché et al., 2012). Yet, continued leopard presence is noted in many locations throughout the country and they may still be present in as much as 85% of the country according to Pierre-Armand Roulet (pers. comm. 2015). Pierre-Armand Roulet (pers. comm. 2011) noted that leopards may still be present in the northwest corner of the country near the border with Cameroon and Chad. In the southwest, Ray and Sunquist (2001) suggested continued leopard presence in Dzanga-Sangha National Park, and Melletti et al. (2009) identified leopard scat and spoor during surveys from 2002-2004 in the Bai-Hokou, a sector of Dzanga-Ndoki National Park. Also in the southwest, Roulet (pers. comm. 2011) reported the leopard is commonly spotted in Ngotto Forest. Roulet (pers. comm. 2011) reported leopard tracks and kills are spotted along the Oubangui River, south of the Bangassou Forest in the south and center of the country. In addition, recent camera trapping studies carried out in the remote Chinko Area revealed the persistence of leopards in southeastern CAR (http://www.chinkoproject.com; accessed in November 2015). However, long-term persistence of the leopard in the Congo Basin will depend on strict regulation of excessive and unsustainable bushmeat harvesting practices (Henschel et al., 2011). On the eastern border with South Sudan,

leopards were the most commonly spotted carnivores in the Zemongo Faunal Reserve (Roulet et al., 2007). Finally, Boulet et al. (2008) collated 95 leopard observations between 2004 and 2008, primarily from hunting zones in the eastern part of the country.

Chad

Status: Extant

Historically, the leopard occurred south of the Sahara Desert in the Sahelian Acacia Savanna and the East Sudanian Savanna zone with the Ennedi Massif as the northernmost refuge (P. Gerngross unpublished data). No publications regarding the recent status or presence of leopards in Chad were collected during this review. Already in the 1970's, leopards were considered scarce due to inadequate habitat (G. Child cited in Teer & Swank, 1977) and were only resident in the southern third of the country (Anna, 1971 cited in Shoemaker, 1993). However, leopards are still present in Zakouma National Park; staff observe them regularly and have photographed at least one in 2015 (Tim Wacher pers. comm. 2015).

Republic of the Congo

Status: Extant

The leopard historically occurred throughout the entire country (P. Gerngross unpublished data) and likely remains widely distributed (Henschel, 2008) although there is little recent information. Myers (1976) noted that human population density was low throughout much of the country and much of the heavily forested country was potential habitat. This remains the assumption. Martin and de Meulenaer (1988) speculated that leopards were common throughout the country except for the Pool area, the extreme southeast, and within 50 km of Brazzaville where most wildlife has been poached out. More recently in 2007, Henschel et al. (2010; Henschel, Malanda & Hunter, 2014) documented leopard via sign and camera traps in Odzala-Kokoua National Park. Henschel (2009) collated a number of sources to argue that leopards remain present in a number of protected areas throughout the Congo including Nouabale-Ndoki National Park - Ososky, 1998; the proposed Ogooué-Leketi National Park - Aust et al., 2005; Lesio-Louna Reserve – Mathot, Ikoli & Missilou, 2006; and Conkouati-Douli - H. VanLeeuwe. Yet, long-term persistence of the leopard in the Congo Basin likely depends on strict regulation of excessive and unsustainable bushmeat harvesting practices (Henschel et al., 2011). The illicit trade in leopard skins poses another serious threat to leopards throughout the country, with specialized leopard hunters operating specifically in northeast Congo (Henschel & Ray, 2003).

Democratic Republic of the Congo (DRC)

Status: Extant

Historically ubiquitous throughout the DRC (P. Gerngross unpublished data) and found up to 4200 m on the Rwenzori Mountains (Verschuren, 1987 cited in Shoemaker, 1993), the leopard is likely still widespread (John Hart pers. comm. 2015). Shoemaker (1993) speculated that the DRC, along with Congo and Gabon "is and will [continue to] be the largest reservoir of leopards for a long time," although Henschel (2009) and others

(Jackson, 1989; Norton, 1990; Bailey, 1993; Jenny, 1996) argued that prey densities are a limiting factor in Central African forest ecosystems. Indeed, there is limited recent information on leopards from the country and densities remain unknown. D'Amour, Hohmann & Fruth (2006) found leopard sign in Salonga National Park in a survey from 2004. Henschel et al. (2010) documented leopard presence in Upemba National Park in 2008. Leopards are also still present in Garamba National Park (Natalia Casado Bolaños pers. comm. 2014). John Hart (pers. comm. 2015) reported that while evidence of leopards is abundant in the Ituri Forest, Bili-Uere, Salonga, and TL2 landscape, a fast growing human population of 70 million has put unsustainable pressure on prey populations and has led to crashes of the leopard's primary prey base. Long-term persistence of the leopard in the Congo Basin likely depends on strict regulation of excessive and unsustainable bushmeat harvesting practices (Henschel et al., 2011). Hart additionally noted that leopard attacks on livestock and people were relatively low.

Equatorial Guinea

Status: Extant

Absent from nearly half of Equatorial Guinea today (Henschel, 2008), leopards historically occurred throughout the country (P. Gerngross unpublished data). Presently, leopards are likely restricted to a small population at Monte Alén National Park in the center of the country (Henschel 2008), and to the edges of the country. Though over half of Equatorial Guinea is forested, oil production and the bushmeat trade likely have pushed leopards out of many unprotected areas (Henschel, 2009). Leopards were not detected during surveys in either Rio Campo Reserve or Altos de Nsork National Park (Larison et al., 1999). Henschel (2009) also found no data about leopard in Estuario del Muni Reserve and concluded they may be absent.

Gabon

Status: Extant

Gabon historically hosted leopards throughout the entire country (P. Gerngross unpublished data). Leopards were speculated to be widespread and numerous in the 1970's (Myers, 1976) and the country likely still supports significant leopard populations with populations in virtually all protected areas (Henschel, 2008; Henschel, 2009). Using camera trap and individual recognition, Henschel et al. (2011) estimated population densities of 2.7, 4.6 and 12.1 individuals per 100 km^2 in three study sites near Ivindo National Park with densities significantly increasing with increasing distance from human establishments. Henschel (2009) cited a number of other authors to indicate presence in other locations of Gabon: Moukalaba-Doudou National Park – Boddicker, 2006; Loango National Park – Boddicker, 2006; Birougou National Park - Nseme & Ndoukoue, 2007; Batéké Plateau National Park – Bout, 2006; Waka National Park – Abitsi, 2006; Lopé National Park - Henschel et al. 2005; Pongara National Park – Latour, 2006; Monts de Cristal National Park – Nseme, 2006; Minkébé National Park – World Wildlife Fund unpublished data, and Mwagné National Park – Panthera unpublished data. Additionally, Henschel (2009) suspected leopards were present in Wonga-Wongué Reserve although there was no supporting data. An ongoing concern in Gabon is the intensity of bushmeat extraction as small and medium sized ungulates are targeted by hunters and are the preferred prey of leopards. Indeed, leopards are disappearing in some forested regions where bushmeat hunting is most

intense (Hunter & Balme, 2004). Long-term persistence of the leopard in the Congo Basin likely depends on strict regulation of excessive and unsustainable bushmeat harvesting practices (Henschel et al., 2011).

Sudan

Status: Extant

Historically, leopards were likely absent from the Sahara but present everywhere else including along the Nile River, along the eastern coast of the Red Sea Hills, and in the Sahelian and Sudanian savannas (P. Gerngross unpublished data). We know little about wildlife, including leopards, in Sudan after decades of political turmoil and instability (UNEP, 2007). For a while it seemed that political conflict may have reduced poaching rates and allowed wildlife to flourish (Myers, 1976) but this may no longer be the case as wildlife numbers (albeit limited to certain geographic regions) show significant declines (UNEP, 2007). The north and west are primarily desert and were unlikely to ever contain much wildlife. The area bordering the Nile River is heavily populated and supports only birdlife and small animals (UNEP, 2007). The Sahel belt has largely been converted to agriculture, but much of the rest of the country was productive savanna (before South Sudan became an independent country in 2011) habitat and some areas contained tremendous wildlife resources (Myers, 1976; UNEP, 2007). Shoemaker (1993) reported that in the 1970's and 1980's it was thought that leopard populations in northern Sudan and along the Red Sea were mostly fragmented, while a substantial population existed in the region south of Khartoum (Myers, 1976; Teer & Swank, 1977). Even by the mid-1970's heavy poaching and desertification was taking a toll on wildlife in Darfur and Kordofan Provinces in the west and the Red Sea province in the east (Myers, 1976; Martin & de Meulenaer, 1988). The Marra plateau and Nuba Mountains in Darfur and Kordofan respectively were being heavily poached in the 1990's with massive wildlife declines (UNEP, 2007). Most of the leopard populations in previous literature relate to locations now in South Sudan, although leopards may still be present in Dinder National Park (Nowell & Jackson, 1996), but this is unconfirmed and the park is starved of support and funding (UNEP, 2007). Leopards may still be present in the arid mountains along the Red Sea (UNEP, 2007) and in 2014 a herder killed a leopard in the Hala'ib triangle region of southeastern Egypt, near the Red Sea and Sudan border (Alaa Eldin pers. comm. 2014).

South Sudan

Status: Extant

Historically, the leopard occurred throughout South Sudan except for the Sudd wetland (P. Gerngross unpublished data). South Sudan gained independence from Sudan in 2011, although political instability and conflict have continued, hampering research and conservation. Much of the literature about wildlife from South Sudan is from decades ago and before the country's independence. No published data were found regarding the current status or distribution of leopard in South Sudan. Myers (1976) noted that southern Sudan (i.e., the area south of Khartoum) "could become some of the best wildlife country left in eastern Africa." In 1983, leopards were present in all wildlife conservation areas of southern Sudan including Boma, Lantoto, Nimule, and Southern National Parks, and Ashana, Kidepo, Cheikou, Boro, Numatina, Juba, Meshra, Fanyikang, Zeraf, Shambe, Badingilo, Bangangai, Bire Kpatuous, and Mbarizunga Game Reserves (J. Hillman

pers. comm. cited in Shoemaker, 1988). Nowell and Jackson (1996) speculated leopard presence in Southern National Park although there has been no recent confirmation. More recent information is limited but available knowledge suggests large-scale wildlife declines across South Sudan due to uncontrolled hunting (UNEP, 2007). The most detailed information comes from aerial surveys in 1980 and 2001 across Boma National Park showing precipitous drops in all herbivore populations (UNEP, 2007). UNEP (2007) acknowledge that almost all available recent information is anecdotal and poorly substantiated, however, based on their collected information, they concluded that many protected areas in South Sudan likely still had "remnant populations of most species." The only positive recent evidence of leopard presence is that on the border with Uganda in the Imatong Mountains, hunters recently reported leopard presence (Tom Butynski, Yvonne de Jong & Gerhard Nikolaus, pers. comm. 2015).

East Africa

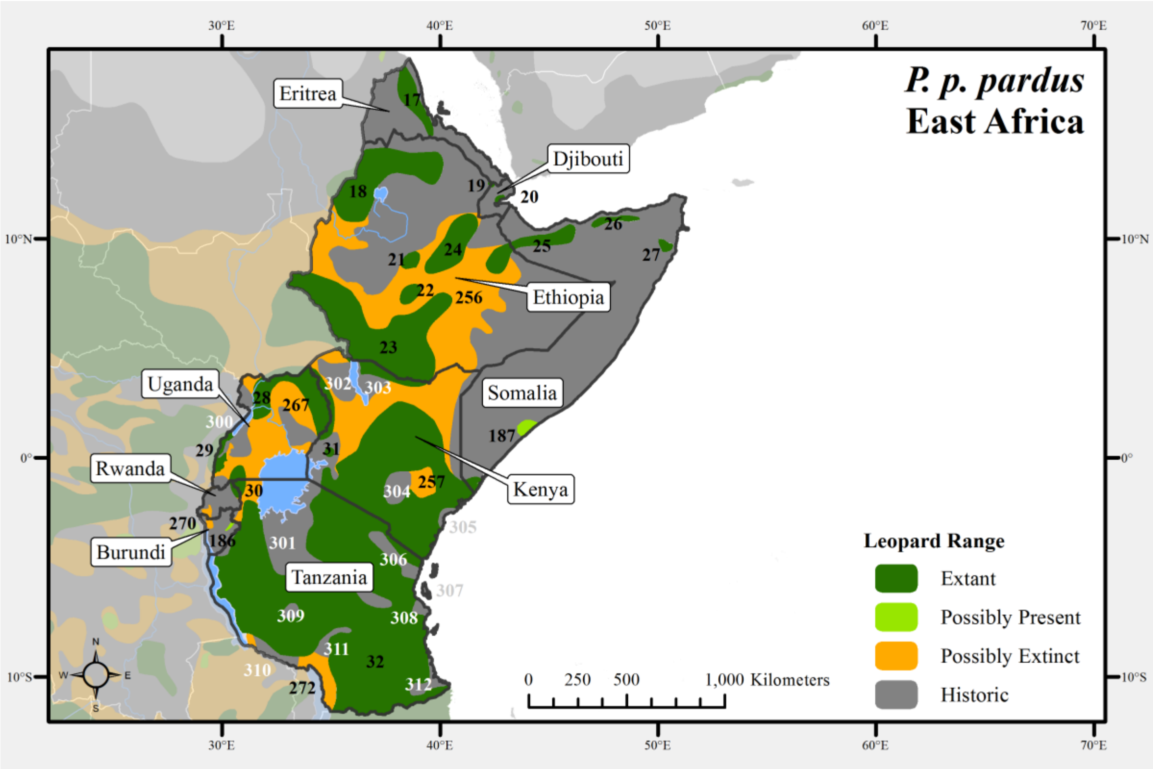

Burundi

Status: Possibly Present
Last Record: NA

Historically, the leopard occurred throughout Burundi (P. Gerngross unpublished data) yet its present status is poorly known. Field surveys by Viv Wilson (1990 in Shoemaker, 1993) estimated 272 leopards throughout Burundi, mainly in Ruvubu and Kibira National Parks and Vyanda Forest Reserve. However, these numbers were thought too high and by the time of the Shoemaker report, the estimated population in Burundi was estimated at only 120 individuals (Viv Wilson and Verschuren pers. comm. in Shoemaker, 1993). Much of the country is converted to agriculture (Jacobson et al., 2015) with high human population densities and low wild prey densities (Shoemaker, 1993). The leopard is no longer observed in Vyanda or Kibira (Nyiratuza Madeleine and Ildephonse Kambogo pers. comm. 2016) although it possibly remains in Ruvubu National Park (Nzigidahera Benoit pers. comm. 2016).

Djibouti

Status: Extant

The leopard historically occurred throughout Djibouti (P. Gerngross unpublished data) although no publications regarding the recent presence of leopards there were collected during this review. However, it is likely that leopards still exist due to some anecdotal reports. In the 1980's and 1990's leopards were noted as present in the mountains of Randa, near the city of Tadjourah, in the Day Forest ecosystem, and in the Moussa Ali mountains on the border with Ethiopia (Jackson, 1988; Shoemaker, 1993). More recently, a conservation workshop in 2012 on Djibouti's priority land mammals suggested leopard presence in 6 small, isolated regions although the data supporting that distribution is unknown (Holst et al., 2013). The patches include a population on the northern border with Ethiopia near the tri-country border with Eritrea and Ethiopia, the largest patch centered on the Day forest ecosystem, a coastal population between the town of Arta and the Gulf of Tadjoura, an inland population south of Arta, a population on the southern border with Ethiopia and near the tri-country border with Somalia and Ethiopia, and a sixth population on the western border with Ethiopia surrounding Lake Abbe (Abhe) (Holst et al., 2013). In one of these regions, the Day Forest ecosystem of the Goda Mountains, locals report continued persistence of the leopard, and a leopard killed several cattle and camels in September 2015 (Houssein Rayaleh pers. comm. 2015). In SE Djibouti, near the village of Holl Holl in the Ali-Sabieh region, a male leopard was killed sometime after 2010 (Houssein Rayaleh pers. comm. 2015). In addition, Rayaleh photographed a leopard skin at Asbole's tourist camp near Lake Abbe in western Djibouti, December 2015. Locals reported it was freshly killed on the Le'ado plain on the northeast side of the Lake (Houssein Rayaleh pers. comm. 2015).

Eritrea

Status: Extant

Eritrea historically hosted leopards throughout the country (P. Gerngross unpublished data), yet no publications regarding the recent presence of leopards in Eritrea were collected during this review. Shoemaker (1993) speculated that numbers are "probably low due to previous trapping activities during the 1970's for skins as well as high human populations, degraded habitat, poor prey base", and warfare with Ethiopia. Since that time, there are only unconfirmed sightings and conflicts with leopard. During their research on baboons in central Eritrea in 1997-1998, Zinner et al. (2001) found leopard spoor but never saw or heard a leopard, despite the Ministry of Agriculture belief that leopards may have increased after the conflict with Ethiopia ended in 1991. Overall, leopards may still exist in a few regions (Tom Butynski pers. comm. 2014). The remaining highland forests throughout central and eastern Eritrea are likely habitat (Anonymous, 2014). There are reports of leopards in the Mrara and Fil Fil areas of Semenawi Bahri, in Elaberid, Debresina, Wasdama Mountain and Geleb in the Anseba region, around Huger Mountain in the Nakfa region, and along the eastern escarpment of central Asmara region, particularly near the Debre Bizen Monastery (Dawit Berhane pers. comm. 2015).

Leopards may also exist along much of the border with Ethiopia in the Gash Barka region (Dawit Berhane pers. comm.). Ethiopia has confirmed leopard sightings (Demeke & Aklilu, 2008; Yirga & Bauer, 2011; Yimer & Yirga, 2013), and populations may extend across their borders into Eritrea. Indeed, the riverine forests in

Gash Barka are also habitat (Anonymous, 2014) and, the latest potential sighting of a leopard in the country was early January 2015 near the border with Sudan and Ethiopia (Dawit Berhane pers. comm. 2015).

Ethiopia

Status: Extant

Leopards historically occurred throughout all of Ethiopia (P. Gerngross unpublished data). Densities were very high in certain forested regions (Myers, 1976) and considered high in Wollega, Illubahor, Kaffa, Gemu Goffa provinces and "reasonable" in Gondar, Gojjam, Harrar, and Shoa provinces (Martin and de Meulanaer 1988). Shoemaker (1993) acknowledged leopards were found widely across the country although their populations were depressed in some regions due to degraded habitat, like the Central Highlands, Ogaden, Danakil and the Northwest areas. More recently, there are only limited records of leopards in various areas throughout Ethiopia although Gebresenbet et al. (2009) reported "several thousand" leopards following the 2009 National Lion Conservation Workshop and they are listed as Least Concern by the Ethiopian 2008 Red List. Gonfa et al. (2015) documented leopard presence in 2013 during line transects in Datil Wolel National Park. Yimer and Yirga (2013) reported one leopard sighting in Mazie National Park (southwestern Ethiopia) during their studies from 2007-2008. Presence was also noted in the Babille Elephant Sanctuary (Demeke & Aklilu, 2008) and May Anbesa in the Northern Ethiopian Highlands (Yirga & Bauer, 2011).

Kenya

Status: Extant

Historically, the leopard occurred throughout all of Kenya (P. Gerngross unpublished data) although densities varied greatly across the country (Hamilton, 1981). Hamilton (1981; 1987) conducted a thorough population assessment of leopards in Kenya, estimating between 10,000-12,000 leopards concentrated in Maasailand and forested regions of central Kenya. Hamilton (1981) noted high leopard densities in several regions (at least 1 per 15 km^2) including Nairobi province, Mount Kenya, the Aberdares, Tsavo, and Lake Nakuru. Shoemaker (1993) suggested that leopards were largely absent from the arid regions of north and eastern Kenya apart from Samburu but more widespread in the south including in 32 conservation areas. This distribution largely holds true today.

The Kenya Wildlife Service notes leopard presence in Amboseli, Chyulu Hills, Hell's Gate and Mt. Longonot, Mt. Kenya, Ruma, and Tsavo (East, West) National Parks, Meru National Park and adjacent forest reserves, Boni and Dodori National Reserves, and the Mau forest complex in their respective management plans (http://www.kws.go.ke/downloads, accessed December 2015). The management plans mention some specific threats to populations as well, such as poisoning by local herders near Amboseli, human-wildlife conflict near Hell's Gate and Ruma, and some isolated cases of "trophy poaching" at Mt. Kenya. Leopards are also commonly known from Nairobi National Park and Masai Mara National Reserve. Juliet King notes leopards are widespread in the conservancies of northern and eastern Kenya, possibly only avoiding extremely arid areas in the far north and northeast, and dense settlements. They remain extant in the Kirisia Forest, Matthews Range, southern Samburu, Tana River Primate Reserve/Ishaqbini conservancy, and along

the Ewaso Nyiro and Tana rivers (pers. comm. 2016). Based off research on human-wildlife conflict events, leopards are also present in unprotected lands surrounding the Masai Mara National Reserve(Kolowski & Holekamp, 2006), Tsavo National Park (Patterson et al., 2004), Amboseli National Park (Okello, Bonham & Hill, 2014; Okello, Kiringe & Warinwa, 2014) and around Laikipia (Ogada et al., 2003; Woodroffe et al., 2007).

Romañach, Lindsey & Woodroffe (2007) and Ogada et al. (2003) document leopard presence in the Laikipia district of central Kenya. At the Mpala ranch, O'Brien and Kinnaird (2011) used camera traps to estimate between 8.4 and 12 leopards per 100 km^2 according to various algorithms. Svengren (2008) used a variety of leopard sign and DNA analysis of scat to estimate a minimum of 13 leopards around Sangare Ranch Conservancy in the southern part of Laikipia.

Rwanda

Status: Extant

Despite little published information, Gerngross (in prep.) believe the leopard historically occurred throughout Rwanda. No publications regarding the recent status and/or presence of leopards in Rwanda were collected during this literature review. Much of the country is converted to agriculture (Jacobson et al., 2015) with high human population densities. Verschuren (1987 cited in Shoemaker, 1993) suggested leopards were present in Akagera National Park, and they likely still are present based off claims from the African Parks NGO (African Parks, n.d.). Shoemaker (1993) found no information on leopards in Volcanoes National Park, Mutara Hunting Reserve, the Gishwati Forest Reserve or the Nyungwe Forest Reserve. Deogratias Tuyisingize (pers. comm. 2014) agrees and suggests the leopard may be absent from Volcanoes National Park. Leopards also appear to be absent from the Nyungwe/Kibira protected areas (Beth Kaplin, Nyiratuza Madeleine and Ildephonse Kambogo, pers. comm. 2016).

Somalia

Status: Extant

Historically present throughout Somalia, (P. Gerngross unpublished data), leopards were described as "plentiful" in Somaliland (Drake-Brockman, 1910). Very few publications regarding the recent status and/or presence of leopards in Somalia were collected during this literature review. Data is largely unavailable due to continuing unrest in the region (Amir, 2006). Leopard skins were heavily exported (reaching 5,000 or 6,000 a year) and by the 1960's leopard numbers were at very low levels (Funaioli & Simonetta, 1966; Shoemaker, 1993). Habitat degradation also became a concern such that even if poaching lessened, numbers would continue to be low (Martin & de Meulenaer, 1988). However, leopards likely continue to persist in a few regions. Sommerlatte and Umar (2000) surveyed 10,000 km^2 of the coastal plains including the region between Berbera (east) and Loyada (west) and confirmed the leopard still existed in the foothills but were poached for their pelts and sold in the markets of Djibouti. A more recent assessment on the wildlife trade in Somalia noted a decline of leopard skins in Somalian markets although it is possible that is because the animal itself is very rare (Amir, 2006). Leopards may still persist in the forested escarpments of Gaan Libaax and the Daallo Forest in Somaliland according to local reports and where a possible leopard scat was found

during field work in 2010 (David Mallon pers. comm. 2014). Leopards may also persist in some southern areas of Somalia. Leopards were spotted between 2006 and 2008 in the Lower Shabelle ("shebel" means leopard in the Somali language) region while other carnivores (e.g. lion, and African wild dog, *Lycaon pictus*) have continued to be seen up to 2012 (Abow, 2013).

Tanzania

Status: Extant

Leopards historically occurred throughout Tanzania (P. Gerngross unpublished data) and remain widely distributed although they are extinct on Zanzibar (see below; Foley et al., 2014). On the mainland, wildlife authorities regarded the leopard as "extremely common" up through the 1980's (Martin & de Meulenaer, 1988). Leopards even inhabited the upper reaches of Mt. Kilimanjaro as suggested by the discovery of a carcass on the rim of Kibo Crater in 1926 at 5,700 m (Moreau, 1944 cited in Estes, 1991). Various researchers estimated large national populations up to 100,000 individuals (Spong et al., 2000; Martin & de Meulenaer, 1988). However, few studies have scientifically estimated leopard densities or gathered population trends. An old study in Serengeti National Park estimated roughly 1,330 individuals based on home ranges of a small number of animals and sex ratios (Bertram, 1982; Stander, 1998). Density estimates for Tarangire National Park are between 6-10 leopards/100 km^2 (Msuha, 2009). Based on eight radio-collared individuals, male home ranges were estimated at 136 km^2, and 25 km^2 for females suggesting relatively high densities in the Rungwa/Piti ecosystem (Caso, 2002).

A large national population is also reflected in high export quotas. The Convention on International Trade in Endangered Species (CITES) awarded Tanzania one of the highest export quotas for leopard trophies and an average of 303 wild leopard trophies were exported per year between 1996 and 2006 (Packer et al., 2010). However, upon review, excessive harvests may have driven a decline in leopards similar to but less extensive than those seen in lions (Packer et al., 2010).

Certainly, there has been population decline possibly starting in the mid-1960's (Myers, 1976). A survey of leopard occurrence between 1955 and 1975 split the country into 1200 quarter degree squares and concluded the leopard was present in over 90% of the units (Shoemaker, 1993). Throughout Tanzania however the leopard is declining in the face of expanding agriculture and growing human populations (Myers, 1976). Currently, they are found throughout all national parks except Gombe National Park (52 km^2) and Rubondo National Park, an island in Lake Victoria (Foley et al., 2014). They are also present in all of the Game Reserves, Nature Reserves, many of the larger Forest Reserves, as well as at low densities in some unprotected mountain ranges such as the Pare and Usambara Mountains (Foley et al., 2014). Finally, Foley et al. (2014) believe they are well distributed throughout much of the unprotected landscapes.

Leopard presence is collated through the Tanzania Mammal Atlas project. This project contributed to the Tanzania Lion and Leopard Conservation Action Plan workshop, which identified distribution, threats, conservation needs, and data gaps for both species (Durant et al., 2006).

Zanzibar

Status: Extinct
Last Record: 1995 (Goldman & Walsh, 2007)

The Zanzibar leopard was poorly known and possibly an endemic subspecies to the main island, Unguja Island, of Zanzibar (Goldman & Walsh, 1997). An island-wide eradication program organized by the government after the Zanzibar Revolution in 1964 had devastating impact (Goldman & Walsh, 2007). Compounding this, rapid human population growth, expansion of agriculture, and conflict with people were key threats (Goldman & Walsh, 2007). Although anecdotal reports continue, the leopard probably went extinct on the island in the mid 1990's (Goldman & Walsh, 2007).

Uganda

Status: Extant

Leopards historically occurred throughout Uganda (P. Gerngross unpublished data) and though they were still widespread by the 1970's, they were uncommon and possibly rare (Eaton, 1977). Decline was attributed to habitat loss, prey declines, human-leopard conflict, and the skin trade (Anonymous, 2007b). Leopards remained throughout agricultural areas and even in more densely populated areas close to Kampala into the 1980's (Martin & de Meulenaer, 1988). More recently, the Uganda Wildlife Authority crafted the *Uganda Large Carnivore Action Plan* that detailed leopard distribution, threats, and identified goals for leopard conservation (Uganda Wildlife Authority, 2010). Unlike the other large carnivores, leopards were sighted regularly both inside and outside protected areas (Uganda Wildlife Authority, 2010). However, they report that leopards are 'likely to have declined even more drastically [relative to other species of concern] because of their widespread presence outside protected areas,' but that limited data are available to confirm this. Additionally, there are no good population estimates anywhere for the species and the population may be lower than 150-200 individuals (Uganda Wildlife Authority, 2010).

Currently, leopards are extant in Queen Elizabeth, Mt. Elgon, Murchison Falls, Lake Mburo and Kidepo Valley National Parks as well as Ajai, Bokora, Pian-Upe, and Toro-Semiliki Wildlife Reserves (Uganda Wildlife Authority, 2010). Additionally, leopard tracks were observed in the Imatong Mountains near the South Sudan border in October 2014 (Tom Butynski and Yvonne de Jong pers. comm. 2015). Leopards also remain in the Greater Virunga landscape with Queen Elizabeth a likely stronghold for the species (Uganda Wildlife Authority, 2010). In other parts of Greater Virunga, leopards may be absent in some locations and at low densities in others (Treves et al., 2010). The leopard disappeared from the Bwindi Impenetrable National Park in the 1970's (Butynski, 1984) and camera trap surveys by the Wildlife Conservation Society failed to find presence in Kasyoha-Kitomi and Kalinzu Forest Reserves (Anonymous, 2007b). Despite conflicting evidence, leopards appear to persist at low densities throughout the Rwenzori Mountains and are at best only transient in Kibale National Park; human pressure surrounding these areas is great and connectivity is restricted (Anonymous, 2007b; Treves et al., 2010; Colin Chapman pers. comm. 2015).

Southern Africa

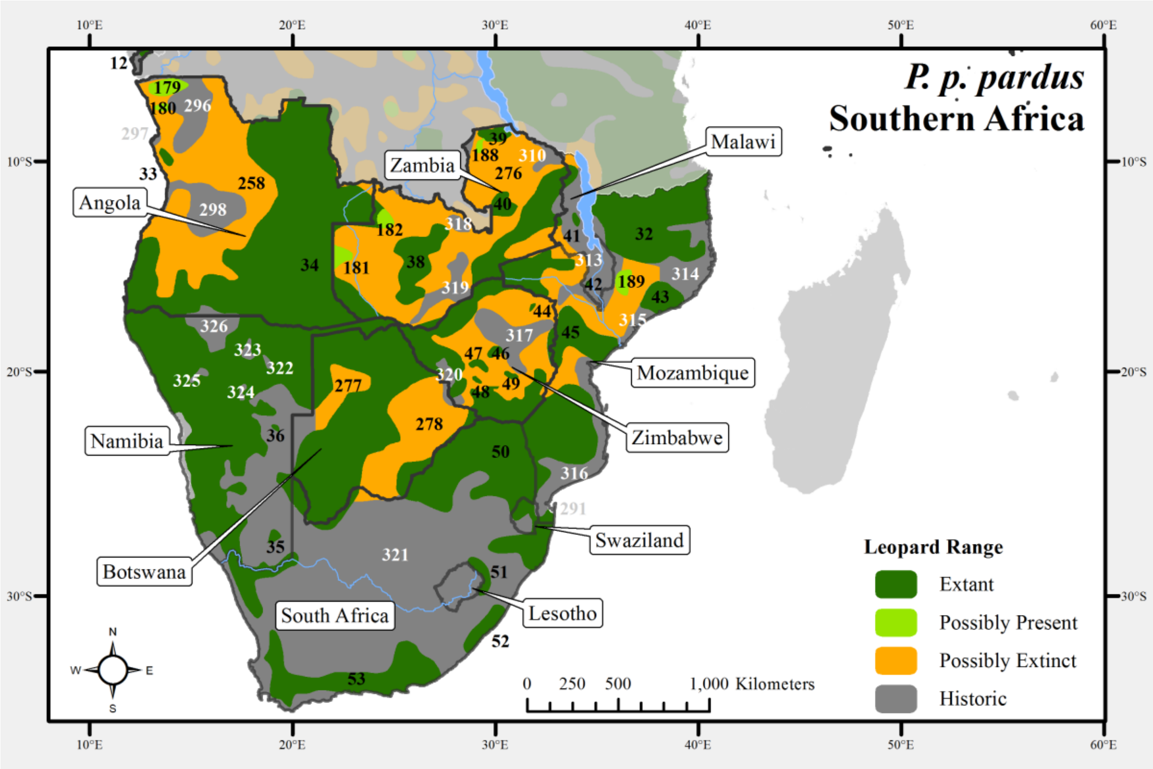

Angola

Status: Extant

No recent publications regarding the presence of leopards in Angola were collected during this literature review although the leopard is suspected to have historically occurred throughout Angola (P. Gerngross unpublished data). Myers (1976) believed that Angola had a very large leopard population at least into the 1950's. Leopards were reported in 11 protected areas and reserves in 1982 (Horsten, 1982). However, Angola descended into civil war after independence in 1975, but due to low human population densities, the leopard population may have remained stable (Shoemaker, 1993). Despite a cessation of the civil war in 2002, conservation and research is only just starting to return. The government of Angola (http://www.angola.org/index.php?page=tourism, accessed December, 2015) provides vague detail stating that leopard can be found in Iona and Kameia National Parks, while a recent travel guide (Stead et al., 2013) mentions that leopards can be seen in the Namibe province. This information is of questionable providence. While there are likely many leopards in Angola, there is still no hard data (Lise Hanssen pers. comm. 2015). Encouragingly, large carnivore surveys are planned for 2015/2016 (Philipp Henschel pers. comm. 2015).

Botswana

Status: Extant

Botswana hosted widespread and common leopard populations (P. Gerngross unpublished data) into the 1980's (Stuart & Wilson, 1988; Martin & de Meulenaer, 1988). Smithers (1971) noted that even in areas where the leopard was "intensively hunted," it persisted. Habitat quality was probably more important than human influence, as leopards were more common around Gaborone than in the unpopulated Kalahari Desert (Teer & Swank, 1977). For management purposes, Botswana is divided into a northern and southern zone, and further subdivided by land use. The Predator Strategy (DWNP, 2003 in CARACAL, 2006) estimated between 4,404 and 6,830 leopards in these four zones. The Northern Conservation Zone had large, stable populations of leopards and comprised the Okavango, Kwando/Chobe, Dry North, Pandamatenga, and Pans management units. The Southern Conservation Zone also had a large stable population comprised of the Central Kgalagadi Game Reserve, Kgalagadi wildlife management areas, and the Kalahari Transfrontier Park. Leopards in the Northern and Southern Agriculture Zones on the other hand were lower density and depended on some conservation management to maintain their populations.

Ongoing research has provided updated density estimates in some locations. The Shashe-Limpopo research group (2010) documented leopard presence via camera traps in the late 2000's throughout the Greater Mapungubwe Transfrontier Conservation Area. Boast and Houser (2012) estimate very low density populations in the Ghanzi commercial farmlands of 0.1 individuals per 100 km^2 with a range from 0 to 0.25 individuals per 100 km^2. Kent (2011) on the other hand provides a slightly higher density estimate in the Ghanzi region of 0.48 individuals per 100 km^2.

Winterbach et al. (2014) assessed the landscape suitability of large carnivores across Botswana. While large wild prey is more restricted to conservation areas, leopards prefer small to medium prey, which are less restricted to conservation areas. This suggests leopard may be able to persist throughout the country in conservation zones as well as agricultural areas given proper conflict mitigation strategies (Winterbach et al., 2014).

Lesotho

Status: Possibly Extinct
Last Record: 1980's (Morake, 2010)

Leopards historically occurred throughout Lesotho (P. Gerngross unpublished data) but may have become extinct by the 1980's (Arnett, 1982; Stuart & Wilson, 1988; Shoemaker, 1993). However, Morake (2010) conducted interviews with locals around the country and found that many still believe leopards were present. Locals in the Ketane, Libibing, Liphiring, Ha Kotsane regions note it is uncommon but present (Morake, 2010). The Biological Diversity of Lesotho report (2000 cited in Morake, 2010) also listed leopards as rare but present in the mountains of Lesotho. Additionally, the Field Guide to Mammals of Southern Africa, (Stuart & Stuart, 2001) suggested leopards were still present in the Quthing, Qacha's nek, Mokhotlong, and Butha-Buthe districts. Finally, just outside the eastern border of Lesotho, leopards are present in the Drakensberg Mountains of South Africa (see Friedmann & Daly, 2004). Despite the uncertainty over its continued presence,

the most recent leopard records were from killed individuals in the 1980's near Ha Sekantsi and Phamong (Morake, 2010).

Malawi

Status: Extant

Historically, the leopard occurred throughout all of Malawi (P. Gerngross unpublished data), and remained widespread even into more densely populated areas such as Lilongwe (Myers, 1976; Ansell & Dowsett, 1988; Martin & de Meulenaer, 1988). Myers (1976) noted that human-wildlife conflict with leopards was particularly problematic in Malawi. Shoemaker (1993) reported that leopards were present in Lengwe National Park and likely present in Nyika, Kasungu, and Liwonde National Parks, Vwaza Marsh, Nkhotakota, Majete, Mwabvi Game Reserves, and North Forest Reserve and Army Range, South Viphya, Chimaliro, Dwambezi, Namizumu, Managochi, Zomba, Mulanje Mountain and Thyolo forest reserves. Leopards were also likely present in Lake Malawi National Park during the park's designation in 1981 (Mésochina et al., 2010).

More recent references are less geographically comprehensive. A number of anecdotal reports do exist including a 'healthy leopard population' in Nyika National Park (Purchase, Mateke & Purchase, 2007), presence in Vwaza Marsh Wildlife (NWT, n.d.), NORAD (2009) lists the presence of leopards in the Mulanje Mountains, and ALERT documented two leopards in 2009 within Kasungu National Park (ALERT, n.d.). Additionally, the African Parks organization relocated up to eight leopards from South Africa to the Majete Wildlife Reserve in 2011 and 2012 and now list nine leopards in the park (APN, 2012). Leopards may now be absent from Liwonde National Park (Purchase, Mateke & Purchase, 2007).

Mozambique

Status: Extant

The leopard historically occurred throughout Mozambique (P. Gerngross unpublished data), likely with little change to their distribution into the 1980's (Stuart & Wilson, 1988). Leopards were common everywhere, particularly in the northern areas although they were depleted in a few other areas, such as between Beira and Cabora Bassa, and south of the Limpopo River (Myers, 1976).

However, the Mozambican Civil War, from 1977 to 1992, depleted wildlife around the country and negatively impacted carnivores such as lions (Fusari et al., 2010; Lindsey & Bento, 2011). Since the cessation of hostilities, leopards are still found in several regions although outside of protected areas, leopard presence is poorly monitored and largely unknown. Leopards persist in Limpopo (Everatt et al., 2014), Gorongosa (Purchase, Mateke & Purchase, 2007), and Banhine National Parks (Leah Andresen pers. comm. 2014), in Xonghile Game Reserve (Strampelli, 2015) and in Gilé National Reserve (Mésochina, Langa & Chardonnet, 2008). Strampelli (2015) estimated a density of ~1.5 leopards/100 km^2 in Xonghile Game Reserve. Managers estimated 10 leopards in Coutada #9 of Manica Province in 2010 (Lindsey & Bento, 2011). In northwest Tete province, interviews and human-wildlife conflict suggest continuing leopard presence (Jacobson et al., 2013).

Niassa National Reserve is the largest protected area in Mozambique, and a lion stronghold (Riggio et al., 2013) with a substantial leopard population. From camera trap data collected during 2008-2010, Agostinho (2012) estimated leopard densities in both riparian and miombo woodland sites. Densities were roughly two times higher in riparian areas (3.7 – 12.65 leopards/100 km^2) than in miombo woodland (2.18 – 4.31) (Agostinho, 2012). A comprehensive camera-trap survey was undertaken in 2014 for leopards (Niassa Carnivore Project, 2014) but the results are not yet released. Primary threats to the leopard in the area are poaching for leopard skins, snaring/trapping, and sport hunting; evidence suggests that the current levels of legal and illegal offtake are not sustainable (Niassa Carnivore Project, 2014).

In a study of the sustainability of leopard sport hunting in Mozambique's Niassa National Reserve (Jorge et al., 2013), researchers found that the reserve's leopard densities were comparable with other parts in Africa, and the status of leopard could still be regarded as secure, notwithstanding increasing anthropogenic threats. However, the Mozambican government has recently pushed to increase the quota on leopard hunting due to an increase in human-wildlife conflict, under the pretenses that leopard populations are sustainable countrywide (CITES, 2007). According to their report, leopard populations have been recovering over the preceding 10 to 15 years with the end of the civil war and demonstrated by an increase in human-wildlife conflict in many areas. They estimated a current population of around 20,000 (CITES, 2007) using a conservative adaptation of Martin and de Meulenaer's (1988) estimates. In terms of range, the government estimated that up to 80% of Mozambique was capable of supporting leopard populations, however their methodology involved using LANDSAT imagery from 1998, suggesting that current habitat conditions may be less suitable than reported.

Namibia

Status: Extant

Except for some coastal parts of the Namib Desert, the leopard historically occurred throughout Namibia (P. Gerngross unpublished data). The distribution remained largely the same from the 1930's into the 1980's (Shortridge, 1934; Stuart & Wilson, 1988). Leopards appeared more numerous in Damaraland, the Grootfontein district, the Caprivi strip and other undisturbed areas or parks while they are sparse in Kaokoveld and Namaqualand, and nearly absent in Ovamboland (Myers, 1976). Shoemaker (1993) indicated that leopards were present in Etosha, Mudumu and Mamili National Parks, Namib-Naukluft Park, the Waterburg Plateau Park, Von Bach Recreational Park, Hardap Game Reserve, Western Caprivi Game Reserve, Fish River Canyon Nature Reserve, and conservation areas in the Bushmanland and Kavango.

Hanssen and Stander (2004) collected observation data from safari guides and ranchers across Namibia to create a *Large Carnivore Atlas*, the first of its kind in Africa. They compiled 939 observations of leopards, most of which came from central and northern Namibia, and demonstrated that leopards would use almost any habitat in Namibia with the exception of the coastal desert areas (Hanssen & Stander, 2004). They estimated a total countrywide population between 5,469 and 10,610 individuals with densities ranging from 0.5 to 3.8 leopards/100 km^2 (Hanssen & Stander, 2004). The Namibian government used a conservative density of 0.82 leopards/100 km^2 from the Nyae Nyae Conservancy to extrapolate a countrywide population of 5,185 leopards (Anonymous, 2004) and set an export quota of 250 to maintain a sustainable harvest. This national quota was reached in 2010, prompting an update to the *Carnivore Atlas*.

The Ministry of Environment and Tourism (MET) updated the *Atlas* by initiating a similar large carnivore survey in 2010 (Stein et al. 2012). MET sent questionnaire surveys to farmers around the country to gather information on presence/absence and occurrence levels. This information was collated to create contour maps of leopard presence at three density levels. Stein et al. (2012) found that leopards were the most widely distributed large carnivore in Namibia, occurring across nearly 570,000 km^2 and only absent from 30% of their historic range in the country. The majority of their range, 308,586 km^2, was deemed "high density" although this could be biased due to the subjective nature of the questionnaire survey. Furthermore, they estimated a national population between 13,356 and 22,706 individuals, an increase of at least 110% from 2004. The population increase was primarily driven by an expansion of "high density" areas. Much of leopard range is outside the protected area system and therefore subject to human wildlife conflict. Indeed, negative landowner reactions resulting from leopard conflict is a primary threat to their persistence in Namibia (Stein, Andreas & Oschenborn, 2012). Poorly managed trophy hunting is an additional threat to the leopard (Stein, Andreas & Oschenborn, 2012).

Through camera trapping and telemetry studies, Stein et al. (2011) estimated about 1 leopard/100 km^2 in the Waterberg Plateau National Park compared to the 3.6 leopard/100 km^2 on the surrounding farmlands (Stein et al., 2011). This matched the higher levels of ungulate prey abundance on the surrounding farmland. Roughly 110 leopards are estimated throughout Bwabwata National Park via spoor survey (Funston et al., 2014).

South Africa

Status: Extant

The leopard historically occurred throughout South Africa (P. Gerngross unpublished data) although habitat has shrunk considerably (Henschel et al., 2008). Myers (1976) noted the leopard had virtually disappeared from the Orange Free State, the Karoo and the High Veld while the strongholds were the parks and reserves. By the 1980's leopards were completely eradicated from the central Karoo, the foothills of the Drakensberg Mountains, and most of the Northern Cape north of the Orange River due to hunting and conflict (Norton, 1989 cited in Martin & de Meulenaer, 1988). Ranges are now largely restricted to the Cape Fold Mountain ranges and South African borders with Botswana, Lesotho, Mozambique, Namibia, Swaziland, and Zimbabwe.

Leopards have been recently documented in many protected areas including: Addo Elephant Park (Hayward et al., 2009), Kalahari-Gemsbok National Park (Bothma & Bothma, 2012), Karongwe Private Game Reserve (Owen, Niemann & Slotow, 2010), Mkhuze Game Reserve (Balme, Slotow & Hunter, 2010), Phinda Game Reserve (Balme, Slotow & Hunter, 2010), Zululand Rhino Reserve (CBSG, 2005), the Greater Mapungubwe Transfrontier Conservation Area (SLPRG, 2010), and Kruger National Park (CBSG, 2005) and its surrounding reserves including Sabi Sands and Londolozi (Chase-Grey, 2011) and N'wanetsi concession (Maputla, Chimimba & Ferreira, 2013). Rogers (2008) reported the results of a survey in the Baviannskloof mountain wilderness of the Eastern Cape Province, which suggested leopard may number as few as 30 individuals throughout the 300,000 ha region.

Densities along South Africa's northeastern regions have been documented between 7-11 individuals/100 km^2 within protected areas, but only 2.5/100 km^2 outside of protected areas (Balme, Slotow & Hunter, 2010; Owen, Niemann & Slotow, 2010; Maputla, Chimimba & Ferreira, 2013). Some areas in and around Kruger National Park have by far the highest densities of leopards, estimated at 52 individuals/100 km^2 (Chase-Grey, 2011) or as many as 900 total individuals (CBSG, 2005). Leopards are also documented at high densities (nearly 20 individuals/100 km^2) in the Soutpansberg Mountains (Chase-Grey, 2011). Countrywide, the Conservation Breeding Specialist Group (CBSG) of Southern Africa (2005) referenced population estimates from a 2004 study by Friedmann and Daly (2004). Friedmann and Daly (2004) combined presence from both museum records and personal observations to derive countrywide total estimates between 2,185-6,780 leopards, broken down into several core regions.

Swanepoel et al. (2013) recently investigated the habitat suitability of leopards throughout South Africa using Maxent, a species distribution modeling software. They found that only roughly 20% of South Africa is suitable leopard habitat and that it largely conforms to existing distribution maps (Henschel et al., 2008). The habitat is severely fragmented by anthropogenic impacts and largely restricted to four general areas. They concluded that unprotected, mostly private land is extremely important for leopard conservation as only approximately 1/3rd of suitable leopard habitat overlapped with protected areas (Swanepoel et al., 2013).

Despite considerable ongoing leopard research and trophy hunting, there are no formal, national monitoring programs for leopards (Friedmann & Traylor-Holzer, 2008). Based off a population viability exercise, current trade and hunting quotas may lead to population decline although there is little risk of extinction in the Greater Kruger, North Limpopo, Western Cape and Kalahari regions (Friedmann & Traylor-Holzer, 2008). Populations in other areas of the country are at some risk of extinction due to hunting quotas including Waterberg/Mpumalanga, North KwaZulu-Natal, Orange River, and East Cape Mountain while populations in the Eastern Cape Valley and Wild Coast regions are at the greatest risk of extinction (Friedmann & Traylor-Holzer, 2008). Possibly due to the concern over population viability, South Africa has recently imposed a national, year-long hunting ban on leopards for 2016 (http://www.theguardian.com/world/2016/jan/25/south-africa-bans-leopard-hunting-2016, accessed February 2016).

Swaziland

Status: Extant

No publications regarding the recent presence of leopards in Swaziland were collected during this literature review although the leopard historically occurred throughout the country (P. Gerngross unpublished data). Shoemaker (1993) was also unable to gather any recent data related to leopard status in Swaziland. It is possible leopards were extinct by the 1970's (Eaton, 1977). However, leopards may have persisted in the lowveld and mountainous north into the 1980's (Stuart & Wilson, 1988). Given the high densities of leopards bordering Swaziland in South Africa (e.g., Kruger National Park), it is possible that transient individuals use regions of Swaziland or that leopards could have recovered naturally from previous persecution.

Zambia

Status: Extant

Historically leopards occurred throughout Zambia including the montane areas (Ansell 1978; P. Gerngross unpublished data). They remained widespread and abundant into the 1970's, with continued presence even near settlements (Ansell, 1978). The Luangwa Valley, Kafue National Park, and Lower Zambezi National Park were noted for their high abundance (Ansell, 1978; Martin & de Meulenaer, 1988). Myers (1976) estimated that just the protected areas alone in Zambia could hold 10,000 to 20,000 leopards.

The Zambia Wildlife Authority and a number of researchers have continued to assess leopard presence and abundance throughout Zambia. Purchase, Mateke & Purchase (2007) asserted that leopards were present in several national parks throughout Zambia, including the Lower Zambezi, Kafue, South and North Luangwa, and Sioma Ngwezi National Parks. Elias Rosenblatt, member of the Zambian Carnivore Programme, contacted a number of researchers and park authorities regarding leopard presence in 2015 on behalf of this review. Leopards were confirmed present in a few additional National Parks, including Kasanka, Lavushi Manda, Luambe, Lukusuzi, and Nsumbu, although they were likely extirpated in Isangano (Elias Rosenblatt pers. comm. 2015). In addition, ongoing existence in Liuwa Plains National Park is doubtful, as leopards have not been documented over the last four years despite intensive monitoring; if they are present they are transient or at very low densities (Elias Rosenblatt pers. comm. 2015). Puchase, Mateke & Purchase (2007) also suggested that leopards are present in some of the Game Management Area's (GMAs) surrounding these parks, including West Petauke, Chiawa, Rufunsa, Kasonso-Busanga, and West Zambezi. Rosenblatt confirmed presence in Chiawa, but not the other GMAs that Purchase et al. (2007) mentioned. However, Rosenblatt confirmed ongoing presence in Kafinda, Lumimba, Lupande, Mukungule, Munyamadzi, Mupunda (a proposed GMA between Luvushi Manda and South Luangwa National Parks), Musalangu, Sandwe, and Tondwa GMAs. Although needing additional verification, leopards were possibly extirpated from Chambeshi, Kaputa, Luwingu, and Mansa GMAs (Elias Rosenblatt pers. comm. 2015). Midlane (2014) documented leopards in Kafue National Park via spoor and call-in surveys. Ray (2011) estimated leopard densities of 3-5 individuals/100 km^2 in Luambe National Park and Game Management Area. Density estimates from South Luangwa and Kafue are in preparation and have not yet been published (Elias Rosenblatt pers. comm. 2015).

Zambia had a ban on leopard hunting for 2013 and 2014, but has recently reinstated leopard hunting for the 2015-2016 year (http://www.theguardian.com/world/2015/may/19/zambia-to-lift-ban-hunting-lions-leopards-big-cats, accessed December, 2015).

Zimbabwe

Status: Extant

Leopards were historically widely distributed throughout Zimbabwe (P. Gerngross unpublished data) and even in settled areas (Teer & Swank, 1977). They were considered common in the Zambezi and Limpopo valleys, throughout the eastern districts, Motobo National Park, in the northern sector of Hwange National Park, and in the Gwanda/West Nicholson area in the southwest (Stuart & Wilson, 1988). Leopard population

estimates varied widely, from 2,288 (Eaton, 1977) to 38,000 individuals (Child, 1985 cited in Shoemaker, 1993). However, Wilson argued that 10,000 was closer to the truth by the late 1980's (Shoemaker, 1993). Leopards were known or likely present in 25 conservation areas (Shoemaker, 1993). However, land redistribution led to decreased wildlife and predator densities (Degeorges & Reilly, 2007). No detailed assessment of the actual leopard population in Zimbabwe exists (Wilson, 2006) although they persist in several conservation areas including: Hwange, Zambezi, Matusadona, and Mana Pools National Parks, and Matetsi, Chirisa, Chete, Charara, Hurungwe, Chewore, Doma, and Umfurundzi Safari Areas (Purchase, Mateke & Purchase, 2007).

There are several population density estimates from different regions of Zimbabwe. A combination of spoor surveys and camera traps were used in 2010 to assess the leopard population in a 200 km^2 area in the Mangwe District (Grant, 2012). Grant (2012) estimated a population of roughly 19 leopards, with densities of around 5 individuals/100 km^2. Groom & Brand (2011) reported densities averaging 8.3 individuals/100 km^2 for an estimate of 315 individuals (split into south and north) in Gonarezhou National Park based on a spoor survey in 2009. Williams (2011) estimated 193 leopards in Save Valley Conservancy based off densities of 7.6 individuals/100 km^2 using spoor surveys in 2008. Additionally, 54 leopards are estimated via camera trap survey in the Northern Tuli Game Reserve at a density of roughly 7.5 individuals/100 km^2 (Shashe-Limpopo Predator Research Group, 2010)

Asia

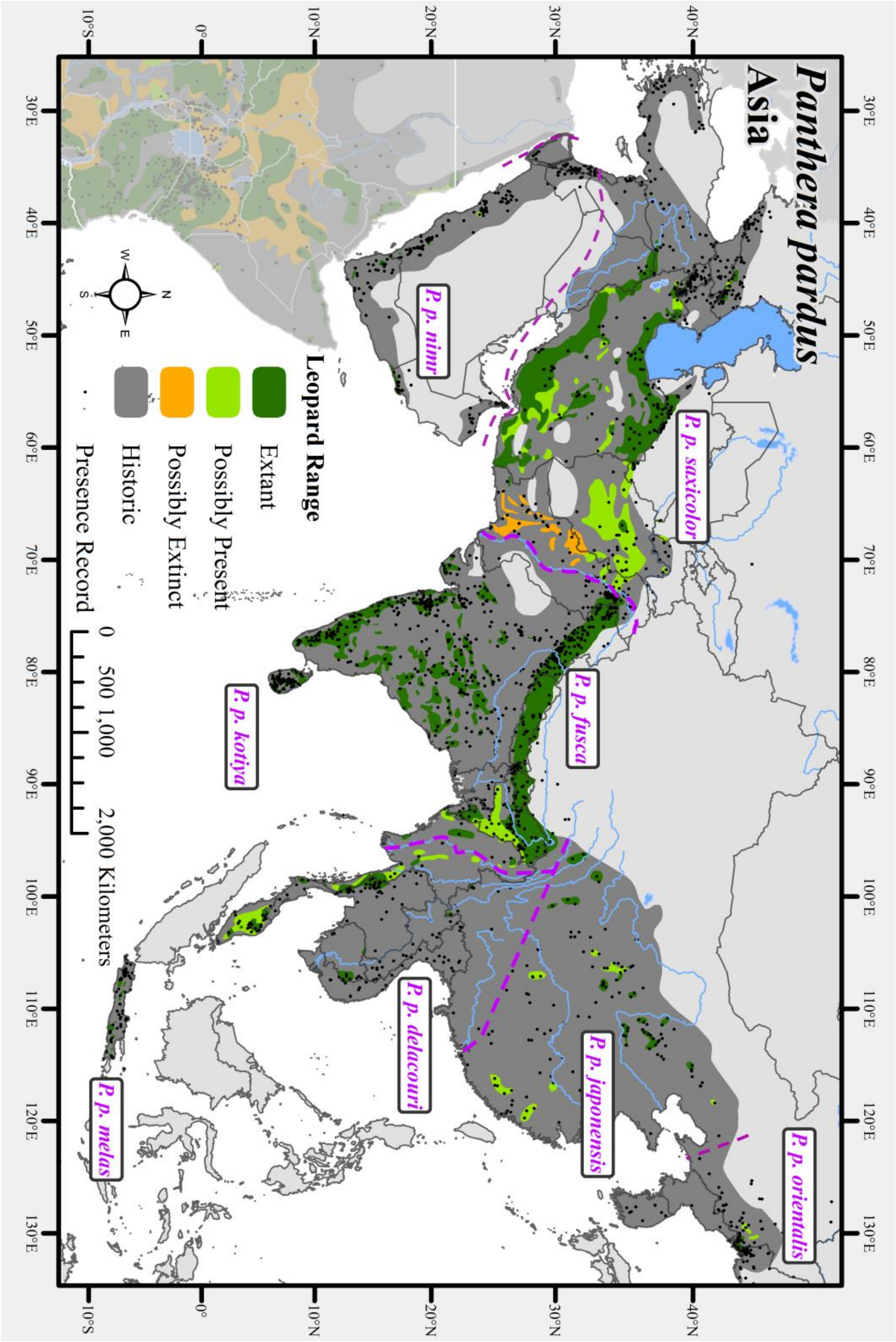
Panthera pardus
Asia
P. p. nimr
P. p. saxicolor
P. p. fusca
P. p. kotiya
P. p. delacouri
P. p. japonensis
P. p. orientalis
P. p. melas
Leopard Range
Extant
Possibly Present
Possibly Extinct
Historic
Presence Record
0 500 1,000 2,000 Kilometers
N
S
E
W
10°S
0°
10°N
20°N
30°N
40°N
30°E
40°E
50°E
60°E
70°E
80°E
90°E
100°E
110°E
120°E
130°E

Arabian Leopard

Panthera pardus nimr

Middle East

Listed as Critically Endangered by the IUCN since 1996 in recognition of its small and fragmented population, the Arabian leopard is a subspecies endemic to the Arabian Peninsula. At a regional workshop in 2007 facilitated by the IUCN/SSC Cat Specialist Group, the participants agreed to a long-term vision to have "viable and sustainably managed populations of the Arabian leopard, its wild prey and natural habitats in co-existence with local communities across its range in the Arabian Peninsula" (Breitenmoser et al., 2010). Participants agreed to regional conservation targets and activities with some processes delegated to National Action Plans. The workshop identified 11 objectives, including more survey and monitoring activities, effectively managing a network of protected areas that safeguard leopard habitat and corridors, and ensuring support from local communities and stakeholders (Breitenmoser et al., 2010). By 2015, Oman and Saudi Arabia have adopted National Action Plans.

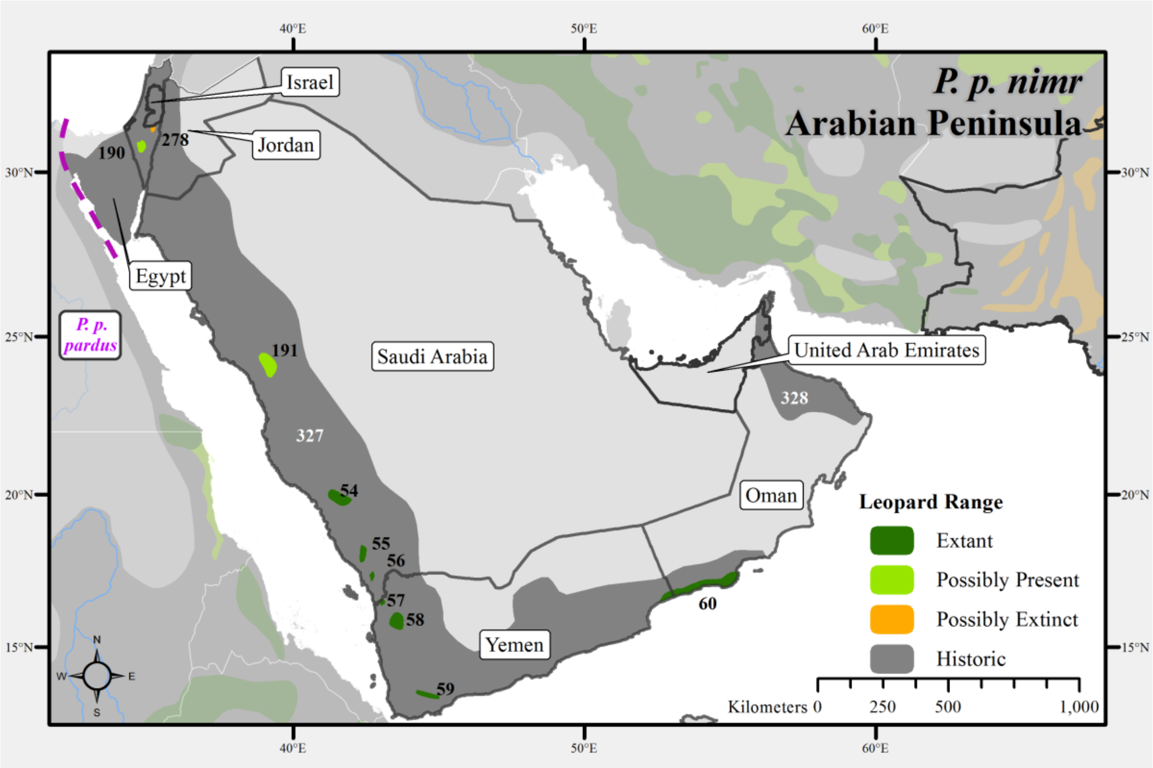

Egypt (Sinai Peninsula)

Status: Extinct
Last Record: 1997 (Spalton & al Hikmani 2006)

Historically, the Arabian leopard occurred throughout the Sinai Peninsula probably excluding the North Sinai Desert along the Mediterranean coast (P. Gerngross unpublished data). Through historical time, the climate warmed and dried affecting the ecological community of the Sinai (Yeakel et al., 2014). Overhunting was the primary threat to wildlife but overgrazing, and deforestation were important contributors in driving many species to extinction (Mendelssohn, 1989; Manlius, 2001). By the late 1960's leopards were already thought extinct (Mendelssohn 1989). However, in 1995, Saleh et al. (1995) reported the presence of leopard tracks in Wadi El Omiyed, ~5 km inland from the Gulf of Aqaba, and an adult leopard was caught in a leg-hold trap along the western border of the Abu Gallum protected area (Spalton & al Hikmani, 2006). Additional sightings of leopards reported in 1997 initiated a long-term camera-trapping project to detect further leopard presence in the Sinai Peninsula (Spalton & Al Hikmani, 2006). No additional evidence of leopard occupancy has been reported, and the leopard is likely extinct in the Sinai Peninsula (Perez, Geffen & Mokady, 2006; Spalton & al Hikmani, 2014).

Israel

Status: Possibly Present
Last Record: 2000 (Perez, Geffen & Mokady, 2006)

The leopard historically occurred throughout Israel as evidenced by the frequent use of the word "Namer" for locations as well as numerous Biblical references (Tristam, 1884 cited in Qumsiyeh, 1996; P. Gerngross unpublished data). Already rare at the end of the 19th Century, the leopard was nearly extirpated in the first half of the 20th Century due to continued habitat loss and hunting (Qumsiyeh, 1996). After 1960, Qumsiyeh (1996) compiled only a handful of leopard records concentrated in the far north near Lebanon, near the Dead Sea, and scattered in the Negev Highlands. Hunting, although this became illegal in the 1960's, low breeding success, accidents, and management errors contributed to population declines during the late 20th Century (Timna, 2000 cited in Perez, Geffen & Mokady, 2006). Fecal samples collected by Perez, Geffen & Mokady (2006) during field surveys in the early 2000's indicated a few individuals each in the Judean Desert and the Negev Highlands. However, a small countrywide population and long-standing isolation of leopards in Israel has increased the rate of inbreeding, which complicated the use of molecular markers for individual analyses (Perez, Geffen & Mokady, 2006). Since then, there has been no research on leopards due to lack of funding. There have been no confirmed sightings of leopards anywhere outside the Negev Highlands since the early 2000's and leopards in the Judean Desert, as well as elsewhere, are likely extinct (Eli Geffen and Yehoshua Shkedy pers. comm. 2015). Khalaf-von Jaffa (2002) referenced occasional reports of leopard in the Golan Heights region, possibly being connected with a population on Mount Hermon, Lebanon, (which may be *P. p. saxicolor*) but confirmation of leopard persisting in this region is lacking. Unconfirmed reports in the West Bank continue, the most recent on the banks of the Jordan River in 2014 (Eli Geffen and Andrew Spalton pers. comm. 2015). No more than a few individuals likely persist within the entire country.

Jordan

Status: Extinct
Last Record: 1987 (Qumsiyeh, Amr & Shafei, 1993)

Currently extinct, the leopard historically occurred throughout western and southern Jordan (P. Gerngross unpublished data). Most records of leopard in Jordan pre-date 1980, and are predominately reports of leopards being shot (Qarqaz & Baker, 2006). Timna (2000) estimated no more than eight leopards in Jordan in the 1970's. In February 1987, shepherds observed a leopard attack and kill two sheep near Tafilah. This was the last confirmed record in Jordan (Amr & Disi, 1988). Unconfirmed reports of leopards have been made since 1987; however, research efforts have not found sufficient evidence to substantiate the anecdotal reports (Breitenmoser et al., 2010). For instance, Timna (2000) reference two "known observations" in the late 1990's, and there was an unconfirmed report of a leopard on the banks of the Jordan River at Bethany beyond the Jordan in 2014 (Andrew Spalton pers. comm. 2015).

Qarqaz and Baker (2006) and Breitenmoser et al. (2010) identified the Dana Reserve near Tafilah as suitable habitat for the leopard that could be recolonized by leopards from Israel or, less likely, Saudi Arabia. Jordan has established 1300 km^2 of protected areas (Mazzolli, 2008), which could act as potential habitat.

Kuwait

Status: Outside Historical Range

Kuwait is out of the historical range of leopards (Harrison & Bates, 1991; P. Gerngross unpublished data).

Oman

Status: Extant

Historically, the Arabian leopard occurred throughout two discontinuous areas in Oman (Spalton et al., 2006a; P. Gerngross unpublished data). In the north, the leopard was likely widespread along the Hajar mountain range from the Musandam peninsula in the west to the Eastern Hajar range near Sur (Spalton et al., 2006a). In the south, the leopard occurred in the Dhofar Mountains and along coastal Oman in the southwest bordering Yemen. Currently the leopard is believed to be extinct in the north and restricted to the Dhofar Governorate in southern Oman (Spalton & al Hikmani, 2014). The total leopard population in Oman is estimated between 44 and 58 individuals (Spalton & al Hikmani, 2014). Up to 30 of these individuals are estimated to inhabit the Jabal Samhan Nature Reserve. Prior estimates including results from a camera trap survey from 1997-2000, suggested a breeding population of at least 17 leopards in the Jabal Samhan Nature Reserve, and an additional 9-11 leopards in Jabal Qara and Jabal Qamar extending west of Jabal Samham into Yemen (Spalton et al., 2006b). Recent camera trapping in 2014 captured the leopard in the Nejd, or the north-facing valleys of the Dhofar Mountains, representing potential range expansion into historical habitats (Al-Hikmani et al., 2015).

In the north, leopards are believed extinct although unconfirmed reports continue (Spalton & al Hikmani, 2014). The last leopard seen on the Musandam Peninsula was in 1997 following 30 years of persistent and indiscriminate persecution by local shepherds (Spalton et al., 2006a). However, Jongbloed (2001) has reported persecution of leopards as recent as 2001 in neighboring United Arab Emirates. Breitenmoser et al. (2010) reported leopards in adjacent mountain ranges of UAE and the Musandam Peninsula dating from the 1990's and 2000's, in addition to leopard tracks identified in the Musandam Peninsula in 2007. A dead leopard reported in 1976 near Rustak was the last confirmed presence of leopards in the Hajar mountain range (Spalton et al., 2006a).

Saudi Arabia

Status: Extant

Historically, the Arabian leopard occurred along the mountains bordering the Red Sea coast in western Saudi Arabia (P. Gerngross unpublished data) although Judas et al. (2006) believe it may have occurred farther inland, possibly as far east as the Riyadh region. Regardless, the leopard's range has shrunk considerably, possibly by 90% since the beginning of the 19th century (Judas et al., 2006). Habitat fragmentation and degradation, and direct persecution are the primary threats (Judas et al., 2006; Zafar-ul Islam et al., 2014). Judas et al. (2006) used confirmed reports of leopards from four locations to assess viable habitat range. They estimated 29,724 km^2 of viable habitat, capable of supporting 60-425 leopards in the Asir and Hijaz mountain ranges on the basis of previous studies of leopard distribution in regions of Africa with comparable climate (Judas et al. 2006). However, many recent camera trapping efforts have failed at confirming presence (Judas et al., 2006; Al-Johany, 2007; Mallon & Budd, 2011). In fact, most confirmed records have been of deceased individuals such as two leopards near the Yemen border in 1999, two poisoned individuals in 2007 near an-Namas in the Asir Mountains (Mallon & Budd, 2011), and poisoned adult males in Bani Saad in 2011, and Wadi Nauman in 2014 (Zafar-ul Islam et al., 2014). The most recent population estimate speculated less than 100 individuals remain in the wild (Boug & Zafar-ul Islam, 2009) although even this may be generous (Andrew Spalton pers. comm. 2015).

United Arab Emirates (UAE)

Status: Extinct
Last Record: 2007 (Reinl, 2007)

Historically, the Arabian leopard occurred in the Hajar Mountains on the Musandam Peninsula spanning into Oman (Harrison & Bates, 1991; P. Gerngross unpublished data), but they are likely extinct in the Musandam and UAE (Breitenmoser et al., 2010; Mallon & Budd, 2011; Spalton & al Hikmani, 2014). From 1986-1997 seven leopards were killed in the UAE (Edmonds et al., 2006). A population assessment workshop hosted by the IUCN Conservation Breeding Specialist Group in 2000 estimated the population in northern UAE and Musandam to consist of only 5-10 individuals (Edmonds et al., 2006). The same workshop identified the UAE as a possible corridor between the Musandam Peninsula and the Hajar Mountains, however, with the extinction of the leopard in the Hajar range, Edmonds et al. (2006) suspected this region would not be utilized. Breitenmoser et al. (2010) mentioned possible leopard reports in the 1990's and 2000's, including "field signs" in 2006, and tracks in Musandam in 2007.

Yemen

Status: Extant

The Arabian leopard is thought to have historically occurred throughout the mountainous areas of Yemen, stretching from Saudi Arabia in the northwest southwards along the western mountains, and also in southern Yemen east to the border with Oman (Harrison & Bates 1991; Al Jumaily et al., 2006; P. Gerngross unpublished data). It was likely absent in the desert regions of Al Jouf, Marib, and Shabwa (P. Gerngross unpublished data). The distribution and population has shrunk considerably owing to direct persecution and prey base depletion (Al Jumaily et al., 2006).

Al-Jumaily et al. (2006) compiled reports of leopard presence in Yemen from post-1990 and identified five regions of particular interest, but given the varied and dated nature of the reports also cautioned their use. The five sites include the central-western highlands (Al Hayma, Jebel Raymah, Jebel Bura'a), near Wada'a and Hajja, southwest Yemen (between Radfan and Al Kaur and extending northwest near Ta'iz), central-southern Yemen (Wadi Hajar and Hadhramaut), and Hawf forest in Al Mahra. Since those five regions were identified, presence has been confirmed in two, Hawf, as well as Wada'a and Hajja (Mallon & Budd, 2011). DNA barcoding revealed a scat recovered from Hajja in western Yemen in 2009 as leopard (Mallon & Budd, 2011). A trapped leopard confirmed continued presence near Wada'a along the northwestern border with Saudi Arabia, and the population is thought to potentially connect through to the Sarawat Mountains in Saudi Arabia (Al-Jumaily et al., 2006). Camera traps placed by the Foundation for the Protection of the Arabian Leopard captured two individuals in the Hawf Forest protected area, a mountainous region bordering Oman, over four months in 2011 (Mallon & Budd, 2011). Wadi Masilah has not been surveyed for leopard presence (as far as the authors know), but is noted to provide access to flowing water and areas to the north of Hadhramaut reportedly support ibex (Al-Jumaily et al., 2006). Spalton & al Hikmani (2014) suggested a few governorates that may still contain leopards including: Hajjah and 'Amran (north of Sana'a), Lahij, Abyan and Al Bayda' (in the south), and the Mahra governorate (on the border with Oman).

Persian Leopard

Panthera pardus saxicolor

Southwest Asia

The Persian leopard, endemic to southwest Asia, has been listed as Endangered by the IUCN since 1996 in recognition of its greatly reduced distribution and population size. At a regional workshop in 2007 hosted by the WWF Caucasus Programme Office, the participants agreed to a long-term vision: "leopards and all wildlife prosper in natural habitats across the Caucasus ecoregion in harmony with people" (Breitenmoser et al., 2007). After a compilation of current knowledge, the participants agreed to conservation targets and activities, and were encouraged by the IUCN/SSC Cat Specialist Group to implement the regional conservation strategy via adoption of National Action Plans. By 2015, three countries have adopted Action Plans, Armenia, Azerbaijan, and Georgia.

Two regional distribution modeling approaches have identified suitable leopard habitat in the Caucasus (Zimmermann et al., 2007) and across Persian leopard range (Gavashelishvili & Lukarevskiy, 2008). The first, a more simplified approach, identified roughly 120,000 km^2 of suitable leopard habitat in 12 clusters across the Caucasus ecoregion. Based off low to moderate leopard densities of 0.5-1 leopard/100 km^2, the potential total regional population could be between 600-1200, with ~1/3rd in the Greater and 2/3rds in the Lesser Caucasus (Zimmermann et al., 2007). However, only about 9,250 km^2 were known occupied with a total population of ~65 individuals (Zimmermann et al., 2007). A more complicated modeling approach across southwest Asia and northern Middle East, concluded that leopards were found in or near dry, rugged terrain, and positively correlated with vegetation productivity (Gavashelishvili & Lukarevskiy, 2008). Results also suggested that leopards avoided deserts, areas with long-duration snow cover, and urban development. Finally, they predicted overall distribution of the leopard throughout the area, and identified potential habitat corridors (Gavashelishvili & Lukarevskiy, 2008).

Due to the transboundary nature of leopards in the Caucasus ecoregion, we briefly give some regional comments before continuing with Country Profiles. Askerov et al. (2015) assert there are four separate transboundary populations, the Talysh Mountains in southern Azerbaijan (and connecting to the Alborz Mountains), Nakhchivan/Azerbaijan/southern Armenia, the Iori-Ajinour Plateau (eastern Georgia and northwest Azerbaijan), and the eastern Greater Caucasus (Georgia, and Daghestan/Russia). Recent monitoring has shown limited presence in the eastern Greater Caucasus and Iori-Ajinour Plateau, but a persistent population in the other two populations (Askerov et al., 2015), although there was a February 2015, sighting in Daghestan, Russia (Y. Yarovenko and N. Zazanashvili unpublished data). See Breitenmoser et al. (2014) for detailed and coordinated information on leopard presence, status, and conservation actions of range countries.

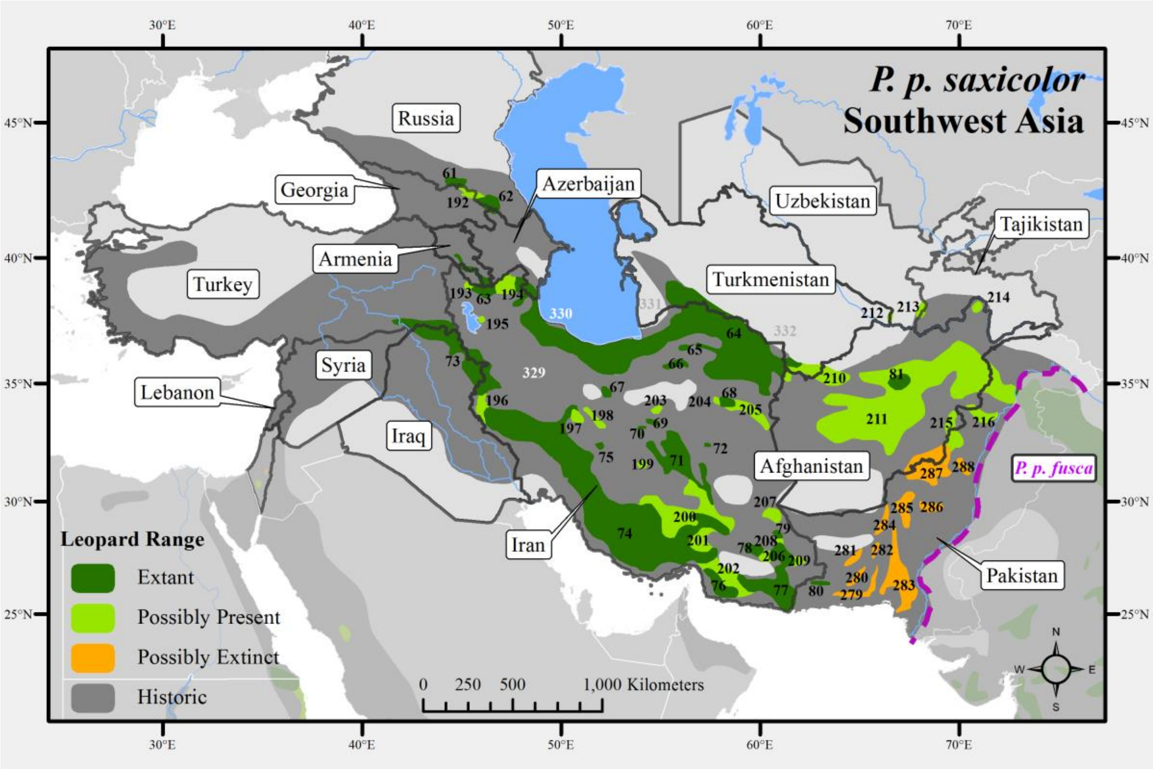

Afghanistan

Status: Extant

Historically, the Persian leopard occurred throughout most of the country but was most abundant in montane forests and subalpine regions (Shoemaker, 1993; P. Gerngross unpublished data). In general however, distribution and population records of leopards in Afghanistan are lacking. With almost no information, 30 plus years of military conflict, little to no wildlife law enforcement, and depressed prey populations, it is likely that leopards remain only at very low numbers and under significant threat (Moheb & Bradfield, 2014). Habibi (2004) estimated 200-300 leopards in Afghanistan before the Soviet invasion in 1979.

An expanding human population with demand for skins and furs, coupled with prolonged civil unrest and increased access to weaponry have decimated wildlife populations in Afghanistan and surrounding countries (Johnson & Wingard, 2010). Surveys of fur shops in the Kabul market conducted by Mishra and Fitzherbert (2004) revealed leopard as one of the five most commonly targeted species on the market. Formerly, skins were sold openly and suspected to have originated from Herat and Badakshan (Shoemaker, 1993). From 1971-1976, a total of 1,738 leopard and snow leopard (*Panthera uncia*) skins were exported from Afghanistan (Shoemaker, 1993). More recent survey efforts have shown a substantial decrease in the number of skins being traded with vendors selling 3-10 leopard skins per shop per year (Johnson & Wingard, 2010). This reduction is possibly due to decreased abundance of leopards but also could be from increasingly wary shop owners (Johnson & Wingard, 2010). Complicating matters, traders suggest the pelts have numerous origins (Manati, 2009). Live animals are sometimes captured as well; Husain (2001) found two male leopard cubs in Pakistan, captured from mountains near the Afghan-Pakistan border.

Currently, there is little known beyond the general assumption that leopards are present but declining in Afghanistan. Stevens et al. (2011) attempted to capture leopards in eastern Afghanistan using cameras in 2007 and 2009, but to no avail despite reported "evidence" of leopards from survey teams and residents. While leopards were commonly blamed for livestock loss in Nuristan, photographic evidence and DNA barcoding dismissed these claims (Stevens et al., 2011). In 2011, Moheb and Bradfield (2014) captured a leopard on camera in the Yakawlang District of Bamyan Province. Also in Yakawlang District, a team from the Wildlife Conservation Society observed a female with three cubs in August 2015 (Nasart Jahet and Stephane Ostrowski pers. comm. 2016). Moheb and Bradfield (2014) also reference two other locations where interviews with local residents reported potential presence of leopard: Darwaz in Badakhshan Province, and on the border of Nuristan and Kunar Provinces.

Armenia

Status: Extant

Landlocked and mountainous, the Persian leopard was historically found throughout all of Armenia (P. Gerngross unpublished data). Its distribution has shrunk considerably and has been restricted only to the far south of the country since the mid-1960s (Heptner & Sludskii, 1992; Khorozyan, 1999). There has not been confirmed presence of leopards in the northern part of Armenia since 1970 (Heptner & Sludskii, 1992; Khorozyan, 1999). Lukarevsky, Askerov & Hazaryan (2004) documented spoor, scat, and scrapes as evidence of leopard presence in the Lesser Caucasus. On the basis of their findings, they estimated 18-23 leopards in the Lesser Caucasus. Within Armenia, Lukarevsky, Askerov & Hazaryan (2004) estimated 2-3 leopards occupy the Khosrov Nature Reserve and an additional 3-5 leopards in the Bargushat Mountains. Khorozyan, Malkhasyan & Abramov (2008) estimate leopard density at 0.34 individuals/100 km^2 in the Nuvadi area of Meghri Ridge, southeastern Armenia based off field surveys in 2006-2007. This is one of the smallest density estimates ever documented (Stein & Hayssen, 2013). This compares with a potential density of ~7 individuals/100 km^2 based off ungulate prey biomass. The reduced actual densities are attributed to poaching, wild fire, and human disturbance of habitat (Khorozyan, Malkhasyan & Abramov, 2008). Ongoing military conflict and training are also a likely threat (Breitenmoser et al., 2007). Over 1,200 km were surveyed for leopard presence between 2004 and 2006 across 16 areas in southern Armenia; Khorozyan et al. (2010) only found leopard presence in five of them: Khosrov Reserve, the Nuvadi area, and to a lesser extent, in the Meghri Ridge, Sisian and Ajubaj areas (Khorozyan et al., 2010). Leopard presence was correlated with dry mountain grassland, sparse forests, and rugged terrain (Khorozyan et al., 2010).

Except for the Khosrov Reserve of central Armenia, the majority of leopard range lies in the south of the country, highlighting the continued dependence of leopards in Armenia on the immigration of leopards from Iran via the Zangezur Ridge (Heptner and Sludskii, 1992; Johnson, 2003; Breitenmoser et al., 2007; T-PVS, 2010; Farhadinia et al., 2015). Areas in southern Armenia bordering Azerbaijan and Iran are highlighted as important areas for leopard conservation as leopards likely move between the countries (T-PVS, 2010; Askerov et al., 2015). Recent observations from ongoing research have revealed one male and two females in Arevik National Park, Syunik province (Breitenmoser et al., 2014), suggesting that leopards may be more common than previously thought (Nugzar Zazanashvili pers. comm. 2015).

Azerbaijan

Status: Extant

The Persian leopard was historically found throughout Azerbaijan (Shoemaker, 1993), though may not have occurred in parts of the Kura Lowland (P. Gerngross unpublished data). By the mid-1960's the leopard was extirpated from the majority of its historical range in the country, but persisted in southern regions and one or two animals were killed on an annual basis (Heptner & Sludskii, 1992). Currently, the leopard is restricted to the far south of the country although it probably persists along the northern border with Georgia (Breitenmoser et al., 2007). Important ongoing threats include poaching, prey depletion, and international military conflict and training (Breitenmoser et al., 2007). Furthermore, isolation from source populations in Iran may threaten continued population viability (Farhadinia et al., 2015).

Ongoing research has recorded numerous leopard sightings within two main landscapes, the Nakhchivan Autonomous Region and the Talysh Mountains in the southeast (Breitenmoser et al., 2007; Askerov et al., 2015). Both populations are well connected with the larger Iranian population (Breitenmoser et al., 2007; Askerov et al., 2015). Indeed, a recent study confirmed the importance of southern Azerbaijan as an important habitat corridor from Iran to the Lesser Caucasus (Farhadinia et al., 2015). Between 2012 and 2014, 41 photos and 8 videos were taken of leopards in Hyrkan National Park, of the Talysh Mountains (Breitenmoser et al., 2014). In that same time period, 170 photos and 20 videos of leopard were captured in Zangezur National Park, in Nakchivan. Three individuals were identified and found to move into Armenia as well (Breitenmoser et al., 2014). Lukarevsky et al. (2007) found signs of 3-5 individuals in the Talysh Mountains, 3-4 individuals in the Zangezur Range in Nakhchivan, and 3-4 individuals in the western part of the Karabakh and Murov-Dagh ranges. Presently, it is estimated that the "Zangezur-Kiamaky" population harbors 7-10 adult leopards (Askerov et al., 2015).

Leopard conservation in Azerbaijan has developed primarily through the extension of protected areas; a total of 161,340 km^2 has been added to the network since 2004 (CLWG, 2014), more than double the area under protection in 2000 (T-PSV 2010). Frequent sightings near the Iranian border in the Talysh Mountains and in the Nakhchivan region in 2013 may suggest that leopards continue to emigrate from Iran and contribute to population viability (Askerov et al., 2015). Leopard persistence in the broader Lesser Caucasus is strongly dependent on transboundary populations and connections (Farhadinia et al., 2015).

Georgia

Status: Possibly Present
Last Record: 2008 (T-PVS, 2010)

Historically occurring throughout Georgia (P. Gerngross unpublished data), the only remaining potential leopard range is in the Greater Caucasus range along the border with Russia and Azerbaijan. Arabuli (2006) reported only 12 instances of leopard occurrences over the course of the 20th Century. The last confirmed sighting was of a frequently recorded individual, "Noah," in Vashlovani National Park in 2008 although unconfirmed reports continue from both the northern border with Russia and the southern border with Azerbaijan (Breitenmoser et al., 2014). Recent camera-trapping efforts beginning in 2009 in the Eastern Greater Caucasus near Tusheti have not yielded photographs of leopards despite evidence of "leopard signs" and local reports (T-PVS, 2010; Breitenmoser et al., 2014). With the success of World Wildlife Fund's camera-trapping programs in neighboring countries (Breitenmoser et al., 2007), the failure to detect a

resident population of leopards in the Tusheti and Khevsureti areas may indicate the leopard has only a transient presence in Georgia, as was likely the scenario in Vashlovani (Khorozyan, Malkhasyan & Asmaryan, 2005). Any remaining leopards in Georgia may be transboundary individuals (Breitenmoser et al., 2007; T-PSV, 2010), or could be remnants of a small population centered on the Eastern and Central Greater Caucasus (Nugzar Zazanashvili pers. comm. 2015). While no widely accepted population figure is given (T-PVS, 2010), Nugzar Zazanashvili (pers. comm. 2013) estimates a population of 3-4 leopards within Georgia based on "indirect" signs.

Iran

Status: Extant

Except for desert regions such as the Desht-e-Kevir and Desht-e-Lut in central and eastern Iran, the leopard historically ranged throughout most of Iran (P. Gerngross unpublished data). Iran supports the largest population of Persian leopards estimated to number between 550-850 animals, with an estimated 55% living within protected areas (Kiabi et al., 2002; Khorozyan, 2008b). Despite supporting the largest national population of the Persian leopard, leopards in Iran occur at low densities of approximately 0.06 – 0.1 individuals/100 km^2 (Kiabi et al., 2002), although leopards within well-protected reserves can reach higher densities. For example, leopards in Bamu National Park were estimated at 1.87 individuals/100 km^2 (Ghoddousi et al., 2010), 2.6 individuals/100 km^2 in Golestan National Park (Hamidi et al., 2014), and 1.5 individuals/100 km^2 in Bafq Protected Area (M. Farhadinia unpublished data). The Iranian population acts as a source population for neighboring countries (Aghili, 2005; Breitenmoser et al., 2011; Farhadinia et al., 2015). Indeed, except Turkmenistan where leopard breeding has been detected near Iran's border (Kaczensky & Linnell, 2015) as well as Afghanistan (Husain, 2001), no reproduction beyond Iran in the Caucasus region and northern Iraq has been documented since the late 1990's, whereas breeding populations are known from the Lesser Caucasus Mountains in Iran, and the Alborz Mountains south of the Caspian (Farhadinia et al., 2015). The Persian leopard is officially protected by law in Iran and occurs throughout the country; nevertheless, to safeguard persistence of the subspecies, trans-boundary partnerships between Iran and neighboring countries are a priority.

Overall, the Iranian leopard population is composed of several large discontinuous habitat patches. Sanei and Zakaria (2011) conducted a series of rapid surveys across 138 sites and detected leopards at 74 protected and non-protected areas. Detection was based on questionnaires detailing direct observations, secondary sign (scat and spoor), camera trap records, and local knowledge. Detection was highest (69% of detected sites) in northern Iran (Sanei & Zakaria, 2011). However, the habitat patches are threatened by fragmentation (A. Sanei unpublished data). Indeed, the primary threats to leopards are poaching, mainly due to retaliatory killings, and environmental degradation inside protected areas (Farhadinia et al., 2007; Khorozyan, 2008b). The collapse of the leopard's natural prey due to poaching has led to increased human-leopard conflict in various parts of the country, as leopards are persecuted in response to livestock depredation (Abdoli et al., 2008, Farhadinia et al., 2009; Ghadirian & Ghasemi, 2012; Pishvaei, 2014).

In the Caucasus ecoregion of northwestern Iran, there are two main leopard landscapes, the Alborz and "Boundary" (in East Azerbaijan province) landscapes. These regions are connected only through southern Azerbaijan (Farhadinia et al., 2015). Even within the Iranian range, which is supposed to support the viability of leopards throughout the Lesser Caucasus (Breitenmoser et al., 2010), a recent investigation revealed that most of the reserves are poorly able to support viable leopard populations (Moqanaki et al., 2013). This is more pessimistic than previously thought (Lukarevskiy et al., 2004; Aghili, 2005). Breitenmoser et al. (2014)

gathered 36 confirmed records of leopard presence in these regions between 2007 and 2014. Furthermore, human-leopard conflict in several locations threatens the viability of leopards in northwestern Iran (Moqanaki et al., 2013; Babrgir, Farhadinia, and Moqanaki, 2015; M. R. Masoud pers. comm. 2015), and leopards throughout the Lesser Caucasus need urgent conservation action.

North and northeastern Iran host a number of well-protected reserves with fair numbers of Persian leopard, from the Alborz to the Khorasan Mountains adjoining Kopet-Dagh in Turkmenistan (Kiabi et al., 2002; Ziaie, 2008). Leopard distribution in central Iran is patchy, only confirmed from a number of reserves such as Bafq, Abbas Abad, Naybandan, Darband-e-Ravar, Kavir, and Touran (Hunter et al., 2007; Farhadinia et al., 2008; Ziaie, 2008). Farther to the south, leopards are also recently confirmed from different locations mostly along the northern mountainous ranges of the Persian Gulf and Sea of Oman (Abdoli et al., 2008; Ghadirian & Ghasemi, 2012).

Human-wildlife conflict is a continuing and serious threat outside protected areas (Farhadinia et al., 2007; 2009; 2015b) and at the peripheries of national parks (Khorozyan et al., 2015). Yet, leopards predominantly predate on wild ungulates within reserves (Farhadinia et al., 2014; Ghoddousi et al., 2015).

Iraq

Status: Extant

Historically, the Persian leopard occurred in regions of Iraq north of the Euphrates River (P. Gerngross unpublished data). Currently, its distribution is limited to the mountainous border region with Iran and Turkey. Records of leopard occurrences in Iraq are limited. Hatt (1959) and Harrison (1968) compiled a handful of records of leopards in Bira Kipra near Aqra and Rowanduz, Kurdistan, Harir Dagh in the mountains of Erbil and Mosul, Kut al Imara along the Tigris River, and Jebel Baradust Al Emerijje, near Rawa along the Euphrates River.

In 2011, Nature Iraq conducted field surveys to document the status of wild goats in Qara Dagh, a proposed protected area in the Zagros Mountain Forest Steppe. Among the wildlife captured was a photograph of the left flank of a male leopard on Jazhna Mountain, which they reported as the first photo of a Persian leopard in Iraq (Raza et al., 2012b). Prior to 2011, Raza et al. (2012a) reported two records of leopard deaths in Iraq in 2008 and 2009. In 2012, Raza et al. (2012a) captured a second photograph of a leopard on Jazhna Mountain, however, the authors could not ascertain if this was a different individual as only one camera trap was deployed and the photo captured was the right flank. Interviews with locals in 2010 suggest leopards occupy the Kurdistan region, while another local claimed to have killed a leopard in self-defense (Raza et al., 2012a). Avgan et al. (in press) collated nine confirmed reports of leopard occurrence, mostly males, in the Zagros Mountains, extending from Iran into Iraq between 2001 and 2014. Given a sufficient prey base and available habitat Iraq may support a small population of transboundary leopards with Turkey and Iran (Avgan et al., in press; Raza et al., 2012b).

Kazakhstan

Status: Outside Historical Range
Last Record: 2000 (Shakula, 2004)

Kazakhstan is well outside the current or historical range of Persian leopards (Khorozyan, 2008a; P. Gerngross unpublished data). However, an old male leopard was killed in Kazakhstan in 2000 (Shakula, 2004), seemingly hundreds of kilometers from the nearest confirmed source population in Uzbekistan or Tajikistan. Prior to this confirmed presence, there had been no scientific evidence of leopards occurring in Kazakhstan.

Lebanon

Status: Extinct
Last Record: NA

The leopard was historically present throughout Lebanon (P. Gerngross unpublished data). Johnson (2003) claims leopards may be present in the Mount Hermon region in southern Lebanon, however, this claim is unsubstantiated. No additional information regarding the leopard in Lebanon was collected during this literature review and they are considered extinct.

Pakistan

Status: Extant

Besides the region of Gilgit-Balistan in the far northeast and the Thar Desert on the eastern border with India, leopards historically occurred throughout Pakistan (P. Gerngross unpublished data). Pakistan is where two subspecies, *P. p. saxicolor* and *fusca*, probably intermix; we use the Indus River as the boundary. Until as late as the 1970's, leopards were occasionally seen in almost all the mountains along the Karachi-Hyderabad highway and were rarely seen in the 1990's in the mountains that are connected with the Pab and Dumbar ranges (Husain, 2001). Leopards are critically endangered in Pakistan, and there is little recent confirmed evidence of leopards outside of the Himalayan Mountains. It is believed they are limited to forested regions at higher altitudes of the Himalayas, and to valleys in arid regions (Lodhi, 2007; Waseem & Kabir, 2010; Kabir et al. 2013), and occur at relatively higher densities in the northeast of the country (Roberts, 1997). Maan and Chaudhry (2000) reported a density of 1.69 individuals/100 km^2 in the Murree Hills, Rawalpindi District in the Punjab Province. Ahmed (2007) reported 30 leopards in the 8,000 acres of the Galiyat (also spelled Galliat) forests of the Ayubia National Park, while leopard estimates ranged from 60-250 in the Galiyat area.

Outside the Himalayan region, locals reported leopards in the Makran Mountains, Baluchistan, and a leopard was killed in the Kalatuk Mountain range in 2011 (Mohammad Kabir pers. comm. 2015). In 2013, a leopard was killed from the Kutcha area in Shah Belo near Sukkar, Sindh (Mohammad Kabir pers. comm. 2015).

Human-wildlife conflict and poaching represent two outstanding, critical threats to leopard persistence (Kabir et al., 2013). Waseem and Kabir (2010) used a combination of field observations, interviews, and local/expert knowledge to assess human-leopard conflict in Pir Lasora National Park, a protected area in the foothills of

the Himalayas. Between 2000 and 2010, Waseem and Kabir (2010) reported six retaliatory killings near villages, the trapping of one leopard, and three attacks on people. Lodhi (2007) compiled data from the Wildlife Department of Pakistan since 2000 on human-leopard conflict in and around Ayubia National Park, North West Frontier Province, and reported nine human deaths by leopards, 30 leopard killings, and 82 instances of livestock depredation. Kabir et al. (2013) conducted 173 interviews in villages in and surrounding Machiara National Park in northeastern Pakistan between 2007 and 2008. Respondents reported the depredation of 301 livestock (mostly goats and sheep), primarily due to poor animal husbandry practices, and ineffective or absent corrals. Also in and around Machiara National Park, Dar et al. (2009) found that leopards were believed to be responsible for over 90% of livestock and dog deaths from wildlife. Depredation was driving negative perception of the leopard although most respondents recognized it as protected under national law (Dar et al. 2009). More recent dietary analyses based on fecal investigations revealed continued moderate to high levels of leopard-livestock conflict in Machiara (Chatta et al., 2015), and Ayubia National Parks (Shehzad et al., 2015).

Russia (Caucasus Region)

Status: Extant

Historical distribution of the leopard in the Caucasus region of Russia is limited to the extent of the Greater Caucasus Mountains (P. Gerngross unpublished data). Although this estimate is several years old now, Khorozyan et al. (2008) speculated fewer than 10 leopards inhabited the North Caucasus. Askerov et al. (2015) suggests current leopard range in the Russian Caucasus is isolated into two patches, the Iori-Ajinour Plateau (southeast Georgia and northwest Azerbaijan), and the eastern Greater Caucasus (Georgia, and Dagestan/Russia). The leopard is likely absent in the western Greater Caucasus (Breitenmoser et al., 2007; Breitenmoser et al., 2014). However, it is possible these two populations are tenuously connected along the Greater Caucasus range within Kabardino-Balkaria, North Ossetia, Ingushetia, Chechnya, and Dagestan. Leopard reports are recorded on both Georgian and Russian side of the mountains (Breitenmoser et al., 2014). The leopard may persist in the Chegem River canyon of Kabardino-Balkaria (Khorozyan & Abramov, 2007) and in the upper Andi/Andiyskoe Koisu and Avarskoye Koisu drainages of Dagestan (Breitenmoser et al., 2007). There have been a few recent confirmed reports of leopard and a number of unconfirmed reports including one in the Argun River basin Chechnya in 2013, Kabardino Balkaria in 2013, and in Dagestan in 2013 and 2014 (Breitenmoser et al., 2014). In North Ossetia in 2011, the Russian military border service captured a video of leopard (Askerov et al., 2015), and a photo by the Gizeldon hydropower plant near the village of Kakhtisar, Kuban river watershed (Nugzar Zazanashvili pers. comm. 2015). In Dagestan, a hunter provided photographic evidence of leopard from April 2009, and a male leopard was captured on video in February 2015 near the border of Tlyaratinsky Federal Zakaznik between Tlyarata and Kamilukh (Yarovenko, n.d.).

The Kavkazskiy Zapovetnik region of the northwest Caucasus (and the Primorski-Krai in the Russian Far East) marks the northern edge of the leopard's global distribution. Local occurrence was eradicated decades ago, but the region is now considered for a reintroduction project (Breitenmoser et al., 2014). Two males from Turkmenistan and two females from Iran were transported to the Sochi reintroduction site in the Western Caucasus (T-PSV, 2010). Recently, additional founders were brought from European zoos to enrich the breeding stock in Sochi where they have started breeding (http://wwf.panda.org/?210435/Another-pair-of-Persian-leopards-born-in-Russia, accessed December 2015).

Syria

Status: Extinct
Last Record: 1963 (Masseti, 2009)

Excluding the Syrian Desert, the Persian leopard historically ranged throughout Syria (P. Gerngross unpublished data). The leopard is extinct in Syria and has likely been extinct since the 1960's (Shoemaker, 1993). The last confirmed report is of a leopard killed in 1963 outside the village of Bab Jannè approximately 20 km from the Turkish border (Masseti, 2009). Khalaf-von Jaffa (2002) and Johnson (2003) reference reports of leopards in the Golan Heights region, bordering Israel, however, concrete evidence is lacking.

Tajikistan

Status: Possibly Extinct
Last Record: NA

Persian leopard distribution in Tajikistan was historically limited to the border regions of Afghanistan and Uzbekistan (P. Gerngross unpublished data). The leopard may have gone extinct in 1977 (Sokov, 1990) but anecdotal reports continue and the leopard may still persist in the country. Local reports claimed leopard presence in the Khodza-Kazian and Teke-Kamar Mountains, a region where the leopard was suspected to have gone extinct by the late 1980's (Lukarevsky, 1996). Claims were dismissed following searches for leopard sign in the region between the Vaksh and Kafimigan Rivers in 1989 (Lukarevsky, 1996). There are historic and recent leopard records in the Babatag Mountains of Uzbekistan, bordering Tajikistan (Natalya Marmazinskaya pers. comm. 2016).

Turkey

Status: Extant

Historically, the leopard occurred along the coast in western Turkey, along the Taurus Mountains in the south, and throughout most of eastern Turkey (P. Gerngross unpublished data). Can Bilgin (pers. comm. in T-PVS, 2010) suggested a countrywide estimate of 40-60 leopards on the basis of "new evidence" since the 1990's, with no more than 5-10 individuals in any given region. However, intensive camera trapping efforts along northeastern and eastern Turkey initiated in 2007 failed to document leopard presence (Breitenmoser et al., 2007). Baskaya and Bilgili (2004) reportedly found footprints at 16 sites in the Eastern Karadeniz Mountains, but no hard evidence was provided to substantiate claims. Khorozyan (2009), however, reported a harvested leopard in 1999 in Yusefeli County of the Eastern Karadeniz Ridge near the border with Georgia. Also, Johnson (2003) references expeditions undertaken by two separate field teams in the spring of 2002 claiming to have footage of leopards in Turkey, one of a leopard in a pine tree and the second of a leopard along a Hazidag outcrop, but no evidence was provided. Locals reported leopards around Mount Ararat in the 2000's and a poacher near there shot a leopard in 2007 (T-PVS, 2010). However, no leopards were found despite 8,832 camera trapping days and 77,243 photos between 2010-2015 in the forests surrounding Sarıkamış Allahuekber Mountains National Park (Chynoweth & Sekercioglu in prep.). Regardless of the lack of a documented population, leopards may continue to inhabit northeastern and eastern Turkey as transients from Iran and neighboring countries (Cagan Sekercioglu pers. comm. 2013).

Outside of the Caucasus region, there are a few recent records (Breitenmoser et al., 2014). Avgan et al. (in press) surmise a small reproducing population exists in the northwestern part of Zagros Mountains in western Iran, northern Iraq and southeastern Turkey based on four leopard casualties (three males and one of unknown gender) since the early 2000's in southeastern Turkey and the long distance from a breeding population in Iran. Poachers killed a leopard near Lake Van in 2006, and other reports of leopards near Şemdinli in the extreme southeast, and south of Uludere continue (T-PVS, 2010). In November 2010, a male leopard was found dead at Mt. Gabar in Şirnak near the border with Iraq (Breitenmoser et al., 2014). Farther to the west, in November 2013, reports of a leopard shot and killed by a Diyarbakır shepherd made media coverage (Mehmet, 2013) and is the most recent confirmed observation of the leopard in Turkey. In and around the Beydağları Mountains Termessos National Park near the Mediterranean coast, Albayrak et al. (2012) conducted interviews and camera trap surveys between 2005-2009, but failed to photograph leopards or obtain anecdotal evidence.

Turkmenistan

Status: Extant

Historically, the Persian leopard occurred in regions of higher elevation bordering Iran and Afghanistan in southwestern and southeastern Turkmenistan, respectively (P. Gerngross unpublished data). Overhunting contributed to population decline throughout Turkmenistan, as 360 leopards were killed from 1924 to 1966 (Atamuradov et al., 1999). In 1990 the leopard population was roughly estimated at 130-150 individuals (Chemonics International Inc., 2001). Lukarevsky (2001) later estimated a countrywide estimate of 78-90 leopards in Turkmenistan although Atamuradov et al. (1999) guessed the total number of leopards in Turkmenistan was no larger than 30-40 individuals. In 2007, Lukarevsky et al. reported on "reliable sightings" of 28 leopards including some with kittens in Turkmenistan and Iran, however, the date and location of these sightings were not specified. Around the year 2000, leopards were reported as present in the Badkhyz State Nature Reserve, Kopetdag Ridge, and Giaz-Gyadyk Ridge (Lukarevsky, 2001). Most recently, in 2014, Kaczensky and Linnell (2015) captured photographs and identified spoor of leopards, including breeding females, in the Badkhyz State Nature Reserve near Iran.

Uzbekistan

Status: Possibly Present

If present, likely no more than about 10 individuals still occur in two small, discontinuous mountain ranges (the Babatag and Kugitang) in the far southeastern region of Uzbekistan bordering Afghanistan, Tajikistan, and Turkmenistan (P. Gerngross & N. Marmazinskaya unpublished data). No detailed studies of leopards have been undertaken in Uzbekistan. The Kugitang Mountains represent an isolated patch of leopard range (Henschel et al., 2008) and are protected via the Surkhanskiy Nature Reserve in southern Uzbekistan along with the Kugitang Nature Reserve and Hodjapil Nature Sanctuary in Turkmenistan. In the north Kugitang Mountains, a leopard killed a horse in 1976, and in 2010 poachers killed a leopard (Natalya Marmazinskaya pers. comm. 2016). There are several historical records of leopard in the Babatag Mountains on the border with Tajikistan. An individual was killed at the rock Radzhab-Markhur near Itbulak in 1950. Possible leopard

presence was also noted around the 1950's at gorges near the springs of Kashkabulak, Big Turanga, Pattali, Biyessimam, Kardzhumalak, in the Gaurgen gorge and Paytal-Tudgy's gorge. Based on interview data, a leopard was spotted between Itbulak and Anorbulak springs in 2006, young leopards were noted in 2009 near the Orlinaya sopka (between the Amu Darya River and South Babatag Mountains), and spoor is regularly spotted around Kumkurgan in southern Babatag (N. Marmazinskaya unpublished data). The primary threats to leopards in the country are poaching and prey loss (Bogdanov 1952; Ishunin 1961; Anonymous 1983, 2009).

Indian Leopard

Panthera pardus fusca

South Asia

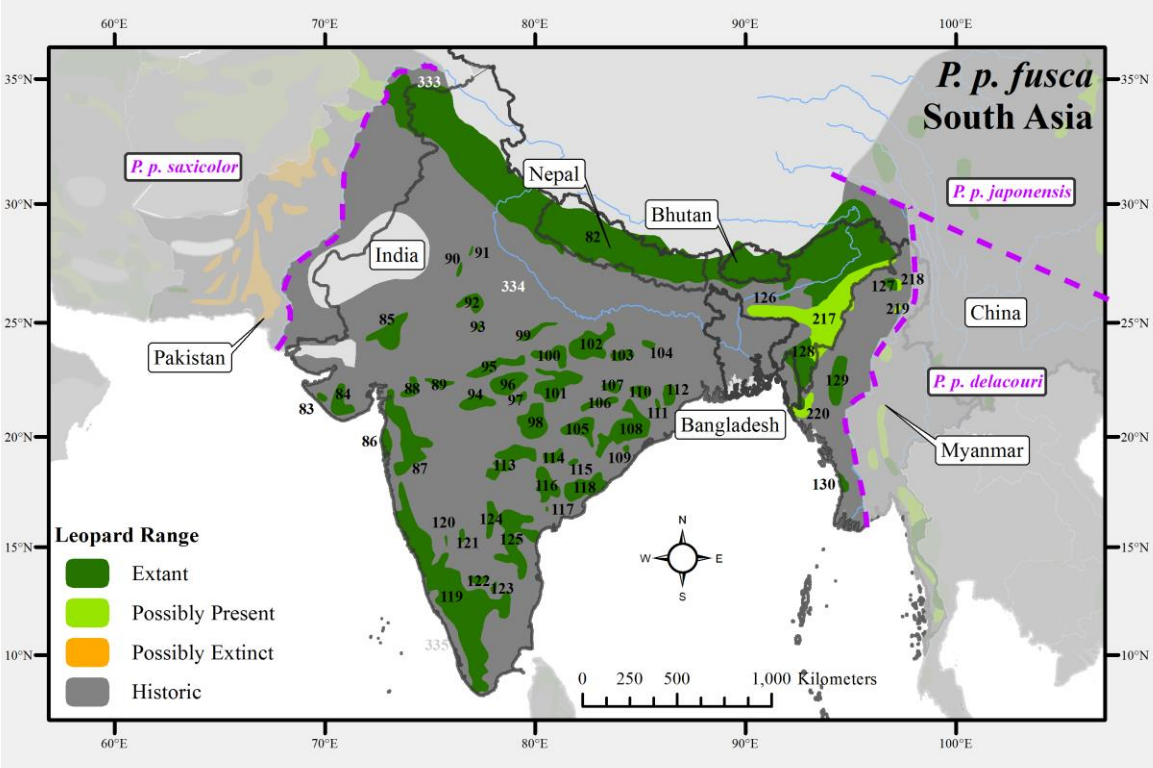

Bangladesh

Status: Extant

Historically, the Indian leopard occurred throughout Bangladesh (Green, 1987) but it is debatable whether they occurred in the most remote regions of the Sundarbans (P. Gerngross unpublished data). Data on leopard population and distribution is extremely limited for Bangladesh (Green, 1987). By the 1980's, the leopard was already threatened with extinction and restricted to a few small populations in the evergreen forests of Sylhet, Chittagong, Chittagong Hill Tracts, and Cox's Bazaar forest division (Green, 1987). While totally protected by the law, habitat disturbance, hunting, and depletion of prey base were identified as important threats (Green, 1987).

The only recent or ongoing research appears to be in southeastern Bangladesh. In select areas of Chittagong Hills with more than 50% forest cover, Chakma (2015) conducted track and sign surveys from 2010-2011 and found leopard occupancy to be near 50%. In 2015, a leopard was captured in a camera trap in the extreme southeast corner of Chittagong Hills Tract on the border with Myanmar (Shahriar Cesar Rahman and Suprio Chakma pers. comm. 2015). In the extreme south, villagers killed a leopard in 2012 or 2013 in the Tekhnaf

Peninsula near Cox's Bazaar forest (Suprio Chakma pers. comm. 2015). However, there are anecdotal reports of leopards elsewhere. Newspaper reports suggest leopards may continue to reside in the Sylhet, Moulovibazar, and Panchagar districts based on occasional sightings and killings of leopards by locals (Abidur Rahman pers. comm. 2015). Additionally, a leopard reportedly killed a cow in the Lauchapra Forest of the Balijhuri Range in 2000 (Khan, 2004).

Bhutan

Status: Extant

Historically, the leopard was abundant and widespread throughout Bhutan up to approximately 3300 m asl and did not occur at the highest elevations bordering China (Green, 1987; Wangchuk, 2004; P. Gerngross unpublished data). Approximately 70% of Bhutan is forested and is known to support leopards as well as 11 other felid species (Wang & Macdonald, 2009). Into the 1980's, the leopard was still numerous but "nothing" was known about the size of the population and there was no "precise information" regarding distribution (Green, 1987). Green (1987) identified no immediate threats to leopards in the country although legal protection was not yet in place and leopards were known stock raiders. However, the leopard became fully protected by law due to the Nature Conservation Act of 1995 (Wang & Macdonald, 2006).

In the 1980's, leopards occurred in Manas and Neoli Wildlife Sanctuaries, Phipsoo (Mochu) Forest Reserve, and were likely present in the massive Jigme Dorji Wildlife Sanctuary (Green, 1987). More recently, in 2006-2007, Wang and Macdonald (2009) conducted a camera trap study in the Jigme Singye Wangchuck National Park located in south-central Bhutan and captured 48 photographs of leopards over 50 days. Despite the availability of contiguous tracts of habitats, leopards were found to occur at a comparatively low density of 1.04 leopards per 100 km^2 (Wang & Macdonald, 2009). Additional camera trapping studies conducted by Tempa et al. (2013) captured 107 photographs of leopards in the Royal Manas National Park from 2010-2011 but they did not calculate a population density estimate.

In a study of human-wildlife conflict throughout the country conducted by Sangay and Vernes (2008), leopards were responsible for 70% of livestock depredations documented over a two-year period. Wang and Macdonald (2006) noted that leopards were also the most important predator of livestock at Jigme Singye Wangchuck National Park and that conflict increased after the establishment of the park in 1993. In Toebesa Gewog of the Punakha Dzongkhag, northeast of Thimphu, Katel, Morin & Kelly (2013) found that leopards were the secondmost important predator of livestock to the dhole (*Cuon alpinus*). Conflict with humans and livestock is a prevailing issue among Bhutan's large carnivores (e.g., tigers [*Panthera tigris*] and leopards) and responses to livestock loss default to retaliatory killings despite their protected status (Sangay & Vernes, 2008; Wang & Macdonald, 2009).

China

Status: Extant

The Himalayan Mountains extend from Arunachal Pradesh, India, into southern China and hence the Indian leopard is also expected to extend into southern China (P. Gerngross unpublished data). The Indian leopard subspecies intermixes with *P. p. japonensis* and *delacouri* somewhere to the east of Namcha Barwa, the

eastern end of the Himalayas. In south-central Tibet, leopards were recorded in surveys from 2010 to 2012 in the forested areas of Mt. Qoomolangma (Mt. Everest) National Nature Reserve, between 2,000 to 3,000 m (Jiazuo County; Hu et al., 2014). Furthermore, researchers saw a leopard and a leopard skin was recovered from a villager's house in Resuo County (Hu et al., 2014).

India

Status: Extant

Historically the leopard occurred throughout India with the exception of high altitude areas in the Tibetan plateau and the Thar Desert region primarily in Rajasthan (P. Gerngross unpublished data). In Himachal Pradesh, the leopard is widely distributed up to ~3,400 m in elevation (Green, 1987). India remains the primary stronghold for *P. p. fusca* and the leopard is found across the country, both within protected areas and outside of them in human-dominated landscapes (Athreya et al., 2015). Some of the highest leopard densities are recorded in India (e.g., 30.9 individuals per 100 km^2 in Sariska Tiger Reserve; Edgaonkar, 2008) due to the tremendous productivity of the landscape. However, leopards have declined over time due to habitat loss, decreasing prey densities, poaching and, historically at least, via hunting. Hunters killed roughly 150,000 in 50 years from 1875 to 1925 (Ranganathan 200). Most scientific studies of leopard distribution or abundance are centered on protected areas and hence information on leopard distribution outside protected areas is scant (Ghosal et al., 2013; Athreya et al., 2015). Leopard numbers in tiger ranging reserves and adjoining landscapes are enumerated each year by the Indian government (Jhala et al., 2011; 2015). A recent and not yet released survey by the Wildlife Institute of India, surveyed tigers and leopards using camera traps and estimated a countrywide total of 12,000-14,000 individuals (http://timesofindia.indiatimes.com/home/environment/flora-fauna/Finally-India-gets-a-count-of-its-leopard-numbers-12000-14000/articleshow/48850420.cms, accessed December 2015). Large subpopulations are reported in northern (Uttarakhand), central (Madhya Pradesh, Maharashtra, Chhattisgarh) and southern (Karnataka) India. Smaller, restricted subpopulations are distributed throughout the subcontinent.

Conflict is a little understood phenomenon. Leopards and humans interact frequently and in many different ways although interactions are primarily labeled as 'conflict' (Inskip & Zimmermann, 2009). Ecological studies and media reports typically focus only on a portion of these interactions, i.e., attacks on livestock and humans (Goyal, 2000; Vijayan & Pati, 2002; Madhusudan, 2003; Karanth et al., 2012; Karanth et al., 2013; Athreya et al., 2015). Any non-negative interactions are rarely mentioned in ecological studies although they are sometimes discussed through social sciences (Ghosal & Kjosavik, 2015). Through interviews in the Western Ghats and in central India around Kanha Tiger Reserve, leopards were ranked as some of the most important predators on livestock (Karanth et al., 2012; 2013). Conflict with livestock was also reported in Junnar, and indeed throughout much of India (Athreya et al., 2004). Changing crop patterns was one possibility as to why leopard attacks on livestock and humans may have been increasing around the Gir protected area (Vijayan & Pati, 2002). Leopards are also responsible for many human deaths (Joshi & Agarwal, 2012; Dhanwatey et al., 2013; Athreya et al., 2015). Translocation of the leopard is a frequent outcome of conflict although research has suggested that this practice is ineffectual and may increase human-leopard conflict (Athreya et al., 2011). Joshi and Agarwal (2012) found that 50 leopards were declared man-eaters between 2000 and 2008 in Uttarakhand and most of these were ultimately killed.

Poaching is a serious ongoing threat to leopards in India (Raza et al., 2012c). The illegal fur trade has been ongoing for decades, particularly since a boom in the 1960's (Green, 1987). Raza et al. (2012c) estimated nearly 2,300 leopards were poached and illegally traded between 2001 and 2010. Aghor (2008) estimated

500 leopards are killed annually across India. Leopard mortalities due to vehicular collisions also seem to be increasing in recent years likely due to an expanding road network and increasing vehicular traffic (Gubbi et al., 2014b).

Interspecies interactions also affect leopard abundance and distribution. Leopard densities can be greater in areas where tigers have been extirpated (Edgaonkar, 2008; Mondal et al., 2012) although leopards may still achieve high densities where tigers are present (Kalle et al., 2011). The reintroduction of tigers in Sariska Tiger Reserve elicited a shift in the spatial and temporal occurrence of the leopard to achieve segregation of the two cats (Mondal et al., 2012). This conforms to some studies outside of India where tigers displaced leopards from prime habitat and pushed them to the margins of protected areas through interference competition (Odden et al., 2010). Therefore it is possible that increasing tiger populations through conservation actions may negatively impact leopard populations.

Since 2000, a minimum of eight peer-reviewed leopard population density and distribution studies were published in India (Supplemental Table 7) with many more contained within unpublished reports. Population densities were estimated at 3.4 individuals/100 km^2 in Manas National Park (Borah et al., 2014), 1.68 in Manas core area (Goswami & Ganesh, 2014), 23.5 in Sariska Tiger Reserve (Chauhan et al., 2005), 15 in Chilla range of Rajaji National Park (Harihar, Pandav & Goyal, 2009), 4.8 in Maharastra (Athreya et al., 2013), 4.2-6.2 in Satpura Tiger Reserve (Edgaonkar, 2008), 14.9 in Mudumalai Tiger Reserve (Kalle et al., 2011), 1.1-2.99 in Pakke Tiger Reserve (Selvan et al., 2014), and 11.1 in Bhadravathi Territorial Division (Gubbi et al., 2014a). Dutta et al. (2012) used genetics from scat to estimate effective population sizes and contemporary gene flow in the central Indian highlands between four tiger reserves: Satpura, Melghat, Pench, and Kanha. Despite all these studies, more research is still needed, with an emphasis in unprotected landscapes, to more precisely determine leopard occupancy across India.

Myanmar

Refer to *Panthera pardus delacouri*

Nepal

Status: Extant

Historically, the leopard occurred throughout Nepal but was not known to occur along the northern border between Nepal and China (Shrestha, 1997; P. Gerngross unpublished data). Nepal is situated within the Himalayan Mountains, and the leopard persists in a band along these mountains. Hence, the Nepalese population is contiguous and supplemented by leopards in other Himalayan range countries such as Pakistan, India, Bhutan, and China. A camera trap recently caught a melanistic leopard at the highest recorded elevation in Nepal at 4,300 m (Thapa et al., 2013). In a status review of Nepal's mammals, Jnawali et al. (2011) estimated less than 1,000 leopards remain countrywide. Despite protection under CITES, there is no national legislation to protect leopards in Nepal, and human-wildlife conflict continues to pose threats for leopards (Jnawali, 2011). Poaching for the illegal wildlife trade is also a substantial threat (Aryal, 2003). Other threats include poisoning and habitat destruction (Shrestha, 1997).

Appel et al. (2012) documented leopard sign (photo, spoor, and scat) and anecdotal reports of leopards throughout the 7,629 km^2 Annapurna Conservation Area in north-central Nepal but no population figure was

provided. In addition, leopard presence was reported in Dhorpatan Hunting Reserve (Aryal & Kreigenhofer, 2009), Makalu-Barun National Park (Ghimirey & Acharya, 2012), Royal Chitwan National Park (Appel, Ghimirey & Acharya 2012), and Kanchanjunga conservation areas (Thapa et al., 2013). The *National Biodiversity Strategy Report* (HMGN/MFSC, 2002) also detailed leopards in Khaptad, Koshi Tappu, Parsa, Rara, and Shivapuri National Parks and Wildlife Reserves. In Bardia National Park, Wegge et al. (2009) estimated leopard density was consistent at 5 individuals/100 km^2 although prey and tiger populations both increased after the establishment of the park. In the subtropical forests of Parsa Wildlife Reserve, Thapa et al. (2014) estimated 5.6 leopard/100 km^2 using spatially-explicit capture-recapture models.

Pakistan

Refer to *Panthera pardus saxicolor*

Sri Lankan Leopard

Panthera pardus kotiya

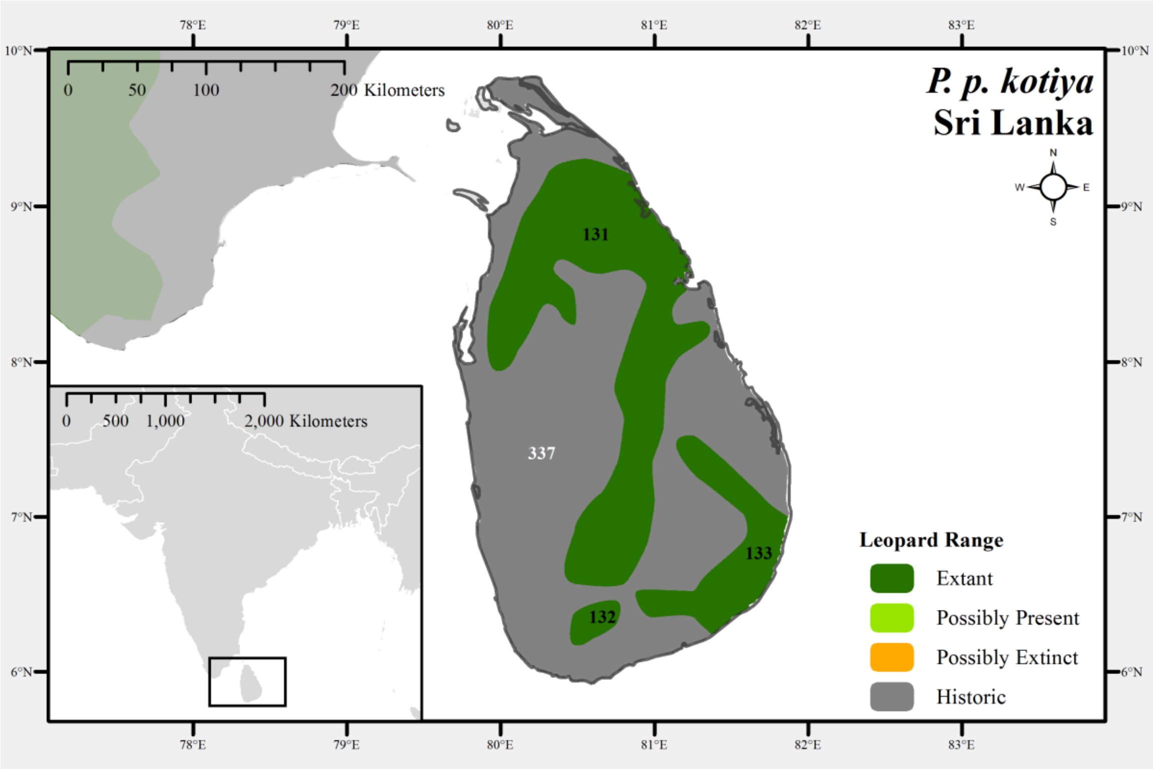

Sri Lanka

Status: Extant

Leopards historically ranged throughout Sri Lanka (Phillips, 1935; P. Gerngross unpublished data) and have been the apex predator on the island for at least the last 5,000 years (Kittle & Watson, 2007a). The leopard suffered intense commercial exploitation for its skin (Phillips 1984), and by the early 1980's, the population had declined sharply and Santiapillai, Chambers & Ishwaran (1982) estimated less than 1,000 leopards. The subspecies was listed as Endangered in the 1994 Red List update (Kittle and Watson 2008). Kittle and Watson (2007b) used data collected from five years of presence-absence surveys to estimate a population of 700-950 leopards in Sri Lanka based off density estimates for leopards in regions of varying habitat quality. No sub-population was determined to support more than 250 individuals. Poaching and deforestation remain as longstanding threats to leopard populations on Sri Lanka (Shoemaker, 1993; Santiapillai & Jayewardene, 2004; Kittle & Watson, 2005). In the mid-19th century, approximately 80% of Sri Lanka was forested (Santiapillai & Jayewardene, 2004) but this has since dropped to just 20% (Kittle & Watson, 2005). Poaching for the illegal wildlife trade, including trade with India, also poses an ongoing threat (Kittle & Watson, 2003). In northern Sri Lanka (i.e., the Wanni jungles), civil conflict has prevented wildlife surveys, but leopard presence is expected based off remaining suitable habitat (Kittle & Watson, 2005). Human-leopard conflict is present as some leopards kill cattle and domestic dogs (Phillips, 1984), but Kittle et al. (2014) found domestic prey was

virtually absent from leopard scat in the central highlands. In addition, man-eating is rare and the last habitual man-eater was a leopard from Punani, killed in 1924 (Phillips, 1984).

The most recent island-wide survey found leopards in 44 locations, including 16 outside formal protected areas (Kittle & Watson, 2005). Kittle and Watson (2005) found resident populations in all major climatic zones of the country except for the most highly developed areas of the western wet zone. Furthermore, they estimated the extent of occurrence was ~37,650 km^2, or over 50% of the country, but the area of occupancy (areas where reproductive adults are verified) at less than 15% of the country (Kittle & Watson, 2007a). Kittle and Watson (2002) also conducted the only rigorous population study on the island, estimating a leopard density of 17.9 individuals/100 km^2 in block I of Ruhuna (Yala) National Park. This block (~140 km^2) is probably the best remaining leopard habitat on the island, and thus represents the very high end of population density estimates for Sri Lanka (Kittle & Watson, 2008). Finally, as an illustration of how leopards have adapted to more anthropogenic habitats, an eight-year study of habitat use in the central hills region revealed the long-term use of small (<5 km^2) patches of secondary forest by leopards spanning >5 years (Kittle et al., 2012).

Indochinese Leopard

Panthera pardus delacouri

Southeast Asia

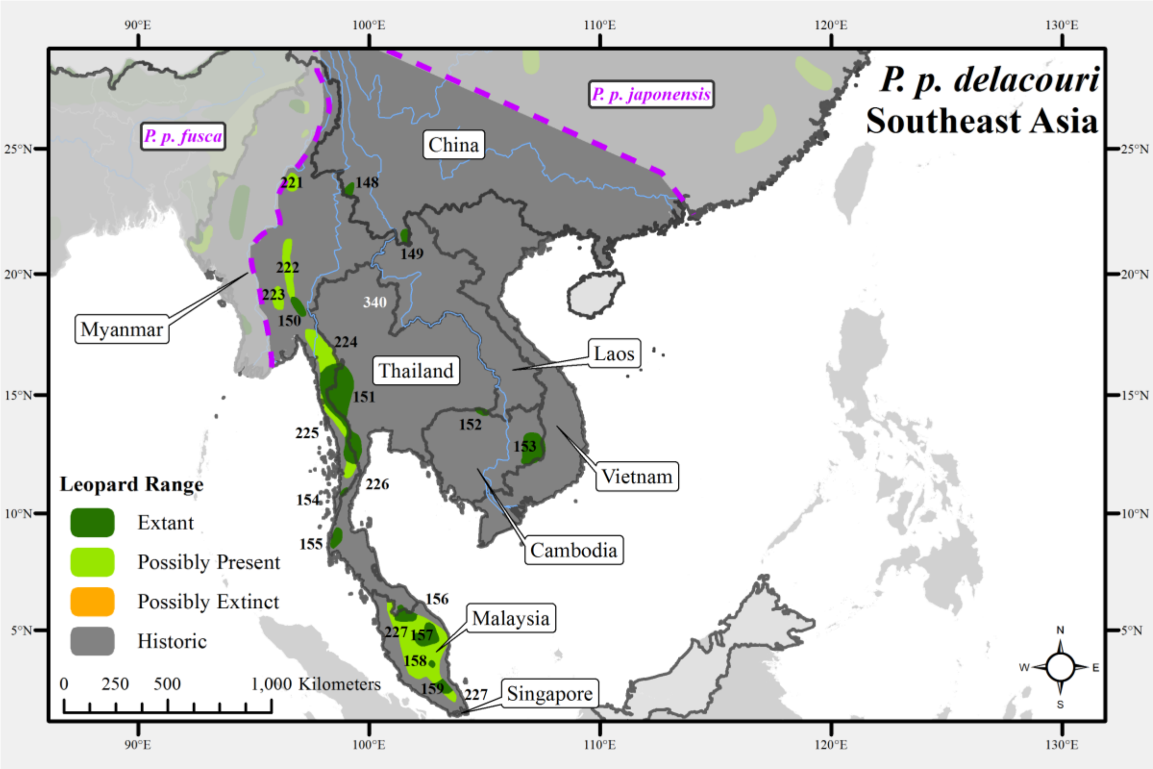

Cambodia

Status: Extant

Historically, the Indochinese leopard occurred throughout Cambodia (P. Gerngross unpublished data). A recent study reviewed the current status and distribution of leopard in the country (Rostro-García et al., 2016), and the following is a summary. There appears to be only one remaining viable population of leopard in the country, within the Eastern Plains Landscape (EPL). Leopard was recently detected in four protected areas within EPL (Freeland Foundation, WCS Cambodia, WWF Cambodia, unpublished data), as well as two nearby areas (Chhlong area [Kratie Province] and Prey Khieu Forest [Mondulkiri-Stung Treng provinces]; Gray et al., 2012). However, in Mondulkiri Protected Forest, the largest protected area within EPL, the leopard population decreased 70% from 2009 (3.6 leopard/100 km^2; Gray and Prum, 2012) to 2014 (1.1 leopard/100 km^2; S. Rostro-García, unpublished data), indicating this subpopulation is decreasing rapidly and might soon become extirpated. Poaching for the illegal wildlife trade is likely the main reason for the rapid decline of leopard in EPL because prey numbers remained stable during that period. Leopard also was recently detected in the Northern Plains Landscape, however only two individuals were identified in 3 years of extensive camera trapping in Preah Vihear Protected Forest (Ai Suzuki, unpublished data), indicating this

population probably is not viable, especially given that leopard was not detected in neighboring PAs in Cambodia or Thailand. Leopard appears to have been extirpated from the Cardamom Mountains because no individuals were detected in the region despite recent extensive camera trapping in two of the main protected areas in the complex. Similarly, the leopard is probably now extirpated from the Northeastern Complex, which includes Virachey National Park. Leopard was not detected in at least eight additional sites outside of these complexes. Because both the Cardamom Mountains and Northeastern complexes still contain extensive forests and apparently sufficient prey numbers, extirpation of leopard in these regions was likely due to poaching for the illegal wildlife trade.

China

Status: Extant

The leopard historically occurred throughout central, eastern and southern China, with the exception of Taiwan, and Hainan (Smith et al., 2010; P. Gerngross unpublished data). The leopard was absent from the highest elevations of the Himalayas, the Tibetan plateau, Xinjiang province, and, loosely speaking, a swath of northern China roughly parallel with the Mongolian border (Ma, 1998). Given the current delineation of nine leopard subspecies (Uphyrkina et al., 2001), four occurred in China: *P. p. fusca*, *delacouri*, *japonensis*, and *orientalis*. Historically, *P. p. fusca* probably had the smallest range in China, as it was largely restricted to the Himalayas (see *P. p. fusca* China). *P. p. japonensis* is endemic to China, occurring through the central and eastern portions of China. However, where *P. p. japonensis* mixes with the other three subspecies is poorly known. It's likely there was not a hard, defined geographical boundary between any of the subspecies, rather, the subspecies likely intermixed extensively and had clinal variations as suggested by Miquelle et al. (2010) for *P. p. orientalis* and *japonensis*. Additionally, it's unlikely we could ever know the exact boundaries as leopard range has contracted extensively throughout China and leopard populations are now isolated.

National or countrywide programs and policies likely affect all subspecies. Predator control programs during the 1950's had severe impacts on leopard populations in China, killing as many as 2,000-3,000 leopards annually in the southern region of the country (Shoemaker, 1993). Current Chinese legislation categorizes leopards as Class I protected species, prohibiting hunting and trade of derived products. However several incidences of retaliatory killing and poaching are still documented in the media and in local reports. Other key threats are habitat loss and fragmentation, and prey depletion, even in protected areas (Jutzeler et al., 2010).

At the national level, leopard distribution and population has declined precipitously. The China Red Data Book of Endangered Mammals estimated 1,000 individuals (Ma, 1998). The State Forestry Administration estimated 3,310 individuals based on the First National Terrestrial Wildlife Survey conducted between 1995 and 2003 (State Forestry Administration, 2009). However, based on a countrywide review and extrapolation based on leopard densities of no more than 2 leopards/100 km^2, Laguardia et al. (2015) estimate no more than 400 individuals countrywide with *P. p. japonensis* as the most numerous subspecies. (Li et al., 2010)

In southeastern China, *P. p. delacouri* was historically distributed widely south of the Pearl River. No physical evidence of leopards in Hong Kong was collected during this literature review and leopards have long thought to be extinct in Hong Kong (Henschel et al., 2008). From 2000 onwards, leopards have only been recorded in camera traps in Nangunhe and Xishuangbanna National Nature Reserves, southwestern Yunnan Province near the border with Myanmar (Jutzeler et al., 2010; Laguardia et al., 2015). Worse, these

populations are isolated from one another and from any known populations in other Southeast Asian countries. The remaining population of *P. p. delacouri* in China is low (probably < 10 individuals) and is unlikely to recover due to high levels of habitat fragmentation, poaching, and low prey numbers (Laguardia et al., 2015). There are no other recent records of *P. p. delacouri* in southeastern China, and this subspecies might be on the verge of extirpation in the country (Laguardia et al., 2015).

Lao PDR (Laos)

Status: Possibly Extinct
Last record: 2004 (Wildlife Conservation Society Lao PDR Program, unpublished data)

Historically, the Indochinese leopard occurred throughout Laos (P. Gerngross unpublished data). A recent study reviewed the current status and distribution of leopard in the country (Rostro-García et al., 2016), and the following is a summary. Recent surveys indicate the leopard is probably absent from the country. For example, in Nam Et-Phou Louey National Protected Area in northern Laos, one of the largest and best protected areas in the country (Johnson et al., 2006), leopard was last photographed in 2004, despite extensive annual camera-trapping and DNA testing of >500 scats since that time (Wildlife Conservation Society Lao PDR, unpublished data). Similarly, leopard was not recorded during extensive camera-trap surveys in Nakai-Nam Theun (Coudrat et al., 2014) and Nam Kading, in central Laos (Wildlife Conservation Society Lao PDR Program, unpublished data), or during field surveys in Xe Sap National Protected Area in southern Laos (Gray et al., 2013). Leopard was not detected in any additional surveys in other areas of country during the past 15 years. This species seems to have disappeared from areas that contain sufficient habitat and prey (e.g., Nam Et-Phou Louey), suggesting local extirpations were likely because of poaching for the illegal wildlife trade, similar to that observed in neighboring Cambodia. The leopard is now functionally extinct, if not fully extirpated, from Laos.

Malaysia (peninsular)

Status: Extant

Historically, the Indochinese leopard occurred throughout Peninsular Malaysia (P. Gerngross unpublished data). A recent study reviewed the current status and distribution of leopard in the country (Rostro-García et al., 2016), and the following is a summary. Leopard still occurs throughout most forests in the country, including large complexes in the north (Belum-Temengor; Rayan et al., 2013), central (Taman-Negara; Kawanishi & Sunquist, 2004, Hedges et al., 2015), and south (Endau-Rompin; Gumal et al., 2014). In a wildlife corridor within the Taman-Negara Complex, leopard density was recently estimated to be about 3 leopards/100 km^2 (Hedges et al., 2015). Leopard was also detected in fragmented secondary forests and plantations southwest of Kuala Lumpur (Sanei & Zakaria, 2011), and in at least seven other forest fragments around the country, including Ayer Ngah, Jengai, Jerangau, Krau, Ulu Lepar, Ulu Muda, and Ulu Temiang (Laidlaw, Rahmna & Zainal, 2000; Mohd Azlan & Sharma, 2006; Lynam et al., 2007; Tan et al., 2015). Peninsular Malaysia is one of the last remaining strongholds for the Indochinese leopard, although the population may be under threat from recent poaching. There is recent evidence of leopard poaching in Peninsular Malaysia, and leopard was not detected in 36% (5 of 14) sites that were surveyed outside of the three major complexes mentioned above. Nevertheless, poaching is presumably not as extensive as other countries in Southeast Asia, so most forested areas in the country could potentially contain leopard. Interestingly, all records from

Peninsular Malaysia are of melanistic leopard (Kawanishi et al., 2010), except for a few individuals (Tan et al., 2015).

Myanmar

Status: Extant

Historically, the Indochinese leopard occurred throughout Myanmar (P. Gerngross unpublished data). A recent study reviewed the current status and distribution of leopard in the country (Rostro-García et al., 2016), and the following is a summary. Leopard was detected in only 8 of 18 survey areas located throughout the country from 1999 to 2004, and distributions likely decreased since that time due to increased poaching. In fact, several border areas of Myanmar have become centers for the illegal trade in wildlife parts, and numerous leopard parts are reportedly sold there (e.g., Shepherd & Nijman, 2008). A more recent publication indicated leopard presence in 8 of 43 protected areas on the basis of information provided by the Myanmar Forest Department (Instituto Oikos, 2011), suggesting that leopard is absent from most of the country. For example, although the Northern Forest Complex has several large protected areas, leopard is probably functionally extinct there. In this complex, the only recent verified leopard records were from the core zone in Hukaung Valley in 2007, despite extensive annual camera trapping from 2001 to 2011 (Naing et al., 2015), as well as camera trapping in several other protected areas in the complex (Zaw et al., 2014). The largest viable population of leopard in the country is in the Southern Forest Complex in the peninsula, which is contiguous with two large forest complexes in Thailand that contain leopard (WEFCOM and Kaeng Krachan-Kuibiri complexes – see Thailand below). Outside of this complex there are probably only three small viable populations of leopard remaining in Myanmar: Alaungdaw Kathapa – Mahamyaing complex, Rakhine Yoma Elephant Range Wildlife Sanctuary, and northern Karen (Kayin) State.

Singapore

Status: Extinct
Last Record: 1990 (Yang, Yong & Lim 2000)

Historically, the Indochinese leopard occurred throughout the country (P. Gerngross unpublished data), but the leopard is extinct in Singapore (Panthera, 2012), and has likely been extinct since at least 1990 (Yang, Yong & Lim 2000).

Thailand

Status: Extant

Historically, the Indochinese leopard occurred throughout Thailand (P. Gerngross unpublished data). A recent study reviewed the current status and distribution of leopard in the country (Rostro-García et al., 2016), and the following is a summary. Currently, leopard occurs only in 4 of 19 protected area complexes located throughout the country: the Western Forest Complex (WEFCOM), Kaeng Krachan-Kuibiri Complex, Klong Saeng-Khao Sok Complex, and the Hala-Bala Complex. In the late 1990's and early 2000's, there were a few leopard records in forest complexes in the north-central (Phu Khieo-Nam Nao Complex; Borries & Koenig, 2014) and south-central parts of Thailand (Dong Phayayen-Khao Yai Complex; Lynam, Round & Brockelman, 2006; Ngoprasert et al., 2012), but extensive camera trap surveys have indicated they are now

likely extirpated from these areas. There have been few wildlife surveys in northern Thailand, but viable populations of leopard are unlikely to occur there given high rates of deforestation and poaching in the area. The WEFCOM is the largest complex where leopard still occurs, and within this complex Huai Kha Khaeng Wildlife Sanctuary is the best protected area with probably the highest leopard density (Rabinowitz, 1989; Simcharoen et al., 2008). Nevertheless, leopard was not detected in all protected areas within the complex, indicating this leopard population might be under threat. Kuiburi National Park also was reported to have relatively high leopard densities (Steinmetz et al., 2009). In fact, WEFCOM and Kaeng Krachan-Kuibiri Complex, together with the adjacent Southern Forest Complex in Myanmar, comprise one large contiguous forest complex. Outside of these complexes, leopards were recently detected only in the peninsula, including both the Klong Saeng-Khao Sok Complex and Hala Bala Wildlife Sanctuary. Although leopard was recently recorded in Klong Saeng Wildlife Sanctuary, they might not occur in adjacent Khao Sok due to extensive human disturbance in that protected area (Luke Gibson, pers. comm. 2014).

Vietnam

Status: Possibly Extinct
Last record: 2004 (Eames et al. 2004)

Historically, the Indochinese leopard occurred throughout Vietnam (P. Gerngross unpublished data). A recent study reviewed the current status and distribution of leopard in the country (Rostro-García et al., 2016), and the following is a summary. There are no recent leopard records from Vietnam, although in the 1960’s and 1970’s there was still an estimated 400-500 leopard skins per year in northern Vietnam (and an estimated 3,000-3,200 individuals in the region). In contrast, there was no leopard documented in southern Vietnam even as far back as the 1960’s (Shoemaker, 1993; Tien n.d.). From 1995 to 2013, there were no photographs of leopard from camera-trapping studies in the country, including those in the largest and best protected areas, such as Cat Tien National Park. The last unverified report of leopard from Vietnam was probably from the early 2000s in Yok Don National Park in central Vietnam (Eames et al., 2004), which might have been a transient leopard originating from the adjacent population in eastern Cambodia. It is doubtful that leopard still occurs in Yok Don given high levels of hunting and snaring in Vietnam, which have decimated populations of smaller felids in the country (Willcox et al., 2014). There appears to be no viable leopard populations remaining in Vietnam, and this species is likely extirpated from the country.

North Chinese Leopard

Panthera pardus japonensis

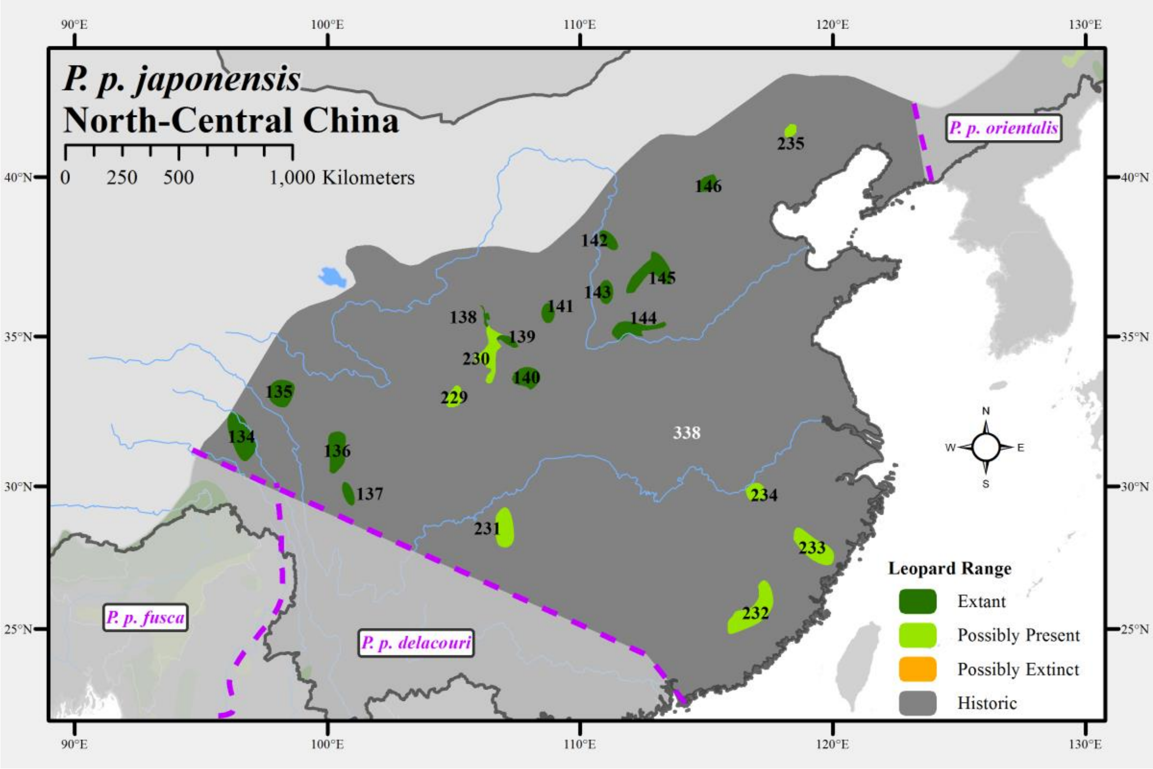

China

Status: Extant

This subspecies is endemic to China and was historically distributed throughout the eastern and central part of the country (Smith et al., 2010; P. Gerngross unpublished data). Shoemaker (1993) suggested that the fur trade impacted leopards from this region less than those in southern China due to preferences for particular fur patterns. This did not relieve them from threat however, as leopards were routinely killed in retaliation for livestock depredation (Shoemaker, 1993). Widespread killing or capturing over decades drove significant population declines (Shoemaker, 1993).

Laguardia et al. (2015) reviewed the current distribution and status of *P. p. japonensis.* Leopards were found at 33 sites in eight provinces: Ningxia, northern Hebei, Shanxi, Shaanxi, northern Henan, western Sichuan, southern Qinghai, and eastern Tibet (whose populations could be *P. p. fusca*). Most populations in these provinces are small (mean size 255 km^2) and occur in isolated nature reserves, therefore it is unknown if they are viable in the long term (Laguardia et al., 2015). There are no confirmed records in other provinces, as recent surveys with transects and camera traps have failed to find evidence of leopards in Hunan, Hubei, Zhejiang, Fujian, Guangxi and Jiangxi (Li et al., 2010; Laguardia et al., 2015). Based on known leopard locations and standard population densities (1-2 individuals/100 km^2), Laguardia et al. (2015) estimated 92-

183 leopards. After including leopards in potentially occupied sites, the population of *P. p. japonensis* is no larger than 350 individuals. However, these population estimates are speculative due to lack of data.

See *P. p. delacouri* – China for information affecting leopards nationally.

Amur Leopard

Panthera pardus orientalis

Northeast Asia

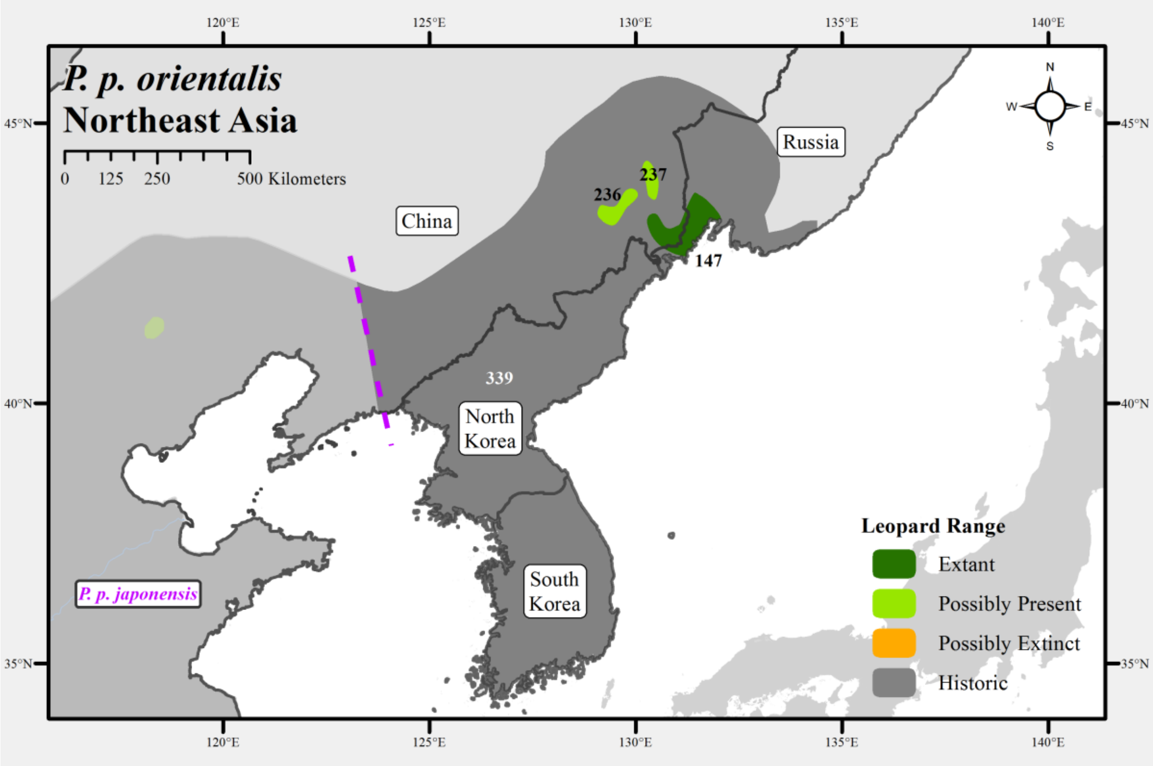

China

Status: Extant

Historically the leopard occurred throughout Liaoning, and the eastern portions of Jilin and Heilongjiang Provinces (Miquelle et al. 2010; Gerngross in prep.) north until Lake Khanka. Miquelle et al. (2010) suggested the Amur leopard formed a meta-population with *P. p. japonensis* with no clear boundaries between the two subspecies. Indeed, they argued a specific boundary between the two subspecies may be a recent construct as range has collapsed. Heptner and Sludskii (1972) suggested that *P. p. orientalis* could have ranged as far south as Beijing. We use a somewhat arbitrary boundary separating the subspecies through the Liaoning Province similar to that in Jutzeler et al. (2010).

After decades of persecution, hardly any Amur leopards remained by the 1970's and 1980's (Shoemaker, 1993). None were recorded in a 1974-1976 survey in Heilongjiang (Shoemaker, 1993). In addition, David Prynn reported "very few" in the Chang Baishan, Kentai Alin, and Lao Yeling Mountain ranges near the Russian and North Korean borders (Shoemaker, 1993). The population in Jilin Province declined from 50 in

the 1970's to less than 10 at the end of the 1990's (Han, 2001). By 1996, the IUCN listed the Amur leopard as critically endangered (Jackson & Nowell, 2008).

After concerted conservation effort, Amur leopard populations are rebounding in China (Qi et al., 2015; Wang et al., 2015; WWF, 2015). The most recent surveying efforts to document the Amur leopard indicated 65-69 individuals across the current range, with 8-12 individuals occupying territories in China (WWF, 2015). Qi et al. (2015) estimated a leopard density of 0.62 individuals/100 km^2 from camera trapping efforts in 2013-2014 in the Southern Laoye Mountains, Jilin Province. Another large-scale camera trap survey across ~6,000 km^2 of northeast China, identified 42 leopards (Wang et al., 2015). The majority of leopards continue to remain in a narrow band along the Russian border although some reproduction is documented in China as far as 50 km from the border (Wang et al., 2015). However, human disturbance and livestock grazing are primary hurdles to rebounding populations (Wang et al., 2015). Continued recovery of the Amur leopard population across its range is highly contingent upon the cooperative transboundary conservation efforts of range countries, primarily Russia and China, but also North Korea (Jutzeler et al., 2010, Wang et al., 2015).

See *P. p. delacouri* – China for information affecting leopards nationally.

North Korea

Status: Possibly Extinct
Last Record: 1998 (Pikunov et al., 2003)

While likely no longer present today (Miquelle et al., 2010), the leopard historically occurred throughout North and South Korea (Miquelle et al., 1996; P. Gerngross unpublished data). Indeed, in the more mountainous regions of North Korea the leopard was quite common before their decline due to uncontrolled hunting and deforestation during the Japanese occupation and Korean War (1910-1953) (Shoemaker, 1993). In the decades following, the leopard population may have remained stable at around 40 individuals although centered in the Hamgyong Province (Shoemaker, 1993). Current information on leopards in North Korea is extremely limited; any remaining habitat for the Amur leopard is likely restricted to the more remote regions bordering China and Russia (Miquelle et al., 2010). Limited resources coupled with high human population densities are the primary limiting factors preventing colonization in northern North Korea (Miquelle et al., 2010).

Shoemaker (1993) referenced an assessment of leopards spanning North Korea, China, and Russia and reported the overall population did not exceed 50 individuals. Investigation and questionnaire surveys estimated 3-5 individuals may persist in the Paektusan region of North Korea (Pikunov et al., 2003), and two leopards were also reported based on interviews with villagers in the southern region of Lyangan Province (Miquelle, 1998). Surveys conducted by Pikunov et al. (2000) in the mountainous Paektusan region of North Korea adjacent to China did not yield evidence of leopard presence. However, more recent and reliable evidence of leopards in North Korea is lacking (Miquelle et al., 2010). Leopards along the Chinese-Korean border (on the Chinese side) are known to occur in Jilin and Heilongjiang Provinces (Qi et al., 2015; WWF, 2015) and may occur in North Korea, but have not been verified (Dale Miquelle pers. comm. 2014).

South Korea

Status: Extinct
Last Record: 1962 (Miquelle et al., 2010)

The leopard historically occurred throughout the Korean Peninsula, including South Korea (P. Gerngross unpublished data) but its distribution and population collapsed during the 20th century (Miquelle et al., 2010). Similar to North Korea, leopards probably declined due to uncontrolled hunting and deforestation during the Japanese occupation and Korean War (Shoemaker, 1993). Shoemaker (1993) also cited prey depletion as a probable cause of decline. The leopard likely went extinct in the 1960's (Shoemaker, 1993; Miquelle et al., 2010) although the exact date is uncertain. Shoemaker (1993) references a leopard captured in 1969 on Odo Mountain, in the South Kyongsang Province before it was moved to the Seoul Zoo where it died in 1973. Miquelle et al. (2010) (likely) reference the same individual, although they indicate the capture occurred in 1962 and the individual died in 1967 before reproducing. Some unconfirmed reports of leopards in South Korea and the demilitarized zone between North and South Korea continue after the 1960's but nothing was substantiated (Miquelle et al., 2010). Won (1988 cited in Shoemaker, 1993) indicated leopard tracks were spotted on Chii Mountain and the Sorak Mountains.

Russia (Far East)

Status: Extant

According to historical reports, the Amur leopard occurred throughout southern Primorsky Krai (Province), in the Sikhote-Alin Mountains south of a line between Lake Khanka and Olga Bay (Arseniev, 1914 cited in Miquelle et al., 2010; P. Gerngross unpublished data). Heptner and Sludskii (1972) cited occasional reports of leopards above this line but they most likely represented dispersing or transient individuals. The IUCN listed the Amur leopard as Endangered in 1994 and Critically Endangered in 1996 (Jackson & Nowell, 2008). Overhunting and habitat loss are primary reasons for the significant declines in leopard distribution and abundance throughout the 20th Century (Miquelle et al., 2010). Yet, even historically the leopard persisted at lower densities than the Amur tiger (*Panthera tigris altaica*) (Miquelle et al., 2010).

Abramov and Pikunov (1974) conducted the first rigorous assessment of leopard numbers in Primorsky Krai over the 1972-1973 winter. They found the leopard already subdivided into three populations totaling between 38-46 individuals. Additionally, Spitzen et al. (2012) reported the extirpation of the Amur leopard from Southern Sikhote-Alin, Pogranichny and Khankaisky counties, in the early 1970's. The leopard was regularly monitored after that (eight additional surveys up to 2007; see Miquelle et al. 2010) although numbers remained below 50 individuals. Leopards disappeared from the two northern sites by 1985 (Pikunov & Korkishko, 1985).

After decades of conservation effort, leopard population and distribution appears to be expanding. A survey in 2013 found 50 individuals (ITAR-Tass, 2013) and a survey in Land of the Leopard National Park (established in 2012) found 57 individuals (WWF 2015) with an additional 8-12 leopards in the surrounding area of China.

Preservation of additional sites in China and Russia is vital to the long-term recovery of the species (Jutzeler et al., 2010). Hebblewhite et al. (2011) modeled habitat suitability in southern Primorsky Krai and identified eight potential habitat patches totaling ~10,500 km^2 of habitat that could hold an additional potential population of 105 leopards. They additionally found a strong positive relationship of leopards with prey availability and Korean pine habitat while a negative relationship with deciduous forests, meadows, and agricultural areas (Hebblewhite et al., 2011).

Javan Leopard

Panthera pardus melas

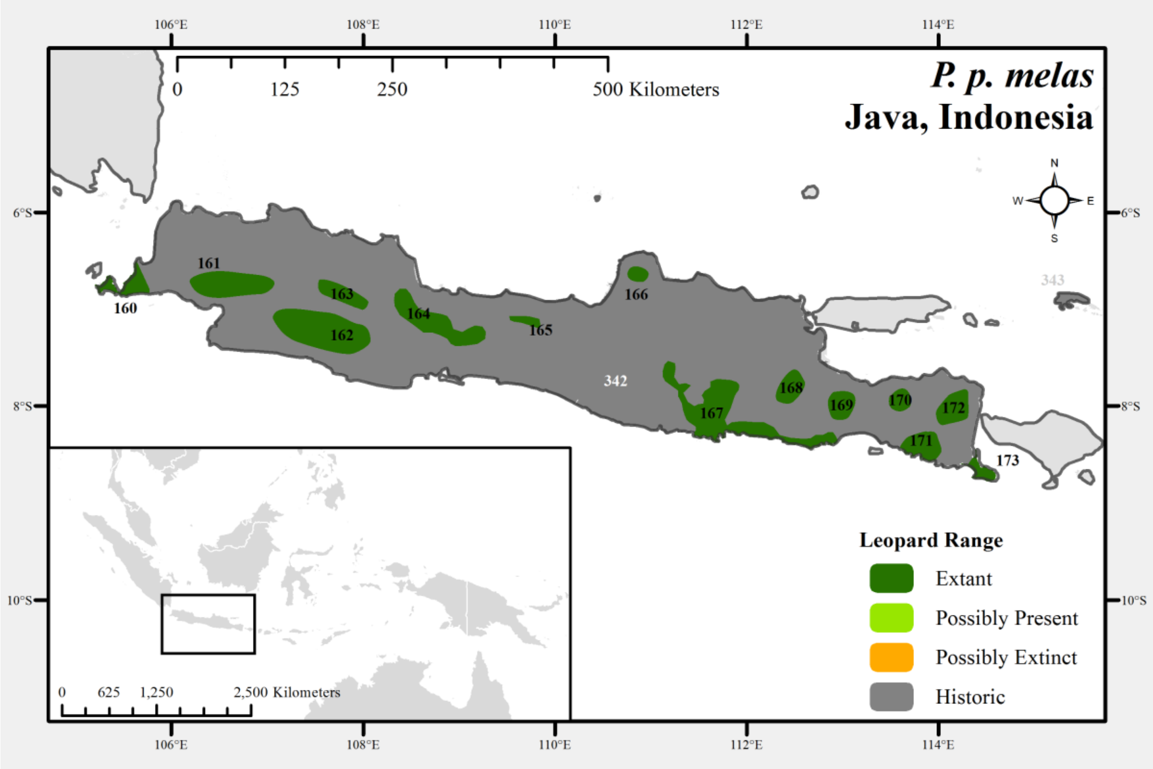

Indonesia (Java)

Status: Extant

Despite its highly fragmented distribution today, the Javan leopard historically occurred widely and abundantly throughout the island of Java (Hoogerwerf, 1970; P. Gerngross unpublished data). The Javan leopard is intriguingly only found on Java and not on the other islands between it and mainland Asia (Sumatra and Borneo). Meijaard (2004) speculated that leopards reached Java via a land bridge that bypassed the other islands during the Middle Pleistocene and were subsequently unable to colonize the other islands during the last glacial maximum. Additionally, leopards may be historically present on the Kangean Islands, ~ 125 km to the northeast of Java although they have not been recorded on the much closer Madura or Bali islands (Direktorat PPA, 1978; 1982; Van Helvoort, de Iongh & van Bree, 1985). There was an isolated incident of a leopard killed after being caught in a snare ~4-5 km southwest of Torjek village in 1983, and while an abundance of prey was detected (Van Helvoort, de Iongh & van Bree, 1985), no further evidence of leopards has been documented on the Kangean Islands since then (Peter Gerngross pers. comm. 2002).

Java has one of the densest human populations on the world, and had lost more than 90% of its natural habitat by the early 1990's (Santiapillai & Ramono, 1992). Tigers went extinct in the mid 1970's but the adaptable leopard persisted. Santiapillai and Ramono (1992) documented 12 reserves where leopards occurred and

noted the leopard could be more widely distributed pending field surveys. Santiapillai and Ramono (1992) estimated in the early 1990's there could be 350-700 leopards remaining in conservation areas based off extrapolation of density estimates (10 to 20 individuals/100 km^2). However, leopard populations have likely shrunk considerably over the last 20 years concurrent with a reduction in range (Gunawan, 2009). Major ongoing threats to the leopard are human population growth, habitat loss, and poaching/illegal killing (Anonymous 2013). Indeed, in a 16-year period (1990-2006), Central Java Province lost nearly 450,000 ha (88%) of its dry land natural forest (Gunawan et al., 2010), likely leading to the local extirpation of 17 leopard subpopulations (Gunawan et al., 2013). Habitat loss may be spurring human-leopard conflict as well. Since 2001, 71 cases of conflict were recorded in western Java (Hendra Gunawan pers. comm. 2015). The events often begin with livestock predation and end with the death or capture of the leopard.

Ario (2007) estimated leopard range had shrunk to roughly 2,267 - 3,277 km^2 by the mid 2000's. Extrapolating from a few known densities led to a countrywide estimate of 363-525 individuals (Ario, 2007). Using camera traps, Ario and Hidayat (2009) estimated a density of between 5 and 13 individuals/100 km^2 in Gunung Gede-Pangrango National Park. Ario (2007) found slightly higher density estimates at Gunung Halimun National Park (15 individuals/100 km^2).

A 2009 survey by Gunawan et al (2012) suggested the leopard was more widely distributed through the forest plantations of central Java than otherwise thought. More recently the Indonesian Ministry of Forestry has written a *Strategy and Action Plan for Conservation of the Javan Leopard 2013 – 2023* (Anonymous, 2013). This plan identifies key threats, current distribution and population estimates, and lays out a conservation strategy. The Action Plan identified leopards in 34 known locations including national parks, conservation areas, and remaining forests and mountain habitats. Five sites used camera trap to identify presence and estimate density including Gunung Gede Pangrango, Gunung Halimun-Salak, Gunung Ceremai National Parks, and Gunung Salak. Populations for the remaining sites were estimated from field signs and interviews. Summing these results led to a total population of 681 individuals.

An additional site not listed in the Action Plan is Alas Purwo National Park where two leopards were camera trapped in 2013 by Bengt Holst (Secher, 2013).

Table 1. Patch name and IDs across leopard range

Patch ID	Patch Name	Subspecies	Presence	Area (km2)	Prot area km2 (cat. 1 -4)	Prot area %	Mean human pop'n density	Transboundary	Countries
1	Niokolo Koba and Guinea	pardus	Extant	61,000	7,700	13%	11.8	y	Guinea, Guinea-Bissau, Mali, Senegal
2	Upper Niger National Park	pardus	Extant	10,000	600	6%	11.5	n	Guinea
3	Foya, Gola and forests, northern Liberia	pardus	Extant	10,300	300	3%	7.5	y	Liberia, Sierra Leone
4	Tai forest	pardus	Extant	27,000	5,600	21%	30.9	y	Ivory Coast, Liberia
5	Comoe and Mole	pardus	Extant	40,900	15,800	39%	9.7	y	Burkina Faso, Ivory Coast, Ghana
6	n Ghana and s Burkina; Kabore-Tambi	pardus	Extant	8,300	1,000	12%	33.2	y	Ghana, Burkina Faso
7	W-Arly-Pendjari	pardus	Extant	24,700	13,100	53%	21.3	y	Benin, Burkina Faso, Niger
8	Kainji and Trois Rivieres	pardus	Extant	8,600	3,000	35%	21.9	y	Benin, Nigeria
9	Benoue ecosystem	pardus	Extant	51,900	12,300	24%	11.1	y	Cameroon, Nigeria, Chad
10	Mbam et Djerem	pardus	Extant	9,400	3,800	40%	4.1	n	Cameroon
11	Campo Ma'an	pardus	Extant	8,200	2,500	30%	11.8	y	Cameroon, Equatorial Guinea
12	West Congo basin	pardus	Extant	754,100	89,100	12%	6.0	y	Angola, CAR, Cameroon, DRC, Republic of the Congo, Gabon, Equatorial Guinea
13	Lomako-Yokokala	pardus	Extant	13,400	-	0%	5.5	n	DRC
14	Central DRC	pardus	Extant	405,400	62,300	15%	6.0	n	DRC
15	Eastern Central Africa Republic	pardus	Extant	690,300	133,000	19%	3.4	y	CAR, DRC, Sudan, South Sudan, Chad
16	Gebel Elba	pardus	Extant	12,700	-	0%	0.6	y	Egypt, Sudan
17	Eritrean highlands	pardus	Extant	22,800	1,700	7%	39.4	y	Eritrea
18	northern Ethiopia	pardus	Extant	104,600	8,800	8%	54.6	y	Ethiopia, Sudan
19	Mousa Ali mountain Djibouti	pardus	Extant	800	-	0%	7.0	y	Djibouti, Eritrea, Ethiopia
20	Day forest Djibouti	pardus	Extant	1,200	-	0%	26.7	n	Djibouti
21	central Ethiopia highlands	pardus	Extant	6,300	-	0%	673.7	n	Ethiopia
22	Abijatta-Shalla	pardus	Extant	8,000	1,800	23%	227.5	n	Ethiopia

23	Boma-Gambella & southern Ethipia	pardus	Extant	280,900	51,600	18%	34.5	y	Ethiopia, Kenya, South Sudan
24	Awash and Yangudi Rassa	pardus	Extant	40,400	7,600	19%	51.9	n	Ethiopia
25	Gaan Libaax and eastern Ethiopia	pardus	Extant	33,200	-	0%	41.6	y	Ethiopia, Somalia
26	Daalo forest Somalia	pardus	Extant	6,700	-	0%	8.5	n	Somalia
27	Eastern Somalia montane xeric woodlands	pardus	Extant	3,200	-	0%	3.6	n	Somalia
28	northern and eastern Uganda	pardus	Extant	72,300	13,600	19%	46.4	y	Kenya, South Sudan, Uganda
29	greater Virunga	pardus	Extant	15,300	8,800	58%	85.2	y	DRC, Uganda
30	Akagera, Rumanyika and Lake Mburo	pardus	Extant	10,800	2,100	19%	126.3	y	Rwanda, Tanzania, Uganda
31	Kakamega forest	pardus	Extant	2,000	-	0%	451.3	n	Kenya
32	Kenya, Tanzania, N Mozambique	pardus	Extant	1,153,200	236,000	20%	36.0	y	Kenya, Mozambique, Somalia, Tanzania
33	Quicama Angola	pardus	Extant	4,400	3,800	86%	7.2	n	Angola
34	central Southern Africa	pardus	Extant	1,926,100	334,900	17%	3.3	y	Angola, Botswana, DRC, Mozambique, Malawi, Namibia, South Africa, Zambia, Zimbabwe
35	Kalahari savanna SE Namibia	pardus	Extant	2,800	-	0%	0.2	n	Namibia
36	Kalahari savanna eastern Namibia	pardus	Extant	1,000	-	0%	1.1	n	Namibia
37	Upemba and Kundelungu Zambia	pardus	Extant	28,900	20,300	70%	15.9	n	DRC
38	Kafue ecosystem	pardus	Extant	62,500	22,100	35%	3.2	n	Zambia
39	Nsumbu and N Zambia	pardus	Extant	11,200	3,300	29%	8.8	n	Zambia
40	Bangweulu swamps and GMAs	pardus	Extant	14,100	2,000	14%	4.0	n	Zambia
41	Nkhota-Kota Malawi	pardus	Extant	2,200	1,500	68%	30.5	n	Malawi
42	Majete Malawi	pardus	Extant	1,100	800	73%	21.3	n	Malawi
43	Gile and coastal Mozambique	pardus	Extant	26,600	2,800	11%	25.7	n	Mozambique
44	Umfurudzi safari area Zimbabwe	pardus	Extant	900	-	0%	9.9	n	Zimbabwe
45	Marromeu and central Mozambique	pardus	Extant	61,800	5,600	9%	20.0	y	Mozambique, Zimbabwe
46	Zimbabwe unprotected lands	pardus	Extant	3,500	-	0%	45.0	n	Zimbabwe
47	southern Zimbabwe unprotected lands 2	pardus	Extant	3,900	-	0%	8.2	n	Zimbabwe

48	southern Zimbabwe unprotected lands	pardus	Extant	1,100	-	0%	16.4	n	Zimbabwe
49	Kyle and Mushandike sanctuaries	pardus	Extant	2,000	100	5%	36.8	n	Zimbabwe
50	Kruger and eastern Southern Africa	pardus	Extant	436,000	47,300	11%	26.4	y	Botswana, Mozambique, South Africa, Swaziland, Zimbabwe
51	Drakensberg mountains	pardus	Extant	9,600	200	2%	25.9	y	Lesotho, South Africa
52	KwaZulu-Cape forest mosaic	pardus	Extant	15,800	-	0%	83.9	n	South Africa
53	southern South Africa	pardus	Extant	99,900	1,700	2%	19.7	n	South Africa
54	Jabal Shada Saudi Arabia	nimr	Extant	3,000	-	0%	88.1	n	Saudi Arabia
55	Raydah Saudi Arabia	nimr	Extant	1,300	-	0%	155.8	n	Saudi Arabia
56	Jazan Saudi Arabia	nimr	Extant	400	-	0%	9.8	n	Saudi Arabia
57	transboundary Yemen Saudi	nimr	Extant	500	-	0%	207.1	y	Saudi Arabia, Yemen
58	Hajjah Yemen	nimr	Extant	2,700	-	0%	346.7	n	Yemen
59	Lahijj Yemen	nimr	Extant	1,500	-	0%	55.0	n	Yemen
60	Jabal Samhan and coastal Oman	nimr	Extant	8,000	1,500	19%	23.4	y	Oman, Yemen
61	Greater Caucasus mountains, N Ossetia	saxicolor	Extant	3,000	1,000	33%	8.2	y	Georgia, Russian Federation
62	Greater Caucasus mountains, Dagestan	saxicolor	Extant	5,300	1,500	28%	8.5	y	Georgia, Russian Federation
63	Lesser Caucasus, transboundary	saxicolor	Extant	9,600	4,200	44%	16.0	y	Armenia, Azerbaijan, Iran
64	Alborz	saxicolor	Extant	198,600	37,800	19%	66.5	y	Azerbaijan, Iran, Turkmenistan
65	N of Touran	saxicolor	Extant	1,300	100	8%	1.1	n	Iran
66	Touran	saxicolor	Extant	3,400	3,000	88%	0.6	n	Iran
67	Kavir	saxicolor	Extant	1,900	1,900	100%	0.0	n	Iran
68	S Khorasan	saxicolor	Extant	3,000	600	20%	9.0	n	Iran
69	Abbas Abad 2	saxicolor	Extant	1,500	1,500	100%	0.0	n	Iran
70	Abbas Abad 1	saxicolor	Extant	1,000	700	70%	0.0	n	Iran
71	Yazd	saxicolor	Extant	32,500	6,900	21%	2.3	n	Iran
72	Eastern Naybandan	saxicolor	Extant	600	600	100%	0.2	n	Iran
73	Zagros Mountains in Iraq/Turkey	saxicolor	Extant	44,900	2,000	4%	51.0	y	Iran, Iraq, Turkey
74	Zagros mountains	saxicolor	Extant	229,200	38,200	17%	28.9	n	Iran
75	Esfahan	saxicolor	Extant	1,100	500	45%	36.5	n	Iran

76	Hormozgan	saxicolor	Extant	11,400	-	0%	3.6	n	Iran
77	Baluchestan	saxicolor	Extant	40,000	5,600	14%	10.0	n	Iran
78	south of Bazman	saxicolor	Extant	4,300	1,700	40%	1.9	n	Iran
79	Balochistan Iran and Kuh-e-Birk	saxicolor	Extant	1,600	-	0%	6.8	n	Iran
80	southern Central Makran mountain Pakistan	saxicolor	Extant	2,000	-	0%	18.5	n	Pakistan
81	Ajar Valley Afghanistan	saxicolor	Extant	5,700	800	14%	8.4	n	Afghanistan
82	Himalaya mountains	fusca	Extant	488,900	69,700	14%	114.1	y	Bhutan, China, India, Myanmar, Nepal, Pakistan
83	Barda, Gujarat, India	fusca	Extant	1,600	200	13%	160.8	n	India
84	Gir, India	fusca	Extant	10,600	1,600	15%	322.0	n	India
85	Mount Abu, Rajasthan, India	fusca	Extant	16,200	1,700	10%	153.1	n	India
86	Sanjay Gandhi and West coastal India	fusca	Extant	5,700	100	2%	2904.9	n	India
87	SE Gujarat, W Maharashtra, India	fusca	Extant	35,800	800	2%	444.1	n	India
88	Dhumkal and Ratanmahal, India	fusca	Extant	5,000	300	6%	152.3	n	India
89	western Vindhya range, India	fusca	Extant	4,000	-	0%	159.4	n	India
90	Sariska, India	fusca	Extant	1,200	600	50%	114.2	n	India
91	southern Haryana, India	fusca	Extant	500	-	0%	333.5	n	India
92	Ranthambore Palpur-Kuno, India	fusca	Extant	7,200	400	6%	113.3	n	India
93	north of Guna, India	fusca	Extant	800	-	0%	91.1	n	India
94	Gugamal and Melghat, India	fusca	Extant	9,600	1,900	20%	73.7	n	India
95	Vindhya range, Noradehi, India	fusca	Extant	12,300	1,700	14%	128.9	n	India
96	Bori, Pachmarhi, Satpura, India	fusca	Extant	14,300	1,400	10%	127.4	n	India
97	Pench, India	fusca	Extant	2,200	1,000	45%	57.0	n	India
98	eastern Maharashtra, Andhari, India	fusca	Extant	19,000	900	5%	134.5	n	India
99	Panna, India	fusca	Extant	5,000	800	16%	91.3	n	India
100	Bandhavgarh, Panpatha, India	fusca	Extant	9,600	700	7%	135.5	n	India
101	Kanha, Achanakmar, India	fusca	Extant	21,100	1,600	8%	87.6	n	India
102	Sanjay and Kaimur, India	fusca	Extant	22,000	4,200	19%	127.8	n	India
103	Palamau, India	fusca	Extant	4,600	1,000	22%	90.4	n	India
104	Hazaribagh, India	fusca	Extant	2,000	500	25%	374.5	n	India

105	Sitanadi, Sunabeda, India	fusca	Extant	12,800	1,500	12%	107.1	n	India
106	Barnawapara to Debrigarh, India	fusca	Extant	5,500	800	15%	131.3	n	India
107	Hemgir forest range, India	fusca	Extant	3,800	-	0%	120.0	n	India
108	central Odisha forests, India	fusca	Extant	29,700	1,400	5%	120.7	n	India
109	Lakhari Valley, India	fusca	Extant	1,000	200	20%	102.7	n	India
110	northern Odisha forest, Kendujhar India	fusca	Extant	4,500	-	0%	192.6	n	India
111	Ghatgaon forest, India	fusca	Extant	1,000	-	0%	176.9	n	India
112	Simlipal, India	fusca	Extant	5,500	2,500	45%	111.2	n	India
113	northern Telangana, Kawal, India	fusca	Extant	19,200	1,300	7%	152.0	n	India
114	Indravati and Bhairamgarh, India	fusca	Extant	4,700	1,000	21%	14.8	n	India
115	Kangar Valley, India	fusca	Extant	1,300	100	8%	88.1	n	India
116	Pakhal, Eturnagaram, Kinnerasni, India	fusca	Extant	13,700	1,800	13%	117.8	n	India
117	Kondapelle forest, eastern Ghats, India	fusca	Extant	300	-	0%	212.4	n	India
118	Papikonda, Alluri Sitaramaraju, India	fusca	Extant	18,000	500	3%	243.5	n	India
119	western Ghats, India	fusca	Extant	146,800	13,000	9%	290.8	n	India
120	central Karnataka, India	fusca	Extant	600	-	0%	127.9	n	India
121	eastern Karnataka, India	fusca	Extant	1,800	-	0%	265.8	n	India
122	southern Karnataka, India	fusca	Extant	3,500	-	0%	290.1	n	India
123	southeastern Karnataka, India	fusca	Extant	1,400	-	0%	371.9	n	India
124	Gundla Brahmeswaram India	fusca	Extant	7,600	500	7%	238.3	n	India
125	Nagarjunasagar to Sri Venkateswara	fusca	Extant	36,000	100	0%	141.5	n	India
126	W of Pabitora, Assam	fusca	Extant	1,300	-	0%	982.4	n	India
127	Hukaung Valley	fusca	Extant	3,000	3,000	100%	1.2	n	Myanmar
128	Chittagong and Chin hills	fusca	Extant	25,900	1,600	6%	42.0	y	Bangladesh, India, Myanmar
129	Alaungdaw & western Myanmar	fusca	Extant	15,200	1,600	11%	26.8	n	Myanmar
130	Tangup Pass and Rakhine Yoma, Myanmar	fusca	Extant	3,100	-	0%	23.8	n	Myanmar
131	central Sri Lanka	kotiya	Extant	18,300	8,600	47%	136.9	n	Sri Lanka
132	sw Sri Lanka	kotiya	Extant	800	100	13%	282.7	n	Sri Lanka
133	Yala, se Sri Lanka	kotiya	Extant	5,300	3,500	66%	37.3	n	Sri Lanka

134	Tibetan plateau south	japonensis	Extant	12,200	4,800	39%	6.1	n	China
135	Tibetan plateau north	japonensis	Extant	7,500	2,100	28%	4.7	n	China
136	western Sichuan & Yuke	japonensis	Extant	8,500	1,200	14%	4.8	n	China
137	western Sichuan	japonensis	Extant	2,900	-	0%	4.6	n	China
138	NIngxia & Gansu	japonensis	Extant	900	800	89%	33.7	n	China
139	W of Xi'an, Shaanxi province	japonensis	Extant	1,800	-	0%	55.3	n	China
140	Zhouzhijinsihou and Qinling Mtns	japonensis	Extant	6,000	1,600	27%	9.5	n	China
141	S of Yan'an	japonensis	Extant	2,700	-	0%	7.8	n	China
142	Fanjia mountain; Shanxi	japonensis	Extant	2,800	200	7%	113.4	n	China
143	Wulushan	japonensis	Extant	3,400	200	6%	45.4	n	China
144	Lishan, southern Shanxi	japonensis	Extant	6,800	800	12%	53.1	n	China
145	Taihang mountains	japonensis	Extant	9,800	-	0%	45.1	n	China
146	Xiaowutaishan to Baihuashan	japonensis	Extant	2,700	300	11%	18.7	n	China
147	Primorsky Krai and eastern China	orientalis	Extant	8,100	1,800	22%	6.1	y	China, Russian Federation
148	Nangunhe	delacouri	Extant	2,200	600	27%	58.2	n	China
149	Xishuangbanna	delacouri	Extant	2,300	2,200	96%	42.4	y	China, Lao PDR
150	northern Karen (Kayin)	delacouri	Extant	3,700	-	0%	6.0	n	Myanmar
151	Southern Forest Complex	delacouri	Extant	42,600	19,000	45%	9.2	y	Myanmar, Thailand
152	Northern Plains landscape	delacouri	Extant	1,900	1,000	53%	9.7	y	Cambodia, Thailand
153	Mondulkiri and Phnom Prich, Yok Don	delacouri	Extant	13,400	8,500	63%	5.4	n	Cambodia
154	southern Lenya	delacouri	Extant	1,000	100	10%	3.8	y	Myanmar, Thailand
155	Khao Sok, Thailand	delacouri	Extant	4,000	2,700	68%	14.4	n	Thailand
156	Belum-Temengor	delacouri	Extant	5,800	600	10%	14.2	y	Malaysia, Thailand
157	Taman Negara	delacouri	Extant	8,900	4,500	51%	2.8	n	Malaysia
158	N of Endau Rompin	delacouri	Extant	900	-	0%	49.2	n	Malaysia
159	Endau Rompin (Johor)	delacouri	Extant	3,700	1,300	35%	4.9	n	Malaysia
160	Ujung Kulon	melas	Extant	800	400	50%	178.3	n	Indonesia
161	Halimun-Salak to Gede-Pangrango	melas	Extant	2,300	900	39%	234.2	n	Indonesia
162	Gunung Simpang, Papndayan	melas	Extant	3,900	400	10%	470.6	n	Indonesia
163	Burangrang to Masigit Kareumbi	melas	Extant	1,000	-	0%	1039.4	n	Indonesia
164	Gunung Ciremai to Slamat	melas	Extant	2,400	100	4%	472.0	n	Indonesia

165	Pekalongan and Batang forests,	melas	Extant	300	-	0%	83.2	n	Indonesia
166	Gunung Muria	melas	Extant	300	-	0%	85.7	n	Indonesia
167	Gunung Lawu and Wilis, southern coast	melas	Extant	4,600	-	0%	320.2	n	Indonesia
168	Raden Suryo	melas	Extant	900	-	0%	321.3	n	Indonesia
169	Bromo Tengger Semeru	melas	Extant	800	400	50%	76.6	n	Indonesia
170	Dataran Tinggi Yang	melas	Extant	500	100	20%	14.8	n	Indonesia
171	Gunung Betiri	melas	Extant	1,100	500	45%	162.1	n	Indonesia
172	kawah Ijen and east Java	melas	Extant	1,100	-	0%	40.8	n	Indonesia
173	Alas Purwo	melas	Extant	400	400	100%	75.4	n	Indonesia
174	northern Sierra Leone & s Guinea	pardus	Possibly Present	3,400	-	0%	13.9	y	Guinea, Sierra Leone
175	east of Mount Bintumani	pardus	Possibly Present	1,600	-	0%	19.7	y	Guinea, Sierra Leone
176	Tassili-n-Ajjer, montane xeric woodlands	pardus	Possibly Present	3,600	-	0%	0.0	n	Algeria
177	central unprotected Cameroon	pardus	Possibly Present	60,400	1,900	3%	12.5	y	Cameroon, CAR, Chad
178	Red Sea coast	pardus	Possibly Present	11,200	-	0%	52.6	n	Sudan
179	northern Angola near mouth of Congo	pardus	Possibly Present	15,400	-	0%	12.5	n	Angola
180	southern Zaire, Angola	pardus	Possibly Present	2,900	-	0%	4.8	n	Angola
181	Liuwa Plains	pardus	Possibly Present	8,500	3,200	38%	5.0	n	Zambia
182	Lavushi Manda	pardus	Possibly Present	7,000	1,800	26%	2.7	n	Zambia
183	west central Kataanga province	pardus	Possibly Present	5,200	-	0%	5.5	n	DRC
184	sw Maniema province	pardus	Possibly Present	13,800	-	0%	7.6	n	DRC
185	Itombwe and Luama-Kivu	pardus	Possibly Present	20,000	-	0%	24.4	n	DRC
186	Ruvubu	pardus	Possibly Present	900	500	56%	127.7	n	Burundi
187	Lower Shabelle	pardus	Possibly Present	5,600	-	0%	21.8	n	Somalia
188	Lusenga Plain	pardus	Possibly Present	2,600	900	35%	13.5	n	Zambia
189	northern Zambezi province	pardus	Possibly Present	8,700	-	0%	42.2	n	Mozambique
190	Negev Highlands	nimr	Possibly Present	900	300	33%	5.8	n	Israel
191	west of Medina	nimr	Possibly Present	3,400	-	0%	0.4	n	Saudi Arabia
192	Greater Caucasus mountains	saxicolor	Possibly Present	3,000	1,500	50%	1.3	y	Georgia, Russian Federation

193	W Lesser Caucasus, Iran	saxicolor	Possibly Present	1,400	1,300	93%	15.9	y	Azerbaijan, Iran
194	Lesser Caucasus, northern Iran	saxicolor	Possibly Present	6,500	1,300	20%	17.4	n	Iran
195	E of Lake Urumieh	saxicolor	Possibly Present	800	500	63%	16.8	n	Iran
196	northern Zagros mountains - Ghalajeh	saxicolor	Possibly Present	4,500	800	18%	23.9	n	Iran
197	Mooteh	saxicolor	Possibly Present	2,900	2,000	69%	5.3	n	Iran
198	Karkas	saxicolor	Possibly Present	3,400	1,200	35%	11.0	n	Iran
199	W of Kalmand	saxicolor	Possibly Present	1,700	400	24%	16.6	n	Iran
200	N of Kerman	saxicolor	Possibly Present	40,200	3,700	9%	27.3	n	Iran
201	S of Kerman	saxicolor	Possibly Present	9,400	200	2%	8.3	n	Iran
202	Eastern Hormozgan	saxicolor	Possibly Present	14,700	1,200	8%	11.8	n	Iran
203	Southern Dasht-e Kavir Desert	saxicolor	Possibly Present	1,500	300	20%	0.9	n	Iran
204	Western south Khorasan	saxicolor	Possibly Present	900	400	44%	0.3	n	Iran
205	S Khorasan possibly present	saxicolor	Possibly Present	8,900	1,800	20%	6.1	n	Iran
206	Balochistan Iran and Kuh-e-Birk	saxicolor	Possibly Present	2,500	-	0%	1.5	n	Iran
207	eastern Iran unprotected land, Zahedan	saxicolor	Possibly Present	4,800	-	0%	100.0	n	Iran
208	Balochistan Iran and Kuh-e-Birk	saxicolor	Possibly Present	800	-	0%	6.6	n	Iran
209	connection between Gando & Kuh-e-Birk	saxicolor	Possibly Present	2,200	-	0%	15.1	n	Iran
210	NW Afghanistan wildlife managed reserve	saxicolor	Possibly Present	17,400	400	2%	10.3	y	Afghanistan, Turkmenistan
211	Hindu Kush saxicolor	saxicolor	Possibly Present	182,600	400	0%	48.9	y	Afghanistan, Pakistan
212	Kugitang Mountains	saxicolor	Possibly Present	1,200	700	58%	1.7	y	Turkmenistan, Uzbekistan
213	Babatag Mountains	saxicolor	Possibly Present	2,700	-	0%	7.9	n	Uzbekistan
214	northern Badakhshan	saxicolor	Possibly Present	1,700	-	0%	9.2	n	Afghanistan
215	Sulaiman mountains	saxicolor	Possibly Present	9,900	-	0%	82.9	y	Afghanistan, Pakistan
216	Hindu Kush saxicolor	saxicolor	Possibly Present	6,400	-	0%	199.4	y	Afghanistan, Pakistan
217	connxn between Chittagong and Himalayas	fusca	Possibly Present	72,600	1,600	2%	84.1	y	India, Myanmar
218	Bumhpabum Myanmar	fusca	Possibly Present	1,100	200	18%	0.2	n	Myanmar

219	Pidaung Myanmar	fusca	Possibly Present	800	100	13%	3.0	n	Myanmar
220	south Chittagong and Chin hills	fusca	Possibly Present	6,600	200	3%	106.6	y	Bangladesh, Myanmar
221	northern Shan state	delacouri	Possibly Present	4,000	-	0%	7.8	n	Myanmar
222	central Myanmar	delacouri	Possibly Present	9,500	300	3%	18.2	n	Myanmar
223	N of Pegu Yomas	delacouri	Possibly Present	4,600	-	0%	8.4	n	Myanmar
224	N of Huai Kha Khaeng complex	delacouri	Possibly Present	14,200	100	1%	86.8	n	Myanmar
225	W of Huai Kha Khaeng complex	delacouri	Possibly Present	4,400	-	0%	11.6	n	Myanmar
226	N of Lenya NP	delacouri	Possibly Present	3,200	500	16%	14.8	n	Myanmar
227	central mainland Malaysia	delacouri	Possibly Present	48,800	1,300	3%	58.7	n	Malaysia
228	Endau-Kota Tinggi (west)	delacouri	Possibly Present	1,800	-	0%	14.9	n	Malaysia
229	Baishuijiang and southern Gansu	japonensis	Possibly Present	3,500	1,300	37%	36.4	n	China
230	unprotected land, Shaanxi province	japonensis	Possibly Present	8,300	200	2%	34.3	n	China
231	northern Guizhou	japonensis	Possibly Present	9,100	500	5%	174.7	n	China
232	southern Wuyi mountains	japonensis	Possibly Present	16,700	800	5%	80.3	n	China
233	n Fujian and s Zhejiang	japonensis	Possibly Present	12,100	400	3%	60.6	n	China
234	north of Po Yang Hu	japonensis	Possibly Present	4,100	100	2%	81.2	n	China
235	Helihe in northern Hebei	japonensis	Possibly Present	1,400	400	29%	9.6	n	China
236	northern Jilin province	orientalis	Possibly Present	2,500	-	0%	20.8	n	China
237	southern Heilongjiang	orientalis	Possibly Present	1,600	-	0%	11.6	n	China
238	Bou tferda, Middle Atlas	pardus	Possibly Extinct	1,100	-	0%	20.9	n	Morocco
239	western Saharan Atlas Mountains	pardus	Possibly Extinct	26,600	-	0%	10.7	y	Algeria, Morocco
240	Coastal Senegal & Guinea to Baoule	pardus	Possibly Extinct	55,700	900	2%	40.6	y	Guinea, The Gambia, Guinea-Bissau Senegal
241	W Mali	pardus	Possibly Extinct	55,000	2,700	5%	10.9	y	Guinea, Mali, Senegal
242	Gori HIlls and eastern Sierra Leone	pardus	Possibly Extinct	1,600	-	0%	44.4	n	Sierra Leone
243	northern Liberia & Massif du Ziama	pardus	Possibly Extinct	18,900	300	2%	26.9	y	Guinea, Liberia, Sierra Leone
244	surroundings of Tai forest	pardus	Possibly Extinct	31,100	900	3%	36.7	y	Liberia, Ivory Coast
245	Diecke	pardus	Possibly Extinct	1,400	-	0%	35.9	y	Guina, Liberia

246	NImba & other forests, w Ivory Coast	pardus	Possibly Extinct	2,800	300	11%	57.6	y	Guinea, Ivory Coast, Liberia
247	forests of western Ivory Coast	pardus	Possibly Extinct	42,100	1,600	4%	24.9	y	Guinea, Ivory Coast
248	Surroundings of Comoe & Mole	pardus	Possibly Extinct	33,600	2,200	7%	19.0	y	Burkina Faso, Ivory Coast, Ghana
249	coastal Guinean forests	pardus	Possibly Extinct	25,100	800	3%	52.7	y	Ghana, Ivory Coast
250	Digya	pardus	Possibly Extinct	3,200	2,200	69%	6.9	n	Ghana
251	western Nigeria forests, Kouffe	pardus	Possibly Extinct	4,400	-	0%	11.9	n	Benin
252	Okomu sanctuary	pardus	Possibly Extinct	1,700	1,100	65%	51.6	n	Nigeria
253	Cross River	pardus	Possibly Extinct	5,700	2,800	49%	26.9	y	Cameroon, Nigeria
254	Kambari forest reserve	pardus	Possibly Extinct	600	400	67%	13.6	n	Nigeria
255	central Central Africa Republic	pardus	Possibly Extinct	202,000	2,300	1%	14.0	y	CAR, Chad, DRC
256	South Sudan, Ethiopia, N Kenya	pardus	Possibly Extinct	909,300	9,000	1%	40.8	y	CAR, Chad, Ethiopia, Kenya, South Sudan, Sudan, Uganda
257	unprotected land W of Tana River	pardus	Possibly Extinct	15,800	200	1%	6.3	n	Kenya
258	western Angola	pardus	Possibly Extinct	416,300	21,400	5%	15.3	y	Angola, DRC
259	DRC near mouth of Congo river	pardus	Possibly Extinct	4,700	-	0%	41.1	n	DRC
260	Bombo Lumene	pardus	Possibly Extinct	6,300	-	0%	22.5	n	DRC
261	western DRC forest-savanna mosaic	pardus	Possibly Extinct	9,500	-	0%	7.1	y	Congo, DRC
262	western DRC lowland forests	pardus	Possibly Extinct	14,300	-	0%	5.8	n	DRC
263	central DRC lowland forests	pardus	Possibly Extinct	178,500	-	0%	16.2	n	DRC
264	Btwn Maikia-Penge & Garamba	pardus	Possibly Extinct	8,200	-	0%	10.2	n	DRC
265	east of Okapi reserve	pardus	Possibly Extinct	5,900	-	0%	23.3	n	DRC
266	Tanya & Kisimba Ikobo	pardus	Possibly Extinct	7,000	500	7%	19.0	n	DRC
267	central Uganda and nw Tanzania	pardus	Possibly Extinct	112,600	4,300	4%	162.4	y	DRC, Tanzania, Uganda
268	central DRC forest-savanna mosaic	pardus	Possibly Extinct	41,700	-	0%	14.8	n	DRC
269	central DRC forest-savanna & Sankuru	pardus	Possibly Extinct	23,800	-	0%	6.0	n	DRC
270	west of Luama-Kivu	pardus	Possibly Extinct	25,100	-	0%	12.2	n	DRC
271	Nyungwe Rwanda	pardus	Possibly Extinct	4,000	1,300	33%	270.5	y	Burundi, Rwanda

272	sw border of DRC - Bushimaie	pardus	Possibly Extinct	51,300	-	0%	12.2	y	Angola, DRC
273	DRC Zambezian miombo woodlands	pardus	Possibly Extinct	2,300	-	0%	2.5	n	DRC
274	DRC Zambezian miombo woodlands 2	pardus	Possibly Extinct	5,000	-	0%	3.2	n	DRC
275	DRC Zambezian miombo - Kataanga	pardus	Possibly Extinct	42,700	-	0%	6.1	n	DRC
276	Zambia	pardus	Possibly Extinct	507,400	2,400	0%	15.0	y	Angola, DRC, Malawi, Tanzania, Zambia
277	southern Okavango and Xai Xai	pardus	Possibly Extinct	39,400	-	0%	0.9	n	Botswana
278	southern Africa unprotected lands	pardus	Possibly Extinct	491,600	3,100	1%	28.1	y	Botswana, Malawi, Mozambique, South Africa, Zambia, Zimbabwe
279	Judean Desert	nimr	Possibly Extinct	300	-	0%	66.8	n	Israel
280	n of Hingol NP	saxicolor	Possibly Extinct	7,300	200	3%	3.5	n	Pakistan
281	Central Makran mtns, S	saxicolor	Possibly Extinct	3,000	-	0%	1.8	n	Pakistan
282	Central Makran mtns	saxicolor	Possibly Extinct	3,300	-	0%	2.5	n	Pakistan
283	Central Makran mtns, E	saxicolor	Possibly Extinct	1,900	-	0%	10.4	n	Pakistan
284	Koh-e-Geish to Dhrun & Kirthar	saxicolor	Possibly Extinct	29,000	4,700	16%	15.6	n	Pakistan
285	w of Central Bruhui mtns	saxicolor	Possibly Extinct	4,300	-	0%	8.3	n	Pakistan
286	Central Bruhui mtns; Hazar Ganji-Chiltan	saxicolor	Possibly Extinct	8,900	-	0%	19.8	n	Pakistan
287	east of Ziarat Juniper	saxicolor	Possibly Extinct	4,200	-	0%	7.2	y	Afghanistan, Pakistan
288	Afghan and Pakistan border	saxicolor	Possibly Extinct	28,000	-	0%	10.9	n	Pakistan
289	central Sulaiman mountains	saxicolor	Possibly Extinct	3,100	-	0%	27.5	n	Pakistan
290	NW coastal Africa	pardus	Extinct	494,400	-		101.2		
291	Maputo bay	pardus	Extinct	300	-		8.1		
292	Sahel, Red Sea coast, horn of Africa	pardus	Extinct	5,895,400	-		63.9		
293	coastal Guinea Bissau	pardus	Extinct	11,300	-		69.7		
294	Air Mountains	pardus	Extinct	30,600	-		0.9		
295	Qattara Depression	pardus	Extinct	13,200	-		0.0		
296	scattered Congo Basin tropical forest	pardus	Extinct	1,225,600	-		63.0		
297	Luanda, Angola	pardus	Extinct	2,100	-		2561.6		

298	central Angola	pardus	Extinct	73,800	-		47.4		
299	Yakoma DRC	pardus	Extinct	4,700	-		49.7		
300	western Uganda	pardus	Extinct	14,900	-		149.4		
301	eastern Lake Victoria	pardus	Extinct	154,900	-		161.3		
302	western Lake Turkana	pardus	Extinct	25,200	-		14.1		
303	western Chalbi desert	pardus	Extinct	13,500	-		1.2		
304	central Kenya	pardus	Extinct	18,000	-		79.1		
305	coastal Kenya, Tana delta	pardus	Extinct	3,900	-		75.8		
306	northern Tanzania, Usambara Mtns	pardus	Extinct	18,400	-		84.6		
307	Unguja, Zanzibar	pardus	Extinct	1,500	-		556.8		
308	Dar es Salaam, Tanzania	pardus	Extinct	4,800	-		892.1		
309	W of Rungwa GR, Tanzania	pardus	Extinct	5,300	-		13.0		
310	Tanzania, Zambia, Malawi border	pardus	Extinct	24,800	-		47.2		
311	E of Ruaha NP, Tanzania	pardus	Extinct	8,600	-		55.4		
312	SE coastal Tanzania	pardus	Extinct	11,200	-		69.1		
313	Malawi	pardus	Extinct	106,400	-		131.8		
314	NE coastal Mozambique	pardus	Extinct	61,400	-		60.3		
315	central coastal Mozambique	pardus	Extinct	12,700	-		70.6		
316	SE coastal Mozambique	pardus	Extinct	69,600	-		67.6		
317	Harare & central Zimbabwe	pardus	Extinct	56,700	-		36.1		
318	copper mining region Zambia	pardus	Extinct	7,100	-		112.1		
319	Lusaka, Zambia	pardus	Extinct	34,300	-		84.1		
320	Francistown, Botswana/Zimbabwe	pardus	Extinct	13,500	-		18.1		
321	central South Africa & eastern Namibia	pardus	Extinct	1,063,600	-		24.5		
322	E of Grootfontein	pardus	Extinct	2,000	-		1.0		
323	N of Grootfontein	pardus	Extinct	4,200	-		5.0		
324	S of Otjozondjupa	pardus	Extinct	2,100	-		0.1		
325	eastern Kunene region	pardus	Extinct	3,100	-		3.7		
326	Etosha Pan and north	pardus	Extinct	39,200	-		19.6		
327	coastal Red Sea	nimr	Extinct	832,400	-		53.3		
328	Hajar Mtns & Musandam Peninsula	nimr	Extinct	56,000	-		42.8		
329	SW Asia	saxicolor	Extinct	2,634,100	-		62.7		

330	coastal Caspian Sea, Iran	saxicolor	Extinct	1,900	-		256.0		
331	N Iran and Turkmenistan	saxicolor	Extinct	24,100	-		22.2		
332	NE Iran and transboundary	saxicolor	Extinct	3,100	-		4.9		
333	Gilgit-Baltistan, Pakistan	fusca	Extinct	17,600	-		32.5		
334	India, E Pakistan, Bangladesh	fusca	Extinct	2,379,100	-		420.3		
335	SW coastal India, Kerala	fusca	Extinct	23,200	-		1025.5		
336	Western Mynamar	fusca	Extinct	290,700	-		47.0		
337	Sri Lanka	kotiya	Extinct	40,900	-		372.7		
338	central & northern China	japonensis	Extinct	3,182,500	-		237.0		
339	Korean Peninsula, China & Russia	orientalis	Extinct	420,700	-		147.4		
340	SE Asia	delacouri	Extinct	2,348,500	-		130.2		
341	Singapore	delacouri	Extinct	500	-		8810.8		
342	Java, Indonesia	melas	Extinct	104,200	-		1078.3		
343	Kangean islands, Indonesia	melas	Extinct	400	-		274.0		

Bibliography

Abdoli A., Ghadirian T., Khaleghi Hamidi A., Mostafavi H., Moshiri H., Pour'salem S. & Ghoddousi A. 2008. First evidence of Persian leopard from Khaeez area, southern Iran. *CAT News* 48, 17.

Abow, A. 2013. Somali Environmental & Conservation Group.

Abramov V.K., & Pikunov, D.G. 1974. The snow leopard in the Soviet Far East and its conservation. *Bull Mosc Soc Nat Amateur Sec Biol* ,79, pp.5-15.

African Parks, Our Parks. n.d. *African Parks*. Available at: www.african-parks.org/Our+Parks [Accessed March 1, 2014].

Aghili, A. 2005. Leopard survey in Caucasus ecoregion (Northwest) of Iran. Report: 1-31. Leopard Conservation Society, Centre for Sustainable Development (CENESTA), Iran Department of Environment, Natural Environment & Biodiversity Office.

Aghor, A. 2008. Leopards more endangered than tigers: Activists. www.dnaindia.com [Accessed February 2008.]

Agostinho, A. J. 2012. The sustainability of leopard Panthera pardus sport hunting in Niassa National Reserve, Mozambique. MS Dissertation. School of Life Sciences, University of KwaZulu-Natal.

Ahmed, N. 2007. Saving the common leopards in Pakistan. www.wildlifeofpakistan.com www.wildlifeofpakistan.com/WildlifeCampaigns/savingthecommonleopardsinpakistan.htm [Accessed in April 2010.]

Albayrak, T., Giannatos, G., & Kabasakal, B. 2012. Carnivore and ungulate populations in the Beydaglari mountains (Antalya ,Turkey): border region between Asia and Europe. *Polish Journal of Ecology*, 60(2), pp.419–428.

ALERT. n.d. Funding proposal - Large Carnivore Conservation in Kusungu NP, Malawi.

Al Hikmani, H., Zaabanoot, N., & Zaabanoot, A. 2015. Camera trapping of Arabian leopard in the Nejd region of Dhofar Mountains. *CAT News* 62.

Al-Johany, A.M.H., 2007. Distribution and conservation of the Arabian Leopard *Panthera pardus nimr* in Saudi Arabia. *Journal of Arid Environments*, 68(1), pp.20–30.

Al Jumaily, M., Mallon, D.P., Nasher, A.K. & Thowabeh, N. 2006. Status Report on Arabian Leopard in Yemen. *CAT News*, SI 1, pp.20-25.

Amir, O. 2006. Wildlife trade in Somalia. Report to the IUCN / SSC/ Antelope Specialist Group - Northeast African Subgroup.

Amr, Z.S. & Disi, A.M. 1988. Jordanian Mammals Acquired by the Jordan University Natural History Museum. Publications of the University of Jordan, Amman, pp.1-32.

Angelici, F.M., Akani, G.C., & Luiselli, L. 1998. The leopard (*Panthera pardus*) in southeastern Nigeria: status, ecological correlates of occurrence and conservation implications. *Italian Journal of Zoology* 65: 307-310.

Anonymous. 1983. *The Red Book of the USSR*. Rare and endangered species of animals and plants. Vol 1. Vertebrates. Tashkent: 'Fan'. 128 p. (in Russian)

Anonymous. 2004. Leopard: Export quota for Namibia. 13th meeting of the Conference of the Parties (CoP) to the Convention on International Trade in Endangered Species of Wild Fauna And Flora (CITES), CoP13 Doc. 19.1, 02-14 October 2004, Bangkok, Thailand.

Anonymous. 2007a. Western Area Peninsula Forest (WAPF). National Commission on the Environment and Forestry (NaCEF), Sierra Leone Government.

Anonymous. 2007b. Transfer of the population of Leopard *Panthera pardus* in Uganda from Appendix I to Appendix II. Proposals for the 14th Conference of the Parties in The Hague (Netherlands) 13 - 15 June, 2007.

Anonymous. 2009. *The Red Book of the Republic of Uzbekistan*. Vol. 2. Tashkent. (in Russian)

Anonymous. 2013. *Strategy and action plan for the conservation of Javan leopard (Panthera pardus melas*) 2013-2023.

Anonymous, 2014. Leopard (*Panthera pardus)* distribution in Eritrea.

Ansell, W.F.H. 1978. *The Mammals of Zambia*. National Parks and Wildlife Service, Chilanga. 126pp.

Ansell, W.F.H., & Dowsett, R.J. 1988. *The Mammals of Malawi.* Trendrine Press, Zennor, Cornwall. 170pp.

APN. 2012. African Parks - Annual Report 2012, Johannesburg, South Africa.

Appel, A., Ghimirey, Y. & Acharya, R. 2012. Status assessment of wild felids with a special focus on clouded leopard and Asian golden cat in the Hugu-Kori forest, Annapurna Conservation Area, Nepal, Kathmandu, Nepal.

Appiah-Opoku, S. 2011. Using Protected Areas as a Tool for Biodiversity Conservation and Ecotourism: A Case Study of Kakum National Park in Ghana. *Society & Natural Resources*, 24(5), pp.500–510.

Arabuli, A. 2006. On distribution of leopard (*Panthera pardus ciscaucasica*) in the southern Caucasus (Georgia). Zoologicheskii Zhurnal, 85.

Ario, A., 2007. Javan Leopard (*Panthera pardus melas*) Among Human Activities, Indonesia.

Ario, A. & Hidayat, E. 2009. Protection and Monitoring of the Endangered Species of Javan Leopard (*Panthera pardus melas*) in Gunung Gede Pangrango National Park.

Arnett, G.R. 1982. Threatened status for the Leopard in Southern Africa. Endangered and threatened wildlife and plants - Federal Register / Rules and Regulations, Part II: Department of the Interior, Fish and Wildlife Service.

Aryal, R. 2003. Common leopard in crisis. Kathmandu Post. Available at www.nepalnews.com.np/contents/englishdaily/ktmpost/2003/jan/jan08/features.htm.

Aryal, A. & Kreigenhofer, B. 2009. Summer diet composition of the Common Leopard *Panthera pardus* (Carnivora : Felidae) in Nepal. *Journal of Threatened Taxa*, 1(11), pp.562–566.

Askerov, E., Talibov, T., Manvelyan, K., Zazanashvili, N., Malkhasyan, A., Fatullayev, P. & Heidelberg, A. 2015. South-Eastern Lesser Caucasus: the most important landscape for conserving the Leopard (*Panthera pardus*) in the Caucasus region (Mammalia: Felidae). *Zoology in the Middle East*, (ahead-of-print), pp.1-7.

Atamuradov, H.I., Fet, G.N., Fet, V., Valdez, R., & Feldman, W.R. 1999. Biodiversity, Genetic Diversity, and Protected Areas in Turkmenistan. *Journal of Sustainable Forestry* 9(1) 73-88. DOI:10.1300/JO91v09n01_06.

Athreya, V.R., Thakur, S.S., Chaudhuri, S., & Belsare, A.V. 2004. A Study of the Man-Leopard Conflict in the Junnar Forest Division, Pune District, Maharashtra.

Athreya, V., Odden, M., Linnell, J.D. & Karanth, K.U. 2011. Translocation as a Tool for Mitigating Conflict with Leopards in Human- Dominated Landscapes of India. *Conservation Biology*, *25*(1), pp.133-141. DOI: 10.1111/j.1523-1739.2010.01599.x.

Athreya, V.R., Thakur, S.S., Chaudhuri, S. & Belsare, A.V. 2013. Big cats in our backyards: persistence of large carnivores in a human dominated landscape in India. *PloS one*, 8(3), p.e57872.

Athreya, V., Srivathsa, A., Puri, M., Karanth, K. K., Kumar, N. S., & Karanth, K. U. 2015. Spotted in the News: Using Media Reports to Examine Leopard Distribution, Depredation, and Management Practices outside Protected Areas in Southern India. *PloS one*, 10(11), e0142647.

Avgan, B., Huseynali, T.T., Ismaylov, A., Fatallayev, P., Askerov, E. & Breitenmoser, U. 2012. First hard evidence of leopard in Nakhchivan. *CAT News*, (57), pp.33.

Babrgir, S., Farhadinia, M. S., & Moqanaki, E. M. 2015. Socio-economic consequences of cattle predation by the Endangered Persian leopard *Panthera pardus saxicolor* in a Caucasian conflict hotspot, northern Iran. *Oryx*, Available at: http://dx.doi.org/10.1017/S0030605315000903.

Bailey, T.N. 1993. *The African Leopard: Ecology and Behavior of a Solitary Felid*. Columbia University Press, New York, USA.

Bailey, T.N. 2005. *The African Leopard: Ecology and Bahavior of a Solitary Felid* (2nd edn). The Blackburn Press, New Jersey, USA.

Balme, G. A., Slotow, R. & Hunter, L.T.B., 2010. Edge effects and the impact of non-protected areas in carnivore conservation: leopards in the Phinda-Mkhuze Complex, South Africa. *Animal Conservation*, 13(3), pp.315–323. doi:j.1469-1795.2009.00342.x.

Baskaya, S. & Bilgili, E. 2004. Does the leopard *Panthera pardus* still exist in the Eastern Karadeniz Mountains of Turkey? *Oryx*, 38(02), pp.228–232.

Bauer, H. & Kari, S. 2001. Assessment of the people-predator conflict through thematic PRA in the surroundings of Waza National Park, Cameroon. *PLA Notes*, 41, pp.9–13.

Bertram, B.C.R. 1982. Leopard ecology as studied by radio tracking. In Symp. Zool. Soc. Lond, 49, pp. 341-352.

Boast, L.K., & Houser, A. 2012. Density of Large Predators on Commercial Farmland in Ghanzi , Botswana. *South African Journal of Wildlife Research*, 42(2), pp.138–143.

Bodendorfer, T., Hoppe-Dominik, B., Fischer, F., & Linsenmair, K. E. 2006. Prey of leopard (*Panthera pardus*) and the lion (*Panthera leo*) in the Comoé and Marahoué National Parks, Côte d'Ivoire, West Africa. *Mammalia,* 70, 231-246.

Bogdanov, O.P. 1952. To the distribution of a leopard in Uzbekistan. Proceedings of Academy of Sciences of Uzbekistan. Tashkent.

Borah, J., Sharma, T., Das, D., Rabha, N., Kakati, N., Basumatary, A., Ahmed, M.F. & Vattakaven, J. 2014. Abundance and density estimates for common leopard *Panthera pardus* and clouded leopard *Neofelis nebulosa* in Manas National Park, Assam, India. *Oryx*, 48(01), pp.149–155.

Borries, C. & Koenig, A., 2014. Opportunistic sampling of felid sightings can yield estimates of relative abundance. *CAT News*, 61, pp.34–37.

Bothma, J.D.P. & Bothma, M.D. 2012. Leopard range size and conservation area size in the southern Kalahari. *Koedoe - African Protected Area Conservation and Science*, 54(1), pp.1–4.

Bouché, P., Mange, R.N.M., Tankalet, F., Zowoya, F., Lejeune, P. & Vermeulen, C. 2012. Game over! Wildlife collapse in northern Central African Republic. *Environmental Monitoring and Assessment*, 184(11), pp.7001–7011.

Boug, A., Zafar-ul Islam, M., & Shehri, A. 2009. Captive-breeding of Arabian leopard *Panthera pardus nimr* in the Kingdom of Saudi Arabia. Wildlife Middle East News, Vol. 4, Issue 3, December 2009.

Boulet, H., Chardonnet, P., Crosmary, W., Ferro, P., Fritz, H., Pellerin, M., & Plasschaert, C. 2008. Carnets de brousse - suivi écologique d'espèces peu communes en Afrique Centrale et en Afrique de l'ouest. [in French]

Breitenmoser-Würsten, C., Breitenmoser, U., Mallon, D., & Zazanashvili, N. 2007. Strategy for the Conservation of the Leopard in the Caucasus Ecoregion, Tbilisi, Georgia.

Breitenmoser, U., Breitenmoser, C., Mallon, D. & Edmonds, J. 2010. Strategy for the Conservation of the Leopard in the Arabian Peninsula, Sharjah, UAE.

Breitenmoser, U., Avgan, B., Zimmerman, F., & Breitenmoser, C. 2011. Monitoring for the Conservation of the Leopard in the Caucasus Ecoregion, Tbilisi, Georgia.

Breitenmoser, U., Breitenmoser-Wursten, C., Zazanashvili, N., & Heidelberg, A. 2014. International Expert Workshop Conservation of the Leopard in the Causcasus: 9-10 October 2014. Tblisi Georgia. IUCN/SSC/WWF.

Brugière, D., Badjinca, I., Silva, C., Serra, A. & Barry, M., 2005. Distribution and Status of Lions and Leopards in Southern Guinea Bissau and Western Guinea, West Africa. *CAT News*, 43, pp.13–17.

Burton C., Buedi, E.B., Balangtaa, C., Kpelle, D.G., Sam, M.K. & Brashares, J.S. 2011. The decline of lions in Ghana's Mole National Park. *African Journal of Ecology*, 49(1), pp.122-126.

Busby, G.B., Gottelli, D., Wacher, T., Marker, L., Belbachir, F., De Smet, K., Belbachir-Bazi, A., Fellous, A., Belghoul, M. and Durant, S.M., 2009. Genetic analysis of scat reveals leopard *Panthera pardus* and cheetah *Acinonyx jubatus* in southern Algeria. *Oryx*, 43(03), pp.412.

Butynski, T. M. 1984. Ecological survey of the Impenetrable (Bwindi) Forest, Uganda, and recommendations for its conservation and management. Unpublished report for the Government of Uganda and Wildlife Conservation International, Kampala and New York. 150 pp.

CARACAL, 2006. ODMP Wildlife Management Component Leopard and cheetah baseline inventory in the Okavango Delta particularly in relation to areas of Human Wildlife Conflict, Kasane, Botswana.

Caso, M. S. A. 2002. Leopard pilot population study at Rungwa/Piti ecosystem, Tanzania, East Africa. Final Report.

CBSG, 2005. Leopard population and habitat viability assessment (PHVA).

Chakma, S. 2015. Assessment of large mammals of the Chittagong Hill Tracts of Bangladesh with emphasis on tiger (*Panthera tigris*). Unpublished Ph.D. Thesis. University of Dhaka, Bangladesh. 206 pp.

Chase-Grey, J.N. 2011. Leopard population dynamics, trophy hunting and conservation in the Soutpansberg Mountains, South Africa. Durham University.

Chatta, S.A., Hussain, S.M., Javid, A., Abbas, M.N., Mahmood, S., Barq, M.G, & Hussain, M. 2015. Seasonal diet composition of leopard (*Panthera pardus*) in Machiara National Park, Azad Jammu and Kashmir, Pakistan. *Pakistan Journal of Zoology,* 47(1), pp.201-207.

Chauhan, D.S., Harihar, A., Goyal, S.P., Qureshi, Q., Lal, P. & Mathur, V.B. 2005. Estimating Leopard Population using Camera Traps in Sariska Tiger Reserve, Dehra Dun, India.

Chemonics International Inc. 2001. *Biodiversity Assessment for Turkmenistan.* USAID Central Asian Republics Mission, Almaty, Kazakhstan. Washington, DC.

CHIMBO Foundation. 2014. Annual Report 2014. Chimbo Foundation, Amsterdam, The Netherlands.

CITES, 2007. Leopard Export Quotas for Mozambique. In Fourteenth meeting of the Conference of the Parties. The Hague, Netherlands: CITES, pp. 1–10.

CLWG. 2014. Conservation of the leopard in the Caucasus, Workshop report. 9-10 October 2014. Tblisi, Georgia.

Coudrat, C.N.Z., Nanthavong, C., Sayavong, S., Johnson, A., Jonhston, J.B. & Robichaud, W.G. 2014. Non-Panthera cats in Nakai-Nam Theun National Protected Area, Lao PDR. *CAT News*, SI 8, pp.45–52.

Croes, B.M., Funston, P.J., Rasmussen, G., Buij, R., Saleh, A., Tumenta, P.N. & De Iongh, H.H. 2011. The impact of trophy hunting on lions (*Panthera leo*) and other large carnivores in the Bénoué Complex, northern Cameroon. *Biological Conservation*, 144(12), pp.3064–3072.

Cuzin, F. 2003. *Les grands mammiferes du maroc meridional* (Haut Atlas , Anti Atlas et Sahara): Distribution , écologie et conservation. Université Montpellier.

D'Amour, D.E., Hohmann, G. & Fruth, B., 2006. Evidence of leopard predation on bonobos (*Pan paniscus*). *Folia primatologica; international journal of primatology*, 77(3), pp.212–7.

Dar, N. I., Minhas, R.A., Zaman, Q., & Linkie, M. 2009. Predicting the patterns, perceptions and causes of human-wildlife conflict in and around Machiara National Park, Pakistan. *Biological Conservation,* 142, pp.2076-2082. DOI: 10.1016/j.biocon.2009.04.003.

De Smet, K. 1982. La disparition des grands fauves en Algérie. *Bull Forest Conserv Nat Alger*, 1, pp.23–25.

De Smet, K.J.M. 1989. Distribution and habitat choice of larger mammals in Algeria, with special reference to nature protection. Ph.D. Thesis, Ghent State University. 355 pp. (In Dutch)

di Silvestre, I., Novelli, O. & Bogliani, G. 2000. Feeding habits of the spotted hyaena in the Niokolo Koba National Park, Senegal. *African Journal of Ecology*, 38, pp.102–107.

Degeorges, A. & Reilly, B. 2007. Politicization of land reform in Zimbabwe: impacts on wildlife, food production and the economy. *International Journal of Environmental Studies*, 64(5), pp.571–586.

Demeke, Y. & Aklilu, N., 2008. Alarm bell for biofuel development in Ethiopia: the case of Babille Elephant Sanctuary. In T. Heckett & N. Aklilu, eds. *Agrofuel Development in Ethiopia: Rhetoric, Reality and Recommendations*. Addis Ababa, Ethiopia: Forum for Environment, pp. 83–113.

Dhanwatey, H.S., Crawford, J.C., Abade, L.A., Dhanwatey, P.H., Nielsen, C.K. and Sillero-Zubiri, C., 2013. Large carnivore attacks on humans in central India: a case study from the Tadoba-Andhari Tiger Reserve. *Oryx*, *47*(02), pp.221-227. DOI: 10.1017/S0030605311001803.

Direktorat PPA. 1978. Mamalia di Indonesia. Direktorat PPA, Direktorat Jenderal Kehutanan. Bogor.

Direktorat PPA. 1982. Pedoman Teknik Inventarisasi Mamalia (Dasar-dasar Umum). Direktorat PPA, Direktorat Jenderal Kehutanan. Bogor.

Drake-Brockman, R.E. 1910. *The Mammals of Somaliland*. London, UK. Hurst & Blackett.

Drolet, C. 1990. Biological survey of Nigeria. A programm development submitted to the IUCN. Canadian Wildlife Service, Quebec City.

Drucker, G. 1991. The Barbary leopard *Panthera pardus barbarus*. World Conservation Monitoring Centre.

Durant, S. Erickson, D., Ikanda, D., Kibebe, J., Kazaeli, C., Konzo, E., Lejora, I., Lichtenfeld, L., Lobora, A., Magoma, N., Minushi, L., Mpuya, P., Packer, C., Rwina, N., Rwiza, M., Sabuni, G., & Trout, C. 2006. Tanzania Lion and Leopard Conservation Action Plan Workshop. In Proceedings from the first Tanzania Lion and Leopard Conservtion Action Plan workshop. Arusha, Tanzania: Darwin Initiative, ZSL, TAWIRI, WCS.

Dutta, T., Sharma, S., Maldonado, J.E., Wood, T.C., Panwar, H.S. & Seidensticker, J. 2013. Gene flow and demographic history of leopards (*Panthera pardus*) in the central Indian highlands. *Evolutionary applications*, *6*(6), pp.949-959. DOI:10.1111/eva.12078.

Eames, J.C., Nguyen Duc Tu, Le Trong Trai, Dang Ngoc Can, Ngo Van Tri, Hoang Duc Dat, Thail Ngoc Tri, & Nguyen Thi Thu He, 2004. Final biodiversity report for Yok Don National Park, Dak Lak Province, Vietnam. PARC Project VIE/95/G31&0313, Government of Vietnam (FPD)/UNOPS/UNDP/Scott Wilson Asia-Pacific Ltd./Environment and Development Group and Forest Renewable Resources Ltd., Hanoi.

East, R. 2006. Wildlife populations of northern Central African Republic. Gnusletter, 25 (1).

Eaton, R.L., 1977. *The status and conservation of the leopard in Sub-Saharan Africa*, Tucson, Arizona.

Edgaonkar, A., 2008. *Ecology of the leopard (Panthera pardus) in Bori Wildlife Sanctuary and Satpura National Park, India*. Ph.D Thesis. University of Florida.

Edmonds, J.A., Budd, K.J., al Midfa, A. & Gross, C. 2006. Status of the Arabian Leopard in United Arab Emirates. *CAT News,* SI 1, pp.33-39.

Estes. R. 1991. *The behavior guide to African mammals*. The University of California Press.

Everatt, K.T., Andresen, L. & Somers, M.J. 2014. Trophic scaling and occupancy analysis reveals a lion population limited by top-down anthropogenic pressure in the Limpopo National Park, Mozambique. PLoS ONE, 9(6), e99389. DOI:10.1371/journal.pone.0099389.

Farhadinia, M., Nezami, B., Mahdavi, A. & Hatami, K., 2007. Photos of Persian leopard in Alborz Mountains, Iran. *CAT News*, 46, pp.34–35.

Farhadinia, M.S., Akbari, H., Beheshti, M. & Sadeghi, A. 2008. Felids of Abbasabad Naein Reserve, Iran. *CAT News*, 48, pp.14-16.

Farhadinia, M.S., Nezami, B., Hosseini-Zavarei, F., & Valizadeh, M. 2009. Persistence of Persian leopard in a buffer habitat in north- eastern Iran. *CAT News*, 51, pp.34–36.

Farhadinia, M. S., Farahmand, H., Gavashelishvili, A., Kaboli, M., Karami, M., Khalili, B. and Montazamy, S. 2015. Molecular and craniological analysis of leopard, *Panthera pardus* (Carnivora: Felidae) in Iran: support for a monophyletic clade in Western Asia. *Biological Journal of the Linnean Society*, 114: 721–736. doi: 10.1111/bij.12473.

Farhadinia, M.S., Memarian, I., Shahrdari, A., Taghdisi, M., Jafari, B., Molazem, M., Moghani, F., & Macdonald, D.W. 2015b. Capturing an old problem Persian leopard close to the Iran-Turkmenistan border. *CAT News,* 62, pp.29-31.

Foley, C., Foley, L., Lobora, A., De Luca, D., Msuha, M., Davenport, T.R. & Durant, S.M. 2014. *A Field Guide to the Larger Mammals of Tanzania*. Princeton University Press.

Friedmann Y. and Traylor-Holzer K. 2008. Leopard (*Panthera pardus*) case study Endangered Wildlife Trust, IUCN/SSC Conservation Breeding Specialist Group, 29p.

Funston, P., Hanssen, L. & Moeller, M. 2014. Large Carnivore Survey - Bwabwata National Park, Namibia, July 2014.

Fusari, A., Cumbi, R., Chardonnet, P., & Begg, C. 2010. Conservation Strategy and Action Plan for the African Lion in Mozambique, Maputo, Mozambique.

Gavashelishvili, A. & Lukarevskiy, V. 2008. Modelling the habitat requirements of leopard, *Panthera pardus*, in west and central Asia. *Journal of Applied Ecology*, 45(2), pp.579–588.

Gebresenbet, F., Bauer, H., Hunter, L., Gebretensae, K. 2009. Proceedings of the National Lion Conservation Workshop. Addis Ababa, 12 June 2009. pp.1-42.

Ghadirian, T. & Ghasemi, M. 2012. New records of Persian leopard in Bashagard area, southeastern Iran. *CAT News*, 56, pp.32-33.

Ghimirey, Y., & Acharya, R. 2012. Records of Siberian Weasel *Mustela sibirica* and Yellow-bellied Weasel *M. kathiah* from Makalu–Barun National Park, Nepal. *Small Carnivore Conservation*, 47, pp.65-66.

Ghoddousi, A., Kh Hamidi, A., Ghadirian, T., Ashayeri, D. & Khorozyan, I. 2010. The status of the Endangered Persian leopard *Panthera pardus saxicolor* in Bamu National Park, Iran. *Oryx*, 44(04), pp.551–557.

Ghoddousi, A., Soofi, M., Khorozyan, I., Kiabi, B.H., & Waltert, M. 2015. Effects of ranger stations on predator and prey distribution and abundance in an Iranian steppe landscape. *Animal Conservation*. DOI: 10.1111/acv.12240

Ghosal, S., & Kjosavik, D.J. 2015. Living with leopards: negotiating morality and modernity in western India. *Society and Natural Resources* 0:1-16. DOI:10.1080/08941920.2015.1014597.

Ghosal, S., Athreya, V.A., Linnell, J.D.C., & Vedeld, P.O. 2013. An ontological crisis? A review of large felid conservation in India. *Biodiversity and Conservation,* 22, pp.2665-2681. DOI:10.1007/s10531-013-0549-6.

Goldman, H. V & Walsh, M.T. 2002. Is the Zanzibar Leopard (*Panthera pardus adersi*) extinct? *Journal of East African Natural History*, 91, pp.15–25.

Gonfa, R., Gadisa, T. & Habitamu, T. 2015. The diversity, abundance and habitat association of medium and large-sized mammals of Dati Wolel National Park, Western Ethiopia. *International Journal of Biodiversity and Conservation*, 7(2), pp.112–118.

Goswami, R. & Ganesh, T. 2014. Carnivore and herbivore densities in the immediate aftermath of ethno-political conflict: the case of Manas National Park, India. *Tropical Conservation Science*, 7(3), pp.475-487.

Goyal, S.P. 2000. A study on distribution, relative abundance and food habits of leoaprd (*Panthera pardus*) in Garhwal Himalayas. Wildlife Institute of India, Dehradun.

Grant, T. 2012. Leopard population density, home range size and movement patterns in a mixed landuse area of the mangwe district of Zimbabwe. M.Sc. Thesis. Rhodes University.

Gray, T.N.E., Calame, T., Hayes, B., Hurley, M.J., Nielsen, P.H., Lamxay, V., Timmins, R.J., Thongsamouth, K. 2013. Biodiversity Surveys of Xe Sap National Protected Area Lao PDR 2012. WWF Greater Mekong Vientiane, Lao PDR.

Gray, T.N.E. & Prum, S. 2012. Leopard density in post-conflict landscape, Cambodia: Evidence from spatially explicit capture-recapture. *The Journal of Wildlife Management*, 76(1), pp.163–169..

Gray, T.N.E., Ou, R., Huy, K., Pin, C. & Maxwell, A.L., 2012. The status of large mammals in eastern Cambodia: a review of camera trapping data 1999-2007. *Cambodian Journal of Natural History*, 1, pp.42–45.

Green, M. 1987. The conservation status of the leopard, goral and serow in Bangladesh, Bhutan, northern India and southern Tibet. IUCN Conservation Monitoring Centre for the United States Fish and Wildlife Service.

Groom, R. & Brand, H. 2011. Carnivore Densities in Gonarezhou National Park Results of the June 2011 Spoor Survey. Unpublished report. Zimbabwe Parks and Wildlife ManagementAuthority. Harare, Zimbabwe.

Grubb, P. 1998. *Mammals of Ghana, Sierra Leone and the Gambia*, The Trendrine Press. 330 p.

Gubbi, S., Ghat, R., Nagashettihalli, H. & Madhusudan, M.D. 2014a. Ecology and conservation status of leopards in Bhadravathi Territorial Division. Nature Conservation Foundation, Mysore, India.

Gubbi, S., Poornesha, H.C., Daithota, A. & Nagashettihalli, H. 2014b. Roads emerging as a critical threat to leopards in India? CAT News, 60, pp.30-31.

Guggisberg, C. 1975. *Wild cats of the world*. David and Charles, London.

Gumal, M., Abu Bakar, M., Mohd Nawayai, Y., Horng, L. S., Lee, B. P. Y., Chee Pheng, L., Hasnizam, H., Kong, D., Magintan, D., Ten Choon Yong, D., Ahmad Zulfi, Z., Azima, A., Khalid, N., Thai, P. Y., Voon, M., Cheong Fook Meng, F., & Ng, S. 2014. Small-medium wild cats of Endau Rompin Landscape in Johor, Peninsular Malaysia. *CAT News,* SI 8, pp.10–18.

Gunawan, H. 2009. Ekologi Macan Tutul (*Panthera pardus melas* Cuvier 1809) dan Masalah konsevasinya. Paper disapalkan pada Seminar Penyusunan strategi dan Rencana Aksi Konservasi Macan tutui di hotel safari, Taman safari Indonesia. [in Indonesian]

Gunawan, H., L.B. Prasetyo, A. Mardiastuti & A.P. Kartono. 2010. Fragmentation of Dryland Natural Forest in Central Java Province*. Jurnal Penelitian Hutan dan Konservasi Alam VII* (1), pp.75-91.

Gunawan, H. Prasetyo, L.B., Mardiastuti, A., & Kartono, A.P. 2012. Distribution and Habitat Selection of Javan Leopard, *Panthera pardus melas* in Central Java Province. *Penelitian Hutan dan Konservasi Alam*, 9(4), pp.323–339.

Gunawan, H., L.B. Prasetyo, A. Mardiastuti & A.P. & Kartono. 2013. Analisis Metapopulasi dan Prediksi Kepunahan Lokal Macan Tutul Jawa (Panthera pardus melas Cuvier 1809) di Jawa Tengah. Pp. 360-376 in Prabowo, R.E. et al (eds). *Prosiding Seminar Nasional Biologi PBI XXII* 2013: "Peran Biologi dalam Pendayagunaan Bioresources Indonesia untuk Meningkatkan Daya Saing Bangsa. Purwokerto, 31 August – 1 September 2013. [in Indonesian]

Habibi, K. 2004. *Mammals of Afghanistan*. Report, Kabul.

Hafdaoui, A. 1984. La Panthere. Bull. Ass. Ing. Eaux Forests, 2, pp.47-52.

Hamidi A.K., Ghoddousi, A., Soufi, M., Ghadirian, T., Jowkar, H., & Ashayeri, S. 2014. Camera trap study of Persian leopard in Golestan National Park, Iran. *CAT News,* 60, pp.12–14.

Hamilton, P.J. 1981. The leopard *Panthera pardus* and the cheetah *Acinonyx jubatus* in Kenya. Ecology Status Conservation Management. Report for the U.S. Fish & Wildlife Service.

Hamilton, P.J. 1987. *Status of the leopard in Kenya, with reference to Sub-Saharan Africa*.

Han, X. 2001. Far Eastern leopard and Siberian tiger conservation measures. Jilin Provincial Academy of Forestry Sciences.

Hanssen, L., & Stander, P. 2004. *Namibia Large Carnivore Atlas*, Windhoek, Namibia.

Happold, D.C.D. 1987. *The Mammals of Nigeria*. Clarendon Press, Oxford, UK.

Harihar, A., Pandav, B. & Goyal, S.P., 2009. Density of leopards (*Panthera pardus*) in the Chilla Range of Rajaji National Park, Uttarakhand, India. *Mammalia*, 73(1), pp.68–71.

Harrison, D.L. 1968. *The Mammals of Arabia: In 3 Vols!* Carnivora, Artiodactyla, Hyracoidea. Benn.

Harrison, D. L., & P. J. J. Bates. 1991. Felidae. In: *The Mammals of Arabia* (2nd Edition). Harrison Zoological Museum, Sevenoaks, UK. pp.156-172.

Hatt, R.T. 1959. Cheetah. In: Hatt R.T., editor. *The mammals of Iraq*. Michigan: Ann Arbor. University of Michigan; pp.46-52.

Hayward, M.W., Hayward, G.J., Druce, D.J. & Kerley, G.I. 2009. Do fences constrain predator movements on an evolutionary scale? Home range, food intake and movement patterns of large predators reintroduced to Addo Elephant National Park, South Africa. *Biodiversity and Conservation*, 18(4), pp.887–904..

Hebblewhite, M., Miquelle, D.G., Murzin, A.A., Aramilev, V.V. & Pikunov, D.G. 2011. Predicting potential habitat and population size for reintroduction of the Far Eastern leopards in the Russian Far East. *Biological Conservation*, 144(10), pp.2403–2413.

Hedges, L., Yee Lam, W., Campos-Arceiz, A., Rayan, D.M., Laurance, W.F., Latham, C.J., Saaban, S., & Clements, G.R. 2015. Melanistic leopards reveal their spots: Infrared camera traps provide a population density estimate of leopards in Malaysia. *Journal of Wildlife Management*, 79, pp.846-853.

Heim de Balsac, H. 1923. Contribution a I'omithologie dans le Sahara septentrional en Algerie et en Tunisie avec notes sur la flore et la faune des regions parcoumes. Rev. Fran?. *Omithol.*, 8: 1-112. [in French]

Henschel, P., & Ray, J.C. 2003. *Leopards in African Rainforests: Survey and Monitoring Techniques*. Wildlife Conservation Society, Global Carnivore Program, New York, USA.

Henschel, P. 2008. *The conservation biology of the leopard Panthera pardus in Gabon: Status, threats and strategies for conservation*. Ph.D Dissertation. Georg-August-Universität zu Göttingen.

Henschel, P., Hunter, L., Breitenmoser, U., Purchase, N., Packer, C., Khorozyan, I., Bauer, H., Marker, L., Sogbohossou, E. & Breitenmoser-Wursten, C. 2008. Panthera pardus. *IUCN Red List of Threatened Species*. Available at: www.iucnredlist.org [Accessed March 1, 2014].

Henschel, P. 2009. The Status and Conservation of Leopards and Other Large Carnivores in the Congo Basin, and the Potential Role of Reintroduction. In M. W. Hayward & M. J. Somers, eds. *Reintroduction of Top-Order Predators*. Blackwell Publishing Ltd., pp. 206–237.

Henschel, P., Azani, D., Burton, C., Malanda, G., Saidu, Y., Sam, O., & Hunter, L. 2010. Lion status updates from five range countries in West and Central Africa. *CAT News*, 52, pp.34–39.

Henschel, P., Hunter, L., Coad, L., Abernethy, K., & Mühlenberg, M. 2011. Leopard prey choice in the Congo Basin rainforest suggests exploitative competition with human bushmeat hunters, *Journal of Zoology*, pp.1-10.

Henschel, P., Kiki, M., Sewade, C., & Tehou, A. 2012. Improving the status of lions and cheetahs in their last stronghold in West Africa: The W-arly-pendjari complex. New York: Panthera.Henschel, P., Coad, L., Burton, C., Chataigner, B., Dunn, A., MacDonald, D., Saidu, Y. & Hunter, L.T.B. 2014. The lion in West Africa Is Critically Endangered. *PLoS ONE*, 9, e83500.

Henschel, P., Malanda, G.-A. & Hunter, L. 2014. The status of savanna carnivores in the Odzala-Kokoua National Park, northern Republic of Congo. *Journal of Mammalogy*, 95(4), pp.882–892.

Heptner, V.G., & A.A. Sludskii. 1972. Mammals of the Soviet Union - Carnivora, Hyenas and Cats. Moscow. Vysshaya Shkola. [in Russian]

Heptner, V.G., & A.A. Sludskii. 1992. *Mammals of the Soviet Union.* Vol. 2, part 2: Carnivores (Felidae) Leiden, E.J. Brill.

HMGN/MFSC. 2002. *Nepal biodiversity strategy.*

Hoath, R. 2003. *A field guide to the mammals of Egypt*. The American University in Cairo Press, Cairo, Egypt.

Hoke, P., Demey, R. & Peal, A. 2007. Rapid survey of large mammals of North Lorma, Gola, and Grebo National Forests, Liberia., Arlington, VA, USA.

Holst, B., Ahmed, A., Aouled, A. A., Mohamed, A. A., Laurent, A., Aman, A. M., Desbiez, A., Mulot, B., Lafrance, B., Gibault, C., Mallon, D., Ruivo, E., Rayaleh, H. A., Leus, K., Moehlmann, P., & McGowan, P. 2013. Conserving Djibouti's Priority Land Animals - A seminar and Conservation Workshop, Cerd, Djibouti.

Hoogerwerf, A. 1970. Udjung Kulon: the land of the last Javan rhinoceros. E.J. Brill, Leiden.

Hoppe- Dominik, B., Kühl, H.S., Radl, G. & Fischer, F. 2011. Long-term monitoring of large rainforest mammals in the Cote d'Ivoire Biosphere Reserve of Tai National Park , Cote d'Ivoire. *African Journal of Ecology*, 49, pp.450–458.

Horsten, F. 1982. Documentacao sobre: os parques nacionais es as outras zonas de proteccao da natureza de Angola.

Houpline, C. 2010. Lion d'Afrique de l'Ouest: statut de conservation en Guinée. CEPA magazine, No 22, Octobre 2010.

Hu, Y., Yao, Z., Huang, Z., Tian, Y., Li, H., Pu, Q. et al. 2014. Mammalian fauna and its vertical changes in Mt Qomoloangma National Nature Reserve, China. *Acta Theriologica Sinica*, 34, pp.28-37. [in Chinese]

Hufnagl, E. 1972. *Libyan mammals*. The Oleander Press.

Hunter, L., & Balme, G. 2004. The leopard: the world's most persecuted big cat. *Conservation in Action*, pp.88-94.

Hunter, L., & Balme, G. 2005. Trash or in trouble? - The leopard's predicament. *Africa Geographic*, pp.60-7.

Hunter, L., Jowkar, H., Ziaie, H., Schaller, G., Balme, G., Walzer, C., Ostrowski, S., Zahler, P., Robert-Charrue, N., Kashiri, K. & Christie, S. 2007. Conserving the Asiatic cheetah in Iran: launching the first radio-telemetry study. *CAT News*, 46, pp.8-11.

Hunter, L., Henschel, P. & Ray, J.C., 2013. *Panthera pardus*. In J. S. Kingdon & M. Hoffmann, eds. *The Mammals of Africa*. Amsterdam, the Netherlands: Academic Press.

Husain T. 2001. Survey for the Asiatic cheetah, *Acinonyx jubatus*, in Balochistan province, Pakistan Barbara Delano Foundation; 39 p.

Ikemeh, A.R. 2007. Okomu Leopard Study - A Preliminary Assessment of the Status and Distribution of Leopards in Okomu Forest Reserve, South, Benin City, Nigeria.

Inskip, C., & Zimmermann, A. 2009. Human-Felid Conflict: A Review of Patterns and Priorities Worldwide. *Oryx,* 43, pp.18-34. doi:10.1017/S003060530899030X.

Instituto Oikos. 2011. *Myanmar Protected Areas - Context, Current Status and Challenges*. Milano, Italy.

Ishunin, G.I. 1961. *Mammals (ungulates and predators). Fauna of Uzbek SSR*. Volume 3. Tashkent. [in Russian]

ITAR-Tass. 2013. Number of Amur leopards increases by half. ITAR-TASS News Agency. Available at: http://en.itar-tass.com/archive/690905 [Accessed June 1, 2014].

Jachmann, H. 2008. Monitoring law-enforcement performance in nine protected areas in Ghana. *Biological Conservation*, 141(1), pp.89–99.

Jackson, P. 1989. *The Status of the Leopard in sub-Saharan Africa: A Review by Leopard Specialists.* Unpublished report of the IUCN Cat Specialist Group, IUCN, Gland, Switzerland.

Jackson, P., & Nowell, K. 2008. Panthera pardus ssp. orientalis. The IUCN Red List of Threatened Species 2008: e.T15957A5333757. http://dx.doi.org/10.2305/IUCN.UK.2008.RLTS.T15957A5333757.en. Downloaded on 14 December 2015.

Jacobson A.P., Cattau M.E., Riggio J.S., Petracca L.S., & Fedak D.A. 2013. Distribution and abundance of lions in northwest Tete Province, Mozambique. *Tropical Conservation Science*, 6, pp.87-107.

Jacobson, A., Dhanota, J., Godfrey, J., Jacobson, H., Rossman, Z., Stanish, A., Walker, H., & Riggio, J. 2015. A novel approach to mapping land conversion using Google Earth with an application to East Africa. *Environmental Modelling & Software*, 72, pp.1-9.

Jenny, D. 1996. Spatial organization of leopards Panthera pardus in Tai National Park, Ivory Coast: Is rainforest habitat a "tropical haven?" *Journal of Zoology, London*, 240, pp.427–440.

Jhala, Y.V., Qureshi, Q., Gopal, R., & Sinha, P.R. (eds). 2011. *Status of the tigers, co-predators, and prey in India, 2010*. National Tiger Conservation Authority, New Delhi & the Wildlife Institute of India, Dehradun. TR 2011/003, pp302.

Jhala, Y.V., Qureshi, Q., & Gopal, R. (eds). 2015. *The status of tigers in India 2014.* National Tiger Conservation Authority, New Delhi & the Wildlife Institute of India, Dehradun.

Jnawali, S.R. 2011. *The Status of Nepal's Mammals: The National Red List Series*, Kathmandu, Nepal.

Johnson, A., Vongkhamheng, C., Hedemark, M. & Saithongdam, T. 2006. Effects of human-carnivore conflict on tiger (*Panthera tigris*) and prey populations in Lao PDR. *Animal Conservation*, 9(4), pp.421–430.

Johnson, K., 2003. Status and Distribution of the Leopard (*Panthera pardus*) in Turkey and the Caucasus Mountains. *Endangered Species UPDATE*, 20(3), pp.107–122.

Johnson, M.F. & Wingard, J.R. 2010. *Wild fauna trade in Afghanistan*. Wildlife Conservation Society. Kabul, Afghanistan. pp 1-75.

Jongbloed, M. 2001. Working for Wildlife. Barkers Trident Communications, London.

Jorge, A.A., Vanak, A.T., Thaker, M., Begg, C. & Slotow, R. 2013. Costs and benefits of the presence of leopards to the sport-hunting industry and local communities in Niassa National Reserve, Mozambique. *Conservation Biology*, 27(4), pp.832–43.

Joshi, R. & Agarwal, R. 2012. Mortality in the Protected Leopard's Population, Uttarakhand, North India: A Free-Ranging Wildlife Species in Threat. *International Journal of Ecosystem*, 2(4), pp.44–53.

Judas, J., Paillat, P., Khoja, A. & Boug, A. 2006. Status of the Arabian leopard in Saudi Arabia. *CAT News*, SI 1, pp.11-19.

Jutzeler, E., Wu, Z., Liu, W., & Breitenmoser, U. 2010. Leopard *Panthera pardus*. *CAT News,* SI5, pp.30–33.

Kabir, M., Ghoddousi, A., Awan, M.S. & Awan, M.N. 2013. Assessment of human–leopard conflict in Machiara National Park, Azad Jammu and Kashmir, Pakistan. *European Journal of Wildlife Research*.

Kaczensky, P. & Linnell, J.D.C., 2015. Rapid assessment of the mammalian community of the Badhyz Ecosystem, Turkmenistan, October 2014.

Kalle, R., Ramesh, T., Qureshi, Q. & Sankar, K. 2011. Density of tiger and leopard in a tropical deciduous forest of Mudumalai Tiger Reserve, southern India, as estimated using photographic capture–recapture sampling. *Acta Theriologica*, 56(4), pp.335–342.

Kane, M., Morin D., & Kelly, M. 2015. Counting cats at the northern extreme of their range: preliminary population assessment of lions, leopards, and servals in Niokolo-Koba National Park, Senegal. *Wild Felid Monitor*, 8(1), pp.12–17.

Karanth, K. K., Gopalaswamy, A. M., DeFries, R., & Ballal, N. 2012. Assessing patterns of human-wildlife conflicts and compensation around a central Indian protected area. *PlosOne* e50433.

Karanth, K. K., Gopalaswamy, A. M., Prasad, P. K., & Dasgupta, S. 2013. Patterns of human–wildlife conflicts and compensation: Insights from Western Ghats protected areas. *Biological Conservation,* 166, pp.175-185. DOI:10.1016/j.biocon.2013.06.027.

Katel, O.N., Pradhan, S., & Schmidt-Vogt, D. 2014. A survey of livestock losses caused by Asiatic wild dogs, leopards and tigers, and of the impact of predation on the livelihood of farmers in Bhutan. *Wildlife Research,* 41, pp.300-310. DOI: 10.1071/WR14013.

Kawanishi, K. & M. Sunquist. 2004. Conservation status of tigers in a primary rainforest of Peninsular Malaysia. *Biological Conservation*, 120 (3), pp.329-344.

Kawanishi, K., Sunquist, M.E., Eizirik, E., Lynam, A.J., Ngoprasert, D., Wan Shahruddin, W.N., Rayan, D.M., Sharma, D.S.K., & Steinmetz, R. 2010. Near fixation of melanism in leopards of the Malay Peninsula. *Journal of Zoology*, 282, pp.201-206.

Kent, V.T. 2011. *The Status and Conservation Potential of Carnivores in Semi-Arid Rangelands, Botswana. The Ghanzi Farmlands: A Case Study*. Durham University.

Khalaf-von Jaffa, N. 2002. Wild Cats in Palestine. Gazelle: The Palestinian Biological Bulletin / www.gazelle.8m.net / [Accessed in November 2002].

Khan, M. 2004. Status and distribution of wild cats and primates in Bangladesh. *Bangladesh Journal of Life Sciences*.

Khorozyan, I. 1999. The leopard in Caucasus and Armenia. Persian leopard (*Panthera pardus saxicolor*). EEP Annual Report 1999.

Khorozyan, I., 2008a. Panthera pardus ssp. saxicolor. *IUCN Red List of Threatened Species*. Available at: http://www.iucnredlist.org/details/15961/0 [Accessed June 1, 2014].

Khorozyan, I., 2008b. Research and Conservation of The Persian Leopard (*Panthera pardus saxicolor*) in Bamu National Park, Fars Province, Iran, Yerevan, Armenia.

Khorozyan, I. 2009. The leopard survey in the Artvin province, northeastern Turkey. Mission report. 8 pp.

Khorozyan, I.G., Malkhasyan, A.G., & Abramov, A. V. 2008. Presence-absence surveys of prey and their use in predicting leopard (*Panthera pardus*) densities: a case study from Armenia. *Integrative Zoology*, 3(4), pp.322–32.

Khorozyan I, Malkhasyan A, Asmaryan S. 2005. The Persian leopard prowls its way to survival. Endangered Species UPDATE, 22(2), pp.51.

Khorozyan, I. Abramov, A. 2007. The leopard, *Panthera pardus*, (Carnivora: Felidae) and its resilience to human pressure in the Caucasus. *Zoology in the Middle East,* 41, pp.11-24.

Khorozyan, I.G., Malkhasyan, A.G., Asmaryan, S.G., & Abramov, A.V. 2010. Using geographical mapping and occupancy modeling to study the distribution of the critically endangered leopard (*Panthera pardus*) population in Armenia. In Cushman, S. A., Huettmann, F. (eds) *Spatial Complexity, Informatics, and Wildlife Conservation*. New York: Springer. pp. 329-347.

Khorozyan, I., Soofi, M., Khaleghi Hamidi, A., Ghoddousi, A., Waltert, M. 2015. Dissatisfaction with Veterinary Services Is Associated with Leopard (*Panthera pardus*) Predation on Domestic Animals. *PLoS ONE* 10(6): e0129221. doi:10.1371/journal.pone.0129221

Kiabi, B.H., Dareshouri , B.F., Ghaemi, R.A., Jahanshahi, M. 2002. Population status of the Persian leopard (*Panthera pardus saxicolor*) in Iran. *Zoology in the Middle East*, 26, pp.41-47.

Kittle, A.M. & A.C. Watson, 2002. Homerange, Demography and Behaviour of the Sri Lankan Leopard (*Panthera pardus kotiya*), Block I Ruhunu (Yala) National Park. Department of Wildlife Conservation and Ministry of Environment and Natural Resources, Biodiversity Secretariat. Colombo, Sri Lanka.

Kittle, A. & Watson, A., 2003. Leopard poaching in Sri Lanka. *Loris*, 23, pp.42–49.

Kittle A, & Watson A. 2005. Report on the status and distribution of the Sri Lankan leopard (*Panthera pardus kotiya*) for the Convention on International Trade of Endangered Species (CITES).

Kittle, A. & Watson, A. 2007a. *The Distribution, Ecology and Behaviour of Leopards in Sri Lanka: Does the Absence of Intra-Guild Competition Matter?* Colombo, Sri Lanka.

Kittle, A., & Watson, A. 2007b. How many leopards are in Sri Lanka?: an estimate from field investigations for the IUCN's global Red Listing.

Kittle, A. & Watson, A. 2008. Panthera pardus ssp. kotiya. The IUCN Red List of Threatened Species 2008: e.T15959A5334064. http://dx.doi.org/10.2305/IUCN.UK.2008.RLTS.T15959A5334064.en. Downloaded on 14 December 2015.

Kittle, A., Watson, A.C., Kumara, P.H.C., & Sanjeewani, H.K.N. 2012. Status and Distribution of the Leopard in central hills of Sri Lanka. *CAT News*, 56, pp.28–31.

Kittle, A.M., Watson, A.C., Kumara, P.H.S.C., Sandanayake, S.D.K.C, Sanjeewani, H.K.N. & Fernando, T.S.P. 2014. Notes on the diet and habitat selection of the Sri Lankan leopard *Panthera pardus kotiya* in the central highlands of Sri Lanka. *Journal of Threatened Taxa*, 6(9), pp. 6214-6221.

Kolowski, J.M. & Holekamp, K.E., 2006. Spatial, temporal, and physical characteristics of livestock depredations by large carnivores along a Kenyan reserve border. *Biological Conservation*, 128(4), pp.529–541.

Kowalski, K. & Rzebik-Kowalska, B. 1991. *Mammals of Algeria*, Wroclaw: Polish Academy of Science, Institute of Systematics and Evolution of Animals.

Laguardia, A., Kamler, J.F., Li, S., Zhang, C., Zhou, Z., & Shi K.. 2015. The current distribution and status of leopards *Panthera pardus* in China. *Oryx*, doi:10.1017/S0030605315000988.

Laidlaw, R.K., Rahman, M.T.A., & Zainal, Z. 2000. Large mammal survey in Krau Wildlife Reserve. *J. Wildl. Parks* (Malaysia). 18, pp.75-106.

Larison, B., Smith, T.B., Derek, G., Stauffer, D., Milá, B., Drewes, R.C., Griswold, C.E., Vindum, J.V., Ubick, D., O'Keefe, K. & Nguema, J. 1999. Biotic Surveys of Bioko and Rio Muni, Equatorial Guinea. Unpublished report to the Biodiversity Support Program, Center for Tropical Research, San Francisco State University, San Francisco.

Li, S., Wang, D.J., Lu, Z. & McShea, W.J. 2010. Cats living with pandas: The status of wild felids within giant panda range, China. *CAT News*, 52, pp.20–23.

Lindsell, J. A., Klop, E. & Siaka, A.M. 2011. The impact of civil war on forest wildlife in West Africa: Mammals in Gola Forest, Sierra Leone. *Oryx*, 45(01), pp.69–77. A

Lindsey, P. & Bento, C., 2011. Illegal hunting and the bushmeat trade in central Mozambique.

Lodhi, A., 2007. Conservation of leopards in Ayubia National Park, Pakistan. University of Montana.

Lukarevsky, V. 1996. Modern Distribution, Location, and Number of Persian Leopard (*Panthera pardus*) in Turkmenia.

Lukarevsky, V. 2001. The leopard, striped hyena and wolf in Turkmenistan. Moscow, Signar.

Lukarevsky, V., Askerov, E. & Hazaryan, G. 2004. Condition of the leopard population in the Caucasus. *Beitr zur Jagd- und Wildforsch*, 29, pp.303–319.

Lukarevsky, V., Akkiev, M., Askerov, E., Agili, A., Can, E., Gurielidze, Z., Kudaktin, A.N., Malkhasyan, A. & Yarovenko, Y.A. 2007. Status of the leopard in the Caucasus. *Cat News*, SI 2, pp.15-21.

Lynam A. J., Round P. D. & Brockelman W. Y. 2006. Status of birds and large mammals in Thai-land's Dong Phayayen – Khao Yai Forest Complex. Biodiversity Research and Training (BRT) Program and Wildlife Conservation Society, Bangkok, Thailand.

Lynam, A.J., Laidlaw, R., Wan Shaharuddin, W.N., Elagupillay, S., & Bennett, E.L. 2007. Assessing the conservation status of the tiger *Panthera tigris* at priority sites in Peninsular Malaysia. *Oryx*, 41, pp.454-462.

Ma, Y.Q. 1998. *Panthera pardus*. In Wang, Sung. (Ed.) *China Red Data Book of Endangered Animals*. pp.116-118. Science Press, Beijing, China (in English and Chinese).

Maan, A., & Chaudhry, A. 2000. Common leopard (*Panthera pardus*) – our endangered heritage needs special conservation. *Tigerpaper*, 27(4).

Madhusudan, M.D. 2003. Living amidst large wildlife: livestock and crop depredation by large mammals in the interior villages of Bhadra Tiger Reserve, South India. *Environ. Management,* 31(4), pp.466–475.

Maisels, F., Keming, E., Kemei, M., & Toh, C. 2001. The extirpation of large mammals and implications for mountain forest conservation: the case of the Kilum-Ijum Forest, Northwest Province, Cameroon. *Oryx,* 35, pp.322-331.

Mallon, D. & Budd, K. 2011. Regional red list status of Carnivores in the Arabian Peninsula, Cambridge, UK; Gland, Switzerland; Sharjah, UAE: Environment and Protected Areas Authority.

Manati, A.R. 2009. The Trade in Leopard and Snow Leopard Skins in Afghanistan. TRAFFIC Bulletin, 22(2), pp.57–58.

Manlius, N. 2001. Les grands mammiferes de l'Egypte: Biogeographie et Ecologie historique. Les Amis du Meseum National d'Histoire Naturelle: 208 pp.

Maputla, N.W., Chimimba, C.T. & Ferreira, S.M. 2013. Calibrating a camera trap-based biased mark-recapture sampling design to survey the leopard population in the N'wanetsi concession, Kruger National Park, South Africa. *African Journal of Ecology*, 51(3), pp.422–430.

Martin & DeMeulenaer, 1988. Status and Distribution of leopard *Panthera pardus* in sub-Saharan Africa. CITES, Geneva, Switzerland.

Masseti, M. 2009. Carnivores of Syria. In: Neubert, E., Amr, Z., Taiti, S., Gümüs, B. (Eds.). Animal biodiversity in the Middle East. Proceedings of the First Middle Eastern Biodiversity Congress, Aqaba, Jordan, 20–23 October 2008. *ZooKeys,* 31, pp.229–252.

Mathot, L., Ikoli, F. and Missilou, B.R. 2006. Rapport annuel de monitoring de la faune du Projet Lésio-Louna, 2006. Projet Lésio-Louna, Fondation John Aspinall, Brazzaville.

Mazzolli, M. 2008. Status of the Arabian leopard (*Panthera pardus nimr*) in Dhofar, Sultanate of Oman. Biosphere Expedition report.

Médard, P., Traoré, M. V. & Berzins, R. 2010. Rapport d'enquete Rencensement des observations de guépards et autres carnivores dans les aires protégées de l'ouest du Burkino Faso. [in French]

Mehmet, 2013. Animal Killed by Shepherd is Anatolian Leopard. *Hurriyet*. Available at: http://www.hurriyet.com.tr/gundem/25037458.asp [Accessed August 1, 2014].

Meijaard, E. 2004. Biogeographic history of the Javan leopard *Panthera pardus* based on a craniometric analysis. *Journal of Mammalogy*, 85(2), pp.302–310.

Melletti, M., Mirabile, M., Penteriani, V. & Boitani, L.,, 2009. Habitat use by forest mammals in Dzanga-Ndoki National Park, Central African Republic. *African Journal of Ecology*, 47, pp.797–800.

Mendelssohn, H. 1989. Felids in Israel. *CAT News*, 10, pp.2-4.

Mésochina, P., Langa, F. & Chardonnet, P. 2008. Preliminary survey of large herbivores in Gilé national reserve, Zambezia Province, Mozambique, Paris.

Mésochina, P., Sefu, L., Sichali, E., Chardonnet, P., Ngalande, J., & Lipita, W. 2010. Conservation status of the lion (*Panthera leo* Linnaeus, 1758) in Malawi, SCI Foundation, DNPW.

Midlane, N., O'Riain, M.J., Balme, G.A., Robinson, H.S., & Hunter, L.T.B. 2014. On tracks: a spoor-based occupancy survey of lion (*Panthera leo*) distribution in Kafue National Park, Zambia. *Biological Conservation,* 172, pp.101–108.

Millington, S., & Tiega, A. 1991. Biological diversity assessment for Niger. WWF Niger.

Miquelle, D., Arzhanova, T., & Solkin, V. 1996. A recovery plan for conservation of the Far Eastern leopard: Results of an international conference held in Vladivostok, Russia. Final report to the USAID Russian Far East Environmental Policy and Technology Project, 1996.

Miquelle, D.G. 1998. A Survey of Tigers and Prey Resources in the Paektusan Area, Lyangan Province, North Korea, in Winter, 1998.

Miquelle, D.G., Darman, Y.A., Aramilev, V.V., Hätte, M., Bereznyuk, S.L., Myslenkov, A.A., Solkin, V.A., Pikunov, D.G., Kostyria, A.V., Christie, S., & Lewis, J. 2010. *A program for reintroduction of the Far Eastern leopard into Southern Sikhote-Alin, Primorsky Krai, Russian Far East*.

Mishra, C., & Fitzherbert, A. 2004. War and wildlife: a post-conflict assessment of Afghanistan's Wakhan Corridor. *Oryx*, 38, pp.102-105.

Mohd Azlan, J., & Sharma, D.S.K., 2006. The diversity and activity patterns of wild felids in a secondary forest in Peninsular Malaysia. *Oryx*, 40, pp.36-41.

Moheb, Z. & Bradfield, D. 2014. Status of the common leopard in Afghanistan. *CAT News*, 61, pp.15–16.

Moqanaki E., Breitenmoser U., Kiabi B. H., Masoud M., & Bensch, S. 2013. Persian leopards in the Iranian Caucasus: a sinking 'source' population? *CAT News,* 59, pp.22-25.

Mondal, K., Gupta, S., Bhattacharjee, S., Qureshi, Q. & Sankar, K. 2012. Response of leopards to re-introduced tigers in Sariska Tiger Reserve, Western India. *International Journal of Biodiversity and Conservation*, *4*(5), pp.228-236. DOI: 10.5897/IJBC12.014.

Morake, Puleng. 2010. Documenting Historical Faunal Change in Lesotho and the Adjoining Eastern Free State of South Africa. Witwatersrand Institutional Repository on DSpace (WIReDSpace): Collections of Electronic Thesis and Dissertations. Elektronisches Dokument: <http://wiredspace.wits.ac.za/handle/10539/8561>

Msuha, M.J. 2009. Human impacts on carnivore biodiversity inside and outside protected areas in Tanzania. Doctoral thesis, UCL (University College London).

Myers, N., 1976. *The Leopard Panthera pardus in Africa*, Morges, Switzerland.

Naing, H., Fuller, T.K., Sievert, P.R., Randhir, T.O., Tha Po, S.H., Maung, M., Lynam, A.J., Htun, S., Thaw, W.N., & Myit, T. 2015. Assessing large mammal and bird richness from camera-trap records in the Hukaung Valley of northern Myanmar. *Raffles Bulletin of Zoology*, 63, pp.376-388.

Ndao, I. & Henschel, P. 2011 Rapport de l'étude sur la population des lions au Parc National Niokolo-Koba, Sénégal. Direction des Parcs Nationaux, Dakar, Senegal.

Ngoprasert, D., Lynam, A.J., Sukmasuang, R., Tantipisanuh, N., Chutipong, W., Steinmetz, R., Jenks, K.E., Gale, G.A., Grassman Jr., L.I., Kitamura, S., Howard, J., Cutter, P., Cutter, P., Leimgruber, P., Songsasen, N., & Reed, D.H. 2012. Occurrence of three felids across a network of protected areas in Thailand: prey, intraguild, and habitat associations. *Biotropica,* 44, pp.810-817.

Niassa Carnivore Project. 2014. Niassa Carnivore Project - Annual Report.

NORAD. 2009. Environmental and Socio-Economic Baseline Study – Malawi, Oslo, Norway: Norad, Evaluation Department.

Norton, P.M. 1990. How many leopards? A criticism of Martin and de Meulenaer's population estimate for Africa. *South African Journal of Scienc*e, 86, pp.218-220.

Nowell, K. & Jackson, P. 1996. *Wild Cats - Status Survey and Conservation Action Plan*, Gland, Switzerland: IUCN.

O'Brien, T. G., & M. F. Kinnaird. 2011. Density estimation of sympatric carnivores using spatially explicit capture–recapture methods and standard trapping grid. *Ecological Applications,* 21, pp.2908–2916.

Odden, M., Wegge, P., & Fredriksen, T. 2010. Do tigers displace leopards? If so, why? *Ecological Research,* 25(4), pp.875-881. DOI: 10.1007/s11284-010-0723-1.

Ogada, M.O., Woodroffe, R., Oguge, N.O. & Frank, L.G. 2003. Limiting Depredation by African Carnivores: the Role of Livestock Husbandry. *Conservation Biology*, 17(6), pp.1521–1530.

Okon, D. 2005. Report on Old Ekuri Reconnaissance Survey of Red Colobus. Wildlife Conservation Society, Calabar, Nigeria.

Okello, M.M., Bonham, R. & Hill, T. 2014. The pattern and cost of carnivore predation on livestock in maasai homesteads of Amboseli ecosystem, Kenya : Insights from a carnivore compensation programme. *International Journal of Biodiversity and Conservation*, 6(7), pp.502–521.

Okello, M.M., Kiringe, J.W. & Warinwa, F. 2014. Human-Carnivore Conflicts in Private Conservancy Lands of Elerai and Oltiyiani in Amboseli Area, Kenya. *Natural Resources*, 05(08), pp.375–391.

Osborn, D.J. & Helmy, I. 1980. Felidae. In *The Contem*. Available at: http://www.catsg.org/cheetah/05_library/5_3_publications/N_and_O/Osborn_&_Helmy_1980_Contemporary_mammals_of_Egypt_-_Felidae.pdf.

Ososky, J.J. 1998. Diet of leopards and golden cats in Ndoki Park, Republic of Congo. MSc. Thesis. Northern Illinois University.

Owen, C., Niemann, S. & Slotow, R. 2010. Copulatory parameters and reproductive success of wild leopards in South Africa. *Journal of Mammalogy*, 91(5), pp.1178–1187.

Packer, C., Brink, H., Kissui, B., Maliti, H., Kushnir, H., & Caro, T. 2010. Effects of Trophy Hunting on Lion and Leopard Populations in Tanzania. *Conservation Biology,* 25(1), pp.142-153.

Patterson, B.D., Kasiki, S.M., Selempo, E. & Kays, R.W. 2004. Livestock predation by lions (*Panthera leo*) and other carnivores on ranches neighboring Tsavo National Parks, Kenya. *Biological Conservation*, 119(4), pp.507–516.

Perez, I., Geffen, E. & Mokady, O. 2006. Critically Endangered Arabian leopards *Panthera pardus nimr* in Israel: estimating population parameters using molecular scatology. *Oryx*, 40(03), pp.295.

Phillips, W.W.A. 1935 (revised 1980). Manual of the Mammals of Ceylon. Wildlife and Nature Protection Society of Sri Lanka.

Phillips, W.W.A. 1984. *Manual of the Mammals of Sri Lanka*, *part III*. 2nd Edition. Colombo.

Pikunov, D.G., & Korkishko, V.G. 1985. Present distribution and numbers of leopards (*Panthera pardus*) in the Far East USSR. *Zoology Journal* 64, pp.897–905. [in Russian]

Pikunov, D.G., Abramov, V.K., Korkishko, V.G., Nikolaev, I.G., & Belov, A.I. 2000. A survey of Far Eastern leopards and Amur tigers in Southwest Primorye Krai, in 2000. Pacific Institute of Geography, Far Eastern Branch of Russian Academy of Sciences / Institute of Biology and Soils, Far Eastern Branch of Russian Academy of Sciences / Institute for Sustainable Use of Natural Resources.

Pikunov, D.G., Miquelle, D.G., Abramov, V.K., Nikolaev, I.G., Seredkin, I.V., Murzin, A.A. & Korkishko, V.G., 2003. *A survey of Far Eastern leopard and Amur tiger populations in southwest Primorski Krai, Russian Far East (February 2003)*, Vladivostok, Russia.

Pishvaei, H. 2014. A preliminary study on the status of human-carnivore conflicts in Hormozgan Province. *Persian Wildlife Heritage Foundation Newsletter*, 4(9), pp.7.

Purchase, G., Mateke, C. & Purchase, D. 2007. A review of the status and distribution of carnivores , and levels of human- carnivore conflict, in the protected areas and surrounds of the Zambezi Basin, Famona, Bulawayo, Zimbabwe.

Purroy, P. 2010. *El leopardo del Atlas: salsero y otras andanzas.* Edilesa. [in Spanish]

Qarqaz, M. & Baker, M.A. 2006. The Leopard in Jordan. *CAT News*, SI 1, pp.9-10.

Qi, J., Shi, Q., Wang, G., Li, Z., Sun, Q., Hua, Y. & Jiang, G. 2015. Spatial distribution drivers of Amur leopard density in northeast China. *Biological Conservation*, 191, pp.258-265.

Qumsiyeh, M.B. 1996. *Mammals of the Holy Land*. Texas Tech University Press, Lubbock, Texas, USA.

Qumsiyeh, M.B., Amr, Z.S. & Shafei, D.M. 1993. Status and conservation of carnivores in Jordan. *Mammalia*, 57(1), pp.55–62.

Rabinowitz, A. 1989. The density and behavior of large cats in a dry tropical forest mosaic in Huai Kha Khaeng Wildlife Sanctuary, Thailand. *Nat. Hist. Bull. Siam Soc*. 37, pp.235-251.

Ranganathan, M. 2001. India's wildlife history. Ranthambhore Foundation.

Ray, J.C. & Sunquist, M.E. 2001. Trophic relations in a community of African forest carnivores. *Oecologica*, 127, pp.397–408.

Ray, J.C., Hunter, L. & Zigouris, J. 2005. *Setting conservation and research priorities for larger African carnivores*. WCS Working Paper No. 24. Wildlife Conservation Society, New York.

Ray, R. 2011. Ecology and population status and the impact of trophy hunting of the leopard *Panthera pardus* (Linnaeus , 1758) in the Luambe National Park and surrounding Game Management Areas in Zambia. Rheinische Friedrich-Wilhelms-Universität.

Rayan, D.M., Mohamad, S., Wong, C., Sagtia Siwan, E., Fong Lau, C., Hamirul, M., & Mohamed, A. 2013. Conservation status of tigers and their prey in the Belum-Temengor Forest Complex. WWF-Malaysia, Kuala Lumpur.

Raza, H.A., Abdulhasan, N., Ararat, K., Qadir, M. & Ali, L. 2012a. Conserving Wild Goats at Qara Dagh and Permagroon Mountains, Iraq.

Raza, H.A., Ahmad, S.A., Hassan, N.A., Ararat, K., Qadir, M. & Ali, L. 2012b. First photographic record of the Persian leopard in Kurdistan, northern Iraq. *CAT News*, 56, pp. 34-35.

Raza, R.H., Chauhan, D.S., Pasha, M.K.S. & Sinha, S. 2012c. *Illuminating the Blind Spot: A Study on Illegal Trade in Leopard Parts in India*, Available at: http://www.ncbi.nlm.nih.gov/pubmed/23894877.

Reinl, J. 2007. Back from the wilderness. Emirates Today, 11 February 2007. Frontpage and pp.18-20.

Riggio, J., Jacobson, A., Dollar, L., Bauer, H., Becker, M., Dickman, A., Funston, P., Groom, R., Henschel, P., de Iongh, H., Lichtenfeld, L, & Pimm, S. 2013. The size of savannah Africa: a lion's (*Panthera leo*) view. *Biodiversity and Conservation*, 22(1), pp.17–35. Available at: http://link.springer.com/10.1007/s10531-012-0381-4 [Accessed February 25, 2014].

Roberts, T.J. 1997. *The Mammals of Pakistan*. Oxford University Press. 525 pp.

Rogers, G. 2008. Study raises concerns over leopard numbers. The Herald online, Port Elizabeth, 29 October 2008 / www.theherald.co.za/herald/news/n21_28102008.htm / Accessed in November 2009.

Romañach, S.S., Lindsey, P. A. & Woodroffe, R. 2007. Determinants of attitudes towards predators in central Kenya and suggestions for increasing tolerance in livestock dominated landscapes. *Oryx*, 41, pp.185-195.

Rostro-García, S., Kamler, J. F., Ash, E., Clements, G. R., Gibson, L., Lynam, A. J., McEwing, R.,Naing, H., & Paglia, S. 2016. Endangered leopards: range collapse of the Indochinese leopard (*Panthera pardus delacouri*) in Southeast Asia. *Conservation Biology*.

Roulet, P.A., Pelissier, C., Patek, G., Beina, D., & Ndallot, J. 2007. Projet Zemongo - Un aperçu du contexte écologique et de la pression anthropique sur les ressources naturelles de la Réserve de Faune de Zemongo, Préfecture du Haut-Mbomou, République Centrafricaine. Rapport final de la mission du 15 janvier au 19 mars 2006, MEFCP, Bangui, pp. 85. [in French]

Ryan, J., & Attuquayefio, D. 2000. Mammal fauna of the Muni-Pomadze Ramsar site, Ghana. *Biodiversity and Conservation,* 9, pp.541–560.

Saleh, M.A., Salim, M.I.B., & Bedare, S.M. 1995. Ecological survey of south Sinai protected areas - land vertebrates. Internal Publication Al Azhar University, Egypt.

Sanei, A. & Zakaria, M. 2011. Distribution pattern of the Persian leopard (*Panthera pardus saxicolor*) in Iran. *Asia Life Sciences Supplement*, 7, pp.7–18.

Sangay, T. & Vernes, K. 2008. Human–wildlife conflict in the Kingdom of Bhutan: Patterns of livestock predation by large mammalian carnivores. *Biological Conservation*, 141(5), pp.1272–1282.

Santiapillai, C., Chambers, M.R. & Ishwaran, N. 1982. The leopard *Panthera pardus fusca* (meyer 1794) in the Ruhuna national park, Sri Lanka, conservation. *Biological Conservation*, 23(1), pp.5-14.

Santiapillai, C. and Jayewardene, R. 2004. Conservation of the leopard and other carnivores in Sri Lanka. *Current Science-Bangalore-*, 86(8), pp.1063-1064.

Santiapillai, C. & Ramono, W.S. 1992. Status of the Leopard in Java, Indonesia. *Tiger Paper*, 19(2), pp.1–5.

Sayer, J. 1977. Conservation of large mammals in the Republic of Mali. *Biological Conservation*, Vol. 12: 245-63.

Secher, K. 2013. Critically endangered Javan leopard caught on camera. *ScienceNordic*. Available at: http://sciencenordic.com/critically-endangered-javan-leopard-caught-camera.

Selvan, K.M., Lyngdoh, S., Habib, B., & Gopi, G.V. 2014. Population density and abundance of sympatric large carnivores in the lowland tropical evergreen forest of Indian Eastern Himalayas. *Mammalian Biology-Zeitschrift für Säugetierkunde*, *79*(4), pp.254-258.

Shakula, V. 2004. First record of leopard (*Panthera pardus*) in Kazakhstan. *CAT News,* 41, pp.11-12.

Shashe Limpopo Predator Research Group. 2010. The status of large carnivores of the greater Mapungubwe Transfrontier Conservation Area: 2004-2010. Shashe Limpopo Predator Research Group. Bulawayo, Zimbabwe.

Shehzad, W., Nawaz, M.A., Pompanon, F., Coissac, E., Riaz, T., & Shah, S.A., & Taberlet, P. 2015. Forest without prey: livestock sustain a leopard *Panthera pardus* population in Pakistan. *Oryx,* 49(2), pp. 248–253. doi: 10.1017/s0030605313001026.

Shepherd, C., & Nijman, V. 2008. The wild cat trade in Myanmar. TRAFFIC Southeast Asia, Petaling Jaya, Selangor, Malaysia.

Shoemaker, A. 1993. The status of the leopard, *Panthera pardus*, in nature. A country by country analysis. Riverbanks Zoological Park, Columbia, South Carolina, USA.

Shortridge, G.C. 1934. *The Mammals of South West Africa*. Vols 1 & 2. London, Heinemann.

Shrestha, T.K. 1997. *The Mammals of Nepal*. R.K. Printers, Kathmandu, Nepal.

Simcharoen, S., Barlow, A.C., Simcharoen, A., & Smith, J.L. 2008. Home range size and daytime habitat selection of leopards in Huai Kha Khaeng Wildlife Sanctuary, Thailand. *Biological Conservation*, 141(9), pp.2242–2250.

Smith, A.T., Xie, Y., Hoffmann, R.S., Lunde, D., MacKinnon, J., Wilson, D.E. & Wozencraft, W.C. (Eds), 2010. *A guide to the mammals of China*. Princeton University Press.

Smithers, R.H.N. 1971. *The Mammals of Botswana. Museum Memoir No. 4.* The Trustees of the National Museums of Rhodesia, Salisbury, Rhodesia.

Sogbohossou, E. 2006. Conservation des grands carnivores Afrique de l'Ouest: Perception par les populations et commerce des sous-produits. Wildlife Conservation Society. [in French]

Sokov, A.I. 1990. The present status of the snow leopard population in the south-western Pamir-Altai mountains (Tadzhikistan). In *Int. Ped. Book of Snow leopards*. pp. 33–36.

Sommerlatte, M., & Umar, A. 2000. An Ecological Assessment of the Coastal Plains of North Western Somalia (Somaliland). Somali Natural Resources Management Programme, IUCN Eastern Africa Programme.

Soultan, A., Attum, O., Hamada, A., Hatab, E-B., Ahmed, S.E., Eisa, A., Al Sharif, I., Nagy, A. & Shohdi, W. 2016. Recent observaiton for leopard *Panthera pardus* in Egypt. *Mammalia*: DOI:10.1515/mammalia-2015-0089.

Spalton, J.A. & al Hikmani, H.M. 2006. The Leopard in the Arabian Peninsula - Distribution and Subspecies Status. *CAT News*, SI 1, pp.4–8.

Spalton, J.A., Al Hikmani, H.M., Willis, D. & Said, A.S.B. 2006a. Critically Endangered Arabian leopards *Panthera pardus nimr* persist in the Jabal Samhan Nature Reserve, Oman. *Oryx*, 40(03), pp.287.

Spalton, J.A., Al Hikmani, H.M., Jahdhami, M.H., Ibrahim, A.A., Said, A.S.B. and Willis, D. 2006b. Status Report for the Arabian Leopard in the Sultanate of Oman. *CAT News,* SI 1, pp. 26-32.

Spalton, A. & al Hikmani, H.M. 2014. *The Arabian Leopards of Oman*, London: Stacey International, Limited.

Spitzen, V.V., Miquelle, D.G., Darman, Y.A., Aramilev, V.V., Hötte, M., Bereznyuk, S.L., Laptev, A.A., Aramileva, T.S., Myslenkov, A.A., Kerley, L.L., Salkina, G., Arzjanova, T.D., Solkin, V.A., Pikunov, D.G., Fomenko, P.V., Aramelev, S., Kostyria, A.V. Uphyrkina, O., Christie, S., Lewis, J., Hebblewhite, M., Seidensticker, J., Breitenmoser, U., & Lukas, J. 2012. For reintroduction of the Far Eastern Leopard into Southern Sixhote-Alin, Primorskii Krai, Russian Far East.

Spong, G., Johansson, M., & Björklund, M. 2000. High genetic variation in leopards indicates large and long-term stable effective population size. *Molecular Ecology*, Vol. 9/11.

Stander, P.E. 1998. Spoor counts as indices of large carnivore populations: the relationship between spoor frequency, sampling effort and true density. *Journal of Applied Ecology*, 35(3), pp.378-385.

State Forestry Administration. 2009. National Survey of China's Key Terrestrial Wildlife Species. China Forestry Publishing House, Beijing, China.

Stead, M., Rorison, S. & Scafidi, O. 2013. *Angola* 2nd ed., Guilford, Connecticut: Globe Pequot Press Inc.

Stein, A.B., Fuller, T.K., DeStefano, S. & Marker, L.L., 2011. Leopard population and home range estimates in north-central Namibia. *African Journal of Ecology*, 49, pp.383–387.

Stein, A., Andreas, A. & Aschenborn, O. 2012. *Namibian National Leopard Survey - 2011 Final Report*. Ministry of Environment and Tourism, Namibia. pp.41.

Stein, A.B., Aschenborn, O., Kastern, M., Andreas, A., & Thompson, S. 2012. *Namibia Large Carnivore Atlas*. Ministry of Environment and Tourism, Namibia. pp.12.

Stein, A.B., & Hayssen, V. 2013. *Panthera pardus* (Carnivora: Felidae). *Mammalian Species,* 900(1), pp.30-48. doi:10.1644/900.1.

Steinmetz, R., Seuaturien, N., Chutipong, W., & Poonnil, B. 2009. The ecology and conservation of tigers and their prey in Kuiburi National Park, Thailand. WWF Thailand, and Department of National Parks, Wildlife, and Plant Conservation, Bangkok.

Stevens, K., Dehgan, A., Karlstetter, M., Rawan, F., Tawhid, M.I., Ostrowski, S., Ali, J.M. & Ali, R. 2011. Large mammals surviving conflict in the eastern forests of Afghanistan. *Oryx*, 45(02), pp.265–271.

Strampelli, P. 2015. Status and habitat use responses of leopard (*Panthera pardus*) in a human impacted region of rural Mozambique. MSc Thesis. Imperial College London.

Stuart, C. & Stuart, T. 2001. *Field guide to mammals of southern Africa*. Struik.

Stuart, C., & Wilson, V. 1988. *The cats of Southern Africa*. Chipangali Wildlife Trust, Bulawayo, Zimbabwe.

Svengren, H., 2008. Leopard survey in Sangare Ranch Conservancy, Kenya, using noninvasive methods including individual identification from faecal DNA. MSc Thesis. Uppsala University.

Swanepoel, L.H., Lindsey, P., Somers, M.J., Hoven, W.V. & Dalerum, F. 2013. Extent and fragmentation of suitable leopard habitat in South Africa. *Animal Conservation*, 16(1), pp.41–50.

Tan, C.K.W., Moore, J., bin Saaban, S., Campos-Arceiz, A., & Macdonald, D. W. 2015. The discovery of two spotted leopards (*Panthera pardus*) in Peninsular Malaysia. *Tropical Conservation Science*, 8, pp.732-737.

Teer, J.G. & Swank, W.G. 1977. *Status of the leopard in Africa South of the Sahar*a. Washington, D.C.

Teleki, G. 1980. Hunting and trapping wildlife in Sierra Leone: Aspects of exploitation and exportation. Unpublished Report to World Wildlife Fund (WWF, US), Washington, DC, USA.

Tempa, T., Hebblewhite, M., Mills, L.S., Wangchuk, T.R., Norbu, N., Wangchuk, T., Nidup, T., Dendup, P., Wangchuk, D., Wangdi, Y. & Dorji, T. 2013. Royal Manas National Park, Bhutan: a hot spot for wild felids. *Oryx*, 47(02), pp.207–210.

Thapa, K., Pradhan, N.M.B., Barker, J., Dhakal, M., Bhandari, A.R., Gurung, G.S., Rai, D.P., Thapa, G.J., Shrestha, S. & Singh, G.R. 2013. High Elevation record of a leopard cat in the Kangchenjunga Conservation Area, Nepal. *CAT News*, (58), pp.26–27.

Thapa, K., Shrestha, R., Karki, J., Thapa, G.J., Subedi, N., Pradhan, N.M.B., Dhakal, M., Khanal, P. & Kelly, M.J., 2014. Leopard *Panthera pardus fusca* Density in the Seasonally Dry, Subtropical Forest in the Bhabhar of Terai Arc, Nepal. *Advances in Ecology*. http://dx.doi.org/10.1155/2014/286949.

Tien, D. Van. n.d. The Status of the Leopard, *Panthera pardus*, in Vietnam. Hanoi, Vietnam.

Timna, Y. 2000. Leopards of the Judean Desert. The Society for the Protection of Nature in Israel & Israel Nature and Parks Authority, Tel Aviv, Israel. [in Hebrew]

Toni, P., & Lodé, T. 2013. Fragmented Populations of Leopards in West-Central Africa: Facing an Uncertain Future. *African Zoology*, 48(2), pp.374–387.

T-PVS. 2010. Large Carnivore in the Caucasus. Convention on the conservation of European wildlife and natural habitats. Strasbourg, Tbilssi, Georgia. May 18, 2010.

Treves, A., Mwima, P., Plumptre, A. & Isoke, S. 2010. Camera-trapping forest-woodland wildlife of western Uganda reveals how gregariousness biases estimates of relative abundance and distribution. *Biological Conservation,* 143, pp.521-528.

UEMOA/PNUD. 2014. Inventaire des grands carnviores de l'écosystème W-Arly-Pendjari. UEMOA/PNUD, Ougadougou, Burkina Faso. [in French]

Uganda Wildlife Authority, 2010. *Strategic Action Plan for Large Carnivore Conservation in Uganda.*

UNEP. 2007. Sudan: Post-Conflict Enviromental Assessment, Nairobi, Kenya.

Uphyrkina O., Johnson, W.E., Quigley, H., Miquelle, D., Marker, L., Bush, M., & O'Brien, S.J. 2001. Phylogenetics, genome diversity and origin of modern leopard (*Panthera pardus*). *Molecular Ecology,* 10, pp.2617–2633.

USAID, 2008. Mali biodiversity and tropical forests. 118/119 Assessment. pp. 1-78.

van Helvoort, B.E., de Iongh, H.H. & van Bree, P.J.H. 1985. A Leopard Skin and Skull *(Panthera pardus L.)* from Kangean Island, Indonesia. Z. Säugetierk*.,* 50, pp.182-184.

Verschuren, J. 1987. Liste commentée des Mammifères des Parcs Nationaux du Zaïre, du Rwanda et du Burundi. Bull. Institut Royal des Sciences Naturelles de Belgique, Biologie, 57, pp.17-39.

Vijayan, S. & Pati, B.P., 2002. Impact of Changing Cropping Patterns on Man-Animal Conflicts around Gir Protected Area with Specific Reference to Talala Sub-District, Gujarat, India. *Population and Environment*, 23(6), pp.541–559.

Vogt, M. 2011. Results of Sapo National Park Bio-Monitoring Program 2007-2009. FDA and FFI/Liberia, Monrovia, Liberia.

Wang, S.W. & Macdonald, D.W. 2006. Livestock predation by carnivores in Jigme Singye Wangchuck National Park, Bhutan. *Biological Conservation*, 129(4), pp.558-565.

Wang, S., & Macdonald, D. 2009. The use of camera traps for estimating tiger and leopard populations in the high altitude mountains of Bhutan. *Biological Conservation*, 142(3), pp.606–613.

Wang, T., Feng, L., Mou, P., Wu, J., Smith, J.L.D., Xiao, W., Yang, H., Dou, H., Zhao, X., Cheng, Y., Zhou, B., Wu, H., Zhang, L., Tian, U., Guo, Q., Kou, X., Han, X., Miquelle, D.G., Oliver, C.D., Xu, R. & Ge, J. 2015. Amur tigers and leopards returning to China: direct evidence and a landscape conservation plan. *Landscape Ecology*. DOI: 10.1007/s10980-015-0278-1.

Wangchuk, T. 2004. *A Field Guide to the Mammals of Bhutan*. Department of Forestry, Ministry of Agriculture, Royal Government of Bhutan. Thimphu, Bhutan.

Waseem, M. & Kabir, M., 2010. Human - leopard conflict Assessment in and around Pir Lasora National Park Dist. Kotli, AJ&K.

Wegge, P., Odden, M., Pokharel, C.P., & Storaas, T. 2009. Predator–prey relationships and responses of ungulates and their predators to the establishment of protected areas: a case study of tigers, leopards and their prey in Bardia National Park, Nepal. *Biological Conservation*, *142*(1), pp.189-202.

Willcox, D., Tran, Q.P., Hoang, M.D., & Nguyen, T.T.A. 2014. The decline of non-*Panthera* cat species in Vietnam. *CAT News* S8, 53-61.

Williams, S.T. 2011. The impact of land reform in Zimbabwe on the conservation of cheetahs and other large carnivores. PhD Thesis. Durham University.

Wilson, V. 2006. Survey of the distribution and status of leopard (*Panthera pardus*) & cheetah (*Acinonyx jubatus*) in Zimbabwe. Chipangali Wildlife Trust, Carnivore Research Institute / Zimbabwe Parks and Wildlife Management Authority.

Winterbach, H.E.K., Winterbach, C.W. & Somers, M.J. 2014. Landscape suitability in Botswana for the conservation of its six large African carnivores. *PLoS ONE*, 9(6), pp.1–12.

Woodroffe, R., Lindsey, P.A., Romañach, S.S. & ole Ranah, S.M. 2007. African wild dogs (*Lycaon pictus*) can subsist on small prey: implications for conservation. *Journal of Mammalogy*, 88(1), pp.181–193.

WWF, 2015. Amur Leopard—World's Rarest Cat—Doubles in Population. World Wildlife Fund - Russia. Available at: http://www.worldwildlife.org/stories/amur-leopard-world-s-rarest-cat-doubles-in-population.

Yang, C.M., Yong, K., & Lim. K.K.P. 1990. Wild mammals of Singapore. In: L.M. Chou and P.K.L. Ng, eds. *Essays in Zoology*, pp. 32-41. Zoology Department, National University of Singapore, Singapore.

Yang et al. 2016. Reconstructing the historical distribution of Amur leopards (*P.p.orientalis*) in Northeast China based on historical records. *ZooKeys* (submitted).

Yarovenko, Y. n.d. *GIS mapping of habitats and further field research to identify the priority leopard (Panthera pardus) areas in Dagestan, Russian North Caucasus*. Final report submitted to Rufford Foundation. 14pp.

Yeakel, J.D., Pires, M.M., Rudolf, L., Dominy, N.J., Koch, P.L., Guimarães, P.R. & Gross, T. 2014. Collapse of an ecological network in Ancient Egypt. Proceedings of the National Academy of Sciences of the United States of America, 111(40), pp.14472-14477. doi:10.1073/pnas.1408471111.

Yimer, D. & Yirga, S. 2013. Mammals of the Mazie national park, southern nations, nationalities and peoples regional state, Ethiopia. *Ethiopian Journal of Science*, 36(1), pp.55–61.

Yirga, G. & Bauer, H. 2011. Farmers' Perception of Leopard (*Panthera pardus*) Conservation in a Human Dominated Landscape in Northern Ethiopian Highlands. *Mediterranean Journal of Social Sciences*, 2, pp.95–102.

Zafar-Ul Islam, M., Boug, A., As-Shehri, A., & Al Jaid, M. 2014. Poisoning of endangered Arabian leopard in Saudi Arabia and its conservation efforts. *CAT News,* 60, pp.16-17.

Zaw, T., Myint, T., Htun, S., Po, S., Latt, K., Maung, M. & Lynam, A.J. 2014. Status and distribution of smaller felids in Myanmar. *CAT News*, SI 8, pp.24–30.

Ziaie H. 2008. *A Field Guide to the Mammals of Iran*. Iranian Wildlife Center, Tehran, 420 pp. [In Persian]

Ziegler, S., Nikolaus, G. & Hutterer, R., 2002. High mammalian diversity in the newly established National Park of Upper Niger, Republic of Guinea. *Oryx*, 36(1), pp.73–80.

Zimmermann, F., Lukarevsky, V.S., Beruchashvili, G., Breitenmoser-Würsten, C. & Breitenmoser, U. 2007. Mapping the vision - Potential living space for the leopard in the Caucasus. *Cat News*, SI 2, pp.28-33.

Zinner, D., Peláez, F., & Torkler, F. 2001. Distribution and habitat associations of baboons (*Papio hamadryas*) in central Eritrea. *International Journal of Primatology*, 22(3).
