# Supplementary material for: Leopard (Panthera pardus) status, distribution, and the research efforts across its range
Source: PeerJ. 2016 May 4;4:e1974. doi: 10.7717/peerj.1974 (PMC4861552; doi:10.7717/peerj.1974)
Supplement: Supplemental Information 5 — The number of peer-reviewed publications by year regarding wild leopard ecology or conservation between 2000 and 2014. [file peerj-04-1974-s005.pdf]

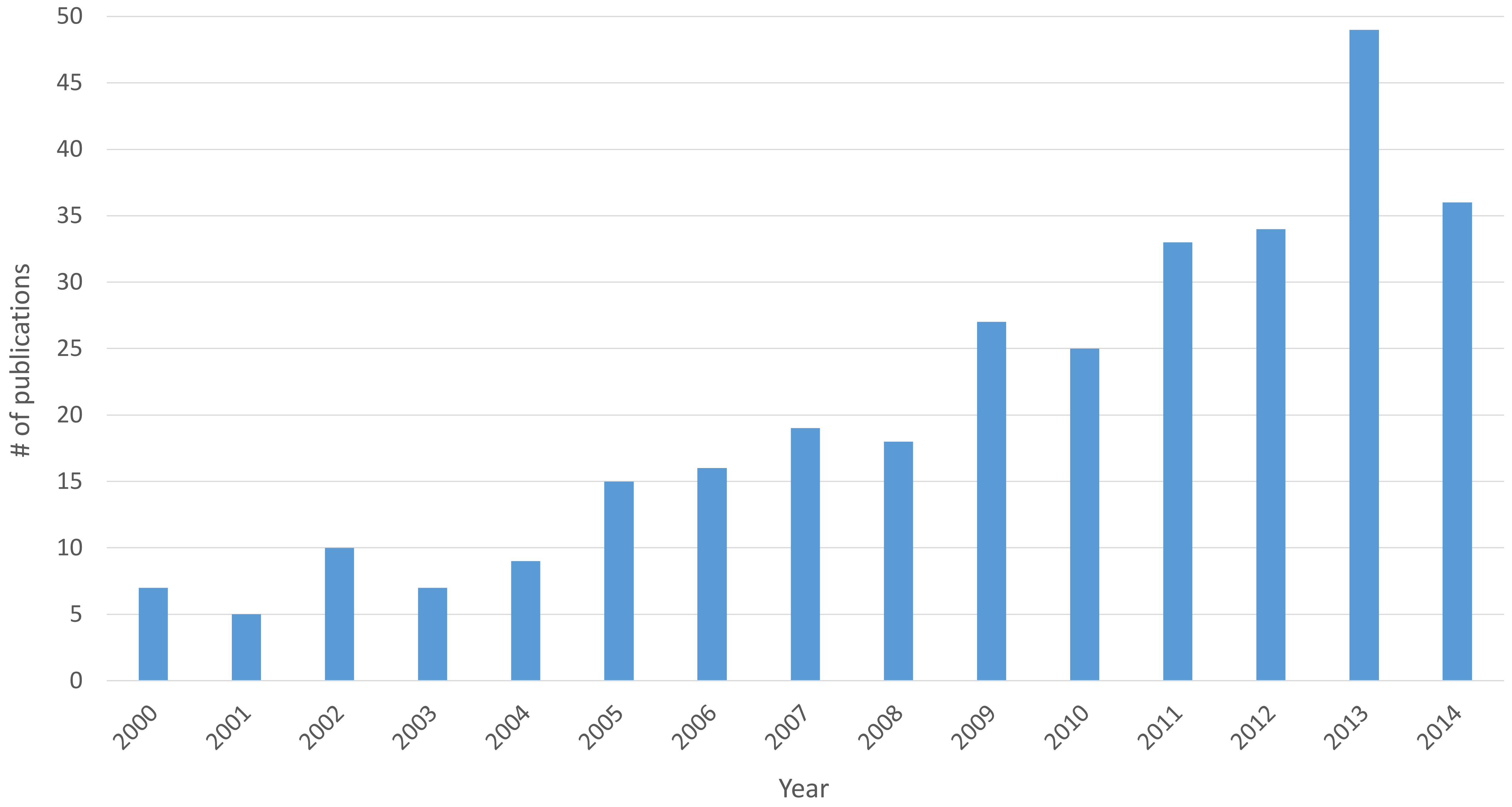

50
45
40
35
30
25
20
15
10
5
0
# of publications
2000
2001
2002
2003
2004
2005
2006
2007
2008
2009
2010
2011
2012
2013
2014
Year
